# Genomic Landscape of the Mitochondrial Genome in the United Arab Emirates Native Population

**DOI:** 10.3390/genes11080876

**Published:** 2020-08-01

**Authors:** Fatma A. Aljasmi, Ranjit Vijayan, Naganeeswaran Sudalaimuthuasari, Abdul-Kader Souid, Noushad Karuvantevida, Raja Almaskari, Hidaya Mohammed Abdul Kader, Biduth Kundu, Khaled Michel Hazzouri, Khaled M. A. Amiri

**Affiliations:** 1 Pediatric Department, United Arab Emirates University, Al Ain, Abu Dhabi 15551, UAE; aljasmif@uaeu.ac.ae (F.A.A.); asouid@uaeu.ac.ae (A.-K.S.); 2 Biology Department, United Arab Emirates University, Al Ain, Abu Dhabi 15551, UAE; ranjit.v@uaeu.ac.ae (R.V.); kvnoushad@me.com (N.K.); r.almeskari@uaeu.ac.ae (R.A.); 201790053@uaeu.ac.ae (H.M.A.K.); biduth.k@uaeu.ac.ae (B.K.); 3 Khalifa Center for Genetic Engineering and Biotechnology, United Arab Emirates University, Al Ain, Abu Dhabi 15551, UAE; naganeeswaran@uaeu.ac.ae (N.S.); khaled_hazzouri@uaeu.ac.ae (K.M.H.)

**Keywords:** Mitochondrial DNA, next generation sequencing, Selection, heteroplasmy, demography, single nucleotide polymorphism (SNP)

## Abstract

In order to assess the genomic landscape of the United Arab Emirates (UAE) mitogenome, we sequenced and analyzed the complete genomes of 232 Emirate females mitochondrial DNA (mtDNA) within and compared those to Africa. We investigated the prevalence of haplogroups, genetic variation, heteroplasmy, and demography among the UAE native population with diverse ethnicity and relatively high degree of consanguinity. We identified 968 mtDNA variants and high-resolution 15 haplogroups. Our results show that the UAE population received enough gene flow from Africa represented by the haplogroups L, U6, and M1, and that 16.8% of the population has an eastern provenance, depicted by the U haplogroup and the M Indian haplogroup (12%), whereas western Eurasian and Asian haplogroups (R, J, and K) represent 11 to 15%. Interestingly, we found an ancient migration present through the descendant of L (N1 and X) and other sub-haplogroups (L2a1d and L4) and (L3x1b), which is one of the oldest evolutionary histories outside of Africa. Our demographic analysis shows no population structure among populations, with low diversity and no population differentiation. In addition, we show that the transmission of mtDNA in the UAE population is under purifying selection with hints of diversifying selection on ATP8 gene. Last, our results show a population bottleneck, which coincides with the Western European contact (1400 ybp). Our study of the UAE mitogenomes suggest that several maternal lineage migratory episodes liking African–Asian corridors occurred since the first modern human emerges out of Africa.

## 1. Introduction

There are two potential routes to the Arabian Peninsula, the northern and southern route, which is the first step out of Africa. This is the primary link between Africa and Eurasia. The maternally inherited mtDNA has been used as a marker to relate lineages across geographic origins culminating in African haplogroup L and the Eurasian M and N, which shared a common route with the African L3, and this radiation likely started the Eurasian colonization [1,2,3]. Furthermore, the star shape radiation in the Indian and East Asian M lineage supports a fast southern dispersal [2]. Previous studies highlight the presence of autochthone M and N lineages along the southern route [3,4,5,6,7]. As a result, the M and N lineages have a unique migration trail [8,9] and the southern coastal trail was the only route for the western Eurasian colonization, which is an early sprout of the southern radiation in India [3,10]. Under these scenarios, the Arabian Peninsula, an obliged link between East Africa and South Asia, attracted a lot of attention. Indeed, several mtDNA studies have been published from this region [11,12,13] and the majority of these studies point to more recent African, Asian, or northern Neolithic origins. Kivisild et al. 2004 defined a new group, L6, with no match to African populations, suggesting an ancient migration from Africa to Yemen. This suggests that ancient migration via the southern route to the neighboring countries such as Oman and UAE is plausible.

Demographic history, such as effective population size changes, short and long distance migrations, as well as admixture, shape the genetic variation of modern African populations. This is in addition to selection on specific loci, combined with recombination and mutation. For instance, one of the migrations that impacts the genetic variation in modern African populations is the migration of agricultural Bantu speaking from West Africa throughout sub-Saharan Africa 4000 years ago followed by admixture with indigenous populations [14,15,16,17,18]. Compared to non-African populations, African populations have higher genetic diversity, population substructure, and low linkage disequilibrium (LD) [19]. In addition, they have evolved an adaptive response to various diets and climate change. Thus far, the evolutionary force(s) that shape the genetic variation and diversity of the UAE population and how they compare to the African population are not known.

Almost in all cases, nuclear DNA is used to describe the signature of selection, while ignoring its effect on the tissue, cells, and subcellular compartments. This is very crucial for mitogenome, which in contrast to the nucleus, serves as a powerhouse for the cell and are present in multiple copies in the cytoplasm that may vary in sequence (heteroplasmy) and quantity among tissues [20]. Each mitochondrion is maternally inherited and codes for enzymes that are mainly involved in cellular bioenergetics [21]. As it is a vital compartment for the generation of cellular metabolism, including ATP production, nucleotide biosynthesis, and other activities, any dysfunction will lead to tissue and systemic disorders [20]. Therefore, strong purifying negative selection acts to remove deleterious mutations, and in parallel, positive selection acts on the mitochondria to promote adaptation of cells, and in return the whole organism, to environmental and physiological changes [20,22,23]. Furthermore, due to the small mitochondria genome size (16.5 Kb), and with the advances in next-generation sequencing, it is facile to get high-throughput information from hundreds of individuals. The information generated from the human mitogenome data help in addressing population evolutionary history and quantify the genetic variation and its effect, which are relevant to metabolic, genetic, and forensic fields [24,25].

The human mitogenome is highly polymorphic, and most of its variants are benign [26]. However, deleterious variants have been reported in various diseases, including Leber hereditary optic nephropathy (LHON); the familial mitochondrial cytopathies; mitochondrial encephalomyopathy, lactic acidosis, and stroke-like episodes (MELAS); and many others [27]. The higher rate of accumulation of deleterious mutations in the mitogenome is due to the small effective population size associated with its haploid inheritance [28]. In addition, heteroplasmy levels of mutations are of importance. For instance, in the 1980s, the first study of heteroplasmy in the mitogenome showed different levels of mutations (e.g., deletions and point mutations) in affected patients [29,30]. Heteroplasmy also exists with no apparent functional consequences and with no mitochondrial diseases [31,32,33,34,35,36].

In this study, we analyzed the mitogenome landscape of the UAE population. The UAE is located in Western Asia on the Gulf, south of the Strait of Hormuz at the southeast end of the Arabian Peninsula. It borders Oman (east) and Saudi Arabia (south) and shares sea borders with Qatar (west) and Iran (north). The country is a federation of seven emirates: Abu Dhabi (capital), Dubai, Sharjah, Ajman, Umm Al-Quwain (UAQ), Ras Al-Khaimah (RAK), and Fujairah. Approximately 13% of the population (947,997 in 2010) is Emirati citizens. Al Ain, the site of the current study, is the largest inland city in UAE and is part of the emirate of Abu Dhabi. It is located east of the capital and south of Dubai and has the highest proportion of Emirati nationals (~20%). Citizens of the UAE have diverse ethnicity that includes links to the Arabian Peninsula, Persia, Baluchistan, and East Africa. The society is mainly tribal, and intratribal marriages are fairly practiced. Consequently, founder variants and prevalence of inborn errors of metabolism and genetic disorders are exceptionally high [37,38,39].

We carried out complete whole mitogenome sequencing, assembly, and annotation of 232 female UAE citizens and highlighted the genetic population grouping (haplogrouping). We characterized variants and their effect and test for selective force acting on the UAE mitogenome. We estimated diversity, population structure, and differentiation among cities and regions. In addition, to characterize variants, we estimated heteroplasmy in the population. Finally, we used mtDNA to construct a portrait of the Holocene and late Pleistocene population size of the UAE population.

## 2. Materials and Methods

### 2.1. Ethics Statement, Sample Collection, and DNA Isolation

Al Ain Medical Human Research Ethics Committee approved this study according to the national regulations (#10/09). This study recruited national UAE female students (age range: 18 to 24 years) matriculated at the UAE University. The UAE University is a federal institution and students’ diversity represents all of the seven Emirates. Venous blood samples (5 mL in EDTA-Vacutainers) were collected randomly from 248 consented female students. Total genomic DNA was extracted from blood lymphocytes using the DNeasyBlood and Tissue Kit (Qiagen, Hilden, Germany). DNA quality and concentration were confirmed with NanoDrop and agarose gel, and the samples were stored at −20 °C. We used already published 70 genomes from East Africa in our analysis (accession numbers JN655773–JN655842) for comparative analysis.

### 2.2. Human Mitogenome Enrichment and Sequencing

Mitogenome enrichment is a critical step to reduce nuclear DNA contamination and was accomplished using a specific long-range PCR amplification step directed by two sets of overlapping primers, where each pair of primers flanked 8500 base pairs (bp) [40]. Two PCR amplicons were used for the NGS library preparation. A 200-bp sequencing kit (Ion Xpress Plus Fragment Library Kit; Life Technologies, Carlsbad, CA, USA) was used to generate a short mitogenome fragment library. The samples were loaded onto sequencing chips, and they were sequenced using an Ion Torrent™ Personal Genome Machine™ (PGM) system platform (Life Technologies, Marsiling, Singapore ). Each sequencing chip had the capacity to sequence mitogenomes from 10–20 subjects at a coverage of 250–500X. Primer pairs (F16441: 5′ACTCTCCTCGCTCCGGGCCC3′, R29: 5′TCTATCACCCTATTAACCAC3′) were used to amplify the control region of the mitogenomes. A Genetic Analyzer (model 3500; Applied Biosystems, software version 3.0, Applied Biosystems, Hitachi, Japan ) was used to sequence the PCR amplified mitogenome control region (~120 bp), and gap regions were filled manually.

### 2.3. Data QC, Assembly, and Variant Identification

The quality of the raw Ion Torrent PGM fastq files was checked using FastQC. The Geneious software platform [41] was used for read trimming, reference mapping, and assembly process. The homopolymer quality reduction option of Geneious was used to manage homopolymer runs that are associated with Ion Torrent data. The raw fastq reads with a quality value less than 20 (Q < 20) were trimmed out. Preprocessed reads were mapped to the revised Cambridge Reference Sequence (rCRS) in Geneious version 9.0.4, using the Bowtie2 mapper (bowtie parameter: bowtie2-align-s -I 0 -X 800 -*p* 20 –sensitive -D 15 -R 2 -N 0 -L 22 -i S,1,1.15) [42]. Homopolymer quality reduction was set to 30% to account for such errors in Ion Torrent reads [43]. Geneious bowtie plugin was used for mitogenome assembly from the aligned BAM files. A variant calling file (VCF) for the merged BAMs was generated using GATK [44] using ploidy 1 for haploid genome. Variant annotation of the VCF was carried out using HmtNote database [45]. The annotation was performed using data from hmtVar, which is a recently published database that collects information from several online databases as well as offering in-house pathogenicity predictions. The number of variants (SNPs, Indels, and multiple nucleotide polymorphisms (MNPs)) was counted per genes. Allele frequency was calculated as well as synonymous and nonsynonymous variants from the VCF file. Circos and bar plots were generated to summarize the variants and their distributions [46].

### 2.4. Tree Reconstruction and Haplogroup Prediction

A maximum parsimony tree was generated using coding region sequences from the 232 female mitogenomes (positions 576-16,023) separately, as well as combined with 70 African mitogenomes using MEGA6 [47]. Branch lengths are proportional to the number of mutations. Tree visualization was performed using Figtree v.1.4.0 [48]. Detected variants were manually assessed to ensure they were not assembly artifacts. Identified variations were exported and converted using in-house scripts for haplogroup determination. Haplogroup, based on PhyloTree Build 17, was determined using HaploGrep2 and mtHap [49]. Haplogroups were defined and their relative frequencies for the UAE population are represented as pie chart. The close geographical proximities of neighboring different Emirates can be essentially considered as one region, which allowed us to add their frequencies and place it on a UAE geographic map generated using R map [46].

### 2.5. Population Structure and Differentiation

We calculated pairwise population differentiation Fst with vcftools using haploid option. We also estimated Hs (Heterozygosity with structure) and Ht (Heterozygosity without structure) as well as Gst, G’st, and D statistic using R package adegenet [50]. The same package was used to describe population structure (K = 1 to 12) using discriminant analysis of principal components (DAPC) [51]. We also evaluated population structure using maximum likelihood phylogenetic tree using raxml [52]. We tested if distinct subpopulations (e.g., cities) are mixed together using analysis ADMIXURE [53] with the same number of clusters and the best k value was evaluated using CLUMP [54]. The analysis was performed on the major cities of the UAE: Al Ain (*n* = 86), Abu Dhabi (*n* = 18), Dubai (*n* = 15), Sharjah (*n* = 15), RAK (*n* = 39), UAQ (*n* = 3), and Fujairah (*n* = 43). Furthermore, Fst was also measured based on the UAE’s geographical regions: Southwest (Al Ain and Abu Dhabi, *n* = 104), most-Northeast (Fujairah, *n* = 43), most-Northwest (RAK, *n* = 39), and mid-Northwest (Dubai, Sharjah, Ajman, and UAQ, *n* = 37).

### 2.6. Diversity, Kinship, and Selection

Coding sequences for the thirteen mitochondrial genes were codon-aligned in frame using pal2nal (version 14) for the UAE individuals. The number of polymorphic sites per population (S), nucleotide diversity (π), Watterson theta (θ), and Tajima’s D [55] were calculated using MEGA6. This was conducted for different haplogroups as well as for each city in the UAE. We used NGSrelate (version 2) to estimate relatedness and plotted kinship matrix relationship among the different 232 female individuals. We inferred the strength of selection using d*N*/d*S* metric implemented in HyPhy using the method BUSTED.

### 2.7. Reconstruction of Demographic History

To reconstruct the demographic history in our samples, we used BEAST version 1.8.0 [56]. The program will estimate wide different model parameters, such as genealogical structure, substitution model, and effective population size given a set of genetic sequences. An uncorrelated relaxed clock model was used, which allows the rate to vary across branches in the genealogy. Demographic history was reconstructed using Bayesian skyline model [57]. The complete BEAST input file is available upon request.

### 2.8. Heteroplasmy and Structural Variation Identification

Heteroplasmy identification was carried out using mtDNA-Server [58]. The mtDNA-Server is optimized to analyze the Ion Torrent PGM-aligned reads. Heteroplasmy was plotted as proxy for heterozygosity and a cut-off of > 5% was used in the identification process. Another manual curation, including coverage, being away from indels, and MNPs, as well as the Ts/Tv ratio, was used in the filtering process. Long-range structural variation was run using eKLIPse [59].

## 3. Results

### 3.1. Mitogenome Assembly and Variant Annotation

**Figure 1:**
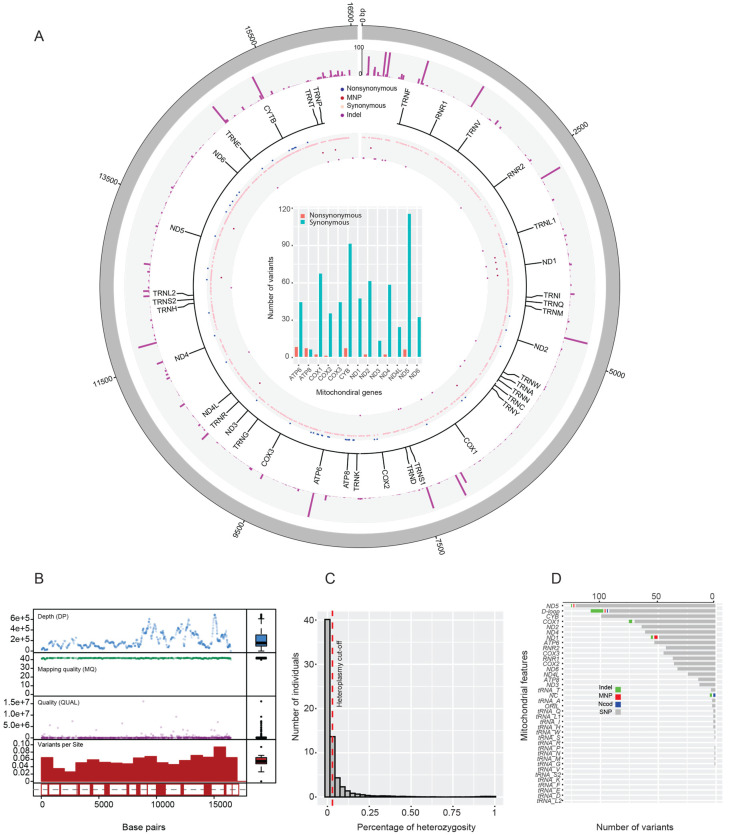
Mitochondrial genomic variation within the UAE population. (**A**) Circos plot depicting the overall mitochondrial genomic variation. From the outer to inner rings: (1) variant allele frequency, (2) variant type, and (3) gene positions. The inner barplot shows the synonymous and nonsynonymous variants for each mitochondrial gene. (**B**) Quality control statistics such as depth of coverage (DP), mapping quality (MQ), and quality of variants (QUAL), as well as the number of variants per site. (**C**) Percentage of heterozygosity as a proxy for heteroplasmy for heterozygote variants depicted on the *x*-axis. The *y*-axis is the number of individuals with heteroplasmic locus. (**D**) The number of variants distribution per mitochondrial gene, RNR, tRNA, and noncoding.

Preprocessed NGS reads were aligned against the reference mitogenome using the Bowtie2 program. We identified 968 SNPs and 30 indels including 11 MNPs (Figure 1A). We found more synonymous than nonsynonymous mutations in our population. This is also the same for all coding genes except for ATP8 gene, where the numbers are the same. Allele frequency found to be skewed toward low-frequency polymorphism (Figure 1A). We found an average read coverage depth of ~400X from the alignments and only high quality variants per site proceeded for analysis (Figure 1B). The level of heteroplasmy was estimated as a proxy for heterozygosity (Figure 1C). From the alignment BAM files, 232 complete mitogenomes were assembled. Annotation of these mitogenomes resulted in 13 protein-coding genes, 2 ribosomal RNA (rRNA) genes, and 22 transfer RNA (tRNA) genes (Figure 1A). The remaining control regions (~120 bp) were separately sequenced and manually filled the mitogenome gap. The number of variants was calculated per genomic features, and we observe more variants in ND5 gene and D-loop (Figure 1D) compared to others. Sequenced mitogenomes (*n* = 232) were deposited in NCBI-Genbank database (accession numbers: MF437054–MF437285), and generated NGS reads were deposited in NCBI-SRA database (SRA ID: PRJNA566159).

### 3.2. Haplogroup Identification

**Figure 2:**
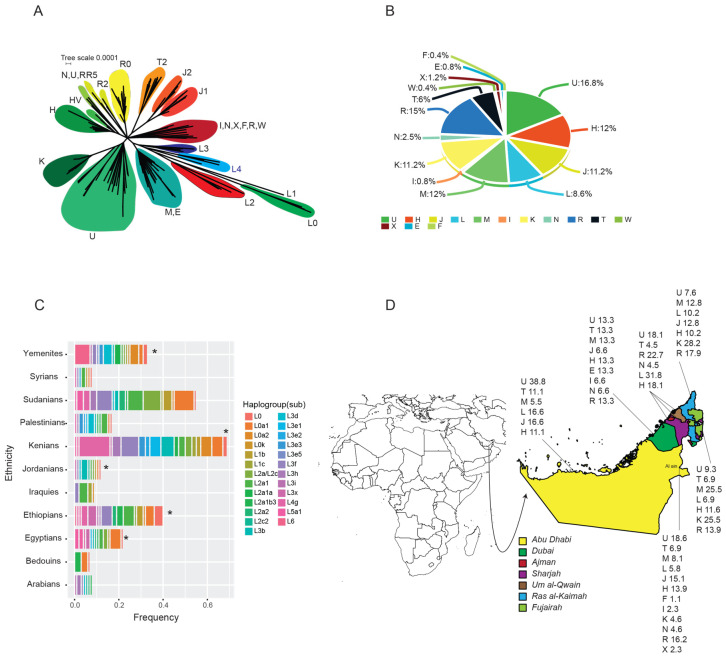
Haplogroup distribution and frequency. (**A**) Network haplogroup tree depicting the ancestral (L) haplogroups and the out of Africa haplogroups. (**B**) Total haplogroup frequencies for the UAE population. (**C**) Stacked bar plots for the frequencies of L3 haplogroup from different Levant and African countries. Stars highlight the presence of L3x1b sub-haplogroups in these countries. (**D**) A map of Africa and the Arabian Peninsula and a zoom on the UAE map with highlights of the different haplogroup frequencies for the different cities of the UAE.

Fifteen haplogroups were identified in the 232 samples (Table 1). A network relationship of different haplogroups is shown in Figure 2A. It presents the ancestral diverse haplogroups with long branch length (L0, L1, L2, L3), indicating more diversity compared to the remaining M and N and other derived haplogroups. A pie chart summarizing the frequencies of different haplogroups is highlighted in Figure 2B. Briefly, haplogroup U was predominant, representing 16.81% (39 samples) of the total studied samples. All of the sub-haplogroups of U (U1, U2, U3, U4, U5, U6, U7, and U9) were identified in the UAE samples. U2 (U2b1, U2b2, U2c1, and U2c1a), U3 (U3a2a1, U3b1, and U3b1a), and U4 (U4c1) constituted 15% of individuals in our population and they are also common haplogroups in India, while North African subclade U6a has a provenance primarily in Morocco. European subclade U2e (U2e1, U2e1b, and U2e3) and rare subclades U9a and U9b represent gene flows from the north (Table S1).

Haplogroup R was the second most common haplogroup, found in 15.08% of samples. Sub-haplogroups of R (R0a2f, R0a2f1b, R0a2h, R2d, R30a1a, R30b1, and R5a2) were identified in 35 samples. Haplogroup M accounted for 12.06% of the total; the generalized African subclade M1a (M1a1, M1a1b1b, and M1a1f) constituted 14% of the total haplogroup. Haplogroup K represented 11.20% of the total population. Haplogroup J was found in 25 samples (10.77%); sub-haplogroups J1b (J1b, J1b1b1, J1b1b3, and J1b2), J2a (J2a2a1a1, J2a2b, J2a2b1, and J2a2c1), and J2b (J2b1 and J2b1f) were detected in this study (Table S1).

Haplogroup L (Sub-Saharan Africa) accounted for 8.62% of the total mitogenome samples (L0, 1.29%; L1, 0.43%; L2, 3.01%; L3, 1.72%; and L4, 2.15%). We also identified one individual with L3x1b sub-haplogroups in the population. Sub-haplogroups L3x1b in L3 is one of the oldest evolutionary steps in the history of out of Africa, and it was previously reported in Kenya, Jordan, Yemen, Ethiopia, and Egypt (Figure 2C).

North Africa haplogroup HV was detected in 4.31% of the total, while Western Asia (the Near East) haplogroup H was detected in 8.18% of the total. Haplogroup T constituted 6.03% of the total, a clade that emanated from the Near East and was common among Iranians. The remaining identified haplogroups (E, F, I, N, W, and X) constituted approximately 7% of the total samples. The frequency of the different haplogroups per city is highlighted on the UAE map (Figure 2D). The frequency distribution of the different sub-haplogroups across the different cities is summarized in Table S1. Briefly, 120 sub-haplogroups were detected, where 58 sub-haplogroups (48.3%) were identified in Al Ain, 26 (21.6%) in Fujairah, 26 (21.6%) in RAK, 13 (10.8%) in Dubai, 12 (10%) in Sharjah, and 15 (12.5%) in Abu Dhabi (Table S1). A Venn diagram of the shared and unique sub-haplogroups across the different cities is summarized in Supplementary Figure S1. 

### 3.3. Population Structure and Differentiation

**Figure 3:**
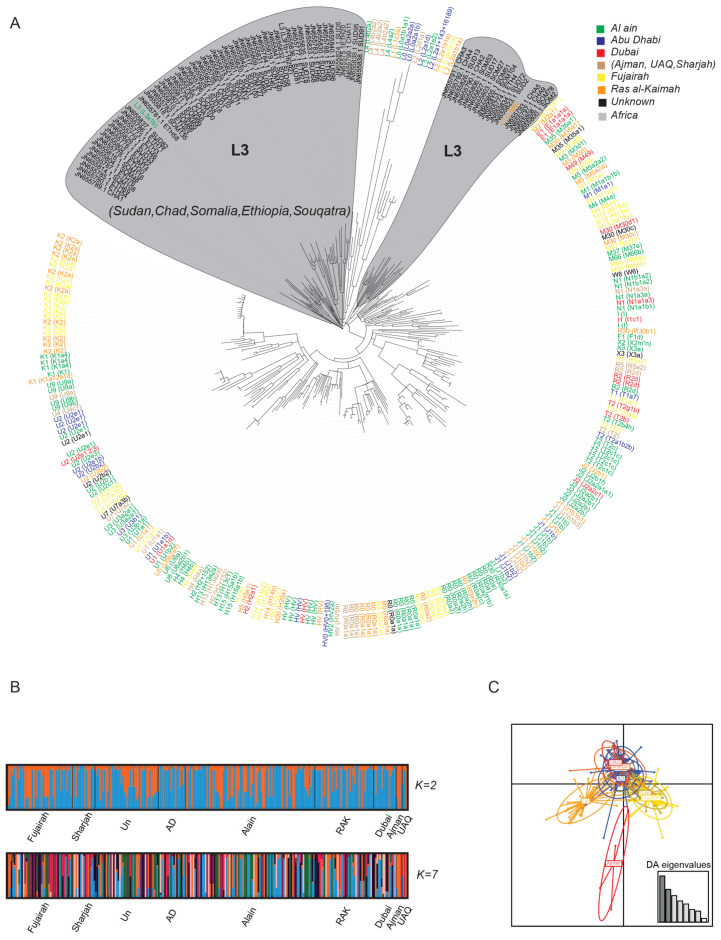
Population structure in UAE population. (**A**) Maximum likelihood phylogenetic tree depicting the network relationship among the different haplogroups (sub-haplogroups), where they are color-coded by city/region. (**B**) Admixture plot for K = 2 and K = 7 ancestral population. (**C**) Discriminant Analysis of Principal Component (DAPC) showing the clustering of the different cities of the UAE population.

The maximum likelihood phylogenetic tree of the different haplogroups colored by cities (Figure 3A) shows there is no clear population structure based on geographical areas. In addition, it is evident, in conjunction with 70 African mitogenomes, that the sub-haplogroup L3x1b clusters with an Ethiopian sample confirming the ancient step out in the region out of African. Furthermore, we observe admixture among geographical regions in the UAE, which is also observed in previous studies [12,60]; Figure 3B depicts admixture results where a clear admixture event at K = 7 number ancestral population can be seen. The lack of population structure is also addressed using a DAPC analysis (Figure 3C), which demonstrate non-clustering of different cities and geographical regions. 

**Figure 4:**
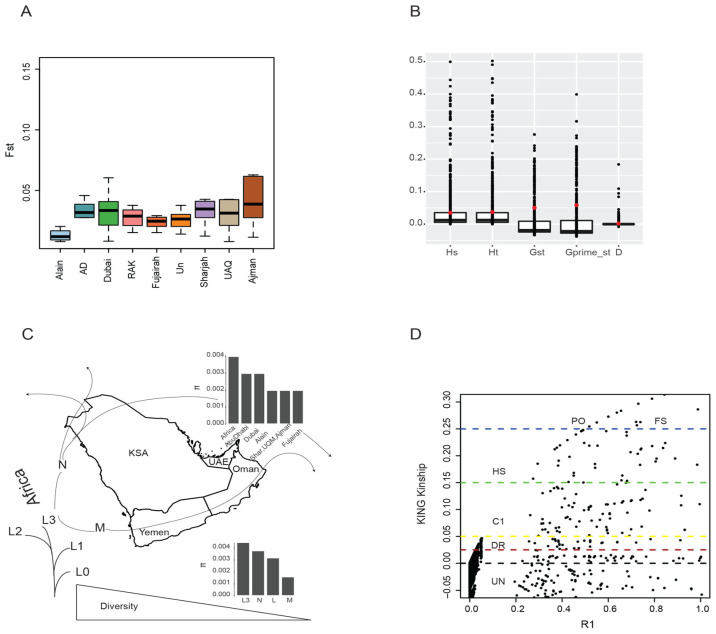
Population differentiation and diversity. (**A**) Population differentiation (Fst) per city of the UAE. (**B**) Population differentiation statistics depicted by Hs, Ht, Gst, G’st, and D. (**C**) A map showing the expected gradient of diversity decrease along the out of Africa passage. Diversity estimates for Africa in comparisons to other UAE cities as well as L haplogroup (and L3) to M and N haplogroups. (**D**) Consanguinity statistics plotted as a KING robust kinship matrix for the different individuals, where the degree of relatedness is shown as UN (unrelated), DS (distantly related), C 1(first cousin), HS (half-sibs), PO (parents-offspring), and FS (full-sibs).

The pairwise population differentiation (Fst) among cities shows no great differentiation with F_st_ varying from 0.009 to 0.07 as it is shown in (Table S2) and the F_st_ boxplot (Figure 4A). We observe a slight differentiation in Alain compared to Abu Dhabi and the other cities. The H_s_, H_t_, G_st_, G’_st_, and D statistics (Tables S3 and S4) (Figure 4B) (*p* < 0.05) confirm that there is no structure and population differentiation among the different cities. 

### 3.4. Diversity, Kinship, and Selection

As it is expected, the pairwise diversity estimate (π) is higher in Africa compared to different cities in the UAE population. It is also higher in Abu Dhabi and Dubai compared to Alain, which is a province of Abu Dhabi. In addition, we observe the L3 haplogroup in Africa, and the UAE population (π = 0.004) has higher diversity compared to M and N haplogroup. It is also higher even when combined the L haplogroup together (Figure 4C). Kinship relationship using KING matrix shows that there is a tapestry of closely related individuals starting from distantly related individuals (DS), first cousins (C1), Half-sibs (HS1), Parent-offspring (PO), and full-sibs (FS) in the population.

Looking at the coding sequences of the 13 different mitochondrial genes, we observe higher Watterson theta (θ) compared to Pi (π), as well as a significant negative Tajima’s D (Table 2). Only ATP8 gene shows the signature of selection. We looked at the strength and nature of selection from HyPhy BUSTED for all the coding sequences, ATP8 show signs of diversifying selection with d_N_/d_S_ ratio > 1.

### 3.5. Demographic History Reconstruction

**Figure 5:**
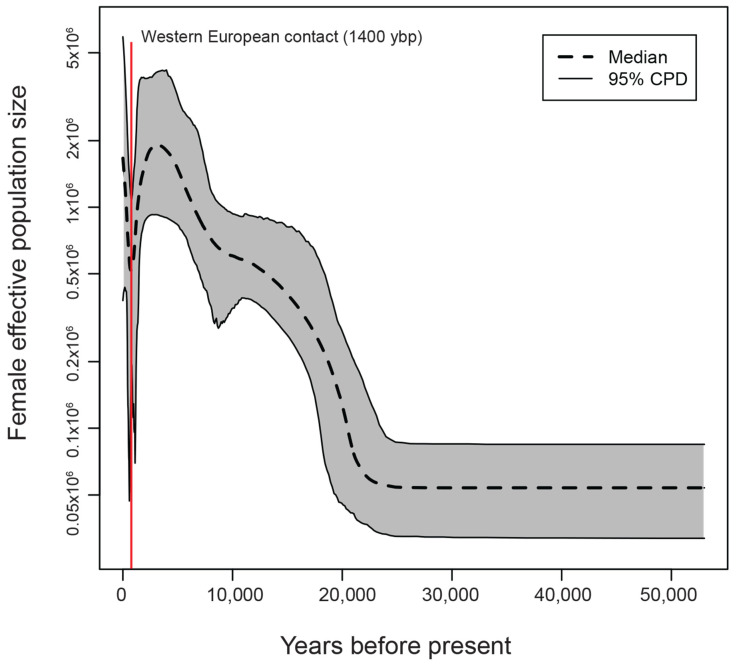
Demographic reconstruction of the UAE mitogenome. Extended Bayesian skyline plot of female effective population size and vertical red line to highlight the bottleneck event that is around 1400 ybp.

BEAST output was used to perform an extended Bayesian skyline plot (EBSP) analysis. We reconstructed the distinct demographic epochs, which highlights a significant and transient contraction in population size some 1400 years before the present (Figure 5). Giving the uncertainty that is associated with the reconstruction method, the plot shows a contraction and reduction for several thousand years and then returned to the level that was before the event. Sd the rate at which lineage coalesces is inversely proportional to population size, our analysis suggests that the bottleneck could affect the different haplogroups disproportionally which is obvious from the branch length distance (Figure 2A).

### 3.6. Heteroplasmy and Structural Variation Identification

Heteroplasmy level estimated using mtDNA server shows that D-loop has 305 followed by ND5 276, which is the highest in the genome. These mutations harbored in these genes hold different known pathogenic diseases, which are reported in the human mitogenome. We documented the number as well as the disease associated according to HmtNote (Table S5). There are three mutations in tRNA: two are annotated according to Mitomap with some tumorigenic risk, while one heteroplasmic mutation in tRNA^His^ gene at position 12172 A->G, with no Mitomap annotation. This mutation is not at higher frequency (1.5%) in the population compared to the others. The RNA fold server was used to predict the change in the secondary structure of the wild type (A) versus the mutant (G). The results highlight a change in the structure in the mutant versus the wild type for only this mutation (Supplementary Figure S2). The eKLIPse results found no significant SV in the 232 different BAMs in the study (Supplementary data zipped file).

## 4. Discussion

The Arabian Peninsula holds the answers to the out-of-Africa migration and the start of modern human continental genetic diversity and structure. Whether a Levantine terrestrial cross between Africa and Southwest Asia or an East African cross of the Red Sea to the south of Arabian Peninsula and moving eastward [3], archeologists and geneticists are still searching for evidence to favor this route or the other that contributed to human evolution. Although the southern route is the favored option [3], many studies confirm admixture events between the Levant and Arabia, which is likely through the Gulf corridor [61,62,63,64].

In our study, the direct descendent (N1, X) of lineage affiliated with L haplogroups suggests an ancient ancestry in the region, which most likely dispersed through the Gulf corridor towards the Levant and Europe 24–55 Ka [65]. Another evidence, in our study, of the Levantine corridor dispersal preference over the horn of Africa is the presence of H, J*, N1b, and T1 haplogroups; other studies, however, confirmed its distribution to be higher in the Levant populations (Iraq, Israeli Druze, Jordan, Palestine, and Syria) compared to Arabian Peninsula groups [11,60,66,67,68,69,70]. In contrast, the M1 haplogroup in our study points to the horn of Africa migration because the frequency of this haplogroup is reported to be high in Ethiopia, low by polymorphic in Yemen and reduced in the Middle East [2,66,68]. Thus, either an Indian or East African origin suggests a favors the horn of Africa route over the Levantine corridor. On the other hand, the distribution of K and HV1 haplogroups of Eurasian origin [67,69] in our study indicate the dispersion by both routes. However, Rowold et al. (2007) showed that the old TMRCA of the star-shaped UAE HV1 network that points to a southern route.

The presence of L1, L2, and L3, in our study, points to sub-Saharan mtDNA lineages. Sub-haplogroup L2a, for example, is associated with the Bantu expansion [71], which is pervasive throughout African and sub-Saharan habitants. However, the presence of the L sub-haplogroup of deep ancestry in the Arabia/Near East reflects its restriction to the horn of African countries (e.g., Ethiopia, Somalia, Sudan, Egypt, etc.). For instance, “ancient” L clades in the Middle East are (L0f2, L0a1d, L0a1c, L1b1a2, L5a1, and L2a1d), and the more prevalent clades are L4, L6, L3i, L3k, L3h, and L3x haplogroups. In contrast, the recently introduced L clades to the Middle East are L0s, L1s, L2s (L2a1a and L2a1b), L3s (L3e, L3b/d’s, and L3f L3f1b), excluding the ancient L clades (L0s and L1s). The former appears to be associated with slave trade in the Middle East. We also observed sub-haplogroups L2a1d and L4 as well as L3x1b, which cluster with Ethiopian individuals ( Figure 2C and Figure 3A), indicating that it is has been in the Arabia/Near East for a long time. The majority of the sub-Saharan genetic contribution in our population is the product of Arab slave trade, which involved the movement of African slaves through an East African trade route 2500 years ago [72].

The mitochondrial genomic landscape of the UAE mitogenomes has 968 variants and the majority is in the D-loop and ND5 gene (Figure 1D). This is expected as the D-loop accumulates more mutation, as it does not encode any specific protein. As for ND5, the accumulation of synonymous variants is not surprising given the high rate of the heteroplasmic allele in ND5 [73] (Table S5). Consanguinity is high in the UAE, which increases the incidence of recessive genetic disorder at the genomic level and might affect the mitochondria due to the genetic interaction between nucleus and mitochondria [74]. Heteroplasmy mutations are involved in cancer and aging, but they are also common in healthy humans, and one allele frequency can change over generations (mother-to-child) because of the bottleneck effect. The bottleneck effect can shift the ratio of alleles in a heteroplasmic mitochondrion causing a generational-dependent disease prevalence [75]. In our study, heteroplasmy is more pronounced in D-loop, ND5, ND4, and CYB, and it is associated with known mapped diseases such as cyclic vomiting syndrome; CPEO/Stroke/CM/breast, renal, and prostate cancer risk/altered brain pH/sCJD (Sporadic Creutzfeldt–Jakob disease), and LHON; PD protective factor/longevity/altered cell pH/metabolic syndrome/breast cancer risk/LS risk/ADHD/cognitive decline; and primary open-angle glaucoma (POAG) (Table S5). In addition, the heteroplasmic mutation in tRNA^His^ reported previously [76] is associated with lung cancer. Further studies are required to elucidate the generation-dependent allelic ratio in heteroplasmy cases. This knowledge is useful especially in genetic counseling and diagnostics among UAE citizens especially in cases involving close relative marriages [77,78].

As it is expected, the mitogenome harbors more synonymous mutations than nonsynonymous mutations (Figure 1A), which suggests a strong purifying selection at purging deleterious mutations to maintain fully functioning mitochondria. This is supported by the allele frequency spectrum that is skewed toward low frequency of polymorphism and the diversity estimate theta (θ) > Pi (π), a negative Tajima’s D and dN/dS < 1 of 12 mitochondrial genes. In contrast, ATP8 shows a signature of diversifying selection, with dN/dS = 1.03 (Table 2) and significant departure from neutrality. One explanation could be that the regional distribution of haplogroups in Eurasia and Africa has been shaped by natural selection on the oxidative phosphorylation pathway in response to change in the climate conditions (e.g., from a cool/warm environment to a hot arid environment) [79].

As expected, higher diversity is observed in Africa (0.004 ± 0.00279) than UAE (0.0021 ± 0.0012) to (0.0033 + 0.00310). The lower diversity is consistent with the paucity of population differentiation and structure as shown in the Fst for the UAE population, which is also consistent with the genomic result from the UAE population that was recently published [80]. The haplogroup diversity, especially the African L (0.0037 ± 0.00302) and non-African M and N mtDNA, shows that L3 has higher diversity (0.004 ± 0.00279), whereas M (Tajima’s D= −1.024, *p* < 0.01) and N (Tajima’s D = −0.908, *p* < 0.01) analyzed separately, show low diversity (0.0016 ± 0.00162; 0.0021 ± 0.0022) and significant deviation from neutrality (Table 3), which is consistent with population expansion out of Africa that distorted the frequency of the mtDNA variants [81,82].

The reconstructed demographic history of the UAE mitogenome sheds light on a bottleneck event around 1400 years ago that coincides with western European contact (Figure 5). The eastern Mediterranean region witnessed Crusader settlements between 11th and 13th centuries that could create immense genetic drift and bottleneck effect in the introduction of western European lineages into the Levant, which will affect a big portion of today’s gene pool [83]. This western European gene background expanded to the eastern Arabian Peninsula, where the influence of the Portuguese was eminent in major parts for the following 150 years.

## 5. Conclusions

This study describes the genomic landscape of the UAE mitochondrial genome and the distribution of haplogroups in different geographic regions in the UAE. The analyzed mitogenomes from 232 female students of UAE University, aged 18–24 years, highlights the high resolution of 15 different haplogroups that share ancestry with Africa, East Asia, and the Near East. Furthermore, it elucidates migration routes to the UAE. The low diversity and population differentiation highlight that the low movement between cities. The Demographic history highlights a bottleneck event that coincides with European contact 1400 ybp. In conclusion, this study also provides a matrilineal history of the UAE and will serve as an asset for genetic counseling, forensic science, and anthropology among other fields.

## Figures and Tables

**Table 1:** UAE population haplogroup distribution.

				NE **	NW **	Mid-NW **				SW **	
Group ^a^	F ^b^	F ^b^%	Unknown	Fujairah	RAK *	UAQ *	Dubai	Sharjah	Ajman	Al Ain	Abu Dhabi
E	2	0.86	0	0	0	0	2	0	0	0	0
F	1	0.43	0	0	0	0	0	0	0	1	0
H	19	8.18	0	5	3	0	1	3	0	7	0
HV	10	4.31	0	0	1	0	1	1	0	5	2
I	3	1.29	0	0	0	0	1	0	0	2	0
J	25	10.77	0	0	5	0	1	2	1	13	3
K	26	11.20	0	11	11	0	0	0	0	4	0
L	20	8.62	1	3	4	0	0	4	0	5	3
M	28	12.06	2	11	5	0	2	0	0	7	1
N	6	2.58	0	0	0	0	1	0	1	4	0
R	35	15.08	1	6	7	2	2	2	1	14	0
T	14	6.03	0	3	0	0	2	1	0	6	2
U	39	16.81	3	4	3	1	2	2	1	16	7
W	1	0.43	1	0	0	0	0	0	0	0	0
X	3	1.29	1	0	0	0	0	0	0	2	0
Total	232	100	9	43	39	3	15	15	4	86	18

** Region-wise classification: NE—Northeast region (*n* = 43), NW—Northwest region (*n* = 39); Mid-NW—mid-Northwest region (*n* = 37); SW—Southwest region (*n* = 104); * RAK—Ras al Khaimah; UAQ—Umm al-Quwain; *a* Haplogroup; *b* Frequency.

**Table 2:** Population genetic parameters and the ratio of nonsynonymous to synonymous substitution rate for each mitochondrial gene.

Genes	M	S	Ps	θ	π	D	d_N_/d_S_
ATP6	232	45	0.067265	0.011244	0.002034	−2.391682	0.29
ATP8	232	17	0.087179	0.014573	0.001415	−2.325058	1.03
COX1	232	75	0.050302	0.008409	0.001305	−2.556932	0.10
COX2	232	34	0.050595	0.008458	0.001166	−2.449534	0.15
COX3	232	40	0.053763	0.008987	0.001757	−2.323988	0.12
CYTB	232	95	0.085818	0.014346	0.002837	−2.457115	0.19
ND1	232	56	0.06041	0.010098	0.002071	−2.361573	0.12
ND2	232	63	0.0625	0.010448	0.00161	−2.533238	0.12
ND3	232	16	0.048048	0.008032	0.003149	−1.548463	0.15
ND4	232	74	0.055306	0.009245	0.002533	−2.195525	0.04
ND4L	232	31	0.050542	0.016855	0.001867	−2.440526	0.06
ND5	232	121	0.068789	0.011499	0.002293	−2.476299	0.14
ND6	232	31	0.060429	0.010102	0.001778	−2.31697	0.08

m: number of sequences. S: Number of segregating sites. Ps: S/*n* (total number of sites). θ: Watterson theta. π: nucleotide diversity. D: Tajima’s D. d_N_/d_S_: ratio of nonsynonymous/synonymous substitution rate.

**Table 3:** Population genetic parameters per cities and haplogroups.

City/Haplogroup	π	D
Abu Dhabi	0.0033 ± 0.00310	−0.9003 ± 0.67193
Al Ain	0.0022 ± 0.00241	−1.0783 ± 0.59466
Dubai, Ajman, UQA	0.0029 ± 0.00264	−0.8337 ± 0.54551
Fujairah	0.0023 ± 0.00258	−1.0999 ± 0.74124
RAK	0.0021 ± 0.00237	−0.8034 ± 0.62341
Sharjah	0.0024 ± 0.00211	−0.9012 ± 0.53421
UAQ	0.0021 ± 0.0012	−0.5231 ± 0.32310
Africa (L3)	0.004 ± 0.00279	−1.7485 ± 0.38859
L	0.0037 ± 0.00302	−0.8079 ± 0.70818
M	0.0016 ± 0.00162	−1.0248 ± 0.56632
N	0.0021 ± 0.00221	−0.9080 ± 0.85152

π: nucleotide diversity. D: Tajima’s D.

## Supplementary Materials

The following are available online at https://www.mdpi.com/2073-4425/11/8/876/s1, Table S1. The haplogroups/sub-haplogroups distribution per city in the UAE population. Table S2. Pairwise Fst among the different cities of the UAE population. Table S3. Summary statistics of Hs and Ht for different cities of the UAE population. Table S4. Summary of the statistic of Gst, Htmax, Gstmax, and G’st. Table S5. Summary of filtered heteroplasmy counts and disease associated annotation. Figure S1. Venn diagram of the different sub-haplogroups among cities of the UAE. Figure S2. Prediction of the MFE structure tRNAHis gene in wild type and mutant (A12172G). Supplementary data zipped. Zipped plots from eKLPse software for the 232 individuals long structural variants.
