# Supplementary material for: Genomic Landscape of the Mitochondrial Genome in the United Arab Emirates Native Population
Source: Genes (Basel). 2020 Aug 1;11(8):876. doi: 10.3390/genes11080876 (PMC7464197; doi:10.3390/genes11080876)
Supplement: Supplementary file 1 [file genes-11-00876-s001.zip › sup_files/Table_S3.html]

| CHROM | POS | Hs\_Alain | Hs\_AD | Hs\_Dubai | Hs\_RAK | Hs\_Fujairah | Hs\_Un | Hs\_Sharjah | Hs\_UAQ | Hs\_Ajman | Ht | n\_Alain | n\_AD | n\_Dubai | n\_RAK | n\_Fujairah | n\_Un | n\_Sharjah | n\_UAQ | n\_Ajman | Gst | Htmax | Gstmax | Gprimest |
| --- | --- | --- | --- | --- | --- | --- | --- | --- | --- | --- | --- | --- | --- | --- | --- | --- | --- | --- | --- | --- | --- | --- | --- | --- |
| NC\_012920 | 36 | 0.0000000 | 0.0000000 | 0.0000000 | 0.0000000 | 0.0464853 | 0.0000000 | 0.0000000 | 0.0000000 | 0.000 | 0.0076627 | 85 | 18 | 15 | 38 | 42 | 40 | 15 | 3 | 4 | 0.0200404 | 0.8113905 | 0.9907453 | 0.0202276 |
| NC\_012920 | 58 | 0.0454300 | 0.0000000 | 0.1244444 | 0.1883657 | 0.0000000 | 0.0487500 | 0.2311111 | 0.4444444 | 0.000 | 0.0804440 | 86 | 18 | 15 | 38 | 43 | 40 | 15 | 3 | 4 | 0.0661784 | 0.8205524 | 0.9084515 | 0.0728475 |
| NC\_012920 | 64 | 0.1687399 | 0.0000000 | 0.1244444 | 0.2659280 | 0.1687399 | 0.0487500 | 0.2311111 | 0.4444444 | 0.000 | 0.1601597 | 86 | 18 | 15 | 38 | 43 | 40 | 15 | 3 | 4 | 0.0350905 | 0.8400152 | 0.8160276 | 0.0430016 |
| NC\_012920 | 66 | 0.0673337 | 0.0000000 | 0.0000000 | 0.0000000 | 0.0000000 | 0.0000000 | 0.0000000 | 0.0000000 | 0.000 | 0.0226385 | 86 | 18 | 15 | 38 | 43 | 40 | 15 | 3 | 4 | 0.0237048 | 0.8165899 | 0.9729339 | 0.0243642 |
| NC\_012920 | 73 | 0.4218496 | 0.2777778 | 0.3200000 | 0.3324100 | 0.3028664 | 0.2550000 | 0.4444444 | 0.4444444 | 0.000 | 0.3531263 | 86 | 18 | 15 | 38 | 43 | 40 | 15 | 3 | 4 | 0.0279421 | 0.8797564 | 0.6098248 | 0.0458199 |
| NC\_012920 | 143 | 0.0229854 | 0.1049383 | 0.0000000 | 0.0499671 | 0.0886966 | 0.0000000 | 0.0000000 | 0.0000000 | 0.000 | 0.0372999 | 86 | 18 | 15 | 39 | 43 | 40 | 15 | 3 | 4 | 0.0185100 | 0.8160881 | 0.9551402 | 0.0193794 |
| NC\_012920 | 146 | 0.2879935 | 0.2777778 | 0.1244444 | 0.4970414 | 0.3807463 | 0.2550000 | 0.4977778 | 0.4444444 | 0.000 | 0.3759488 | 86 | 18 | 15 | 39 | 43 | 40 | 15 | 3 | 4 | 0.1262581 | 0.8708525 | 0.6228039 | 0.2027253 |
| NC\_012920 | 150 | 0.2055165 | 0.1049383 | 0.3200000 | 0.0973044 | 0.0886966 | 0.0950000 | 0.1244444 | 0.0000000 | 0.000 | 0.1469444 | 86 | 18 | 15 | 39 | 43 | 40 | 15 | 3 | 4 | 0.0260720 | 0.8402897 | 0.8296858 | 0.0314239 |
| NC\_012920 | 151 | 0.0673337 | 0.1049383 | 0.1244444 | 0.0973044 | 0.0886966 | 0.1387500 | 0.0000000 | 0.0000000 | 0.375 | 0.0939727 | 86 | 18 | 15 | 39 | 43 | 40 | 15 | 3 | 4 | 0.0206253 | 0.8255721 | 0.8885203 | 0.0232131 |
| NC\_012920 | 152 | 0.4307734 | 0.5000000 | 0.3911111 | 0.4733728 | 0.4780963 | 0.4800000 | 0.2311111 | 0.4444444 | 0.375 | 0.4614784 | 86 | 18 | 15 | 39 | 43 | 40 | 15 | 3 | 4 | 0.0406691 | 0.8945337 | 0.5050936 | 0.0805180 |
| NC\_012920 | 153 | 0.0229854 | 0.0000000 | 0.0000000 | 0.0000000 | 0.0000000 | 0.0487500 | 0.1244444 | 0.0000000 | 0.000 | 0.0225535 | 86 | 18 | 15 | 39 | 43 | 40 | 15 | 3 | 4 | 0.0232903 | 0.8136593 | 0.9729270 | 0.0239384 |
| NC\_012920 | 195 | 0.3083900 | 0.4012346 | 0.2400000 | 0.3353057 | 0.4002163 | 0.0973044 | 0.3200000 | 0.0000000 | 0.000 | 0.3012722 | 84 | 18 | 15 | 39 | 43 | 39 | 15 | 3 | 4 | 0.0347589 | 0.8681065 | 0.6650177 | 0.0522676 |
| NC\_012920 | 198 | 0.0464853 | 0.0000000 | 0.0000000 | 0.0499671 | 0.2055165 | 0.0000000 | 0.0000000 | 0.0000000 | 0.000 | 0.0596450 | 84 | 18 | 15 | 39 | 43 | 39 | 15 | 3 | 4 | 0.0526837 | 0.8230473 | 0.9313495 | 0.0565671 |
| NC\_012920 | 199 | 0.0907029 | 0.0000000 | 0.1244444 | 0.0499671 | 0.0000000 | 0.0973044 | 0.0000000 | 0.0000000 | 0.000 | 0.0596450 | 84 | 18 | 15 | 39 | 43 | 39 | 15 | 3 | 4 | 0.0179516 | 0.8246450 | 0.9289703 | 0.0193242 |
| NC\_012920 | 200 | 0.0688776 | 0.0000000 | 0.0000000 | 0.0000000 | 0.0454300 | 0.0499671 | 0.1244444 | 0.0000000 | 0.000 | 0.0450888 | 84 | 18 | 15 | 39 | 43 | 39 | 15 | 3 | 4 | 0.0143725 | 0.8214201 | 0.9458977 | 0.0151945 |
| NC\_012920 | 204 | 0.1119615 | 0.0000000 | 0.2311111 | 0.0499671 | 0.0454300 | 0.1420118 | 0.0000000 | 0.0000000 | 0.000 | 0.0880473 | 84 | 18 | 15 | 39 | 43 | 39 | 15 | 3 | 4 | 0.0253453 | 0.8294675 | 0.8965411 | 0.0282700 |
| NC\_012920 | 217 | 0.0688776 | 0.2777778 | 0.1244444 | 0.0000000 | 0.0454300 | 0.0487500 | 0.1244444 | 0.0000000 | 0.000 | 0.0736924 | 84 | 18 | 15 | 39 | 43 | 40 | 15 | 3 | 4 | 0.0421749 | 0.8233878 | 0.9142756 | 0.0461293 |
| NC\_012920 | 235 | 0.0886966 | 0.1049383 | 0.1244444 | 0.0000000 | 0.0000000 | 0.0950000 | 0.0000000 | 0.0000000 | 0.000 | 0.0589860 | 86 | 18 | 15 | 39 | 43 | 40 | 15 | 3 | 4 | 0.0212621 | 0.8222470 | 0.9297878 | 0.0228677 |
| NC\_012920 | 263 | 0.0000000 | 0.0000000 | 0.0000000 | 0.0000000 | 0.0000000 | 0.0000000 | 0.0000000 | 0.0000000 | 0.000 | 0.0000000 | 86 | 18 | 15 | 38 | 43 | 39 | 15 | 3 | 4 | NaN | 0.8090310 | 1.0000000 | NaN |
| NC\_012920 | 295 | 0.2566252 | 0.2777778 | 0.1244444 | 0.2285319 | 0.0000000 | 0.0499671 | 0.2311111 | 0.0000000 | 0.375 | 0.1793867 | 86 | 18 | 15 | 38 | 43 | 39 | 15 | 3 | 4 | 0.0487804 | 0.8454368 | 0.7981681 | 0.0611154 |
| NC\_012920 | 318 | 0.0000000 | 0.0000000 | 0.0000000 | 0.0000000 | 0.0000000 | 0.0000000 | 0.1244444 | 0.0000000 | 0.000 | 0.0076627 | 86 | 18 | 14 | 38 | 43 | 39 | 15 | 3 | 4 | 0.0630631 | 0.8084024 | 0.9911189 | 0.0636281 |
| NC\_012920 | 326 | 0.0229854 | 0.0000000 | 0.0000000 | 0.0000000 | 0.0000000 | 0.0000000 | 0.0000000 | 0.0000000 | 0.000 | 0.0076627 | 86 | 18 | 14 | 38 | 43 | 39 | 15 | 3 | 4 | 0.0078118 | 0.8105030 | 0.9906196 | 0.0078858 |
| NC\_012920 | 340 | 0.0454300 | 0.2777778 | 0.0000000 | 0.0000000 | 0.0454300 | 0.0499671 | 0.0000000 | 0.0000000 | 0.000 | 0.0523964 | 86 | 18 | 14 | 38 | 43 | 39 | 15 | 3 | 4 | 0.0597438 | 0.8166568 | 0.9396734 | 0.0635793 |
| NC\_012920 | 373 | 0.0229854 | 0.0000000 | 0.0000000 | 0.0000000 | 0.0454300 | 0.0000000 | 0.0000000 | 0.0000000 | 0.000 | 0.0152082 | 86 | 17 | 15 | 39 | 43 | 39 | 15 | 3 | 4 | 0.0098545 | 0.8121431 | 0.9814585 | 0.0100407 |
| NC\_012920 | 447 | 0.0000000 | 0.0000000 | 0.0000000 | 0.0499671 | 0.0000000 | 0.0000000 | 0.0000000 | 0.0000000 | 0.000 | 0.0076335 | 86 | 18 | 15 | 39 | 43 | 38 | 15 | 3 | 4 | 0.0218935 | 0.8101467 | 0.9907839 | 0.0220971 |
| NC\_012920 | 462 | 0.1495403 | 0.2777778 | 0.0000000 | 0.1840894 | 0.0000000 | 0.0973044 | 0.2311111 | 0.0000000 | 0.375 | 0.1345201 | 86 | 18 | 15 | 39 | 43 | 39 | 15 | 3 | 4 | 0.0409367 | 0.8338675 | 0.8452832 | 0.0484296 |
| NC\_012920 | 480 | 0.0000000 | 0.0000000 | 0.0000000 | 0.0000000 | 0.0454300 | 0.0499671 | 0.0000000 | 0.0000000 | 0.000 | 0.0151506 | 86 | 18 | 15 | 39 | 43 | 39 | 15 | 3 | 4 | 0.0169442 | 0.8116951 | 0.9816508 | 0.0172609 |
| NC\_012920 | 482 | 0.0454300 | 0.0000000 | 0.0000000 | 0.0973044 | 0.0454300 | 0.0499671 | 0.0000000 | 0.0000000 | 0.000 | 0.0447526 | 86 | 18 | 15 | 39 | 43 | 39 | 15 | 3 | 4 | 0.0103313 | 0.8187460 | 0.9459047 | 0.0109221 |
| NC\_012920 | 489 | 0.3309897 | 0.3456790 | 0.3911111 | 0.3813281 | 0.4023797 | 0.2945431 | 0.2311111 | 0.0000000 | 0.375 | 0.3447352 | 86 | 18 | 15 | 39 | 43 | 39 | 15 | 3 | 4 | 0.0126066 | 0.8745994 | 0.6108056 | 0.0206394 |
| NC\_012920 | 497 | 0.0000000 | 0.0000000 | 0.0000000 | 0.0499671 | 0.0000000 | 0.0000000 | 0.0000000 | 0.0000000 | 0.000 | 0.0076045 | 86 | 18 | 15 | 39 | 43 | 39 | 15 | 3 | 4 | 0.0219078 | 0.8104714 | 0.9908228 | 0.0221108 |
| NC\_012920 | 499 | 0.1095187 | 0.1975309 | 0.1244444 | 0.0000000 | 0.0454300 | 0.0499671 | 0.1244444 | 0.4444444 | 0.000 | 0.0874075 | 86 | 18 | 15 | 39 | 43 | 39 | 15 | 3 | 4 | 0.0418209 | 0.8253016 | 0.8985195 | 0.0465442 |
| NC\_012920 | 508 | 0.0673337 | 0.1975309 | 0.1244444 | 0.0000000 | 0.0454300 | 0.0499671 | 0.0000000 | 0.0000000 | 0.000 | 0.0592040 | 86 | 18 | 15 | 39 | 43 | 39 | 15 | 3 | 4 | 0.0255504 | 0.8202902 | 0.9296696 | 0.0274833 |
| NC\_012920 | 517 | 0.0000000 | 0.0000000 | 0.0000000 | 0.0000000 | 0.0454300 | 0.0000000 | 0.0000000 | 0.0000000 | 0.000 | 0.0075471 | 86 | 18 | 15 | 39 | 43 | 41 | 15 | 3 | 4 | 0.0195420 | 0.8111513 | 0.9908777 | 0.0197219 |
| NC\_012920 | 522 | 0.0229854 | 0.0000000 | 0.0000000 | 0.0000000 | 0.0000000 | 0.0000000 | 0.0000000 | 0.0000000 | 0.000 | 0.0075471 | 86 | 18 | 15 | 39 | 43 | 41 | 15 | 3 | 4 | 0.0078698 | 0.8123852 | 0.9907831 | 0.0079430 |
| NC\_012920 | 644 | 0.0000000 | 0.0000000 | 0.0000000 | 0.0000000 | 0.0000000 | 0.0475907 | 0.0000000 | 0.0000000 | 0.000 | 0.0075471 | 86 | 18 | 15 | 39 | 43 | 41 | 15 | 3 | 4 | 0.0206807 | 0.8110939 | 0.9908876 | 0.0208709 |
| NC\_012920 | 669 | 0.0000000 | 0.0000000 | 0.1244444 | 0.0000000 | 0.0000000 | 0.0000000 | 0.0000000 | 0.0000000 | 0.000 | 0.0075471 | 86 | 18 | 15 | 39 | 43 | 41 | 15 | 3 | 4 | 0.0631179 | 0.8103478 | 0.9912745 | 0.0636735 |
| NC\_012920 | 709 | 0.2401298 | 0.4012346 | 0.2311111 | 0.4602235 | 0.4023797 | 0.2097506 | 0.3911111 | 0.0000000 | 0.000 | 0.3244998 | 86 | 18 | 15 | 39 | 43 | 42 | 15 | 3 | 4 | 0.0549486 | 0.8651620 | 0.6455357 | 0.0851210 |
| NC\_012920 | 710 | 0.0229854 | 0.0000000 | 0.0000000 | 0.0000000 | 0.0000000 | 0.0000000 | 0.0000000 | 0.0000000 | 0.000 | 0.0075187 | 86 | 18 | 15 | 39 | 43 | 42 | 15 | 3 | 4 | 0.0078841 | 0.8126166 | 0.9908205 | 0.0079571 |
| NC\_012920 | 739 | 0.0229854 | 0.0000000 | 0.0000000 | 0.0000000 | 0.0000000 | 0.0000000 | 0.0000000 | 0.0000000 | 0.000 | 0.0075187 | 86 | 18 | 15 | 39 | 43 | 42 | 15 | 3 | 4 | 0.0078841 | 0.8126166 | 0.9908205 | 0.0079571 |
| NC\_012920 | 742 | 0.0229854 | 0.0000000 | 0.0000000 | 0.0000000 | 0.0000000 | 0.0000000 | 0.0000000 | 0.0000000 | 0.000 | 0.0075187 | 86 | 18 | 15 | 39 | 43 | 42 | 15 | 3 | 4 | 0.0078841 | 0.8126166 | 0.9908205 | 0.0079571 |
| NC\_012920 | 750 | 0.0229854 | 0.0000000 | 0.0000000 | 0.0000000 | 0.0000000 | 0.0000000 | 0.0000000 | 0.0000000 | 0.000 | 0.0075187 | 86 | 18 | 15 | 39 | 43 | 42 | 15 | 3 | 4 | 0.0078841 | 0.8126166 | 0.9908205 | 0.0079571 |
| NC\_012920 | 769 | 0.1495403 | 0.1975309 | 0.0000000 | 0.1840894 | 0.0886966 | 0.1356336 | 0.3911111 | 0.0000000 | 0.000 | 0.1527778 | 86 | 18 | 15 | 39 | 43 | 41 | 15 | 3 | 4 | 0.0370966 | 0.8376377 | 0.8243749 | 0.0449997 |
| NC\_012920 | 789 | 0.0000000 | 0.0000000 | 0.0000000 | 0.0000000 | 0.0454300 | 0.0000000 | 0.0000000 | 0.0000000 | 0.000 | 0.0075471 | 86 | 18 | 15 | 39 | 43 | 41 | 15 | 3 | 4 | 0.0195420 | 0.8111513 | 0.9908777 | 0.0197219 |
| NC\_012920 | 813 | 0.0229854 | 0.1049383 | 0.0000000 | 0.0000000 | 0.0886966 | 0.0000000 | 0.0000000 | 0.0000000 | 0.000 | 0.0298439 | 86 | 18 | 15 | 39 | 43 | 41 | 15 | 3 | 4 | 0.0252832 | 0.8152261 | 0.9643175 | 0.0262188 |
| NC\_012920 | 825 | 0.0454300 | 0.0000000 | 0.0000000 | 0.0499671 | 0.0454300 | 0.0928019 | 0.0000000 | 0.0000000 | 0.000 | 0.0444215 | 86 | 18 | 15 | 39 | 43 | 41 | 15 | 3 | 4 | 0.0096537 | 0.8193010 | 0.9463046 | 0.0102015 |
| NC\_012920 | 827 | 0.0886966 | 0.0000000 | 0.1244444 | 0.1840894 | 0.0454300 | 0.0475907 | 0.2311111 | 0.4444444 | 0.000 | 0.1004362 | 86 | 18 | 15 | 39 | 43 | 41 | 15 | 3 | 4 | 0.0428579 | 0.8269341 | 0.8837493 | 0.0484955 |
| NC\_012920 | 869 | 0.0000000 | 0.0000000 | 0.0000000 | 0.0499671 | 0.0000000 | 0.0000000 | 0.0000000 | 0.0000000 | 0.000 | 0.0075756 | 86 | 18 | 15 | 39 | 43 | 40 | 15 | 3 | 4 | 0.0219221 | 0.8107678 | 0.9908610 | 0.0221243 |
| NC\_012920 | 870 | 0.0000000 | 0.0000000 | 0.0000000 | 0.0000000 | 0.0000000 | 0.0000000 | 0.1244444 | 0.0000000 | 0.000 | 0.0075756 | 86 | 18 | 15 | 39 | 43 | 40 | 15 | 3 | 4 | 0.0631043 | 0.8100739 | 0.9912383 | 0.0636621 |
| NC\_012920 | 906 | 0.0000000 | 0.1049383 | 0.0000000 | 0.0499671 | 0.0000000 | 0.0475907 | 0.0000000 | 0.0000000 | 0.000 | 0.0224690 | 86 | 18 | 15 | 39 | 43 | 41 | 15 | 3 | 4 | 0.0241059 | 0.8126722 | 0.9730182 | 0.0247744 |
| NC\_012920 | 921 | 0.0000000 | 0.0000000 | 0.0000000 | 0.0499671 | 0.0454300 | 0.0487500 | 0.0000000 | 0.0000000 | 0.000 | 0.0225535 | 86 | 18 | 15 | 39 | 43 | 40 | 15 | 3 | 4 | 0.0133780 | 0.8131099 | 0.9726338 | 0.0137544 |
| NC\_012920 | 930 | 0.0454300 | 0.0000000 | 0.1244444 | 0.0000000 | 0.0000000 | 0.0000000 | 0.0000000 | 0.0000000 | 0.000 | 0.0225535 | 86 | 18 | 15 | 39 | 43 | 40 | 15 | 3 | 4 | 0.0266229 | 0.8149315 | 0.9730615 | 0.0273600 |
| NC\_012920 | 951 | 0.0000000 | 0.0000000 | 0.1244444 | 0.0000000 | 0.0000000 | 0.0000000 | 0.0000000 | 0.0000000 | 0.000 | 0.0075756 | 86 | 18 | 15 | 39 | 43 | 40 | 15 | 3 | 4 | 0.0631043 | 0.8100739 | 0.9912383 | 0.0636621 |
| NC\_012920 | 980 | 0.0000000 | 0.0000000 | 0.0000000 | 0.0000000 | 0.0886966 | 0.0499671 | 0.0000000 | 0.0000000 | 0.375 | 0.0300682 | 86 | 18 | 15 | 39 | 43 | 39 | 15 | 3 | 4 | 0.0780911 | 0.8129480 | 0.9659017 | 0.0808479 |
| NC\_012920 | 988 | 0.0000000 | 0.1049383 | 0.0000000 | 0.0000000 | 0.0000000 | 0.0000000 | 0.0000000 | 0.0000000 | 0.000 | 0.0076045 | 86 | 18 | 15 | 39 | 43 | 39 | 15 | 3 | 4 | 0.0519370 | 0.8098596 | 0.9910978 | 0.0524035 |
| NC\_012920 | 1008 | 0.0229854 | 0.0000000 | 0.0000000 | 0.0000000 | 0.0000000 | 0.0000000 | 0.1244444 | 0.0000000 | 0.000 | 0.0151506 | 86 | 18 | 15 | 39 | 43 | 39 | 15 | 3 | 4 | 0.0317561 | 0.8122487 | 0.9819396 | 0.0323402 |
| NC\_012920 | 1018 | 0.0886966 | 0.1975309 | 0.0000000 | 0.1420118 | 0.0886966 | 0.0950000 | 0.3911111 | 0.0000000 | 0.000 | 0.1209212 | 86 | 18 | 15 | 39 | 43 | 40 | 15 | 3 | 4 | 0.0503026 | 0.8290419 | 0.8614804 | 0.0583909 |
| NC\_012920 | 1048 | 0.0673337 | 0.0000000 | 0.0000000 | 0.0000000 | 0.0454300 | 0.0000000 | 0.0000000 | 0.0000000 | 0.000 | 0.0298439 | 86 | 18 | 15 | 39 | 43 | 41 | 15 | 3 | 4 | 0.0170841 | 0.8182966 | 0.9641523 | 0.0177193 |
| NC\_012920 | 1095 | 0.0000000 | 0.0000000 | 0.0000000 | 0.0000000 | 0.0454300 | 0.0000000 | 0.0000000 | 0.0000000 | 0.000 | 0.0075471 | 86 | 18 | 15 | 39 | 43 | 41 | 15 | 3 | 4 | 0.0195420 | 0.8111513 | 0.9908777 | 0.0197219 |
| NC\_012920 | 1120 | 0.0229854 | 0.0000000 | 0.0000000 | 0.0000000 | 0.0000000 | 0.0475907 | 0.0000000 | 0.0000000 | 0.000 | 0.0150367 | 86 | 18 | 15 | 39 | 43 | 41 | 15 | 3 | 4 | 0.0105130 | 0.8135331 | 0.9817111 | 0.0107088 |
| NC\_012920 | 1189 | 0.1095187 | 0.0000000 | 0.0000000 | 0.0499671 | 0.0000000 | 0.0487500 | 0.0000000 | 0.0000000 | 0.000 | 0.0518151 | 86 | 18 | 15 | 39 | 43 | 40 | 15 | 3 | 4 | 0.0227523 | 0.8236060 | 0.9385189 | 0.0242427 |
| NC\_012920 | 1211 | 0.0000000 | 0.0000000 | 0.0000000 | 0.0000000 | 0.0000000 | 0.0000000 | 0.0000000 | 0.0000000 | 0.375 | 0.0075471 | 86 | 18 | 15 | 39 | 43 | 41 | 15 | 3 | 4 | 0.2471483 | 0.8100321 | 0.9929857 | 0.2488941 |
| NC\_012920 | 1243 | 0.0000000 | 0.0000000 | 0.0000000 | 0.0000000 | 0.0000000 | 0.0928019 | 0.0000000 | 0.0000000 | 0.000 | 0.0150367 | 86 | 18 | 15 | 39 | 43 | 41 | 15 | 3 | 4 | 0.0415193 | 0.8121843 | 0.9822547 | 0.0422694 |
| NC\_012920 | 1391 | 0.0000000 | 0.1049383 | 0.0000000 | 0.0000000 | 0.0000000 | 0.0000000 | 0.0000000 | 0.0000000 | 0.000 | 0.0076045 | 86 | 18 | 15 | 39 | 43 | 39 | 15 | 3 | 4 | 0.0519370 | 0.8098596 | 0.9910978 | 0.0524035 |
| NC\_012920 | 1393 | 0.0454300 | 0.0000000 | 0.0000000 | 0.0000000 | 0.0000000 | 0.0000000 | 0.0000000 | 0.0000000 | 0.000 | 0.0151506 | 86 | 18 | 15 | 39 | 43 | 39 | 15 | 3 | 4 | 0.0157424 | 0.8142591 | 0.9816863 | 0.0160361 |
| NC\_012920 | 1413 | 0.0229854 | 0.0000000 | 0.0000000 | 0.0000000 | 0.0000000 | 0.0000000 | 0.3200000 | 0.0000000 | 0.000 | 0.0300682 | 86 | 18 | 15 | 39 | 43 | 39 | 15 | 3 | 4 | 0.1397738 | 0.8128897 | 0.9681809 | 0.1443674 |
| NC\_012920 | 1420 | 0.0000000 | 0.0000000 | 0.0000000 | 0.0000000 | 0.0000000 | 0.0487500 | 0.0000000 | 0.0000000 | 0.000 | 0.0075756 | 86 | 18 | 15 | 39 | 43 | 40 | 15 | 3 | 4 | 0.0212786 | 0.8107967 | 0.9908554 | 0.0214750 |
| NC\_012920 | 1438 | 0.0000000 | 0.0000000 | 0.1244444 | 0.0000000 | 0.0000000 | 0.0000000 | 0.2311111 | 0.0000000 | 0.000 | 0.0224690 | 86 | 18 | 15 | 39 | 43 | 41 | 15 | 3 | 4 | 0.1008940 | 0.8110939 | 0.9750929 | 0.1034712 |
| NC\_012920 | 1452 | 0.0229854 | 0.0000000 | 0.0000000 | 0.0000000 | 0.0000000 | 0.0000000 | 0.0000000 | 0.0000000 | 0.000 | 0.0075471 | 86 | 18 | 15 | 39 | 43 | 41 | 15 | 3 | 4 | 0.0078698 | 0.8123852 | 0.9907831 | 0.0079430 |
| NC\_012920 | 1503 | 0.0000000 | 0.0000000 | 0.0000000 | 0.0000000 | 0.0454300 | 0.0000000 | 0.0000000 | 0.0000000 | 0.000 | 0.0075756 | 86 | 18 | 15 | 39 | 43 | 40 | 15 | 3 | 4 | 0.0195278 | 0.8108835 | 0.9908400 | 0.0197083 |
| NC\_012920 | 1508 | 0.0000000 | 0.0000000 | 0.0000000 | 0.0000000 | 0.0000000 | 0.0487500 | 0.0000000 | 0.0000000 | 0.000 | 0.0075756 | 86 | 18 | 15 | 39 | 43 | 40 | 15 | 3 | 4 | 0.0212786 | 0.8107967 | 0.9908554 | 0.0214750 |
| NC\_012920 | 1518 | 0.0000000 | 0.0000000 | 0.0000000 | 0.0000000 | 0.0000000 | 0.0487500 | 0.0000000 | 0.0000000 | 0.000 | 0.0075756 | 86 | 18 | 15 | 39 | 43 | 40 | 15 | 3 | 4 | 0.0212786 | 0.8107967 | 0.9908554 | 0.0214750 |
| NC\_012920 | 1531 | 0.0000000 | 0.0000000 | 0.1244444 | 0.0000000 | 0.0000000 | 0.0000000 | 0.0000000 | 0.0000000 | 0.000 | 0.0075471 | 86 | 18 | 15 | 39 | 43 | 41 | 15 | 3 | 4 | 0.0631179 | 0.8103478 | 0.9912745 | 0.0636735 |
| NC\_012920 | 1555 | 0.0000000 | 0.0000000 | 0.0000000 | 0.0499671 | 0.0000000 | 0.0000000 | 0.0000000 | 0.0000000 | 0.000 | 0.0075471 | 86 | 18 | 15 | 39 | 43 | 41 | 15 | 3 | 4 | 0.0219362 | 0.8110365 | 0.9908987 | 0.0221377 |
| NC\_012920 | 1598 | 0.0229854 | 0.0000000 | 0.1244444 | 0.0000000 | 0.0886966 | 0.0000000 | 0.0000000 | 0.0000000 | 0.000 | 0.0298439 | 86 | 18 | 15 | 39 | 43 | 41 | 15 | 3 | 4 | 0.0281038 | 0.8151400 | 0.9644170 | 0.0291407 |
| NC\_012920 | 1664 | 0.0229854 | 0.0000000 | 0.0000000 | 0.0000000 | 0.0000000 | 0.0000000 | 0.0000000 | 0.0000000 | 0.000 | 0.0075187 | 86 | 18 | 15 | 39 | 43 | 42 | 15 | 3 | 4 | 0.0078841 | 0.8126166 | 0.9908205 | 0.0079571 |
| NC\_012920 | 1692 | 0.0000000 | 0.0000000 | 0.0000000 | 0.0499671 | 0.0000000 | 0.0000000 | 0.0000000 | 0.0000000 | 0.000 | 0.0075471 | 86 | 18 | 15 | 39 | 43 | 41 | 15 | 3 | 4 | 0.0219362 | 0.8110365 | 0.9908987 | 0.0221377 |
| NC\_012920 | 1694 | 0.0000000 | 0.0000000 | 0.0000000 | 0.0000000 | 0.0000000 | 0.0000000 | 0.3200000 | 0.0000000 | 0.000 | 0.0224690 | 86 | 18 | 15 | 39 | 43 | 41 | 15 | 3 | 4 | 0.1908046 | 0.8109791 | 0.9775804 | 0.1951805 |
| NC\_012920 | 1703 | 0.0229854 | 0.0000000 | 0.0000000 | 0.0000000 | 0.0454300 | 0.0000000 | 0.0000000 | 0.0000000 | 0.000 | 0.0150935 | 86 | 18 | 15 | 39 | 43 | 40 | 15 | 3 | 4 | 0.0099127 | 0.8133412 | 0.9816266 | 0.0100982 |
| NC\_012920 | 1717 | 0.0000000 | 0.0000000 | 0.0000000 | 0.0000000 | 0.0000000 | 0.0487500 | 0.0000000 | 0.0000000 | 0.000 | 0.0075756 | 86 | 18 | 15 | 39 | 43 | 40 | 15 | 3 | 4 | 0.0212786 | 0.8107967 | 0.9908554 | 0.0214750 |
| NC\_012920 | 1719 | 0.1297999 | 0.0000000 | 0.2311111 | 0.0000000 | 0.0454300 | 0.1387500 | 0.0000000 | 0.0000000 | 0.375 | 0.0939727 | 86 | 18 | 15 | 39 | 43 | 40 | 15 | 3 | 4 | 0.0437747 | 0.8288106 | 0.8915806 | 0.0490978 |
| NC\_012920 | 1721 | 0.0000000 | 0.0000000 | 0.0000000 | 0.0499671 | 0.0000000 | 0.0000000 | 0.0000000 | 0.0000000 | 0.000 | 0.0075756 | 86 | 18 | 15 | 39 | 43 | 40 | 15 | 3 | 4 | 0.0219221 | 0.8107678 | 0.9908610 | 0.0221243 |
| NC\_012920 | 1733 | 0.0673337 | 0.1975309 | 0.0000000 | 0.0973044 | 0.0000000 | 0.0512465 | 0.1244444 | 0.0000000 | 0.375 | 0.0736924 | 86 | 18 | 15 | 39 | 43 | 38 | 15 | 3 | 4 | 0.0404787 | 0.8210390 | 0.9138781 | 0.0442934 |
| NC\_012920 | 1734 | 0.0229854 | 0.0000000 | 0.0000000 | 0.0000000 | 0.0000000 | 0.0000000 | 0.0000000 | 0.0000000 | 0.000 | 0.0076335 | 86 | 18 | 15 | 39 | 43 | 38 | 15 | 3 | 4 | 0.0078265 | 0.8115265 | 0.9906673 | 0.0079002 |
| NC\_012920 | 1780 | 0.0000000 | 0.0000000 | 0.0000000 | 0.0499671 | 0.0000000 | 0.0000000 | 0.0000000 | 0.0000000 | 0.000 | 0.0075756 | 86 | 18 | 15 | 39 | 43 | 40 | 15 | 3 | 4 | 0.0219221 | 0.8107678 | 0.9908610 | 0.0221243 |
| NC\_012920 | 1811 | 0.2879935 | 0.4012346 | 0.1244444 | 0.4444444 | 0.4391563 | 0.1800000 | 0.2311111 | 0.4444444 | 0.375 | 0.3351791 | 86 | 18 | 15 | 39 | 43 | 40 | 15 | 3 | 4 | 0.0519410 | 0.8693201 | 0.6344620 | 0.0818662 |
| NC\_012920 | 1822 | 0.0000000 | 0.0000000 | 0.0000000 | 0.0000000 | 0.0000000 | 0.0487500 | 0.0000000 | 0.0000000 | 0.000 | 0.0075756 | 86 | 18 | 15 | 39 | 43 | 40 | 15 | 3 | 4 | 0.0212786 | 0.8107967 | 0.9908554 | 0.0214750 |
| NC\_012920 | 1888 | 0.1687399 | 0.2777778 | 0.2311111 | 0.0973044 | 0.2055165 | 0.1356336 | 0.1244444 | 0.0000000 | 0.000 | 0.1652893 | 86 | 18 | 15 | 39 | 43 | 41 | 15 | 3 | 4 | 0.0137121 | 0.8411387 | 0.8061880 | 0.0170086 |
| NC\_012920 | 1900 | 0.0229854 | 0.0000000 | 0.0000000 | 0.0000000 | 0.0000000 | 0.0475907 | 0.0000000 | 0.0000000 | 0.000 | 0.0150367 | 86 | 18 | 15 | 39 | 43 | 41 | 15 | 3 | 4 | 0.0105130 | 0.8135331 | 0.9817111 | 0.0107088 |
| NC\_012920 | 1977 | 0.0000000 | 0.0000000 | 0.0000000 | 0.0000000 | 0.0454300 | 0.0000000 | 0.0000000 | 0.0000000 | 0.000 | 0.0075471 | 86 | 18 | 15 | 39 | 43 | 41 | 15 | 3 | 4 | 0.0195420 | 0.8111513 | 0.9908777 | 0.0197219 |
| NC\_012920 | 2056 | 0.0229854 | 0.0000000 | 0.0000000 | 0.0000000 | 0.0000000 | 0.0000000 | 0.0000000 | 0.0000000 | 0.000 | 0.0075471 | 86 | 18 | 15 | 39 | 43 | 41 | 15 | 3 | 4 | 0.0078698 | 0.8123852 | 0.9907831 | 0.0079430 |
| NC\_012920 | 2065 | 0.0000000 | 0.0000000 | 0.0000000 | 0.0499671 | 0.0000000 | 0.0000000 | 0.0000000 | 0.0000000 | 0.000 | 0.0075471 | 86 | 18 | 15 | 39 | 43 | 41 | 15 | 3 | 4 | 0.0219362 | 0.8110365 | 0.9908987 | 0.0221377 |
| NC\_012920 | 2159 | 0.0000000 | 0.0000000 | 0.0000000 | 0.0000000 | 0.0000000 | 0.0000000 | 0.1244444 | 0.0000000 | 0.000 | 0.0076045 | 86 | 18 | 15 | 39 | 43 | 39 | 15 | 3 | 4 | 0.0630907 | 0.8097722 | 0.9912016 | 0.0636507 |
| NC\_012920 | 2218 | 0.0229854 | 0.1049383 | 0.1244444 | 0.0499671 | 0.0454300 | 0.0000000 | 0.1244444 | 0.0000000 | 0.000 | 0.0447526 | 86 | 18 | 15 | 39 | 43 | 39 | 15 | 3 | 4 | 0.0191057 | 0.8154828 | 0.9461698 | 0.0201927 |
| NC\_012920 | 2244 | 0.0000000 | 0.1049383 | 0.0000000 | 0.0000000 | 0.0000000 | 0.0000000 | 0.0000000 | 0.0000000 | 0.000 | 0.0076045 | 86 | 18 | 15 | 39 | 43 | 39 | 15 | 3 | 4 | 0.0519370 | 0.8098596 | 0.9910978 | 0.0524035 |
| NC\_012920 | 2245 | 0.0454300 | 0.0000000 | 0.0000000 | 0.0000000 | 0.0454300 | 0.0000000 | 0.0000000 | 0.0000000 | 0.000 | 0.0226385 | 86 | 18 | 15 | 39 | 43 | 39 | 15 | 3 | 4 | 0.0119422 | 0.8154828 | 0.9725706 | 0.0122790 |
| NC\_012920 | 2259 | 0.0229854 | 0.0000000 | 0.0000000 | 0.0000000 | 0.0000000 | 0.0000000 | 0.1244444 | 0.0000000 | 0.000 | 0.0152082 | 86 | 18 | 15 | 39 | 43 | 38 | 15 | 3 | 4 | 0.0317276 | 0.8119376 | 0.9818635 | 0.0323136 |
| NC\_012920 | 2294 | 0.0454300 | 0.0000000 | 0.0000000 | 0.0000000 | 0.0000000 | 0.0000000 | 0.0000000 | 0.0000000 | 0.000 | 0.0150935 | 86 | 18 | 15 | 39 | 43 | 40 | 15 | 3 | 4 | 0.0157712 | 0.8145267 | 0.9817619 | 0.0160642 |
| NC\_012920 | 2315 | 0.0000000 | 0.0000000 | 0.0000000 | 0.0000000 | 0.0454300 | 0.0000000 | 0.0000000 | 0.0000000 | 0.000 | 0.0076045 | 86 | 18 | 15 | 39 | 43 | 39 | 15 | 3 | 4 | 0.0195135 | 0.8105880 | 0.9908017 | 0.0196947 |
| NC\_012920 | 2332 | 0.0000000 | 0.0000000 | 0.0000000 | 0.0000000 | 0.0000000 | 0.0000000 | 0.1244444 | 0.0000000 | 0.000 | 0.0076045 | 86 | 18 | 15 | 39 | 43 | 39 | 15 | 3 | 4 | 0.0630907 | 0.8097722 | 0.9912016 | 0.0636507 |
| NC\_012920 | 2352 | 0.0229854 | 0.0000000 | 0.0000000 | 0.0000000 | 0.0000000 | 0.0000000 | 0.0000000 | 0.0000000 | 0.000 | 0.0076045 | 86 | 18 | 15 | 39 | 43 | 39 | 15 | 3 | 4 | 0.0078410 | 0.8118408 | 0.9907065 | 0.0079146 |
| NC\_012920 | 2355 | 0.1297999 | 0.0000000 | 0.0000000 | 0.1420118 | 0.1297999 | 0.0000000 | 0.0000000 | 0.0000000 | 0.000 | 0.0874075 | 86 | 18 | 15 | 39 | 43 | 39 | 15 | 3 | 4 | 0.0269911 | 0.8299924 | 0.8975313 | 0.0300726 |
| NC\_012920 | 2387 | 0.0229854 | 0.0000000 | 0.0000000 | 0.0000000 | 0.0000000 | 0.0000000 | 0.0000000 | 0.0000000 | 0.000 | 0.0075756 | 86 | 18 | 15 | 39 | 43 | 40 | 15 | 3 | 4 | 0.0078555 | 0.8121268 | 0.9907451 | 0.0079289 |
| NC\_012920 | 2416 | 0.0229854 | 0.1975309 | 0.0000000 | 0.0973044 | 0.0454300 | 0.0000000 | 0.1244444 | 0.0000000 | 0.000 | 0.0518151 | 86 | 18 | 15 | 39 | 43 | 40 | 15 | 3 | 4 | 0.0352269 | 0.8168110 | 0.9387988 | 0.0375233 |
| NC\_012920 | 2442 | 0.2401298 | 0.0000000 | 0.1244444 | 0.2945431 | 0.1297999 | 0.0487500 | 0.2311111 | 0.4444444 | 0.000 | 0.1842444 | 86 | 18 | 15 | 39 | 43 | 40 | 15 | 3 | 4 | 0.0437481 | 0.8476341 | 0.7921461 | 0.0552273 |
| NC\_012920 | 2483 | 0.0673337 | 0.0000000 | 0.0000000 | 0.0499671 | 0.0000000 | 0.0000000 | 0.0000000 | 0.0000000 | 0.000 | 0.0298439 | 86 | 18 | 15 | 39 | 43 | 41 | 15 | 3 | 4 | 0.0176896 | 0.8181818 | 0.9641694 | 0.0183469 |
| NC\_012920 | 2639 | 0.0229854 | 0.0000000 | 0.0000000 | 0.0000000 | 0.0454300 | 0.0000000 | 0.0000000 | 0.0000000 | 0.000 | 0.0150367 | 86 | 18 | 15 | 39 | 43 | 41 | 15 | 3 | 4 | 0.0099414 | 0.8135904 | 0.9817018 | 0.0101267 |
| NC\_012920 | 2702 | 0.0000000 | 0.0000000 | 0.1244444 | 0.0000000 | 0.0000000 | 0.0000000 | 0.0000000 | 0.0000000 | 0.000 | 0.0075471 | 86 | 18 | 15 | 39 | 43 | 41 | 15 | 3 | 4 | 0.0631179 | 0.8103478 | 0.9912745 | 0.0636735 |
| NC\_012920 | 2706 | 0.1873986 | 0.1975309 | 0.1244444 | 0.1840894 | 0.2055165 | 0.0928019 | 0.2311111 | 0.0000000 | 0.000 | 0.1714589 | 86 | 18 | 15 | 39 | 43 | 41 | 15 | 3 | 4 | 0.0096854 | 0.8436065 | 0.7987234 | 0.0121260 |
| NC\_012920 | 2707 | 0.0000000 | 0.0000000 | 0.0000000 | 0.0000000 | 0.0000000 | 0.0475907 | 0.0000000 | 0.0000000 | 0.000 | 0.0075471 | 86 | 18 | 15 | 39 | 43 | 41 | 15 | 3 | 4 | 0.0206807 | 0.8110939 | 0.9908876 | 0.0208709 |
| NC\_012920 | 2757 | 0.0000000 | 0.0000000 | 0.0000000 | 0.0499671 | 0.0000000 | 0.0000000 | 0.0000000 | 0.0000000 | 0.000 | 0.0075471 | 86 | 18 | 15 | 39 | 43 | 41 | 15 | 3 | 4 | 0.0219362 | 0.8110365 | 0.9908987 | 0.0221377 |
| NC\_012920 | 2758 | 0.0454300 | 0.0000000 | 0.1244444 | 0.0499671 | 0.0454300 | 0.0475907 | 0.0000000 | 0.0000000 | 0.000 | 0.0444215 | 86 | 18 | 15 | 39 | 43 | 41 | 15 | 3 | 4 | 0.0085445 | 0.8186123 | 0.9461993 | 0.0090303 |
| NC\_012920 | 2789 | 0.0229854 | 0.1975309 | 0.0000000 | 0.0973044 | 0.0886966 | 0.0000000 | 0.0000000 | 0.0000000 | 0.000 | 0.0518151 | 86 | 18 | 15 | 39 | 43 | 40 | 15 | 3 | 4 | 0.0356820 | 0.8175628 | 0.9388839 | 0.0380046 |
| NC\_012920 | 2833 | 0.0000000 | 0.1049383 | 0.0000000 | 0.0000000 | 0.0000000 | 0.0000000 | 0.0000000 | 0.0000000 | 0.000 | 0.0075471 | 86 | 18 | 15 | 39 | 43 | 41 | 15 | 3 | 4 | 0.0519645 | 0.8104339 | 0.9911715 | 0.0524274 |
| NC\_012920 | 2885 | 0.0454300 | 0.0000000 | 0.0000000 | 0.0499671 | 0.0454300 | 0.0475907 | 0.0000000 | 0.0000000 | 0.000 | 0.0371614 | 86 | 18 | 15 | 39 | 43 | 41 | 15 | 3 | 4 | 0.0051173 | 0.8182105 | 0.9548145 | 0.0053594 |
| NC\_012920 | 3010 | 0.2401298 | 0.2777778 | 0.0000000 | 0.1840894 | 0.0454300 | 0.0928019 | 0.2311111 | 0.0000000 | 0.375 | 0.1714589 | 86 | 18 | 15 | 39 | 43 | 41 | 15 | 3 | 4 | 0.0377657 | 0.8450126 | 0.8047560 | 0.0469281 |
| NC\_012920 | 3027 | 0.0000000 | 0.0000000 | 0.1244444 | 0.0000000 | 0.0454300 | 0.0000000 | 0.0000000 | 0.0000000 | 0.000 | 0.0150935 | 86 | 18 | 15 | 39 | 43 | 40 | 15 | 3 | 4 | 0.0376429 | 0.8112883 | 0.9820960 | 0.0383292 |
| NC\_012920 | 3170 | 0.0229854 | 0.0000000 | 0.0000000 | 0.0000000 | 0.0000000 | 0.0000000 | 0.0000000 | 0.0000000 | 0.000 | 0.0075756 | 86 | 18 | 15 | 39 | 43 | 40 | 15 | 3 | 4 | 0.0078555 | 0.8121268 | 0.9907451 | 0.0079289 |
| NC\_012920 | 3197 | 0.0000000 | 0.0000000 | 0.0000000 | 0.1420118 | 0.0454300 | 0.0000000 | 0.1244444 | 0.0000000 | 0.000 | 0.0372999 | 86 | 18 | 15 | 39 | 43 | 40 | 15 | 3 | 4 | 0.0460015 | 0.8144111 | 0.9563070 | 0.0481033 |
| NC\_012920 | 3203 | 0.0000000 | 0.0000000 | 0.0000000 | 0.0000000 | 0.0454300 | 0.0000000 | 0.0000000 | 0.0000000 | 0.000 | 0.0075756 | 86 | 18 | 15 | 39 | 43 | 40 | 15 | 3 | 4 | 0.0195278 | 0.8108835 | 0.9908400 | 0.0197083 |
| NC\_012920 | 3213 | 0.0229854 | 0.0000000 | 0.0000000 | 0.0000000 | 0.0000000 | 0.0000000 | 0.0000000 | 0.0000000 | 0.000 | 0.0075756 | 86 | 18 | 15 | 39 | 43 | 40 | 15 | 3 | 4 | 0.0078555 | 0.8121268 | 0.9907451 | 0.0079289 |
| NC\_012920 | 3254 | 0.0000000 | 0.0000000 | 0.0000000 | 0.0000000 | 0.0000000 | 0.0000000 | 0.1244444 | 0.0000000 | 0.000 | 0.0076045 | 86 | 18 | 15 | 39 | 43 | 39 | 15 | 3 | 4 | 0.0630907 | 0.8097722 | 0.9912016 | 0.0636507 |
| NC\_012920 | 3275 | 0.0229854 | 0.0000000 | 0.0000000 | 0.0000000 | 0.0000000 | 0.0000000 | 0.0000000 | 0.0000000 | 0.000 | 0.0076045 | 86 | 18 | 15 | 39 | 43 | 39 | 15 | 3 | 4 | 0.0078410 | 0.8118408 | 0.9907065 | 0.0079146 |
| NC\_012920 | 3290 | 0.0000000 | 0.0000000 | 0.0000000 | 0.0000000 | 0.0000000 | 0.0000000 | 0.0000000 | 0.4444444 | 0.000 | 0.0076045 | 86 | 18 | 15 | 39 | 43 | 39 | 15 | 3 | 4 | 0.3307791 | 0.8094225 | 0.9937127 | 0.3328719 |
| NC\_012920 | 3316 | 0.0229854 | 0.1049383 | 0.0000000 | 0.0000000 | 0.0000000 | 0.0499671 | 0.0000000 | 0.0000000 | 0.000 | 0.0226385 | 86 | 18 | 15 | 39 | 43 | 39 | 15 | 3 | 4 | 0.0197169 | 0.8134433 | 0.9727182 | 0.0202699 |
| NC\_012920 | 3338 | 0.0000000 | 0.0000000 | 0.0000000 | 0.0000000 | 0.0454300 | 0.0499671 | 0.0000000 | 0.0000000 | 0.000 | 0.0151506 | 86 | 18 | 15 | 39 | 43 | 39 | 15 | 3 | 4 | 0.0169442 | 0.8116951 | 0.9816508 | 0.0172609 |
| NC\_012920 | 3348 | 0.0454300 | 0.0000000 | 0.0000000 | 0.0000000 | 0.0000000 | 0.0000000 | 0.0000000 | 0.0000000 | 0.000 | 0.0151506 | 86 | 18 | 15 | 39 | 43 | 39 | 15 | 3 | 4 | 0.0157424 | 0.8142591 | 0.9816863 | 0.0160361 |
| NC\_012920 | 3357 | 0.0229854 | 0.0000000 | 0.0000000 | 0.0000000 | 0.0000000 | 0.0000000 | 0.0000000 | 0.0000000 | 0.000 | 0.0076045 | 86 | 18 | 15 | 39 | 43 | 39 | 15 | 3 | 4 | 0.0078410 | 0.8118408 | 0.9907065 | 0.0079146 |
| NC\_012920 | 3388 | 0.0229854 | 0.0000000 | 0.0000000 | 0.0000000 | 0.0000000 | 0.0000000 | 0.0000000 | 0.0000000 | 0.000 | 0.0075471 | 86 | 18 | 15 | 39 | 43 | 41 | 15 | 3 | 4 | 0.0078698 | 0.8123852 | 0.9907831 | 0.0079430 |
| NC\_012920 | 3394 | 0.0000000 | 0.0000000 | 0.1244444 | 0.0499671 | 0.0000000 | 0.0000000 | 0.0000000 | 0.0000000 | 0.375 | 0.0224690 | 86 | 18 | 15 | 39 | 43 | 41 | 15 | 3 | 4 | 0.1039198 | 0.8115243 | 0.9751899 | 0.1065637 |
| NC\_012920 | 3396 | 0.0000000 | 0.0000000 | 0.0000000 | 0.0499671 | 0.0000000 | 0.0475907 | 0.0000000 | 0.0000000 | 0.000 | 0.0150367 | 86 | 18 | 15 | 39 | 43 | 41 | 15 | 3 | 4 | 0.0175730 | 0.8121843 | 0.9818114 | 0.0178986 |
| NC\_012920 | 3397 | 0.0886966 | 0.1049383 | 0.1244444 | 0.0499671 | 0.1297999 | 0.0928019 | 0.0000000 | 0.0000000 | 0.000 | 0.0867769 | 86 | 18 | 15 | 39 | 43 | 41 | 15 | 3 | 4 | 0.0083215 | 0.8270202 | 0.8959460 | 0.0092879 |
| NC\_012920 | 3423 | 0.0000000 | 0.0000000 | 0.0000000 | 0.0000000 | 0.0000000 | 0.0475907 | 0.0000000 | 0.0000000 | 0.000 | 0.0075471 | 86 | 18 | 15 | 39 | 43 | 41 | 15 | 3 | 4 | 0.0206807 | 0.8110939 | 0.9908876 | 0.0208709 |
| NC\_012920 | 3434 | 0.0000000 | 0.0000000 | 0.0000000 | 0.0000000 | 0.0000000 | 0.0000000 | 0.1244444 | 0.0000000 | 0.000 | 0.0075471 | 86 | 18 | 15 | 39 | 43 | 41 | 15 | 3 | 4 | 0.0631179 | 0.8103478 | 0.9912745 | 0.0636735 |
| NC\_012920 | 3480 | 0.0886966 | 0.0000000 | 0.0000000 | 0.4260355 | 0.3309897 | 0.0000000 | 0.1244444 | 0.0000000 | 0.000 | 0.1781723 | 86 | 18 | 15 | 39 | 43 | 40 | 15 | 3 | 4 | 0.1390716 | 0.8377741 | 0.8169034 | 0.1702424 |
| NC\_012920 | 3483 | 0.0229854 | 0.0000000 | 0.0000000 | 0.0000000 | 0.0000000 | 0.0487500 | 0.0000000 | 0.0000000 | 0.000 | 0.0150935 | 86 | 18 | 15 | 39 | 43 | 40 | 15 | 3 | 4 | 0.0107915 | 0.8132545 | 0.9816409 | 0.0109933 |
| NC\_012920 | 3486 | 0.0000000 | 0.0000000 | 0.0000000 | 0.0000000 | 0.0000000 | 0.0487500 | 0.0000000 | 0.0000000 | 0.000 | 0.0075756 | 86 | 18 | 15 | 39 | 43 | 40 | 15 | 3 | 4 | 0.0212786 | 0.8107967 | 0.9908554 | 0.0214750 |
| NC\_012920 | 3505 | 0.0000000 | 0.0000000 | 0.0000000 | 0.0000000 | 0.0000000 | 0.0928019 | 0.0000000 | 0.0000000 | 0.000 | 0.0150367 | 86 | 18 | 15 | 39 | 43 | 41 | 15 | 3 | 4 | 0.0415193 | 0.8121843 | 0.9822547 | 0.0422694 |
| NC\_012920 | 3516 | 0.0454300 | 0.0000000 | 0.0000000 | 0.0000000 | 0.0454300 | 0.0000000 | 0.0000000 | 0.0000000 | 0.000 | 0.0224690 | 86 | 18 | 15 | 39 | 43 | 41 | 15 | 3 | 4 | 0.0120289 | 0.8159722 | 0.9727947 | 0.0123653 |
| NC\_012920 | 3531 | 0.1297999 | 0.0000000 | 0.0000000 | 0.0000000 | 0.0000000 | 0.0475907 | 0.0000000 | 0.4444444 | 0.000 | 0.0587695 | 86 | 18 | 15 | 39 | 43 | 41 | 15 | 3 | 4 | 0.0688236 | 0.8249254 | 0.9336609 | 0.0737137 |
| NC\_012920 | 3537 | 0.0000000 | 0.0000000 | 0.0000000 | 0.0000000 | 0.0000000 | 0.0475907 | 0.0000000 | 0.0000000 | 0.000 | 0.0075471 | 86 | 18 | 15 | 39 | 43 | 41 | 15 | 3 | 4 | 0.0206807 | 0.8110939 | 0.9908876 | 0.0208709 |
| NC\_012920 | 3546 | 0.0000000 | 0.1049383 | 0.0000000 | 0.0000000 | 0.0454300 | 0.0000000 | 0.0000000 | 0.0000000 | 0.000 | 0.0150367 | 86 | 18 | 15 | 39 | 43 | 41 | 15 | 3 | 4 | 0.0320729 | 0.8116391 | 0.9820678 | 0.0326585 |
| NC\_012920 | 3576 | 0.0000000 | 0.0000000 | 0.1244444 | 0.0499671 | 0.0000000 | 0.0000000 | 0.0000000 | 0.0000000 | 0.000 | 0.0150654 | 86 | 18 | 15 | 39 | 43 | 41 | 15 | 3 | 4 | 0.0407033 | 0.8114382 | 0.9821894 | 0.0414414 |
| NC\_012920 | 3579 | 0.0000000 | 0.0000000 | 0.0000000 | 0.0000000 | 0.0454300 | 0.0000000 | 0.0000000 | 0.0000000 | 0.000 | 0.0075756 | 86 | 18 | 15 | 39 | 43 | 40 | 15 | 3 | 4 | 0.0195278 | 0.8108835 | 0.9908400 | 0.0197083 |
| NC\_012920 | 3594 | 0.0673337 | 0.1975309 | 0.0000000 | 0.1420118 | 0.0886966 | 0.1387500 | 0.1244444 | 0.0000000 | 0.000 | 0.1007966 | 86 | 18 | 15 | 39 | 43 | 40 | 15 | 3 | 4 | 0.0148690 | 0.8269022 | 0.8799158 | 0.0168982 |
| NC\_012920 | 3666 | 0.0229854 | 0.0000000 | 0.0000000 | 0.0499671 | 0.0000000 | 0.0475907 | 0.0000000 | 0.0000000 | 0.000 | 0.0225264 | 86 | 18 | 15 | 39 | 43 | 41 | 15 | 3 | 4 | 0.0118191 | 0.8146235 | 0.9726743 | 0.0121512 |
| NC\_012920 | 3693 | 0.0000000 | 0.0000000 | 0.0000000 | 0.0000000 | 0.0000000 | 0.0000000 | 0.1244444 | 0.0000000 | 0.000 | 0.0075471 | 86 | 18 | 15 | 39 | 43 | 41 | 15 | 3 | 4 | 0.0631179 | 0.8103478 | 0.9912745 | 0.0636735 |
| NC\_012920 | 3705 | 0.0229854 | 0.1049383 | 0.1244444 | 0.0000000 | 0.0886966 | 0.0000000 | 0.0000000 | 0.0000000 | 0.000 | 0.0371614 | 86 | 18 | 15 | 39 | 43 | 41 | 15 | 3 | 4 | 0.0269456 | 0.8156279 | 0.9556660 | 0.0281956 |
| NC\_012920 | 3720 | 0.0673337 | 0.2777778 | 0.0000000 | 0.0000000 | 0.0454300 | 0.0475907 | 0.0000000 | 0.0000000 | 0.000 | 0.0587695 | 86 | 18 | 15 | 39 | 43 | 41 | 15 | 3 | 4 | 0.0528352 | 0.8207358 | 0.9321774 | 0.0566793 |
| NC\_012920 | 3736 | 0.0000000 | 0.0000000 | 0.0000000 | 0.0000000 | 0.0454300 | 0.0000000 | 0.0000000 | 0.0000000 | 0.000 | 0.0075471 | 86 | 18 | 15 | 39 | 43 | 41 | 15 | 3 | 4 | 0.0195420 | 0.8111513 | 0.9908777 | 0.0197219 |
| NC\_012920 | 3741 | 0.0000000 | 0.0000000 | 0.0000000 | 0.0000000 | 0.0886966 | 0.0475907 | 0.0000000 | 0.0000000 | 0.375 | 0.0298439 | 86 | 18 | 15 | 39 | 43 | 41 | 15 | 3 | 4 | 0.0778819 | 0.8135331 | 0.9661727 | 0.0806087 |
| NC\_012920 | 3768 | 0.0454300 | 0.0000000 | 0.0000000 | 0.0000000 | 0.0000000 | 0.0000000 | 0.0000000 | 0.0000000 | 0.000 | 0.0150935 | 86 | 18 | 15 | 39 | 43 | 40 | 15 | 3 | 4 | 0.0157712 | 0.8145267 | 0.9817619 | 0.0160642 |
| NC\_012920 | 3780 | 0.0000000 | 0.0000000 | 0.1244444 | 0.0000000 | 0.0000000 | 0.0000000 | 0.0000000 | 0.0000000 | 0.000 | 0.0075756 | 86 | 18 | 15 | 39 | 43 | 40 | 15 | 3 | 4 | 0.0631043 | 0.8100739 | 0.9912383 | 0.0636621 |
| NC\_012920 | 3796 | 0.0000000 | 0.0000000 | 0.0000000 | 0.0000000 | 0.0454300 | 0.0475907 | 0.0000000 | 0.0000000 | 0.000 | 0.0150654 | 86 | 18 | 15 | 39 | 43 | 41 | 15 | 3 | 4 | 0.0182449 | 0.8122991 | 0.9817917 | 0.0185832 |
| NC\_012920 | 3834 | 0.1095187 | 0.0000000 | 0.1244444 | 0.0000000 | 0.1297999 | 0.0487500 | 0.0000000 | 0.4444444 | 0.000 | 0.0801515 | 86 | 18 | 15 | 39 | 43 | 40 | 15 | 3 | 4 | 0.0441108 | 0.8264396 | 0.9072939 | 0.0486179 |
| NC\_012920 | 3843 | 0.0000000 | 0.0000000 | 0.0000000 | 0.0000000 | 0.0000000 | 0.0487500 | 0.0000000 | 0.0000000 | 0.000 | 0.0075756 | 86 | 18 | 15 | 39 | 43 | 40 | 15 | 3 | 4 | 0.0212786 | 0.8107967 | 0.9908554 | 0.0214750 |
| NC\_012920 | 3847 | 0.2401298 | 0.0000000 | 0.1244444 | 0.3261012 | 0.1297999 | 0.0487500 | 0.2311111 | 0.4444444 | 0.000 | 0.1902586 | 86 | 18 | 15 | 39 | 43 | 40 | 15 | 3 | 4 | 0.0493794 | 0.8483280 | 0.7867997 | 0.0627599 |
| NC\_012920 | 3861 | 0.0000000 | 0.0000000 | 0.0000000 | 0.0499671 | 0.0000000 | 0.0000000 | 0.0000000 | 0.0000000 | 0.000 | 0.0075756 | 86 | 18 | 15 | 39 | 43 | 40 | 15 | 3 | 4 | 0.0219221 | 0.8107678 | 0.9908610 | 0.0221243 |
| NC\_012920 | 3894 | 0.0000000 | 0.0000000 | 0.0000000 | 0.0000000 | 0.0454300 | 0.0000000 | 0.0000000 | 0.0000000 | 0.000 | 0.0076045 | 86 | 18 | 15 | 39 | 43 | 39 | 15 | 3 | 4 | 0.0195135 | 0.8105880 | 0.9908017 | 0.0196947 |
| NC\_012920 | 3915 | 0.0229854 | 0.0000000 | 0.0000000 | 0.0000000 | 0.0000000 | 0.0000000 | 0.0000000 | 0.0000000 | 0.000 | 0.0076045 | 86 | 18 | 15 | 39 | 43 | 39 | 15 | 3 | 4 | 0.0078410 | 0.8118408 | 0.9907065 | 0.0079146 |
| NC\_012920 | 3918 | 0.0454300 | 0.0000000 | 0.0000000 | 0.0000000 | 0.0000000 | 0.0499671 | 0.3200000 | 0.0000000 | 0.000 | 0.0447526 | 86 | 18 | 15 | 39 | 43 | 39 | 15 | 3 | 4 | 0.0912135 | 0.8164151 | 0.9501839 | 0.0959957 |
| NC\_012920 | 3921 | 0.0457004 | 0.0000000 | 0.0000000 | 0.0499671 | 0.0897783 | 0.0000000 | 0.0000000 | 0.0000000 | 0.000 | 0.0376144 | 86 | 18 | 15 | 39 | 43 | 39 | 15 | 3 | 4 | 0.0117247 | 0.8178136 | 0.9545455 | 0.0122830 |
| NC\_012920 | 3992 | 0.0454300 | 0.0000000 | 0.0000000 | 0.0000000 | 0.0454300 | 0.0000000 | 0.1244444 | 0.0000000 | 0.000 | 0.0300682 | 86 | 18 | 15 | 39 | 43 | 39 | 15 | 3 | 4 | 0.0191335 | 0.8158907 | 0.9638519 | 0.0198510 |
| NC\_012920 | 4012 | 0.0000000 | 0.0000000 | 0.0000000 | 0.0000000 | 0.0000000 | 0.0000000 | 0.0000000 | 0.0000000 | 0.375 | 0.0076335 | 86 | 18 | 15 | 39 | 43 | 38 | 15 | 3 | 4 | 0.2471154 | 0.8091191 | 0.9928971 | 0.2488832 |
| NC\_012920 | 4025 | 0.0229854 | 0.0000000 | 0.0000000 | 0.0000000 | 0.0000000 | 0.0000000 | 0.0000000 | 0.0000000 | 0.000 | 0.0076335 | 86 | 18 | 15 | 39 | 43 | 38 | 15 | 3 | 4 | 0.0078265 | 0.8115265 | 0.9906673 | 0.0079002 |
| NC\_012920 | 4048 | 0.0000000 | 0.0000000 | 0.0000000 | 0.0000000 | 0.0454300 | 0.0000000 | 0.0000000 | 0.0000000 | 0.000 | 0.0076335 | 86 | 18 | 15 | 39 | 43 | 38 | 15 | 3 | 4 | 0.0194991 | 0.8102641 | 0.9907627 | 0.0196809 |
| NC\_012920 | 4079 | 0.0000000 | 0.0000000 | 0.0000000 | 0.0000000 | 0.0454300 | 0.0000000 | 0.0000000 | 0.0000000 | 0.000 | 0.0076335 | 86 | 18 | 15 | 39 | 43 | 38 | 15 | 3 | 4 | 0.0194991 | 0.8102641 | 0.9907627 | 0.0196809 |
| NC\_012920 | 4092 | 0.0000000 | 0.0000000 | 0.0000000 | 0.0000000 | 0.0000000 | 0.0512465 | 0.0000000 | 0.0000000 | 0.000 | 0.0076335 | 86 | 18 | 15 | 39 | 43 | 38 | 15 | 3 | 4 | 0.0225709 | 0.8101173 | 0.9907900 | 0.0227807 |
| NC\_012920 | 4093 | 0.0229854 | 0.0000000 | 0.0000000 | 0.0000000 | 0.0000000 | 0.0997230 | 0.0000000 | 0.0000000 | 0.000 | 0.0227243 | 86 | 18 | 15 | 39 | 43 | 38 | 15 | 3 | 4 | 0.0277888 | 0.8136404 | 0.9728470 | 0.0285645 |
| NC\_012920 | 4104 | 0.0673337 | 0.1975309 | 0.0000000 | 0.1420118 | 0.0886966 | 0.0973044 | 0.1244444 | 0.0000000 | 0.000 | 0.0943127 | 86 | 18 | 15 | 39 | 43 | 39 | 15 | 3 | 4 | 0.0141529 | 0.8256512 | 0.8873884 | 0.0159489 |
| NC\_012920 | 4113 | 0.0000000 | 0.0000000 | 0.0000000 | 0.0000000 | 0.0000000 | 0.0000000 | 0.0000000 | 0.4444444 | 0.000 | 0.0076045 | 86 | 18 | 15 | 39 | 43 | 39 | 15 | 3 | 4 | 0.3307791 | 0.8094225 | 0.9937127 | 0.3328719 |
| NC\_012920 | 4188 | 0.0000000 | 0.1049383 | 0.0000000 | 0.0000000 | 0.0454300 | 0.0000000 | 0.0000000 | 0.0000000 | 0.000 | 0.0150935 | 86 | 18 | 15 | 39 | 43 | 40 | 15 | 3 | 4 | 0.0320448 | 0.8113750 | 0.9819938 | 0.0326324 |
| NC\_012920 | 4203 | 0.0000000 | 0.0000000 | 0.0000000 | 0.0000000 | 0.0454300 | 0.0000000 | 0.0000000 | 0.0000000 | 0.000 | 0.0075756 | 86 | 18 | 15 | 39 | 43 | 40 | 15 | 3 | 4 | 0.0195278 | 0.8108835 | 0.9908400 | 0.0197083 |
| NC\_012920 | 4216 | 0.3569497 | 0.4012346 | 0.4444444 | 0.2235371 | 0.0886966 | 0.1840894 | 0.3200000 | 0.4444444 | 0.375 | 0.2894936 | 86 | 18 | 15 | 39 | 43 | 39 | 15 | 3 | 4 | 0.0516306 | 0.8637900 | 0.6821601 | 0.0756869 |
| NC\_012920 | 4218 | 0.0000000 | 0.0000000 | 0.0000000 | 0.0000000 | 0.0000000 | 0.0499671 | 0.0000000 | 0.0000000 | 0.000 | 0.0076045 | 86 | 18 | 15 | 39 | 43 | 39 | 15 | 3 | 4 | 0.0219078 | 0.8104714 | 0.9908228 | 0.0221108 |
| NC\_012920 | 4232 | 0.0229854 | 0.0000000 | 0.0000000 | 0.0000000 | 0.0000000 | 0.0000000 | 0.0000000 | 0.0000000 | 0.000 | 0.0076045 | 86 | 18 | 15 | 39 | 43 | 39 | 15 | 3 | 4 | 0.0078410 | 0.8118408 | 0.9907065 | 0.0079146 |
| NC\_012920 | 4248 | 0.0000000 | 0.0000000 | 0.1244444 | 0.0000000 | 0.0886966 | 0.0499671 | 0.0000000 | 0.0000000 | 0.000 | 0.0300682 | 86 | 18 | 15 | 39 | 43 | 39 | 15 | 3 | 4 | 0.0315472 | 0.8132685 | 0.9641943 | 0.0327187 |
| NC\_012920 | 4257 | 0.0454300 | 0.0000000 | 0.0000000 | 0.0000000 | 0.0000000 | 0.0000000 | 0.0000000 | 0.0000000 | 0.000 | 0.0151506 | 86 | 18 | 15 | 39 | 43 | 39 | 15 | 3 | 4 | 0.0157424 | 0.8142591 | 0.9816863 | 0.0160361 |
| NC\_012920 | 4269 | 0.0229854 | 0.0000000 | 0.0000000 | 0.0000000 | 0.0000000 | 0.0000000 | 0.0000000 | 0.0000000 | 0.000 | 0.0076045 | 86 | 18 | 15 | 39 | 43 | 39 | 15 | 3 | 4 | 0.0078410 | 0.8118408 | 0.9907065 | 0.0079146 |
| NC\_012920 | 4312 | 0.0454300 | 0.0000000 | 0.0000000 | 0.0000000 | 0.0454300 | 0.0000000 | 0.0000000 | 0.0000000 | 0.000 | 0.0225535 | 86 | 18 | 15 | 39 | 43 | 40 | 15 | 3 | 4 | 0.0119857 | 0.8157412 | 0.9726836 | 0.0123223 |
| NC\_012920 | 4314 | 0.0454300 | 0.0000000 | 0.0000000 | 0.0499671 | 0.0000000 | 0.0000000 | 0.0000000 | 0.0000000 | 0.000 | 0.0225535 | 86 | 18 | 15 | 39 | 43 | 40 | 15 | 3 | 4 | 0.0127899 | 0.8156255 | 0.9727019 | 0.0131489 |
| NC\_012920 | 4336 | 0.0000000 | 0.1049383 | 0.0000000 | 0.0000000 | 0.0000000 | 0.0000000 | 0.0000000 | 0.0000000 | 0.000 | 0.0075756 | 86 | 18 | 15 | 39 | 43 | 40 | 15 | 3 | 4 | 0.0519508 | 0.8101606 | 0.9911350 | 0.0524155 |
| NC\_012920 | 4354 | 0.0673337 | 0.1049383 | 0.0000000 | 0.0499671 | 0.0000000 | 0.0487500 | 0.0000000 | 0.0000000 | 0.000 | 0.0445864 | 86 | 18 | 15 | 39 | 43 | 40 | 15 | 3 | 4 | 0.0126154 | 0.8195868 | 0.9462852 | 0.0133315 |
| NC\_012920 | 4386 | 0.0000000 | 0.0000000 | 0.0000000 | 0.0000000 | 0.0000000 | 0.0487500 | 0.0000000 | 0.0000000 | 0.000 | 0.0075756 | 86 | 18 | 15 | 39 | 43 | 40 | 15 | 3 | 4 | 0.0212786 | 0.8107967 | 0.9908554 | 0.0214750 |
| NC\_012920 | 4418 | 0.0229854 | 0.0000000 | 0.0000000 | 0.0000000 | 0.0000000 | 0.0000000 | 0.0000000 | 0.0000000 | 0.000 | 0.0076045 | 86 | 18 | 15 | 39 | 43 | 39 | 15 | 3 | 4 | 0.0078410 | 0.8118408 | 0.9907065 | 0.0079146 |
| NC\_012920 | 4454 | 0.0229854 | 0.0000000 | 0.0000000 | 0.0499671 | 0.0000000 | 0.0000000 | 0.0000000 | 0.0000000 | 0.000 | 0.0151506 | 86 | 18 | 15 | 39 | 43 | 39 | 15 | 3 | 4 | 0.0110855 | 0.8129480 | 0.9815699 | 0.0112936 |
| NC\_012920 | 4491 | 0.0000000 | 0.1049383 | 0.1244444 | 0.0000000 | 0.0454300 | 0.0000000 | 0.0000000 | 0.0000000 | 0.000 | 0.0225535 | 86 | 18 | 15 | 39 | 43 | 40 | 15 | 3 | 4 | 0.0375137 | 0.8117798 | 0.9732595 | 0.0385444 |
| NC\_012920 | 4502 | 0.0000000 | 0.0000000 | 0.0000000 | 0.0000000 | 0.0000000 | 0.0000000 | 0.0000000 | 0.0000000 | 0.375 | 0.0075756 | 86 | 18 | 15 | 39 | 43 | 40 | 15 | 3 | 4 | 0.2471374 | 0.8097558 | 0.9929566 | 0.2488904 |
| NC\_012920 | 4529 | 0.0673337 | 0.0000000 | 0.1244444 | 0.0000000 | 0.0000000 | 0.0487500 | 0.0000000 | 0.0000000 | 0.000 | 0.0372999 | 86 | 18 | 15 | 39 | 43 | 40 | 15 | 3 | 4 | 0.0206446 | 0.8184013 | 0.9553643 | 0.0216092 |
| NC\_012920 | 4541 | 0.0229854 | 0.0000000 | 0.0000000 | 0.0000000 | 0.0886966 | 0.0000000 | 0.0000000 | 0.0000000 | 0.000 | 0.0225535 | 86 | 18 | 15 | 39 | 43 | 40 | 15 | 3 | 4 | 0.0237478 | 0.8144978 | 0.9729676 | 0.0244076 |
| NC\_012920 | 4561 | 0.0000000 | 0.0000000 | 0.0000000 | 0.4049967 | 0.3309897 | 0.0000000 | 0.1244444 | 0.0000000 | 0.000 | 0.1469444 | 86 | 18 | 15 | 39 | 43 | 40 | 15 | 3 | 4 | 0.1747199 | 0.8278275 | 0.8535077 | 0.2047080 |
| NC\_012920 | 4562 | 0.0000000 | 0.0000000 | 0.0000000 | 0.0000000 | 0.0000000 | 0.0000000 | 0.1244444 | 0.0000000 | 0.000 | 0.0075756 | 86 | 18 | 15 | 39 | 43 | 40 | 15 | 3 | 4 | 0.0631043 | 0.8100739 | 0.9912383 | 0.0636621 |
| NC\_012920 | 4577 | 0.0000000 | 0.0000000 | 0.0000000 | 0.0000000 | 0.0454300 | 0.0000000 | 0.0000000 | 0.0000000 | 0.000 | 0.0075471 | 86 | 18 | 15 | 39 | 43 | 41 | 15 | 3 | 4 | 0.0195420 | 0.8111513 | 0.9908777 | 0.0197219 |
| NC\_012920 | 4579 | 0.0229854 | 0.0000000 | 0.0000000 | 0.0000000 | 0.0000000 | 0.0000000 | 0.0000000 | 0.0000000 | 0.000 | 0.0075471 | 86 | 18 | 15 | 39 | 43 | 41 | 15 | 3 | 4 | 0.0078698 | 0.8123852 | 0.9907831 | 0.0079430 |
| NC\_012920 | 4580 | 0.0000000 | 0.0000000 | 0.0000000 | 0.0000000 | 0.0454300 | 0.0000000 | 0.0000000 | 0.0000000 | 0.000 | 0.0075471 | 86 | 18 | 15 | 39 | 43 | 41 | 15 | 3 | 4 | 0.0195420 | 0.8111513 | 0.9908777 | 0.0197219 |
| NC\_012920 | 4586 | 0.0454300 | 0.0000000 | 0.0000000 | 0.0000000 | 0.0454300 | 0.0000000 | 0.0000000 | 0.0000000 | 0.000 | 0.0224690 | 86 | 18 | 15 | 39 | 43 | 41 | 15 | 3 | 4 | 0.0120289 | 0.8159722 | 0.9727947 | 0.0123653 |
| NC\_012920 | 4640 | 0.0000000 | 0.1049383 | 0.0000000 | 0.0000000 | 0.0454300 | 0.0000000 | 0.0000000 | 0.0000000 | 0.000 | 0.0150935 | 86 | 18 | 15 | 39 | 43 | 40 | 15 | 3 | 4 | 0.0320448 | 0.8113750 | 0.9819938 | 0.0326324 |
| NC\_012920 | 4646 | 0.0000000 | 0.0000000 | 0.0000000 | 0.0000000 | 0.0000000 | 0.0487500 | 0.1244444 | 0.0000000 | 0.000 | 0.0150935 | 86 | 18 | 15 | 39 | 43 | 40 | 15 | 3 | 4 | 0.0385217 | 0.8112015 | 0.9821104 | 0.0392234 |
| NC\_012920 | 4654 | 0.0000000 | 0.0000000 | 0.0000000 | 0.0000000 | 0.0000000 | 0.0000000 | 0.1244444 | 0.0000000 | 0.000 | 0.0075756 | 86 | 18 | 15 | 39 | 43 | 40 | 15 | 3 | 4 | 0.0631043 | 0.8100739 | 0.9912383 | 0.0636621 |
| NC\_012920 | 4655 | 0.0886966 | 0.1049383 | 0.1244444 | 0.0000000 | 0.0000000 | 0.1800000 | 0.0000000 | 0.0000000 | 0.000 | 0.0731542 | 86 | 18 | 15 | 39 | 43 | 40 | 15 | 3 | 4 | 0.0341007 | 0.8242132 | 0.9142703 | 0.0372982 |
| NC\_012920 | 4664 | 0.0000000 | 0.0000000 | 0.0000000 | 0.0499671 | 0.0000000 | 0.0000000 | 0.0000000 | 0.0000000 | 0.000 | 0.0076045 | 86 | 18 | 15 | 39 | 43 | 39 | 15 | 3 | 4 | 0.0219078 | 0.8104714 | 0.9908228 | 0.0221108 |
| NC\_012920 | 4676 | 0.0000000 | 0.0000000 | 0.0000000 | 0.0000000 | 0.0000000 | 0.0499671 | 0.0000000 | 0.0000000 | 0.000 | 0.0076045 | 86 | 18 | 15 | 39 | 43 | 39 | 15 | 3 | 4 | 0.0219078 | 0.8104714 | 0.9908228 | 0.0221108 |
| NC\_012920 | 4688 | 0.0000000 | 0.1049383 | 0.0000000 | 0.0499671 | 0.0454300 | 0.0000000 | 0.0000000 | 0.0000000 | 0.000 | 0.0225535 | 86 | 18 | 15 | 39 | 43 | 40 | 15 | 3 | 4 | 0.0236807 | 0.8124738 | 0.9728984 | 0.0243404 |
| NC\_012920 | 4703 | 0.0454300 | 0.0000000 | 0.0000000 | 0.0000000 | 0.0000000 | 0.0000000 | 0.0000000 | 0.0000000 | 0.000 | 0.0150935 | 86 | 18 | 15 | 39 | 43 | 40 | 15 | 3 | 4 | 0.0157712 | 0.8145267 | 0.9817619 | 0.0160642 |
| NC\_012920 | 4724 | 0.0229854 | 0.0000000 | 0.0000000 | 0.0000000 | 0.0000000 | 0.0000000 | 0.0000000 | 0.0000000 | 0.000 | 0.0075756 | 86 | 18 | 15 | 39 | 43 | 40 | 15 | 3 | 4 | 0.0078555 | 0.8121268 | 0.9907451 | 0.0079289 |
| NC\_012920 | 4732 | 0.0000000 | 0.0000000 | 0.0000000 | 0.0499671 | 0.0000000 | 0.0000000 | 0.0000000 | 0.0000000 | 0.000 | 0.0075756 | 86 | 18 | 15 | 39 | 43 | 40 | 15 | 3 | 4 | 0.0219221 | 0.8107678 | 0.9908610 | 0.0221243 |
| NC\_012920 | 4769 | 0.0454300 | 0.0000000 | 0.3200000 | 0.0000000 | 0.0000000 | 0.0000000 | 0.0000000 | 0.4444444 | 0.000 | 0.0445864 | 86 | 18 | 15 | 39 | 43 | 40 | 15 | 3 | 4 | 0.1437738 | 0.8156255 | 0.9531941 | 0.1508337 |
| NC\_012920 | 4772 | 0.0229854 | 0.0000000 | 0.0000000 | 0.0000000 | 0.0000000 | 0.0487500 | 0.0000000 | 0.0000000 | 0.000 | 0.0150935 | 86 | 18 | 15 | 39 | 43 | 40 | 15 | 3 | 4 | 0.0107915 | 0.8132545 | 0.9816409 | 0.0109933 |
| NC\_012920 | 4811 | 0.0000000 | 0.0000000 | 0.0000000 | 0.0000000 | 0.0000000 | 0.0000000 | 0.1244444 | 0.0000000 | 0.000 | 0.0075756 | 86 | 18 | 15 | 39 | 43 | 40 | 15 | 3 | 4 | 0.0631043 | 0.8100739 | 0.9912383 | 0.0636621 |
| NC\_012920 | 4823 | 0.1095187 | 0.0000000 | 0.0000000 | 0.0000000 | 0.0000000 | 0.0000000 | 0.0000000 | 0.0000000 | 0.000 | 0.0372999 | 86 | 18 | 15 | 39 | 43 | 40 | 15 | 3 | 4 | 0.0398864 | 0.8213795 | 0.9564000 | 0.0417048 |
| NC\_012920 | 4890 | 0.0000000 | 0.0000000 | 0.1244444 | 0.0000000 | 0.0000000 | 0.0000000 | 0.0000000 | 0.0000000 | 0.000 | 0.0076045 | 86 | 18 | 15 | 39 | 43 | 39 | 15 | 3 | 4 | 0.0630907 | 0.8097722 | 0.9912016 | 0.0636507 |
| NC\_012920 | 4904 | 0.0229854 | 0.0000000 | 0.0000000 | 0.0000000 | 0.0454300 | 0.0000000 | 0.0000000 | 0.0000000 | 0.000 | 0.0150935 | 86 | 18 | 15 | 39 | 43 | 40 | 15 | 3 | 4 | 0.0099127 | 0.8133412 | 0.9816266 | 0.0100982 |
| NC\_012920 | 4917 | 0.1297999 | 0.1975309 | 0.2311111 | 0.0000000 | 0.0886966 | 0.0950000 | 0.1244444 | 0.0000000 | 0.000 | 0.1075626 | 86 | 18 | 15 | 39 | 43 | 40 | 15 | 3 | 4 | 0.0220347 | 0.8301985 | 0.8732923 | 0.0252318 |
| NC\_012920 | 4937 | 0.0229854 | 0.0000000 | 0.0000000 | 0.0499671 | 0.0454300 | 0.0000000 | 0.1244444 | 0.0000000 | 0.000 | 0.0299556 | 86 | 18 | 15 | 39 | 43 | 40 | 15 | 3 | 4 | 0.0168449 | 0.8148448 | 0.9638569 | 0.0174766 |
| NC\_012920 | 4947 | 0.0229854 | 0.0000000 | 0.0000000 | 0.0000000 | 0.0000000 | 0.0000000 | 0.0000000 | 0.0000000 | 0.000 | 0.0075756 | 86 | 18 | 15 | 39 | 43 | 40 | 15 | 3 | 4 | 0.0078555 | 0.8121268 | 0.9907451 | 0.0079289 |
| NC\_012920 | 4949 | 0.0000000 | 0.0000000 | 0.0000000 | 0.0000000 | 0.0000000 | 0.0000000 | 0.3200000 | 0.0000000 | 0.000 | 0.0225535 | 86 | 18 | 15 | 39 | 43 | 40 | 15 | 3 | 4 | 0.1907692 | 0.8107100 | 0.9774877 | 0.1951628 |
| NC\_012920 | 4958 | 0.0000000 | 0.0000000 | 0.0000000 | 0.0000000 | 0.0000000 | 0.0000000 | 0.1244444 | 0.0000000 | 0.000 | 0.0075756 | 86 | 18 | 15 | 39 | 43 | 40 | 15 | 3 | 4 | 0.0631043 | 0.8100739 | 0.9912383 | 0.0636621 |
| NC\_012920 | 4960 | 0.0229854 | 0.0000000 | 0.0000000 | 0.0000000 | 0.0454300 | 0.0000000 | 0.0000000 | 0.0000000 | 0.000 | 0.0150935 | 86 | 18 | 15 | 39 | 43 | 40 | 15 | 3 | 4 | 0.0099127 | 0.8133412 | 0.9816266 | 0.0100982 |
| NC\_012920 | 4970 | 0.0000000 | 0.0000000 | 0.0000000 | 0.0000000 | 0.0000000 | 0.0487500 | 0.0000000 | 0.0000000 | 0.000 | 0.0075756 | 86 | 18 | 15 | 39 | 43 | 40 | 15 | 3 | 4 | 0.0212786 | 0.8107967 | 0.9908554 | 0.0214750 |
| NC\_012920 | 4976 | 0.0229854 | 0.0000000 | 0.0000000 | 0.0000000 | 0.0000000 | 0.0000000 | 0.0000000 | 0.0000000 | 0.000 | 0.0075756 | 86 | 18 | 15 | 39 | 43 | 40 | 15 | 3 | 4 | 0.0078555 | 0.8121268 | 0.9907451 | 0.0079289 |
| NC\_012920 | 4988 | 0.0229854 | 0.0000000 | 0.0000000 | 0.0000000 | 0.0000000 | 0.0000000 | 0.0000000 | 0.0000000 | 0.000 | 0.0075756 | 86 | 18 | 15 | 39 | 43 | 40 | 15 | 3 | 4 | 0.0078555 | 0.8121268 | 0.9907451 | 0.0079289 |
| NC\_012920 | 4991 | 0.0229854 | 0.1049383 | 0.1244444 | 0.0499671 | 0.0454300 | 0.0487500 | 0.1244444 | 0.0000000 | 0.000 | 0.0518151 | 86 | 18 | 15 | 39 | 43 | 40 | 15 | 3 | 4 | 0.0129301 | 0.8168688 | 0.9373888 | 0.0137938 |
| NC\_012920 | 5004 | 0.0454300 | 0.0000000 | 0.0000000 | 0.0000000 | 0.0454300 | 0.0000000 | 0.1244444 | 0.0000000 | 0.000 | 0.0299556 | 86 | 18 | 15 | 39 | 43 | 40 | 15 | 3 | 4 | 0.0191913 | 0.8161460 | 0.9640006 | 0.0199080 |
| NC\_012920 | 5027 | 0.0229854 | 0.0000000 | 0.0000000 | 0.0000000 | 0.0000000 | 0.0000000 | 0.0000000 | 0.0000000 | 0.000 | 0.0076335 | 86 | 18 | 15 | 39 | 43 | 38 | 15 | 3 | 4 | 0.0078265 | 0.8115265 | 0.9906673 | 0.0079002 |
| NC\_012920 | 5042 | 0.0232526 | 0.0000000 | 0.0000000 | 0.0000000 | 0.0000000 | 0.0000000 | 0.0000000 | 0.0000000 | 0.000 | 0.0076335 | 85 | 18 | 15 | 39 | 43 | 39 | 15 | 3 | 4 | 0.0079638 | 0.8128771 | 0.9906841 | 0.0080387 |
| NC\_012920 | 5046 | 0.0000000 | 0.0000000 | 0.0000000 | 0.0000000 | 0.0000000 | 0.0973044 | 0.0000000 | 0.0000000 | 0.000 | 0.0152082 | 85 | 18 | 15 | 39 | 43 | 39 | 15 | 3 | 4 | 0.0439560 | 0.8125835 | 0.9821068 | 0.0447569 |
| NC\_012920 | 5048 | 0.0000000 | 0.0000000 | 0.0000000 | 0.0000000 | 0.0000000 | 0.0000000 | 0.2311111 | 0.0000000 | 0.000 | 0.0152082 | 85 | 18 | 15 | 39 | 43 | 39 | 15 | 3 | 4 | 0.1266409 | 0.8111742 | 0.9836259 | 0.1287491 |
| NC\_012920 | 5054 | 0.0000000 | 0.0000000 | 0.0000000 | 0.0000000 | 0.0454300 | 0.0000000 | 0.0000000 | 0.0000000 | 0.000 | 0.0076335 | 85 | 18 | 15 | 39 | 43 | 39 | 15 | 3 | 4 | 0.0194991 | 0.8116440 | 0.9907784 | 0.0196806 |
| NC\_012920 | 5082 | 0.0000000 | 0.0000000 | 0.0000000 | 0.0000000 | 0.0000000 | 0.0499671 | 0.0000000 | 0.0000000 | 0.000 | 0.0076045 | 86 | 18 | 15 | 39 | 43 | 39 | 15 | 3 | 4 | 0.0219078 | 0.8104714 | 0.9908228 | 0.0221108 |
| NC\_012920 | 5090 | 0.0000000 | 0.0000000 | 0.0000000 | 0.0973044 | 0.0454300 | 0.0000000 | 0.0000000 | 0.0000000 | 0.000 | 0.0226385 | 86 | 18 | 15 | 39 | 43 | 39 | 15 | 3 | 4 | 0.0308428 | 0.8127440 | 0.9730047 | 0.0316985 |
| NC\_012920 | 5096 | 0.0229854 | 0.0000000 | 0.0000000 | 0.0000000 | 0.0000000 | 0.0000000 | 0.0000000 | 0.0000000 | 0.000 | 0.0076045 | 86 | 18 | 15 | 39 | 43 | 39 | 15 | 3 | 4 | 0.0078410 | 0.8118408 | 0.9907065 | 0.0079146 |
| NC\_012920 | 5108 | 0.0000000 | 0.0000000 | 0.0000000 | 0.0000000 | 0.0454300 | 0.0000000 | 0.0000000 | 0.0000000 | 0.000 | 0.0076045 | 86 | 18 | 15 | 39 | 43 | 39 | 15 | 3 | 4 | 0.0195135 | 0.8105880 | 0.9908017 | 0.0196947 |
| NC\_012920 | 5147 | 0.1095187 | 0.0000000 | 0.1244444 | 0.0499671 | 0.0886966 | 0.0000000 | 0.0000000 | 0.0000000 | 0.000 | 0.0663423 | 86 | 18 | 15 | 39 | 43 | 39 | 15 | 3 | 4 | 0.0192005 | 0.8250685 | 0.9211357 | 0.0208444 |
| NC\_012920 | 5186 | 0.0229854 | 0.1049383 | 0.0000000 | 0.0499671 | 0.0000000 | 0.0499671 | 0.0000000 | 0.0000000 | 0.000 | 0.0300682 | 86 | 18 | 15 | 39 | 43 | 39 | 15 | 3 | 4 | 0.0145717 | 0.8145504 | 0.9636241 | 0.0151217 |
| NC\_012920 | 5201 | 0.0000000 | 0.1049383 | 0.0000000 | 0.0000000 | 0.0000000 | 0.0973044 | 0.0000000 | 0.0000000 | 0.000 | 0.0226385 | 86 | 18 | 15 | 39 | 43 | 39 | 15 | 3 | 4 | 0.0417340 | 0.8120156 | 0.9732841 | 0.0428796 |
| NC\_012920 | 5208 | 0.0229854 | 0.0000000 | 0.0000000 | 0.0000000 | 0.0000000 | 0.0000000 | 0.0000000 | 0.0000000 | 0.000 | 0.0076045 | 86 | 18 | 15 | 39 | 43 | 39 | 15 | 3 | 4 | 0.0078410 | 0.8118408 | 0.9907065 | 0.0079146 |
| NC\_012920 | 5231 | 0.0454300 | 0.0000000 | 0.0000000 | 0.0000000 | 0.0454300 | 0.0000000 | 0.0000000 | 0.0000000 | 0.000 | 0.0226385 | 86 | 18 | 15 | 39 | 43 | 39 | 15 | 3 | 4 | 0.0119422 | 0.8154828 | 0.9725706 | 0.0122790 |
| NC\_012920 | 5237 | 0.0229854 | 0.1049383 | 0.0000000 | 0.0000000 | 0.0454300 | 0.0000000 | 0.0000000 | 0.0000000 | 0.000 | 0.0226385 | 86 | 18 | 15 | 39 | 43 | 39 | 15 | 3 | 4 | 0.0189126 | 0.8135598 | 0.9726997 | 0.0194434 |
| NC\_012920 | 5252 | 0.0000000 | 0.0000000 | 0.0000000 | 0.0499671 | 0.0000000 | 0.0000000 | 0.0000000 | 0.0000000 | 0.000 | 0.0075756 | 86 | 18 | 15 | 39 | 43 | 40 | 15 | 3 | 4 | 0.0219221 | 0.8107678 | 0.9908610 | 0.0221243 |
| NC\_012920 | 5267 | 0.0229854 | 0.0000000 | 0.0000000 | 0.0000000 | 0.0000000 | 0.0487500 | 0.0000000 | 0.0000000 | 0.000 | 0.0150935 | 86 | 18 | 15 | 39 | 43 | 40 | 15 | 3 | 4 | 0.0107915 | 0.8132545 | 0.9816409 | 0.0109933 |
| NC\_012920 | 5285 | 0.0229854 | 0.0000000 | 0.0000000 | 0.0000000 | 0.0000000 | 0.0000000 | 0.0000000 | 0.0000000 | 0.000 | 0.0075756 | 86 | 18 | 15 | 39 | 43 | 40 | 15 | 3 | 4 | 0.0078555 | 0.8121268 | 0.9907451 | 0.0079289 |
| NC\_012920 | 5301 | 0.0000000 | 0.0000000 | 0.0000000 | 0.0000000 | 0.0000000 | 0.0487500 | 0.0000000 | 0.0000000 | 0.000 | 0.0075756 | 86 | 18 | 15 | 39 | 43 | 40 | 15 | 3 | 4 | 0.0212786 | 0.8107967 | 0.9908554 | 0.0214750 |
| NC\_012920 | 5315 | 0.0000000 | 0.0000000 | 0.1244444 | 0.0000000 | 0.0000000 | 0.0000000 | 0.0000000 | 0.0000000 | 0.000 | 0.0075756 | 86 | 18 | 15 | 39 | 43 | 40 | 15 | 3 | 4 | 0.0631043 | 0.8100739 | 0.9912383 | 0.0636621 |
| NC\_012920 | 5319 | 0.0000000 | 0.0000000 | 0.0000000 | 0.0000000 | 0.0000000 | 0.0487500 | 0.0000000 | 0.0000000 | 0.000 | 0.0075756 | 86 | 18 | 15 | 39 | 43 | 40 | 15 | 3 | 4 | 0.0212786 | 0.8107967 | 0.9908554 | 0.0214750 |
| NC\_012920 | 5360 | 0.0000000 | 0.0000000 | 0.0000000 | 0.0000000 | 0.0886966 | 0.0499671 | 0.0000000 | 0.0000000 | 0.375 | 0.0300682 | 86 | 18 | 15 | 39 | 43 | 39 | 15 | 3 | 4 | 0.0780911 | 0.8129480 | 0.9659017 | 0.0808479 |
| NC\_012920 | 5390 | 0.0229854 | 0.1049383 | 0.0000000 | 0.0000000 | 0.0000000 | 0.0499671 | 0.0000000 | 0.0000000 | 0.000 | 0.0226385 | 86 | 18 | 15 | 39 | 43 | 39 | 15 | 3 | 4 | 0.0197169 | 0.8134433 | 0.9727182 | 0.0202699 |
| NC\_012920 | 5426 | 0.0886966 | 0.2777778 | 0.0000000 | 0.0499671 | 0.0454300 | 0.0973044 | 0.0000000 | 0.0000000 | 0.000 | 0.0804440 | 86 | 18 | 15 | 39 | 43 | 39 | 15 | 3 | 4 | 0.0356490 | 0.8247188 | 0.9059361 | 0.0393505 |
| NC\_012920 | 5432 | 0.0454300 | 0.0000000 | 0.0000000 | 0.0499671 | 0.0000000 | 0.0499671 | 0.0000000 | 0.0000000 | 0.000 | 0.0300682 | 86 | 18 | 15 | 39 | 43 | 39 | 15 | 3 | 4 | 0.0093236 | 0.8164734 | 0.9635165 | 0.0096766 |
| NC\_012920 | 5442 | 0.0454300 | 0.0000000 | 0.0000000 | 0.0000000 | 0.0454300 | 0.0000000 | 0.0000000 | 0.0000000 | 0.000 | 0.0226385 | 86 | 18 | 15 | 39 | 43 | 39 | 15 | 3 | 4 | 0.0119422 | 0.8154828 | 0.9725706 | 0.0122790 |
| NC\_012920 | 5460 | 0.1095187 | 0.0000000 | 0.0000000 | 0.0973044 | 0.0454300 | 0.1420118 | 0.1244444 | 0.0000000 | 0.000 | 0.0874075 | 86 | 18 | 15 | 39 | 43 | 39 | 15 | 3 | 4 | 0.0143519 | 0.8280986 | 0.8959628 | 0.0160185 |
| NC\_012920 | 5471 | 0.0229854 | 0.0000000 | 0.0000000 | 0.0000000 | 0.1297999 | 0.0000000 | 0.0000000 | 0.0000000 | 0.000 | 0.0300682 | 86 | 18 | 15 | 39 | 43 | 39 | 15 | 3 | 4 | 0.0405850 | 0.8153371 | 0.9646185 | 0.0420736 |
| NC\_012920 | 5558 | 0.0229854 | 0.0000000 | 0.0000000 | 0.0000000 | 0.0000000 | 0.0950000 | 0.0000000 | 0.0000000 | 0.000 | 0.0225535 | 86 | 18 | 15 | 39 | 43 | 40 | 15 | 3 | 4 | 0.0261002 | 0.8143243 | 0.9730270 | 0.0268237 |
| NC\_012920 | 5568 | 0.0229854 | 0.0000000 | 0.0000000 | 0.0000000 | 0.0000000 | 0.0000000 | 0.0000000 | 0.0000000 | 0.000 | 0.0075756 | 86 | 18 | 15 | 39 | 43 | 40 | 15 | 3 | 4 | 0.0078555 | 0.8121268 | 0.9907451 | 0.0079289 |
| NC\_012920 | 5585 | 0.0000000 | 0.0000000 | 0.0000000 | 0.0000000 | 0.0000000 | 0.0487500 | 0.0000000 | 0.0000000 | 0.000 | 0.0075756 | 86 | 18 | 15 | 39 | 43 | 40 | 15 | 3 | 4 | 0.0212786 | 0.8107967 | 0.9908554 | 0.0214750 |
| NC\_012920 | 5601 | 0.0000000 | 0.0000000 | 0.0000000 | 0.0000000 | 0.0000000 | 0.0475907 | 0.0000000 | 0.0000000 | 0.000 | 0.0075471 | 86 | 18 | 15 | 39 | 43 | 41 | 15 | 3 | 4 | 0.0206807 | 0.8110939 | 0.9908876 | 0.0208709 |
| NC\_012920 | 5603 | 0.0454300 | 0.0000000 | 0.0000000 | 0.0000000 | 0.0454300 | 0.0000000 | 0.0000000 | 0.0000000 | 0.000 | 0.0224690 | 86 | 18 | 15 | 39 | 43 | 41 | 15 | 3 | 4 | 0.0120289 | 0.8159722 | 0.9727947 | 0.0123653 |
| NC\_012920 | 5628 | 0.0229854 | 0.0000000 | 0.0000000 | 0.0000000 | 0.0000000 | 0.0475907 | 0.0000000 | 0.0000000 | 0.000 | 0.0150367 | 86 | 18 | 15 | 39 | 43 | 41 | 15 | 3 | 4 | 0.0105130 | 0.8135331 | 0.9817111 | 0.0107088 |
| NC\_012920 | 5633 | 0.0229854 | 0.0000000 | 0.0000000 | 0.0499671 | 0.0000000 | 0.0000000 | 0.0000000 | 0.0000000 | 0.000 | 0.0150367 | 86 | 18 | 15 | 39 | 43 | 41 | 15 | 3 | 4 | 0.0111431 | 0.8134757 | 0.9817214 | 0.0113506 |
| NC\_012920 | 5671 | 0.0000000 | 0.0000000 | 0.0000000 | 0.0499671 | 0.0000000 | 0.0000000 | 0.0000000 | 0.0000000 | 0.000 | 0.0075471 | 86 | 18 | 15 | 39 | 43 | 41 | 15 | 3 | 4 | 0.0219362 | 0.8110365 | 0.9908987 | 0.0221377 |
| NC\_012920 | 5711 | 0.0229854 | 0.0000000 | 0.0000000 | 0.0000000 | 0.0454300 | 0.0000000 | 0.0000000 | 0.0000000 | 0.000 | 0.0150367 | 86 | 18 | 15 | 39 | 43 | 41 | 15 | 3 | 4 | 0.0099414 | 0.8135904 | 0.9817018 | 0.0101267 |
| NC\_012920 | 5773 | 0.0000000 | 0.1049383 | 0.0000000 | 0.0000000 | 0.0000000 | 0.0000000 | 0.0000000 | 0.0000000 | 0.000 | 0.0075471 | 86 | 18 | 15 | 39 | 43 | 41 | 15 | 3 | 4 | 0.0519645 | 0.8104339 | 0.9911715 | 0.0524274 |
| NC\_012920 | 5774 | 0.0000000 | 0.0000000 | 0.0000000 | 0.0000000 | 0.0000000 | 0.0000000 | 0.0000000 | 0.0000000 | 0.375 | 0.0075471 | 86 | 18 | 15 | 39 | 43 | 41 | 15 | 3 | 4 | 0.2471483 | 0.8100321 | 0.9929857 | 0.2488941 |
| NC\_012920 | 5783 | 0.0000000 | 0.0000000 | 0.0000000 | 0.0000000 | 0.0454300 | 0.0000000 | 0.0000000 | 0.0000000 | 0.000 | 0.0075471 | 86 | 18 | 15 | 39 | 43 | 41 | 15 | 3 | 4 | 0.0195420 | 0.8111513 | 0.9908777 | 0.0197219 |
| NC\_012920 | 5790 | 0.0229854 | 0.0000000 | 0.0000000 | 0.0000000 | 0.0454300 | 0.0000000 | 0.0000000 | 0.0000000 | 0.000 | 0.0150367 | 86 | 18 | 15 | 39 | 43 | 41 | 15 | 3 | 4 | 0.0099414 | 0.8135904 | 0.9817018 | 0.0101267 |
| NC\_012920 | 5895 | 0.0229854 | 0.0000000 | 0.0000000 | 0.0000000 | 0.0000000 | 0.0000000 | 0.0000000 | 0.0000000 | 0.000 | 0.0075471 | 86 | 18 | 15 | 39 | 43 | 41 | 15 | 3 | 4 | 0.0078698 | 0.8123852 | 0.9907831 | 0.0079430 |
| NC\_012920 | 5910 | 0.0000000 | 0.0000000 | 0.0000000 | 0.0000000 | 0.0000000 | 0.0000000 | 0.2311111 | 0.0000000 | 0.000 | 0.0150367 | 86 | 18 | 15 | 39 | 43 | 41 | 15 | 3 | 4 | 0.1267176 | 0.8106921 | 0.9838023 | 0.1288039 |
| NC\_012920 | 5911 | 0.0229854 | 0.0000000 | 0.0000000 | 0.0000000 | 0.0000000 | 0.0000000 | 0.0000000 | 0.0000000 | 0.000 | 0.0075471 | 86 | 18 | 15 | 39 | 43 | 41 | 15 | 3 | 4 | 0.0078698 | 0.8123852 | 0.9907831 | 0.0079430 |
| NC\_012920 | 5951 | 0.0000000 | 0.0000000 | 0.0000000 | 0.0499671 | 0.0000000 | 0.0475907 | 0.0000000 | 0.0000000 | 0.000 | 0.0150367 | 86 | 18 | 15 | 39 | 43 | 41 | 15 | 3 | 4 | 0.0175730 | 0.8121843 | 0.9818114 | 0.0178986 |
| NC\_012920 | 5964 | 0.0454300 | 0.0000000 | 0.0000000 | 0.0000000 | 0.0000000 | 0.0000000 | 0.0000000 | 0.0000000 | 0.000 | 0.0150367 | 86 | 18 | 15 | 39 | 43 | 41 | 15 | 3 | 4 | 0.0157998 | 0.8147670 | 0.9818363 | 0.0160920 |
| NC\_012920 | 5981 | 0.0229854 | 0.0000000 | 0.0000000 | 0.0000000 | 0.0000000 | 0.0000000 | 0.0000000 | 0.0000000 | 0.000 | 0.0075471 | 86 | 18 | 15 | 39 | 43 | 41 | 15 | 3 | 4 | 0.0078698 | 0.8123852 | 0.9907831 | 0.0079430 |
| NC\_012920 | 5999 | 0.0886966 | 0.0000000 | 0.0000000 | 0.0000000 | 0.0000000 | 0.0000000 | 0.1244444 | 0.4444444 | 0.000 | 0.0444215 | 86 | 18 | 15 | 39 | 43 | 41 | 15 | 3 | 4 | 0.0766901 | 0.8198175 | 0.9499708 | 0.0807289 |
| NC\_012920 | 6011 | 0.0000000 | 0.0000000 | 0.0000000 | 0.0000000 | 0.0454300 | 0.0000000 | 0.0000000 | 0.0000000 | 0.000 | 0.0075756 | 86 | 18 | 15 | 39 | 43 | 40 | 15 | 3 | 4 | 0.0195278 | 0.8108835 | 0.9908400 | 0.0197083 |
| NC\_012920 | 6026 | 0.0229854 | 0.1049383 | 0.1244444 | 0.0499671 | 0.0454300 | 0.0000000 | 0.1244444 | 0.0000000 | 0.000 | 0.0444215 | 86 | 18 | 15 | 39 | 43 | 41 | 15 | 3 | 4 | 0.0192798 | 0.8159722 | 0.9466096 | 0.0203673 |
| NC\_012920 | 6045 | 0.0673337 | 0.2777778 | 0.0000000 | 0.0000000 | 0.0454300 | 0.0475907 | 0.0000000 | 0.0000000 | 0.000 | 0.0587695 | 86 | 18 | 15 | 39 | 43 | 41 | 15 | 3 | 4 | 0.0528352 | 0.8207358 | 0.9321774 | 0.0566793 |
| NC\_012920 | 6047 | 0.0000000 | 0.0000000 | 0.0000000 | 0.0000000 | 0.0000000 | 0.0000000 | 0.1244444 | 0.0000000 | 0.000 | 0.0075471 | 86 | 18 | 15 | 39 | 43 | 41 | 15 | 3 | 4 | 0.0631179 | 0.8103478 | 0.9912745 | 0.0636735 |
| NC\_012920 | 6050 | 0.0454300 | 0.0000000 | 0.0000000 | 0.0000000 | 0.0000000 | 0.0000000 | 0.0000000 | 0.0000000 | 0.000 | 0.0150367 | 86 | 18 | 15 | 39 | 43 | 41 | 15 | 3 | 4 | 0.0157998 | 0.8147670 | 0.9818363 | 0.0160920 |
| NC\_012920 | 6071 | 0.0000000 | 0.0000000 | 0.0000000 | 0.0499671 | 0.0000000 | 0.0475907 | 0.0000000 | 0.0000000 | 0.000 | 0.0150367 | 86 | 18 | 15 | 39 | 43 | 41 | 15 | 3 | 4 | 0.0175730 | 0.8121843 | 0.9818114 | 0.0178986 |
| NC\_012920 | 6116 | 0.0000000 | 0.0000000 | 0.0000000 | 0.0000000 | 0.0000000 | 0.0464853 | 0.0000000 | 0.0000000 | 0.000 | 0.0075187 | 86 | 18 | 15 | 39 | 43 | 42 | 15 | 3 | 4 | 0.0201118 | 0.8113635 | 0.9909196 | 0.0202961 |
| NC\_012920 | 6146 | 0.0000000 | 0.0000000 | 0.0000000 | 0.0000000 | 0.0000000 | 0.0000000 | 0.1244444 | 0.0000000 | 0.000 | 0.0075187 | 86 | 18 | 15 | 39 | 43 | 42 | 15 | 3 | 4 | 0.0631313 | 0.8105945 | 0.9913101 | 0.0636847 |
| NC\_012920 | 6152 | 0.0673337 | 0.2777778 | 0.0000000 | 0.0000000 | 0.0454300 | 0.0464853 | 0.0000000 | 0.0000000 | 0.000 | 0.0585546 | 86 | 18 | 15 | 39 | 43 | 42 | 15 | 3 | 4 | 0.0528720 | 0.8209327 | 0.9324442 | 0.0567026 |
| NC\_012920 | 6164 | 0.0229854 | 0.0000000 | 0.0000000 | 0.0000000 | 0.0000000 | 0.0464853 | 0.0000000 | 0.0000000 | 0.000 | 0.0149804 | 86 | 18 | 15 | 39 | 43 | 42 | 15 | 3 | 4 | 0.0102489 | 0.8137843 | 0.9817803 | 0.0104391 |
| NC\_012920 | 6185 | 0.0454300 | 0.0000000 | 0.0000000 | 0.0000000 | 0.0454300 | 0.0000000 | 0.0000000 | 0.0000000 | 0.000 | 0.0223852 | 86 | 18 | 15 | 39 | 43 | 42 | 15 | 3 | 4 | 0.0120717 | 0.8161766 | 0.9729042 | 0.0124079 |
| NC\_012920 | 6221 | 0.0454300 | 0.0000000 | 0.0000000 | 0.0499671 | 0.0000000 | 0.0464853 | 0.0000000 | 0.0000000 | 0.000 | 0.0298184 | 86 | 18 | 15 | 39 | 43 | 42 | 15 | 3 | 4 | 0.0118720 | 0.8172303 | 0.9639460 | 0.0123160 |
| NC\_012920 | 6231 | 0.0000000 | 0.0000000 | 0.0000000 | 0.0000000 | 0.0000000 | 0.0000000 | 0.1244444 | 0.0000000 | 0.000 | 0.0075187 | 86 | 18 | 15 | 39 | 43 | 42 | 15 | 3 | 4 | 0.0631313 | 0.8105945 | 0.9913101 | 0.0636847 |
| NC\_012920 | 6249 | 0.0000000 | 0.0000000 | 0.0000000 | 0.0000000 | 0.0000000 | 0.0464853 | 0.0000000 | 0.0000000 | 0.000 | 0.0075187 | 86 | 18 | 15 | 39 | 43 | 42 | 15 | 3 | 4 | 0.0201118 | 0.8113635 | 0.9909196 | 0.0202961 |
| NC\_012920 | 6253 | 0.0454300 | 0.0000000 | 0.0000000 | 0.0000000 | 0.0454300 | 0.0000000 | 0.0000000 | 0.0000000 | 0.000 | 0.0223852 | 86 | 18 | 15 | 39 | 43 | 42 | 15 | 3 | 4 | 0.0120717 | 0.8161766 | 0.9729042 | 0.0124079 |
| NC\_012920 | 6257 | 0.0229854 | 0.0000000 | 0.0000000 | 0.0000000 | 0.0454300 | 0.0000000 | 0.0000000 | 0.0000000 | 0.000 | 0.0149804 | 86 | 18 | 15 | 39 | 43 | 42 | 15 | 3 | 4 | 0.0099699 | 0.8138127 | 0.9817758 | 0.0101550 |
| NC\_012920 | 6260 | 0.0229854 | 0.0000000 | 0.0000000 | 0.0499671 | 0.0000000 | 0.0000000 | 0.3200000 | 0.0000000 | 0.000 | 0.0370239 | 86 | 18 | 15 | 39 | 43 | 42 | 15 | 3 | 4 | 0.1106741 | 0.8147241 | 0.9595860 | 0.1153352 |
| NC\_012920 | 6261 | 0.1095187 | 0.0000000 | 0.0000000 | 0.0499671 | 0.0886966 | 0.0000000 | 0.0000000 | 0.0000000 | 0.000 | 0.0585546 | 86 | 18 | 15 | 39 | 43 | 42 | 15 | 3 | 4 | 0.0216347 | 0.8251477 | 0.9305726 | 0.0232488 |
| NC\_012920 | 6267 | 0.0229854 | 0.0000000 | 0.0000000 | 0.0000000 | 0.0000000 | 0.0000000 | 0.0000000 | 0.0000000 | 0.000 | 0.0075187 | 86 | 18 | 15 | 39 | 43 | 42 | 15 | 3 | 4 | 0.0078841 | 0.8126166 | 0.9908205 | 0.0079571 |
| NC\_012920 | 6272 | 0.0229854 | 0.0000000 | 0.0000000 | 0.0000000 | 0.0454300 | 0.0000000 | 0.0000000 | 0.0000000 | 0.000 | 0.0149804 | 86 | 18 | 15 | 39 | 43 | 42 | 15 | 3 | 4 | 0.0099699 | 0.8138127 | 0.9817758 | 0.0101550 |
| NC\_012920 | 6284 | 0.0000000 | 0.0000000 | 0.0000000 | 0.0499671 | 0.0000000 | 0.0000000 | 0.0000000 | 0.0000000 | 0.000 | 0.0075187 | 86 | 18 | 15 | 39 | 43 | 42 | 15 | 3 | 4 | 0.0219503 | 0.8112780 | 0.9909357 | 0.0221511 |
| NC\_012920 | 6297 | 0.0000000 | 0.0000000 | 0.0000000 | 0.0000000 | 0.0000000 | 0.0464853 | 0.0000000 | 0.0000000 | 0.000 | 0.0075187 | 86 | 18 | 15 | 39 | 43 | 42 | 15 | 3 | 4 | 0.0201118 | 0.8113635 | 0.9909196 | 0.0202961 |
| NC\_012920 | 6340 | 0.0229854 | 0.1975309 | 0.0000000 | 0.0499671 | 0.0000000 | 0.0000000 | 0.1244444 | 0.0000000 | 0.000 | 0.0371614 | 86 | 18 | 15 | 39 | 43 | 41 | 15 | 3 | 4 | 0.0471858 | 0.8147957 | 0.9565438 | 0.0493295 |
| NC\_012920 | 6345 | 0.0000000 | 0.0000000 | 0.0000000 | 0.0499671 | 0.0000000 | 0.0000000 | 0.0000000 | 0.0000000 | 0.000 | 0.0075471 | 86 | 18 | 15 | 39 | 43 | 41 | 15 | 3 | 4 | 0.0219362 | 0.8110365 | 0.9908987 | 0.0221377 |
| NC\_012920 | 6347 | 0.0229854 | 0.0000000 | 0.1244444 | 0.0973044 | 0.0000000 | 0.0000000 | 0.0000000 | 0.0000000 | 0.000 | 0.0298439 | 86 | 18 | 15 | 39 | 43 | 41 | 15 | 3 | 4 | 0.0305257 | 0.8149105 | 0.9644956 | 0.0316494 |
| NC\_012920 | 6371 | 0.0454300 | 0.0000000 | 0.0000000 | 0.0000000 | 0.0000000 | 0.0475907 | 0.0000000 | 0.0000000 | 0.000 | 0.0224690 | 86 | 18 | 15 | 39 | 43 | 41 | 15 | 3 | 4 | 0.0124114 | 0.8159148 | 0.9728034 | 0.0127583 |
| NC\_012920 | 6378 | 0.0000000 | 0.0000000 | 0.0000000 | 0.0000000 | 0.0454300 | 0.0000000 | 0.0000000 | 0.0000000 | 0.000 | 0.0075471 | 86 | 18 | 15 | 39 | 43 | 41 | 15 | 3 | 4 | 0.0195420 | 0.8111513 | 0.9908777 | 0.0197219 |
| NC\_012920 | 6386 | 0.0886966 | 0.0000000 | 0.0000000 | 0.0000000 | 0.0000000 | 0.0000000 | 0.0000000 | 0.4444444 | 0.000 | 0.0371614 | 86 | 18 | 15 | 39 | 43 | 41 | 15 | 3 | 4 | 0.0865763 | 0.8194157 | 0.9585753 | 0.0903177 |
| NC\_012920 | 6401 | 0.0229854 | 0.0000000 | 0.0000000 | 0.0000000 | 0.0000000 | 0.0000000 | 0.0000000 | 0.0000000 | 0.000 | 0.0075471 | 86 | 18 | 15 | 39 | 43 | 41 | 15 | 3 | 4 | 0.0078698 | 0.8123852 | 0.9907831 | 0.0079430 |
| NC\_012920 | 6446 | 0.0229854 | 0.1049383 | 0.1244444 | 0.0000000 | 0.0886966 | 0.0000000 | 0.0000000 | 0.0000000 | 0.000 | 0.0372999 | 86 | 18 | 15 | 39 | 43 | 40 | 15 | 3 | 4 | 0.0268742 | 0.8153942 | 0.9554847 | 0.0281262 |
| NC\_012920 | 6464 | 0.0000000 | 0.0000000 | 0.0000000 | 0.0000000 | 0.0000000 | 0.0487500 | 0.0000000 | 0.0000000 | 0.000 | 0.0075756 | 86 | 18 | 15 | 39 | 43 | 40 | 15 | 3 | 4 | 0.0212786 | 0.8107967 | 0.9908554 | 0.0214750 |
| NC\_012920 | 6506 | 0.0454300 | 0.0000000 | 0.0000000 | 0.0000000 | 0.0000000 | 0.0000000 | 0.0000000 | 0.0000000 | 0.000 | 0.0150367 | 86 | 18 | 15 | 39 | 43 | 41 | 15 | 3 | 4 | 0.0157998 | 0.8147670 | 0.9818363 | 0.0160920 |
| NC\_012920 | 6518 | 0.0454300 | 0.0000000 | 0.0000000 | 0.0000000 | 0.0000000 | 0.0000000 | 0.0000000 | 0.0000000 | 0.000 | 0.0150367 | 86 | 18 | 15 | 39 | 43 | 41 | 15 | 3 | 4 | 0.0157998 | 0.8147670 | 0.9818363 | 0.0160920 |
| NC\_012920 | 6527 | 0.0000000 | 0.0000000 | 0.0000000 | 0.0000000 | 0.0000000 | 0.0487500 | 0.0000000 | 0.0000000 | 0.000 | 0.0075756 | 86 | 18 | 15 | 39 | 43 | 40 | 15 | 3 | 4 | 0.0212786 | 0.8107967 | 0.9908554 | 0.0214750 |
| NC\_012920 | 6620 | 0.0229854 | 0.0000000 | 0.1244444 | 0.0000000 | 0.0886966 | 0.0000000 | 0.0000000 | 0.0000000 | 0.000 | 0.0299556 | 86 | 18 | 15 | 39 | 43 | 40 | 15 | 3 | 4 | 0.0280469 | 0.8149026 | 0.9642712 | 0.0290861 |
| NC\_012920 | 6635 | 0.0000000 | 0.0000000 | 0.0000000 | 0.0000000 | 0.0000000 | 0.0487500 | 0.0000000 | 0.0000000 | 0.000 | 0.0075756 | 86 | 18 | 15 | 39 | 43 | 40 | 15 | 3 | 4 | 0.0212786 | 0.8107967 | 0.9908554 | 0.0214750 |
| NC\_012920 | 6659 | 0.0000000 | 0.0000000 | 0.0000000 | 0.0000000 | 0.0000000 | 0.0000000 | 0.0000000 | 0.0000000 | 0.375 | 0.0075756 | 86 | 18 | 15 | 39 | 43 | 40 | 15 | 3 | 4 | 0.2471374 | 0.8097558 | 0.9929566 | 0.2488904 |
| NC\_012920 | 6671 | 0.1297999 | 0.1049383 | 0.1244444 | 0.0000000 | 0.0886966 | 0.0000000 | 0.0000000 | 0.0000000 | 0.000 | 0.0731542 | 86 | 18 | 15 | 39 | 43 | 40 | 15 | 3 | 4 | 0.0263647 | 0.8268155 | 0.9138557 | 0.0288499 |
| NC\_012920 | 6680 | 0.0229854 | 0.1049383 | 0.0000000 | 0.0000000 | 0.1297999 | 0.0000000 | 0.2311111 | 0.0000000 | 0.000 | 0.0518151 | 86 | 18 | 15 | 39 | 43 | 40 | 15 | 3 | 4 | 0.0523711 | 0.8168399 | 0.9398885 | 0.0557206 |
| NC\_012920 | 6734 | 0.0000000 | 0.0000000 | 0.1244444 | 0.0000000 | 0.0454300 | 0.0000000 | 0.0000000 | 0.0000000 | 0.000 | 0.0150367 | 86 | 18 | 15 | 39 | 43 | 41 | 15 | 3 | 4 | 0.0376709 | 0.8115530 | 0.9821696 | 0.0383547 |
| NC\_012920 | 6749 | 0.0454300 | 0.0000000 | 0.0000000 | 0.0000000 | 0.0000000 | 0.0000000 | 0.0000000 | 0.0000000 | 0.000 | 0.0150367 | 86 | 18 | 15 | 39 | 43 | 41 | 15 | 3 | 4 | 0.0157998 | 0.8147670 | 0.9818363 | 0.0160920 |
| NC\_012920 | 6755 | 0.0454300 | 0.1975309 | 0.1244444 | 0.0000000 | 0.0454300 | 0.0475907 | 0.0000000 | 0.0000000 | 0.000 | 0.0516242 | 86 | 18 | 15 | 39 | 43 | 41 | 15 | 3 | 4 | 0.0289740 | 0.8184401 | 0.9387512 | 0.0308644 |
| NC\_012920 | 6764 | 0.0229854 | 0.0000000 | 0.0000000 | 0.0000000 | 0.0000000 | 0.0000000 | 0.0000000 | 0.0000000 | 0.000 | 0.0075471 | 86 | 18 | 15 | 39 | 43 | 41 | 15 | 3 | 4 | 0.0078698 | 0.8123852 | 0.9907831 | 0.0079430 |
| NC\_012920 | 6809 | 0.0000000 | 0.0000000 | 0.1244444 | 0.0000000 | 0.0000000 | 0.0000000 | 0.0000000 | 0.0000000 | 0.000 | 0.0075471 | 86 | 18 | 15 | 39 | 43 | 41 | 15 | 3 | 4 | 0.0631179 | 0.8103478 | 0.9912745 | 0.0636735 |
| NC\_012920 | 6827 | 0.0229854 | 0.0000000 | 0.0000000 | 0.0000000 | 0.0886966 | 0.0000000 | 0.0000000 | 0.0000000 | 0.000 | 0.0224690 | 86 | 18 | 15 | 39 | 43 | 41 | 15 | 3 | 4 | 0.0237904 | 0.8147383 | 0.9730779 | 0.0244486 |
| NC\_012920 | 6915 | 0.0229854 | 0.0000000 | 0.0000000 | 0.0000000 | 0.0000000 | 0.0000000 | 0.0000000 | 0.0000000 | 0.000 | 0.0076045 | 86 | 18 | 15 | 39 | 43 | 39 | 15 | 3 | 4 | 0.0078410 | 0.8118408 | 0.9907065 | 0.0079146 |
| NC\_012920 | 6917 | 0.0000000 | 0.0000000 | 0.0000000 | 0.0499671 | 0.0000000 | 0.0000000 | 0.0000000 | 0.0000000 | 0.000 | 0.0076045 | 86 | 18 | 15 | 39 | 43 | 39 | 15 | 3 | 4 | 0.0219078 | 0.8104714 | 0.9908228 | 0.0221108 |
| NC\_012920 | 6962 | 0.1297999 | 0.1049383 | 0.0000000 | 0.0499671 | 0.0000000 | 0.0499671 | 0.0000000 | 0.0000000 | 0.000 | 0.0663423 | 86 | 18 | 15 | 39 | 43 | 39 | 15 | 3 | 4 | 0.0248862 | 0.8260591 | 0.9216869 | 0.0270007 |
| NC\_012920 | 7022 | 0.0000000 | 0.0000000 | 0.0000000 | 0.0499671 | 0.0000000 | 0.0000000 | 0.0000000 | 0.0000000 | 0.000 | 0.0075756 | 86 | 18 | 15 | 39 | 43 | 40 | 15 | 3 | 4 | 0.0219221 | 0.8107678 | 0.9908610 | 0.0221243 |
| NC\_012920 | 7028 | 0.1687399 | 0.1049383 | 0.1244444 | 0.1420118 | 0.1687399 | 0.0000000 | 0.2311111 | 0.0000000 | 0.000 | 0.1340485 | 86 | 18 | 15 | 39 | 43 | 40 | 15 | 3 | 4 | 0.0206108 | 0.8369935 | 0.8431461 | 0.0244451 |
| NC\_012920 | 7055 | 0.0000000 | 0.0000000 | 0.0000000 | 0.0000000 | 0.0000000 | 0.0487500 | 0.0000000 | 0.0000000 | 0.000 | 0.0075756 | 86 | 18 | 15 | 39 | 43 | 40 | 15 | 3 | 4 | 0.0212786 | 0.8107967 | 0.9908554 | 0.0214750 |
| NC\_012920 | 7076 | 0.0000000 | 0.0000000 | 0.0000000 | 0.0000000 | 0.0000000 | 0.0487500 | 0.0000000 | 0.0000000 | 0.000 | 0.0075756 | 86 | 18 | 15 | 39 | 43 | 40 | 15 | 3 | 4 | 0.0212786 | 0.8107967 | 0.9908554 | 0.0214750 |
| NC\_012920 | 7080 | 0.0229854 | 0.0000000 | 0.0000000 | 0.0000000 | 0.0000000 | 0.0000000 | 0.0000000 | 0.0000000 | 0.000 | 0.0075756 | 86 | 18 | 15 | 39 | 43 | 40 | 15 | 3 | 4 | 0.0078555 | 0.8121268 | 0.9907451 | 0.0079289 |
| NC\_012920 | 7129 | 0.0229854 | 0.0000000 | 0.0000000 | 0.0000000 | 0.0000000 | 0.0000000 | 0.0000000 | 0.0000000 | 0.000 | 0.0075756 | 86 | 18 | 15 | 39 | 43 | 40 | 15 | 3 | 4 | 0.0078555 | 0.8121268 | 0.9907451 | 0.0079289 |
| NC\_012920 | 7146 | 0.0454300 | 0.0000000 | 0.0000000 | 0.0499671 | 0.0454300 | 0.0487500 | 0.0000000 | 0.0000000 | 0.000 | 0.0372999 | 86 | 18 | 15 | 39 | 43 | 40 | 15 | 3 | 4 | 0.0051685 | 0.8179676 | 0.9546349 | 0.0054142 |
| NC\_012920 | 7175 | 0.0000000 | 0.0000000 | 0.0000000 | 0.0973044 | 0.0454300 | 0.0000000 | 0.0000000 | 0.0000000 | 0.000 | 0.0225535 | 86 | 18 | 15 | 39 | 43 | 40 | 15 | 3 | 4 | 0.0308854 | 0.8130232 | 0.9731165 | 0.0317387 |
| NC\_012920 | 7193 | 0.0229854 | 0.0000000 | 0.0000000 | 0.0000000 | 0.0000000 | 0.0000000 | 0.0000000 | 0.0000000 | 0.000 | 0.0075756 | 86 | 18 | 15 | 39 | 43 | 40 | 15 | 3 | 4 | 0.0078555 | 0.8121268 | 0.9907451 | 0.0079289 |
| NC\_012920 | 7220 | 0.0454300 | 0.0000000 | 0.0000000 | 0.0000000 | 0.0000000 | 0.0487500 | 0.0000000 | 0.0000000 | 0.000 | 0.0225535 | 86 | 18 | 15 | 39 | 43 | 40 | 15 | 3 | 4 | 0.0125738 | 0.8156544 | 0.9726969 | 0.0129267 |
| NC\_012920 | 7247 | 0.0454300 | 0.0000000 | 0.0000000 | 0.0000000 | 0.0000000 | 0.0000000 | 0.0000000 | 0.0000000 | 0.000 | 0.0150935 | 86 | 18 | 15 | 39 | 43 | 40 | 15 | 3 | 4 | 0.0157712 | 0.8145267 | 0.9817619 | 0.0160642 |
| NC\_012920 | 7256 | 0.0673337 | 0.1975309 | 0.0000000 | 0.1420118 | 0.0886966 | 0.0950000 | 0.1244444 | 0.0000000 | 0.000 | 0.0939727 | 86 | 18 | 15 | 39 | 43 | 40 | 15 | 3 | 4 | 0.0141411 | 0.8258902 | 0.8878255 | 0.0159278 |
| NC\_012920 | 7269 | 0.0000000 | 0.0000000 | 0.0000000 | 0.0000000 | 0.0454300 | 0.0487500 | 0.0000000 | 0.0000000 | 0.000 | 0.0150935 | 86 | 18 | 15 | 39 | 43 | 40 | 15 | 3 | 4 | 0.0166500 | 0.8120112 | 0.9817217 | 0.0169600 |
| NC\_012920 | 7274 | 0.0229854 | 0.1975309 | 0.0000000 | 0.0973044 | 0.0454300 | 0.0000000 | 0.0000000 | 0.0000000 | 0.000 | 0.0445864 | 86 | 18 | 15 | 39 | 43 | 40 | 15 | 3 | 4 | 0.0379982 | 0.8164062 | 0.9474621 | 0.0401052 |
| NC\_012920 | 7334 | 0.0000000 | 0.0000000 | 0.0000000 | 0.0000000 | 0.0454300 | 0.0000000 | 0.0000000 | 0.0000000 | 0.000 | 0.0076045 | 86 | 18 | 15 | 39 | 43 | 39 | 15 | 3 | 4 | 0.0195135 | 0.8105880 | 0.9908017 | 0.0196947 |
| NC\_012920 | 7364 | 0.0673337 | 0.1049383 | 0.0000000 | 0.0499671 | 0.0000000 | 0.0475907 | 0.0000000 | 0.0000000 | 0.000 | 0.0444215 | 86 | 18 | 15 | 39 | 43 | 41 | 15 | 3 | 4 | 0.0125987 | 0.8198175 | 0.9464981 | 0.0133109 |
| NC\_012920 | 7389 | 0.0229854 | 0.0000000 | 0.0000000 | 0.0499671 | 0.0000000 | 0.0475907 | 0.0000000 | 0.0000000 | 0.000 | 0.0224690 | 86 | 18 | 15 | 39 | 43 | 41 | 15 | 3 | 4 | 0.0092950 | 0.8146235 | 0.9726743 | 0.0095562 |
| NC\_012920 | 7424 | 0.0000000 | 0.0000000 | 0.0000000 | 0.0499671 | 0.0454300 | 0.0475907 | 0.0000000 | 0.0000000 | 0.000 | 0.0224690 | 86 | 18 | 15 | 39 | 43 | 41 | 15 | 3 | 4 | 0.0132156 | 0.8133896 | 0.9727411 | 0.0135859 |
| NC\_012920 | 7476 | 0.1297999 | 0.0000000 | 0.1244444 | 0.0499671 | 0.0000000 | 0.0000000 | 0.0000000 | 0.0000000 | 0.000 | 0.0589860 | 86 | 18 | 15 | 39 | 43 | 40 | 15 | 3 | 4 | 0.0344951 | 0.8250517 | 0.9309725 | 0.0370527 |
| NC\_012920 | 7498 | 0.0000000 | 0.0000000 | 0.0000000 | 0.0499671 | 0.0000000 | 0.0000000 | 0.0000000 | 0.0000000 | 0.000 | 0.0075756 | 86 | 18 | 15 | 39 | 43 | 40 | 15 | 3 | 4 | 0.0219221 | 0.8107678 | 0.9908610 | 0.0221243 |
| NC\_012920 | 7521 | 0.0673337 | 0.1975309 | 0.0000000 | 0.1840894 | 0.0886966 | 0.0950000 | 0.1244444 | 0.0000000 | 0.000 | 0.1007966 | 86 | 18 | 15 | 39 | 43 | 40 | 15 | 3 | 4 | 0.0189798 | 0.8268155 | 0.8804044 | 0.0215580 |
| NC\_012920 | 7581 | 0.0229854 | 0.1049383 | 0.1244444 | 0.0499671 | 0.0454300 | 0.0000000 | 0.1244444 | 0.0000000 | 0.000 | 0.0444215 | 86 | 18 | 15 | 39 | 43 | 41 | 15 | 3 | 4 | 0.0192798 | 0.8159722 | 0.9466096 | 0.0203673 |
| NC\_012920 | 7598 | 0.0229854 | 0.0000000 | 0.1244444 | 0.0000000 | 0.0454300 | 0.0000000 | 0.0000000 | 0.0000000 | 0.000 | 0.0224690 | 86 | 18 | 15 | 39 | 43 | 41 | 15 | 3 | 4 | 0.0227450 | 0.8139922 | 0.9730244 | 0.0233755 |
| NC\_012920 | 7600 | 0.0229854 | 0.0000000 | 0.0000000 | 0.0000000 | 0.0000000 | 0.0000000 | 0.0000000 | 0.0000000 | 0.000 | 0.0075471 | 86 | 18 | 15 | 39 | 43 | 41 | 15 | 3 | 4 | 0.0078698 | 0.8123852 | 0.9907831 | 0.0079430 |
| NC\_012920 | 7609 | 0.0000000 | 0.0000000 | 0.0000000 | 0.0000000 | 0.0000000 | 0.0475907 | 0.0000000 | 0.0000000 | 0.000 | 0.0075471 | 86 | 18 | 15 | 39 | 43 | 41 | 15 | 3 | 4 | 0.0206807 | 0.8110939 | 0.9908876 | 0.0208709 |
| NC\_012920 | 7610 | 0.0000000 | 0.0000000 | 0.0000000 | 0.0499671 | 0.0454300 | 0.0000000 | 0.0000000 | 0.0000000 | 0.000 | 0.0150367 | 86 | 18 | 15 | 39 | 43 | 41 | 15 | 3 | 4 | 0.0170015 | 0.8122417 | 0.9818021 | 0.0173166 |
| NC\_012920 | 7632 | 0.0000000 | 0.0000000 | 0.1244444 | 0.0000000 | 0.0000000 | 0.0000000 | 0.0000000 | 0.0000000 | 0.000 | 0.0075756 | 86 | 18 | 15 | 39 | 43 | 40 | 15 | 3 | 4 | 0.0631043 | 0.8100739 | 0.9912383 | 0.0636621 |
| NC\_012920 | 7645 | 0.0000000 | 0.0000000 | 0.0000000 | 0.0973044 | 0.0454300 | 0.0000000 | 0.0000000 | 0.0000000 | 0.000 | 0.0225535 | 86 | 18 | 15 | 39 | 43 | 40 | 15 | 3 | 4 | 0.0308854 | 0.8130232 | 0.9731165 | 0.0317387 |
| NC\_012920 | 7648 | 0.0000000 | 0.0000000 | 0.0000000 | 0.0000000 | 0.0454300 | 0.0000000 | 0.0000000 | 0.0000000 | 0.000 | 0.0075756 | 86 | 18 | 15 | 39 | 43 | 40 | 15 | 3 | 4 | 0.0195278 | 0.8108835 | 0.9908400 | 0.0197083 |
| NC\_012920 | 7657 | 0.0229854 | 0.0000000 | 0.2311111 | 0.0000000 | 0.0000000 | 0.0000000 | 0.0000000 | 0.4444444 | 0.000 | 0.0298439 | 86 | 18 | 15 | 39 | 43 | 41 | 15 | 3 | 4 | 0.1398748 | 0.8131887 | 0.9684335 | 0.1444340 |
| NC\_012920 | 7674 | 0.0000000 | 0.0000000 | 0.0000000 | 0.0000000 | 0.0000000 | 0.0475907 | 0.1244444 | 0.0000000 | 0.000 | 0.0150367 | 86 | 18 | 15 | 39 | 43 | 41 | 15 | 3 | 4 | 0.0382424 | 0.8114956 | 0.9821790 | 0.0389363 |
| NC\_012920 | 7738 | 0.0229854 | 0.0000000 | 0.0000000 | 0.0000000 | 0.0000000 | 0.0000000 | 0.0000000 | 0.0000000 | 0.000 | 0.0075756 | 86 | 18 | 15 | 39 | 43 | 40 | 15 | 3 | 4 | 0.0078555 | 0.8121268 | 0.9907451 | 0.0079289 |
| NC\_012920 | 7765 | 0.0000000 | 0.0000000 | 0.0000000 | 0.0000000 | 0.0000000 | 0.0475907 | 0.0000000 | 0.0000000 | 0.000 | 0.0075471 | 86 | 18 | 15 | 39 | 43 | 41 | 15 | 3 | 4 | 0.0206807 | 0.8110939 | 0.9908876 | 0.0208709 |
| NC\_012920 | 7768 | 0.0000000 | 0.0000000 | 0.0000000 | 0.0499671 | 0.0000000 | 0.0000000 | 0.0000000 | 0.0000000 | 0.000 | 0.0075471 | 86 | 18 | 15 | 39 | 43 | 41 | 15 | 3 | 4 | 0.0219362 | 0.8110365 | 0.9908987 | 0.0221377 |
| NC\_012920 | 7771 | 0.0229854 | 0.1975309 | 0.0000000 | 0.0973044 | 0.0454300 | 0.0000000 | 0.0000000 | 0.0000000 | 0.000 | 0.0444215 | 86 | 18 | 15 | 39 | 43 | 41 | 15 | 3 | 4 | 0.0380833 | 0.8166322 | 0.9476756 | 0.0401860 |
| NC\_012920 | 7772 | 0.0000000 | 0.0000000 | 0.1244444 | 0.0000000 | 0.0000000 | 0.0000000 | 0.0000000 | 0.0000000 | 0.000 | 0.0075471 | 86 | 18 | 15 | 39 | 43 | 41 | 15 | 3 | 4 | 0.0631179 | 0.8103478 | 0.9912745 | 0.0636735 |
| NC\_012920 | 7780 | 0.0229854 | 0.0000000 | 0.0000000 | 0.0000000 | 0.0000000 | 0.0000000 | 0.0000000 | 0.0000000 | 0.000 | 0.0075471 | 86 | 18 | 15 | 39 | 43 | 41 | 15 | 3 | 4 | 0.0078698 | 0.8123852 | 0.9907831 | 0.0079430 |
| NC\_012920 | 7785 | 0.0229854 | 0.0000000 | 0.0000000 | 0.0000000 | 0.0000000 | 0.0000000 | 0.0000000 | 0.0000000 | 0.000 | 0.0075471 | 86 | 18 | 15 | 39 | 43 | 41 | 15 | 3 | 4 | 0.0078698 | 0.8123852 | 0.9907831 | 0.0079430 |
| NC\_012920 | 7789 | 0.0000000 | 0.1049383 | 0.0000000 | 0.0973044 | 0.0000000 | 0.0928019 | 0.0000000 | 0.0000000 | 0.000 | 0.0371614 | 86 | 18 | 15 | 39 | 43 | 41 | 15 | 3 | 4 | 0.0328183 | 0.8147957 | 0.9558886 | 0.0343328 |
| NC\_012920 | 7805 | 0.0673337 | 0.0000000 | 0.0000000 | 0.0499671 | 0.0000000 | 0.0000000 | 0.0000000 | 0.0000000 | 0.000 | 0.0298439 | 86 | 18 | 15 | 39 | 43 | 41 | 15 | 3 | 4 | 0.0176896 | 0.8181818 | 0.9641694 | 0.0183469 |
| NC\_012920 | 7819 | 0.0000000 | 0.0000000 | 0.0000000 | 0.0000000 | 0.0000000 | 0.0475907 | 0.0000000 | 0.0000000 | 0.000 | 0.0075471 | 86 | 18 | 15 | 39 | 43 | 41 | 15 | 3 | 4 | 0.0206807 | 0.8110939 | 0.9908876 | 0.0208709 |
| NC\_012920 | 7837 | 0.0229854 | 0.0000000 | 0.0000000 | 0.0000000 | 0.0000000 | 0.0000000 | 0.0000000 | 0.0000000 | 0.000 | 0.0075471 | 86 | 18 | 15 | 39 | 43 | 41 | 15 | 3 | 4 | 0.0078698 | 0.8123852 | 0.9907831 | 0.0079430 |
| NC\_012920 | 7853 | 0.0229854 | 0.0000000 | 0.0000000 | 0.0000000 | 0.0000000 | 0.0000000 | 0.0000000 | 0.0000000 | 0.000 | 0.0075471 | 86 | 18 | 15 | 39 | 43 | 41 | 15 | 3 | 4 | 0.0078698 | 0.8123852 | 0.9907831 | 0.0079430 |
| NC\_012920 | 7859 | 0.0229854 | 0.0000000 | 0.0000000 | 0.0000000 | 0.0454300 | 0.0475907 | 0.0000000 | 0.0000000 | 0.000 | 0.0224690 | 86 | 18 | 15 | 39 | 43 | 41 | 15 | 3 | 4 | 0.0084908 | 0.8147383 | 0.9726560 | 0.0087295 |
| NC\_012920 | 7961 | 0.0000000 | 0.0000000 | 0.0000000 | 0.0499671 | 0.0000000 | 0.0000000 | 0.0000000 | 0.0000000 | 0.000 | 0.0075471 | 86 | 18 | 15 | 39 | 43 | 41 | 15 | 3 | 4 | 0.0219362 | 0.8110365 | 0.9908987 | 0.0221377 |
| NC\_012920 | 7979 | 0.0000000 | 0.0000000 | 0.0000000 | 0.0000000 | 0.0454300 | 0.0000000 | 0.0000000 | 0.0000000 | 0.000 | 0.0075471 | 86 | 18 | 15 | 39 | 43 | 41 | 15 | 3 | 4 | 0.0195420 | 0.8111513 | 0.9908777 | 0.0197219 |
| NC\_012920 | 8020 | 0.0000000 | 0.0000000 | 0.0000000 | 0.0000000 | 0.0454300 | 0.0000000 | 0.0000000 | 0.0000000 | 0.000 | 0.0075471 | 86 | 18 | 15 | 39 | 43 | 41 | 15 | 3 | 4 | 0.0195420 | 0.8111513 | 0.9908777 | 0.0197219 |
| NC\_012920 | 8023 | 0.0229854 | 0.0000000 | 0.0000000 | 0.0000000 | 0.0454300 | 0.0000000 | 0.0000000 | 0.0000000 | 0.000 | 0.0150367 | 86 | 18 | 15 | 39 | 43 | 41 | 15 | 3 | 4 | 0.0099414 | 0.8135904 | 0.9817018 | 0.0101267 |
| NC\_012920 | 8027 | 0.0000000 | 0.0000000 | 0.0000000 | 0.0499671 | 0.0000000 | 0.0928019 | 0.0000000 | 0.0000000 | 0.000 | 0.0224690 | 86 | 18 | 15 | 39 | 43 | 41 | 15 | 3 | 4 | 0.0300451 | 0.8132748 | 0.9732023 | 0.0308724 |
| NC\_012920 | 8104 | 0.0229854 | 0.0000000 | 0.0000000 | 0.0000000 | 0.0000000 | 0.0000000 | 0.3200000 | 0.0000000 | 0.000 | 0.0298439 | 86 | 18 | 15 | 39 | 43 | 41 | 15 | 3 | 4 | 0.1398748 | 0.8134183 | 0.9684425 | 0.1444327 |
| NC\_012920 | 8110 | 0.0229854 | 0.0000000 | 0.0000000 | 0.0000000 | 0.0000000 | 0.0475907 | 0.0000000 | 0.0000000 | 0.000 | 0.0150367 | 86 | 18 | 15 | 39 | 43 | 41 | 15 | 3 | 4 | 0.0105130 | 0.8135331 | 0.9817111 | 0.0107088 |
| NC\_012920 | 8137 | 0.0000000 | 0.0000000 | 0.0000000 | 0.0000000 | 0.0886966 | 0.0475907 | 0.0000000 | 0.0000000 | 0.375 | 0.0298439 | 86 | 18 | 15 | 39 | 43 | 41 | 15 | 3 | 4 | 0.0778819 | 0.8135331 | 0.9661727 | 0.0806087 |
| NC\_012920 | 8143 | 0.0229854 | 0.0000000 | 0.2311111 | 0.0000000 | 0.0000000 | 0.0000000 | 0.0000000 | 0.4444444 | 0.000 | 0.0298439 | 86 | 18 | 15 | 39 | 43 | 41 | 15 | 3 | 4 | 0.1398748 | 0.8131887 | 0.9684335 | 0.1444340 |
| NC\_012920 | 8152 | 0.0229854 | 0.0000000 | 0.0000000 | 0.0000000 | 0.0000000 | 0.0000000 | 0.0000000 | 0.0000000 | 0.000 | 0.0075471 | 86 | 18 | 15 | 39 | 43 | 41 | 15 | 3 | 4 | 0.0078698 | 0.8123852 | 0.9907831 | 0.0079430 |
| NC\_012920 | 8155 | 0.0454300 | 0.2777778 | 0.1244444 | 0.0000000 | 0.0454300 | 0.0475907 | 0.0000000 | 0.0000000 | 0.000 | 0.0587695 | 86 | 18 | 15 | 39 | 43 | 41 | 15 | 3 | 4 | 0.0539344 | 0.8188131 | 0.9320971 | 0.0578635 |
| NC\_012920 | 8206 | 0.0454300 | 0.1975309 | 0.0000000 | 0.0973044 | 0.0454300 | 0.0000000 | 0.1244444 | 0.0000000 | 0.000 | 0.0585546 | 86 | 18 | 15 | 39 | 43 | 42 | 15 | 3 | 4 | 0.0283188 | 0.8195942 | 0.9305797 | 0.0304313 |
| NC\_012920 | 8222 | 0.0229854 | 0.0000000 | 0.0000000 | 0.0000000 | 0.0000000 | 0.0000000 | 0.0000000 | 0.0000000 | 0.375 | 0.0149804 | 86 | 18 | 15 | 39 | 43 | 42 | 15 | 3 | 4 | 0.1242042 | 0.8127020 | 0.9838566 | 0.1262422 |
| NC\_012920 | 8251 | 0.1687399 | 0.1049383 | 0.1244444 | 0.0499671 | 0.0886966 | 0.0907029 | 0.0000000 | 0.0000000 | 0.000 | 0.1067996 | 86 | 18 | 15 | 39 | 43 | 42 | 15 | 3 | 4 | 0.0163420 | 0.8345461 | 0.8741181 | 0.0186954 |
| NC\_012920 | 8269 | 0.1495403 | 0.2777778 | 0.0000000 | 0.1840894 | 0.0000000 | 0.0464853 | 0.2311111 | 0.0000000 | 0.375 | 0.1266216 | 86 | 18 | 15 | 39 | 43 | 42 | 15 | 3 | 4 | 0.0475557 | 0.8332075 | 0.8552582 | 0.0556039 |
| NC\_012920 | 8292 | 0.1095187 | 0.0000000 | 0.1244444 | 0.1840894 | 0.0000000 | 0.0464853 | 0.3911111 | 0.4444444 | 0.000 | 0.1134639 | 86 | 18 | 15 | 39 | 43 | 42 | 15 | 3 | 4 | 0.0815083 | 0.8285938 | 0.8742259 | 0.0932348 |
| NC\_012920 | 8349 | 0.0000000 | 0.0000000 | 0.0000000 | 0.0000000 | 0.0000000 | 0.0464853 | 0.0000000 | 0.0000000 | 0.000 | 0.0075187 | 86 | 18 | 15 | 39 | 43 | 42 | 15 | 3 | 4 | 0.0201118 | 0.8113635 | 0.9909196 | 0.0202961 |
| NC\_012920 | 8382 | 0.0000000 | 0.0000000 | 0.0000000 | 0.0499671 | 0.0000000 | 0.0000000 | 0.0000000 | 0.0000000 | 0.000 | 0.0075187 | 86 | 18 | 15 | 39 | 43 | 42 | 15 | 3 | 4 | 0.0219503 | 0.8112780 | 0.9909357 | 0.0221511 |
| NC\_012920 | 8386 | 0.0229854 | 0.0000000 | 0.0000000 | 0.0000000 | 0.0000000 | 0.0000000 | 0.0000000 | 0.0000000 | 0.000 | 0.0075187 | 86 | 18 | 15 | 39 | 43 | 42 | 15 | 3 | 4 | 0.0078841 | 0.8126166 | 0.9908205 | 0.0079571 |
| NC\_012920 | 8388 | 0.0000000 | 0.1049383 | 0.0000000 | 0.0000000 | 0.0000000 | 0.0000000 | 0.0000000 | 0.0000000 | 0.000 | 0.0075187 | 86 | 18 | 15 | 39 | 43 | 42 | 15 | 3 | 4 | 0.0519781 | 0.8106800 | 0.9912075 | 0.0524392 |
| NC\_012920 | 8393 | 0.0229854 | 0.0000000 | 0.0000000 | 0.0000000 | 0.0000000 | 0.0000000 | 0.0000000 | 0.0000000 | 0.000 | 0.0075187 | 86 | 18 | 15 | 39 | 43 | 42 | 15 | 3 | 4 | 0.0078841 | 0.8126166 | 0.9908205 | 0.0079571 |
| NC\_012920 | 8395 | 0.0229854 | 0.0000000 | 0.0000000 | 0.0000000 | 0.0000000 | 0.0000000 | 0.0000000 | 0.0000000 | 0.000 | 0.0075187 | 86 | 18 | 15 | 39 | 43 | 42 | 15 | 3 | 4 | 0.0078841 | 0.8126166 | 0.9908205 | 0.0079571 |
| NC\_012920 | 8396 | 0.0000000 | 0.0000000 | 0.1244444 | 0.0499671 | 0.0000000 | 0.0000000 | 0.0000000 | 0.0000000 | 0.000 | 0.0149804 | 86 | 18 | 15 | 39 | 43 | 42 | 15 | 3 | 4 | 0.0389003 | 0.8116768 | 0.9822618 | 0.0396027 |
| NC\_012920 | 8428 | 0.0229854 | 0.0000000 | 0.0000000 | 0.0000000 | 0.0454300 | 0.0000000 | 0.0000000 | 0.0000000 | 0.000 | 0.0149804 | 86 | 18 | 15 | 39 | 43 | 42 | 15 | 3 | 4 | 0.0099699 | 0.8138127 | 0.9817758 | 0.0101550 |
| NC\_012920 | 8435 | 0.0229854 | 0.0000000 | 0.0000000 | 0.0000000 | 0.0000000 | 0.0000000 | 0.0000000 | 0.0000000 | 0.000 | 0.0075187 | 86 | 18 | 15 | 39 | 43 | 42 | 15 | 3 | 4 | 0.0078841 | 0.8126166 | 0.9908205 | 0.0079571 |
| NC\_012920 | 8460 | 0.0229854 | 0.0000000 | 0.0000000 | 0.0000000 | 0.0454300 | 0.0000000 | 0.0000000 | 0.0000000 | 0.000 | 0.0149804 | 86 | 18 | 15 | 39 | 43 | 42 | 15 | 3 | 4 | 0.0099699 | 0.8138127 | 0.9817758 | 0.0101550 |
| NC\_012920 | 8462 | 0.0000000 | 0.0000000 | 0.0000000 | 0.0000000 | 0.0000000 | 0.0464853 | 0.0000000 | 0.0000000 | 0.000 | 0.0075187 | 86 | 18 | 15 | 39 | 43 | 42 | 15 | 3 | 4 | 0.0201118 | 0.8113635 | 0.9909196 | 0.0202961 |
| NC\_012920 | 8468 | 0.0454300 | 0.0000000 | 0.0000000 | 0.0499671 | 0.0454300 | 0.0464853 | 0.0000000 | 0.0000000 | 0.000 | 0.0370239 | 86 | 18 | 15 | 39 | 43 | 42 | 15 | 3 | 4 | 0.0050714 | 0.8184265 | 0.9549916 | 0.0053104 |
| NC\_012920 | 8472 | 0.0229854 | 0.0000000 | 0.0000000 | 0.0000000 | 0.0454300 | 0.0000000 | 0.0000000 | 0.0000000 | 0.000 | 0.0149804 | 86 | 18 | 15 | 39 | 43 | 42 | 15 | 3 | 4 | 0.0099699 | 0.8138127 | 0.9817758 | 0.0101550 |
| NC\_012920 | 8473 | 0.0454300 | 0.0000000 | 0.2311111 | 0.0000000 | 0.0000000 | 0.0464853 | 0.0000000 | 0.4444444 | 0.000 | 0.0442577 | 86 | 18 | 15 | 39 | 43 | 42 | 15 | 3 | 4 | 0.0911423 | 0.8169455 | 0.9507629 | 0.0958623 |
| NC\_012920 | 8475 | 0.0229854 | 0.0000000 | 0.0000000 | 0.0000000 | 0.0000000 | 0.0464853 | 0.0000000 | 0.0000000 | 0.000 | 0.0149804 | 86 | 18 | 15 | 39 | 43 | 42 | 15 | 3 | 4 | 0.0102489 | 0.8137843 | 0.9817803 | 0.0104391 |
| NC\_012920 | 8494 | 0.0000000 | 0.0000000 | 0.0000000 | 0.0000000 | 0.0000000 | 0.0000000 | 0.1244444 | 0.0000000 | 0.000 | 0.0075187 | 86 | 18 | 15 | 39 | 43 | 42 | 15 | 3 | 4 | 0.0631313 | 0.8105945 | 0.9913101 | 0.0636847 |
| NC\_012920 | 8502 | 0.0000000 | 0.0000000 | 0.0000000 | 0.0499671 | 0.0000000 | 0.0000000 | 0.0000000 | 0.0000000 | 0.000 | 0.0075187 | 86 | 18 | 15 | 39 | 43 | 42 | 15 | 3 | 4 | 0.0219503 | 0.8112780 | 0.9909357 | 0.0221511 |
| NC\_012920 | 8527 | 0.0000000 | 0.0000000 | 0.0000000 | 0.0000000 | 0.0000000 | 0.0907029 | 0.0000000 | 0.0000000 | 0.000 | 0.0149804 | 86 | 18 | 15 | 39 | 43 | 42 | 15 | 3 | 4 | 0.0403766 | 0.8124742 | 0.9823064 | 0.0411039 |
| NC\_012920 | 8566 | 0.0454300 | 0.0000000 | 0.0000000 | 0.0000000 | 0.0454300 | 0.0000000 | 0.0000000 | 0.0000000 | 0.000 | 0.0223852 | 86 | 18 | 15 | 39 | 43 | 42 | 15 | 3 | 4 | 0.0120717 | 0.8161766 | 0.9729042 | 0.0124079 |
| NC\_012920 | 8573 | 0.0000000 | 0.0000000 | 0.1244444 | 0.0000000 | 0.0000000 | 0.0000000 | 0.0000000 | 0.0000000 | 0.000 | 0.0075187 | 86 | 18 | 15 | 39 | 43 | 42 | 15 | 3 | 4 | 0.0631313 | 0.8105945 | 0.9913101 | 0.0636847 |
| NC\_012920 | 8584 | 0.0229854 | 0.0000000 | 0.0000000 | 0.0000000 | 0.0454300 | 0.0000000 | 0.0000000 | 0.0000000 | 0.000 | 0.0149804 | 86 | 18 | 15 | 39 | 43 | 42 | 15 | 3 | 4 | 0.0099699 | 0.8138127 | 0.9817758 | 0.0101550 |
| NC\_012920 | 8594 | 0.0000000 | 0.0000000 | 0.0000000 | 0.0000000 | 0.0454300 | 0.0000000 | 0.0000000 | 0.0000000 | 0.375 | 0.0149804 | 86 | 18 | 15 | 39 | 43 | 42 | 15 | 3 | 4 | 0.1300623 | 0.8114774 | 0.9839404 | 0.1321852 |
| NC\_012920 | 8614 | 0.0000000 | 0.0000000 | 0.0000000 | 0.0000000 | 0.0000000 | 0.0907029 | 0.0000000 | 0.0000000 | 0.000 | 0.0149804 | 86 | 18 | 15 | 39 | 43 | 42 | 15 | 3 | 4 | 0.0403766 | 0.8124742 | 0.9823064 | 0.0411039 |
| NC\_012920 | 8618 | 0.0000000 | 0.0000000 | 0.0000000 | 0.0499671 | 0.0454300 | 0.0000000 | 0.0000000 | 0.0000000 | 0.000 | 0.0149804 | 86 | 18 | 15 | 39 | 43 | 42 | 15 | 3 | 4 | 0.0170298 | 0.8124742 | 0.9818760 | 0.0173441 |
| NC\_012920 | 8655 | 0.0454300 | 0.0000000 | 0.0000000 | 0.0499671 | 0.0454300 | 0.0907029 | 0.0000000 | 0.0000000 | 0.000 | 0.0442577 | 86 | 18 | 15 | 39 | 43 | 42 | 15 | 3 | 4 | 0.0093445 | 0.8195372 | 0.9465013 | 0.0098727 |
| NC\_012920 | 8664 | 0.0229854 | 0.0000000 | 0.0000000 | 0.0000000 | 0.0000000 | 0.0000000 | 0.0000000 | 0.0000000 | 0.000 | 0.0075187 | 86 | 18 | 15 | 39 | 43 | 42 | 15 | 3 | 4 | 0.0078841 | 0.8126166 | 0.9908205 | 0.0079571 |
| NC\_012920 | 8676 | 0.0229854 | 0.0000000 | 0.0000000 | 0.0000000 | 0.0454300 | 0.0000000 | 0.0000000 | 0.0000000 | 0.000 | 0.0149804 | 86 | 18 | 15 | 39 | 43 | 42 | 15 | 3 | 4 | 0.0099699 | 0.8138127 | 0.9817758 | 0.0101550 |
| NC\_012920 | 8684 | 0.0000000 | 0.0000000 | 0.0000000 | 0.0000000 | 0.0886966 | 0.0464853 | 0.0000000 | 0.0000000 | 0.375 | 0.0297330 | 86 | 18 | 15 | 39 | 43 | 42 | 15 | 3 | 4 | 0.0777880 | 0.8137843 | 0.9663054 | 0.0805004 |
| NC\_012920 | 8697 | 0.1687399 | 0.1975309 | 0.2311111 | 0.0000000 | 0.0886966 | 0.0464853 | 0.1244444 | 0.0000000 | 0.000 | 0.1134639 | 86 | 18 | 15 | 39 | 43 | 42 | 15 | 3 | 4 | 0.0299679 | 0.8335208 | 0.8679534 | 0.0345271 |
| NC\_012920 | 8701 | 0.2401298 | 0.2777778 | 0.3200000 | 0.3550296 | 0.4672796 | 0.3367347 | 0.3911111 | 0.0000000 | 0.000 | 0.3333286 | 86 | 18 | 15 | 39 | 43 | 42 | 15 | 3 | 4 | 0.0445162 | 0.8674973 | 0.6328634 | 0.0703410 |
| NC\_012920 | 8705 | 0.0000000 | 0.0000000 | 0.0000000 | 0.0000000 | 0.0000000 | 0.0464853 | 0.0000000 | 0.0000000 | 0.000 | 0.0075187 | 86 | 18 | 15 | 39 | 43 | 42 | 15 | 3 | 4 | 0.0201118 | 0.8113635 | 0.9909196 | 0.0202961 |
| NC\_012920 | 8712 | 0.0454300 | 0.0000000 | 0.0000000 | 0.0000000 | 0.0000000 | 0.0000000 | 0.0000000 | 0.0000000 | 0.000 | 0.0149804 | 86 | 18 | 15 | 39 | 43 | 42 | 15 | 3 | 4 | 0.0158281 | 0.8149804 | 0.9819096 | 0.0161197 |
| NC\_012920 | 8730 | 0.0000000 | 0.1049383 | 0.0000000 | 0.0000000 | 0.0000000 | 0.0464853 | 0.0000000 | 0.0000000 | 0.000 | 0.0149804 | 86 | 18 | 15 | 39 | 43 | 42 | 15 | 3 | 4 | 0.0323797 | 0.8118476 | 0.9821452 | 0.0329684 |
| NC\_012920 | 8772 | 0.0229854 | 0.0000000 | 0.0000000 | 0.0000000 | 0.0000000 | 0.0000000 | 0.0000000 | 0.0000000 | 0.000 | 0.0075187 | 86 | 18 | 15 | 39 | 43 | 42 | 15 | 3 | 4 | 0.0078841 | 0.8126166 | 0.9908205 | 0.0079571 |
| NC\_012920 | 8805 | 0.0000000 | 0.0000000 | 0.0000000 | 0.0000000 | 0.0454300 | 0.0000000 | 0.0000000 | 0.0000000 | 0.000 | 0.0075187 | 86 | 18 | 15 | 39 | 43 | 42 | 15 | 3 | 4 | 0.0195560 | 0.8113920 | 0.9909148 | 0.0197353 |
| NC\_012920 | 8812 | 0.0000000 | 0.0000000 | 0.0000000 | 0.0000000 | 0.0454300 | 0.0000000 | 0.0000000 | 0.0000000 | 0.000 | 0.0075187 | 86 | 18 | 15 | 39 | 43 | 42 | 15 | 3 | 4 | 0.0195560 | 0.8113920 | 0.9909148 | 0.0197353 |
| NC\_012920 | 8814 | 0.0000000 | 0.0000000 | 0.0000000 | 0.0000000 | 0.0000000 | 0.0464853 | 0.0000000 | 0.0000000 | 0.000 | 0.0075187 | 86 | 18 | 15 | 39 | 43 | 42 | 15 | 3 | 4 | 0.0201118 | 0.8113635 | 0.9909196 | 0.0202961 |
| NC\_012920 | 8817 | 0.0229854 | 0.0000000 | 0.0000000 | 0.0000000 | 0.0000000 | 0.0000000 | 0.0000000 | 0.0000000 | 0.000 | 0.0075187 | 86 | 18 | 15 | 39 | 43 | 42 | 15 | 3 | 4 | 0.0078841 | 0.8126166 | 0.9908205 | 0.0079571 |
| NC\_012920 | 8836 | 0.0229854 | 0.0000000 | 0.0000000 | 0.0000000 | 0.0454300 | 0.0000000 | 0.0000000 | 0.0000000 | 0.000 | 0.0149804 | 86 | 18 | 15 | 39 | 43 | 42 | 15 | 3 | 4 | 0.0099699 | 0.8138127 | 0.9817758 | 0.0101550 |
| NC\_012920 | 8843 | 0.0000000 | 0.0000000 | 0.1244444 | 0.0000000 | 0.0454300 | 0.0464853 | 0.0000000 | 0.0000000 | 0.000 | 0.0223852 | 86 | 18 | 15 | 39 | 43 | 42 | 15 | 3 | 4 | 0.0268944 | 0.8129583 | 0.9732051 | 0.0276349 |
| NC\_012920 | 8856 | 0.0000000 | 0.0000000 | 0.0000000 | 0.0000000 | 0.0000000 | 0.0000000 | 0.1244444 | 0.0000000 | 0.000 | 0.0075187 | 86 | 18 | 15 | 39 | 43 | 42 | 15 | 3 | 4 | 0.0631313 | 0.8105945 | 0.9913101 | 0.0636847 |
| NC\_012920 | 8860 | 0.0000000 | 0.0000000 | 0.0000000 | 0.0000000 | 0.0000000 | 0.0000000 | 0.0000000 | 0.0000000 | 0.000 | 0.0000000 | 86 | 18 | 15 | 39 | 43 | 42 | 15 | 3 | 4 | NaN | 0.8101958 | 1.0000000 | NaN |
| NC\_012920 | 8865 | 0.0000000 | 0.0000000 | 0.0000000 | 0.0000000 | 0.0000000 | 0.0464853 | 0.0000000 | 0.0000000 | 0.000 | 0.0075187 | 86 | 18 | 15 | 39 | 43 | 42 | 15 | 3 | 4 | 0.0201118 | 0.8113635 | 0.9909196 | 0.0202961 |
| NC\_012920 | 8870 | 0.0000000 | 0.0000000 | 0.0000000 | 0.0000000 | 0.0000000 | 0.0464853 | 0.0000000 | 0.0000000 | 0.000 | 0.0075187 | 86 | 18 | 15 | 39 | 43 | 42 | 15 | 3 | 4 | 0.0201118 | 0.8113635 | 0.9909196 | 0.0202961 |
| NC\_012920 | 8895 | 0.0000000 | 0.1049383 | 0.0000000 | 0.0000000 | 0.0000000 | 0.0000000 | 0.0000000 | 0.0000000 | 0.000 | 0.0075187 | 86 | 18 | 15 | 39 | 43 | 42 | 15 | 3 | 4 | 0.0519781 | 0.8106800 | 0.9912075 | 0.0524392 |
| NC\_012920 | 8901 | 0.0000000 | 0.0000000 | 0.1244444 | 0.0000000 | 0.0000000 | 0.0000000 | 0.0000000 | 0.0000000 | 0.000 | 0.0075187 | 86 | 18 | 15 | 39 | 43 | 42 | 15 | 3 | 4 | 0.0631313 | 0.8105945 | 0.9913101 | 0.0636847 |
| NC\_012920 | 8932 | 0.0000000 | 0.0000000 | 0.0000000 | 0.0000000 | 0.0000000 | 0.0907029 | 0.0000000 | 0.0000000 | 0.000 | 0.0149804 | 86 | 18 | 15 | 39 | 43 | 42 | 15 | 3 | 4 | 0.0403766 | 0.8124742 | 0.9823064 | 0.0411039 |
| NC\_012920 | 8945 | 0.0229854 | 0.0000000 | 0.0000000 | 0.0000000 | 0.0000000 | 0.0000000 | 0.0000000 | 0.0000000 | 0.000 | 0.0075187 | 86 | 18 | 15 | 39 | 43 | 42 | 15 | 3 | 4 | 0.0078841 | 0.8126166 | 0.9908205 | 0.0079571 |
| NC\_012920 | 8994 | 0.0000000 | 0.0000000 | 0.0000000 | 0.0499671 | 0.0000000 | 0.0907029 | 0.0000000 | 0.0000000 | 0.000 | 0.0223852 | 86 | 18 | 15 | 39 | 43 | 42 | 15 | 3 | 4 | 0.0293040 | 0.8135564 | 0.9732911 | 0.0301082 |
| NC\_012920 | 9007 | 0.0229854 | 0.0000000 | 0.0000000 | 0.0000000 | 0.0000000 | 0.0000000 | 0.0000000 | 0.0000000 | 0.000 | 0.0075187 | 86 | 18 | 15 | 39 | 43 | 42 | 15 | 3 | 4 | 0.0078841 | 0.8126166 | 0.9908205 | 0.0079571 |
| NC\_012920 | 9025 | 0.0000000 | 0.0000000 | 0.0000000 | 0.0000000 | 0.0454300 | 0.0000000 | 0.0000000 | 0.0000000 | 0.000 | 0.0075187 | 86 | 18 | 15 | 39 | 43 | 42 | 15 | 3 | 4 | 0.0195560 | 0.8113920 | 0.9909148 | 0.0197353 |
| NC\_012920 | 9042 | 0.0454300 | 0.0000000 | 0.0000000 | 0.0000000 | 0.0454300 | 0.0000000 | 0.0000000 | 0.0000000 | 0.000 | 0.0223852 | 86 | 18 | 15 | 39 | 43 | 42 | 15 | 3 | 4 | 0.0120717 | 0.8161766 | 0.9729042 | 0.0124079 |
| NC\_012920 | 9048 | 0.0000000 | 0.0000000 | 0.0000000 | 0.0000000 | 0.0454300 | 0.0000000 | 0.0000000 | 0.0000000 | 0.000 | 0.0075187 | 86 | 18 | 15 | 39 | 43 | 42 | 15 | 3 | 4 | 0.0195560 | 0.8113920 | 0.9909148 | 0.0197353 |
| NC\_012920 | 9055 | 0.0886966 | 0.0000000 | 0.0000000 | 0.4260355 | 0.3309897 | 0.0000000 | 0.1244444 | 0.0000000 | 0.000 | 0.1769740 | 86 | 18 | 15 | 39 | 43 | 42 | 15 | 3 | 4 | 0.1397838 | 0.8378782 | 0.8183078 | 0.1708206 |
| NC\_012920 | 9061 | 0.0229854 | 0.0000000 | 0.0000000 | 0.0000000 | 0.0886966 | 0.0000000 | 0.0000000 | 0.0000000 | 0.000 | 0.0223852 | 86 | 18 | 15 | 39 | 43 | 42 | 15 | 3 | 4 | 0.0238328 | 0.8149519 | 0.9731865 | 0.0244894 |
| NC\_012920 | 9067 | 0.0000000 | 0.0000000 | 0.0000000 | 0.0000000 | 0.0454300 | 0.0000000 | 0.0000000 | 0.0000000 | 0.000 | 0.0075187 | 86 | 18 | 15 | 39 | 43 | 42 | 15 | 3 | 4 | 0.0195560 | 0.8113920 | 0.9909148 | 0.0197353 |
| NC\_012920 | 9070 | 0.0000000 | 0.0000000 | 0.0000000 | 0.0000000 | 0.0000000 | 0.0000000 | 0.1244444 | 0.0000000 | 0.000 | 0.0075187 | 86 | 18 | 15 | 39 | 43 | 42 | 15 | 3 | 4 | 0.0631313 | 0.8105945 | 0.9913101 | 0.0636847 |
| NC\_012920 | 9072 | 0.0000000 | 0.0000000 | 0.0000000 | 0.0499671 | 0.0000000 | 0.0464853 | 0.0000000 | 0.0000000 | 0.000 | 0.0149804 | 86 | 18 | 15 | 39 | 43 | 42 | 15 | 3 | 4 | 0.0173087 | 0.8124457 | 0.9818805 | 0.0176282 |
| NC\_012920 | 9083 | 0.0000000 | 0.0000000 | 0.0000000 | 0.0000000 | 0.0454300 | 0.0000000 | 0.0000000 | 0.0000000 | 0.000 | 0.0075187 | 86 | 18 | 15 | 39 | 43 | 42 | 15 | 3 | 4 | 0.0195560 | 0.8113920 | 0.9909148 | 0.0197353 |
| NC\_012920 | 9088 | 0.0000000 | 0.0000000 | 0.0000000 | 0.0499671 | 0.0000000 | 0.0000000 | 0.0000000 | 0.0000000 | 0.000 | 0.0075187 | 86 | 18 | 15 | 39 | 43 | 42 | 15 | 3 | 4 | 0.0219503 | 0.8112780 | 0.9909357 | 0.0221511 |
| NC\_012920 | 9094 | 0.0000000 | 0.1049383 | 0.0000000 | 0.0499671 | 0.0000000 | 0.0464853 | 0.0000000 | 0.0000000 | 0.000 | 0.0223852 | 86 | 18 | 15 | 39 | 43 | 42 | 15 | 3 | 4 | 0.0239524 | 0.8129299 | 0.9731231 | 0.0246140 |
| NC\_012920 | 9101 | 0.0454300 | 0.1975309 | 0.1244444 | 0.0000000 | 0.0454300 | 0.0464853 | 0.0000000 | 0.0000000 | 0.000 | 0.0514347 | 86 | 18 | 15 | 39 | 43 | 42 | 15 | 3 | 4 | 0.0289886 | 0.8186543 | 0.9389930 | 0.0308720 |
| NC\_012920 | 9103 | 0.0229854 | 0.0000000 | 0.0000000 | 0.0000000 | 0.0000000 | 0.0000000 | 0.0000000 | 0.0000000 | 0.000 | 0.0075187 | 86 | 18 | 15 | 39 | 43 | 42 | 15 | 3 | 4 | 0.0078841 | 0.8126166 | 0.9908205 | 0.0079571 |
| NC\_012920 | 9116 | 0.0454300 | 0.0000000 | 0.0000000 | 0.0499671 | 0.0000000 | 0.0464853 | 0.0000000 | 0.0000000 | 0.000 | 0.0297330 | 86 | 18 | 15 | 39 | 43 | 42 | 15 | 3 | 4 | 0.0090325 | 0.8172303 | 0.9639460 | 0.0093704 |
| NC\_012920 | 9123 | 0.0673337 | 0.0000000 | 0.0000000 | 0.0000000 | 0.0454300 | 0.0000000 | 0.1244444 | 0.0000000 | 0.000 | 0.0370239 | 86 | 18 | 15 | 39 | 43 | 42 | 15 | 3 | 4 | 0.0204323 | 0.8188822 | 0.9557111 | 0.0213792 |
| NC\_012920 | 9126 | 0.0229854 | 0.0000000 | 0.0000000 | 0.0000000 | 0.0000000 | 0.0000000 | 0.0000000 | 0.0000000 | 0.000 | 0.0075187 | 86 | 18 | 15 | 39 | 43 | 42 | 15 | 3 | 4 | 0.0078841 | 0.8126166 | 0.9908205 | 0.0079571 |
| NC\_012920 | 9139 | 0.0229854 | 0.0000000 | 0.0000000 | 0.0000000 | 0.0000000 | 0.0000000 | 0.0000000 | 0.0000000 | 0.000 | 0.0075187 | 86 | 18 | 15 | 39 | 43 | 42 | 15 | 3 | 4 | 0.0078841 | 0.8126166 | 0.9908205 | 0.0079571 |
| NC\_012920 | 9156 | 0.0229854 | 0.0000000 | 0.0000000 | 0.0000000 | 0.0000000 | 0.0000000 | 0.0000000 | 0.0000000 | 0.000 | 0.0075187 | 86 | 18 | 15 | 39 | 43 | 42 | 15 | 3 | 4 | 0.0078841 | 0.8126166 | 0.9908205 | 0.0079571 |
| NC\_012920 | 9165 | 0.0454300 | 0.0000000 | 0.0000000 | 0.0000000 | 0.0000000 | 0.0000000 | 0.0000000 | 0.0000000 | 0.000 | 0.0149804 | 86 | 18 | 15 | 39 | 43 | 42 | 15 | 3 | 4 | 0.0158281 | 0.8149804 | 0.9819096 | 0.0161197 |
| NC\_012920 | 9174 | 0.0000000 | 0.0000000 | 0.0000000 | 0.0000000 | 0.0454300 | 0.0000000 | 0.0000000 | 0.0000000 | 0.000 | 0.0075187 | 86 | 18 | 15 | 39 | 43 | 42 | 15 | 3 | 4 | 0.0195560 | 0.8113920 | 0.9909148 | 0.0197353 |
| NC\_012920 | 9177 | 0.0886966 | 0.0000000 | 0.0000000 | 0.0000000 | 0.0000000 | 0.0000000 | 0.0000000 | 0.0000000 | 0.000 | 0.0297330 | 86 | 18 | 15 | 39 | 43 | 42 | 15 | 3 | 4 | 0.0318988 | 0.8195372 | 0.9648771 | 0.0330599 |
| NC\_012920 | 9221 | 0.0454300 | 0.1975309 | 0.0000000 | 0.0973044 | 0.0454300 | 0.0000000 | 0.1244444 | 0.0000000 | 0.000 | 0.0585546 | 86 | 18 | 15 | 39 | 43 | 42 | 15 | 3 | 4 | 0.0283188 | 0.8195942 | 0.9305797 | 0.0304313 |
| NC\_012920 | 9242 | 0.0229854 | 0.0000000 | 0.0000000 | 0.0000000 | 0.0000000 | 0.0000000 | 0.0000000 | 0.0000000 | 0.000 | 0.0075187 | 86 | 18 | 15 | 39 | 43 | 42 | 15 | 3 | 4 | 0.0078841 | 0.8126166 | 0.9908205 | 0.0079571 |
| NC\_012920 | 9245 | 0.0000000 | 0.0000000 | 0.0000000 | 0.0499671 | 0.0000000 | 0.0000000 | 0.0000000 | 0.0000000 | 0.000 | 0.0075187 | 86 | 18 | 15 | 39 | 43 | 42 | 15 | 3 | 4 | 0.0219503 | 0.8112780 | 0.9909357 | 0.0221511 |
| NC\_012920 | 9254 | 0.0229854 | 0.0000000 | 0.0000000 | 0.0499671 | 0.0000000 | 0.0464853 | 0.0000000 | 0.0000000 | 0.000 | 0.0223852 | 86 | 18 | 15 | 39 | 43 | 42 | 15 | 3 | 4 | 0.0091422 | 0.8148665 | 0.9727802 | 0.0093980 |
| NC\_012920 | 9266 | 0.0454300 | 0.0000000 | 0.0000000 | 0.0499671 | 0.0000000 | 0.0000000 | 0.0000000 | 0.0000000 | 0.000 | 0.0223852 | 86 | 18 | 15 | 39 | 43 | 42 | 15 | 3 | 4 | 0.0128759 | 0.8160627 | 0.9729225 | 0.0132342 |
| NC\_012920 | 9287 | 0.0229854 | 0.0000000 | 0.0000000 | 0.0000000 | 0.0000000 | 0.0000000 | 0.0000000 | 0.0000000 | 0.000 | 0.0075187 | 86 | 18 | 15 | 39 | 43 | 42 | 15 | 3 | 4 | 0.0078841 | 0.8126166 | 0.9908205 | 0.0079571 |
| NC\_012920 | 9329 | 0.0000000 | 0.0000000 | 0.0000000 | 0.0000000 | 0.0000000 | 0.0464853 | 0.0000000 | 0.0000000 | 0.000 | 0.0075187 | 86 | 18 | 15 | 39 | 43 | 42 | 15 | 3 | 4 | 0.0201118 | 0.8113635 | 0.9909196 | 0.0202961 |
| NC\_012920 | 9335 | 0.0229854 | 0.0000000 | 0.0000000 | 0.0000000 | 0.0454300 | 0.0000000 | 0.0000000 | 0.0000000 | 0.000 | 0.0149804 | 86 | 18 | 15 | 39 | 43 | 42 | 15 | 3 | 4 | 0.0099699 | 0.8138127 | 0.9817758 | 0.0101550 |
| NC\_012920 | 9336 | 0.0229854 | 0.0000000 | 0.0000000 | 0.0000000 | 0.0000000 | 0.0000000 | 0.0000000 | 0.0000000 | 0.000 | 0.0075187 | 86 | 18 | 15 | 39 | 43 | 42 | 15 | 3 | 4 | 0.0078841 | 0.8126166 | 0.9908205 | 0.0079571 |
| NC\_012920 | 9344 | 0.0229854 | 0.0000000 | 0.0000000 | 0.0000000 | 0.0000000 | 0.0000000 | 0.0000000 | 0.0000000 | 0.000 | 0.0075187 | 86 | 18 | 15 | 39 | 43 | 42 | 15 | 3 | 4 | 0.0078841 | 0.8126166 | 0.9908205 | 0.0079571 |
| NC\_012920 | 9347 | 0.0454300 | 0.0000000 | 0.0000000 | 0.0000000 | 0.0454300 | 0.0000000 | 0.0000000 | 0.0000000 | 0.000 | 0.0223852 | 86 | 18 | 15 | 39 | 43 | 42 | 15 | 3 | 4 | 0.0120717 | 0.8161766 | 0.9729042 | 0.0124079 |
| NC\_012920 | 9377 | 0.0000000 | 0.0000000 | 0.0000000 | 0.0000000 | 0.0000000 | 0.0464853 | 0.0000000 | 0.0000000 | 0.000 | 0.0075187 | 86 | 18 | 15 | 39 | 43 | 42 | 15 | 3 | 4 | 0.0201118 | 0.8113635 | 0.9909196 | 0.0202961 |
| NC\_012920 | 9386 | 0.0000000 | 0.0000000 | 0.1244444 | 0.0000000 | 0.0000000 | 0.0000000 | 0.0000000 | 0.0000000 | 0.000 | 0.0075187 | 86 | 18 | 15 | 39 | 43 | 42 | 15 | 3 | 4 | 0.0631313 | 0.8105945 | 0.9913101 | 0.0636847 |
| NC\_012920 | 9389 | 0.0454300 | 0.0000000 | 0.0000000 | 0.0499671 | 0.0000000 | 0.0464853 | 0.0000000 | 0.0000000 | 0.000 | 0.0297330 | 86 | 18 | 15 | 39 | 43 | 42 | 15 | 3 | 4 | 0.0090325 | 0.8172303 | 0.9639460 | 0.0093704 |
| NC\_012920 | 9449 | 0.0000000 | 0.0000000 | 0.0000000 | 0.0000000 | 0.0000000 | 0.0464853 | 0.0000000 | 0.0000000 | 0.000 | 0.0075187 | 86 | 18 | 15 | 39 | 43 | 42 | 15 | 3 | 4 | 0.0201118 | 0.8113635 | 0.9909196 | 0.0202961 |
| NC\_012920 | 9477 | 0.0229854 | 0.0000000 | 0.0000000 | 0.0499671 | 0.0000000 | 0.0000000 | 0.0000000 | 0.0000000 | 0.000 | 0.0149804 | 86 | 18 | 15 | 39 | 43 | 42 | 15 | 3 | 4 | 0.0111716 | 0.8136988 | 0.9817954 | 0.0113788 |
| NC\_012920 | 9489 | 0.0229854 | 0.0000000 | 0.0000000 | 0.0000000 | 0.0000000 | 0.0000000 | 0.0000000 | 0.0000000 | 0.000 | 0.0075187 | 86 | 18 | 15 | 39 | 43 | 42 | 15 | 3 | 4 | 0.0078841 | 0.8126166 | 0.9908205 | 0.0079571 |
| NC\_012920 | 9530 | 0.0000000 | 0.1049383 | 0.0000000 | 0.0000000 | 0.0000000 | 0.0000000 | 0.0000000 | 0.0000000 | 0.000 | 0.0075187 | 86 | 18 | 15 | 39 | 43 | 42 | 15 | 3 | 4 | 0.0519781 | 0.8106800 | 0.9912075 | 0.0524392 |
| NC\_012920 | 9531 | 0.0000000 | 0.0000000 | 0.0000000 | 0.0000000 | 0.0454300 | 0.0000000 | 0.0000000 | 0.0000000 | 0.000 | 0.0075187 | 86 | 18 | 15 | 39 | 43 | 42 | 15 | 3 | 4 | 0.0195560 | 0.8113920 | 0.9909148 | 0.0197353 |
| NC\_012920 | 9540 | 0.2401298 | 0.2777778 | 0.3200000 | 0.3550296 | 0.4672796 | 0.3426532 | 0.3911111 | 0.0000000 | 0.000 | 0.3342516 | 86 | 18 | 15 | 39 | 43 | 41 | 15 | 3 | 4 | 0.0446116 | 0.8674242 | 0.6318525 | 0.0706045 |
| NC\_012920 | 9554 | 0.0000000 | 0.0000000 | 0.0000000 | 0.0000000 | 0.0454300 | 0.0000000 | 0.1244444 | 0.0000000 | 0.000 | 0.0149804 | 86 | 18 | 15 | 39 | 43 | 42 | 15 | 3 | 4 | 0.0376986 | 0.8117907 | 0.9822421 | 0.0383801 |
| NC\_012920 | 9572 | 0.0229854 | 0.0000000 | 0.0000000 | 0.0000000 | 0.0000000 | 0.0000000 | 0.0000000 | 0.0000000 | 0.000 | 0.0075187 | 86 | 18 | 15 | 39 | 43 | 42 | 15 | 3 | 4 | 0.0078841 | 0.8126166 | 0.9908205 | 0.0079571 |
| NC\_012920 | 9581 | 0.0229854 | 0.0000000 | 0.0000000 | 0.0000000 | 0.0000000 | 0.0000000 | 0.0000000 | 0.0000000 | 0.000 | 0.0075187 | 86 | 18 | 15 | 39 | 43 | 42 | 15 | 3 | 4 | 0.0078841 | 0.8126166 | 0.9908205 | 0.0079571 |
| NC\_012920 | 9614 | 0.0000000 | 0.1049383 | 0.0000000 | 0.0499671 | 0.0000000 | 0.0475907 | 0.0000000 | 0.0000000 | 0.000 | 0.0224690 | 86 | 18 | 15 | 39 | 43 | 41 | 15 | 3 | 4 | 0.0241059 | 0.8126722 | 0.9730182 | 0.0247744 |
| NC\_012920 | 9620 | 0.0229854 | 0.0000000 | 0.0000000 | 0.0000000 | 0.0000000 | 0.0000000 | 0.0000000 | 0.0000000 | 0.000 | 0.0075471 | 86 | 18 | 15 | 39 | 43 | 41 | 15 | 3 | 4 | 0.0078698 | 0.8123852 | 0.9907831 | 0.0079430 |
| NC\_012920 | 9653 | 0.0000000 | 0.0000000 | 0.0000000 | 0.0000000 | 0.0000000 | 0.0475907 | 0.0000000 | 0.0000000 | 0.000 | 0.0075471 | 86 | 18 | 15 | 39 | 43 | 41 | 15 | 3 | 4 | 0.0206807 | 0.8110939 | 0.9908876 | 0.0208709 |
| NC\_012920 | 9656 | 0.0000000 | 0.1049383 | 0.0000000 | 0.0000000 | 0.0454300 | 0.0000000 | 0.0000000 | 0.0000000 | 0.000 | 0.0150367 | 86 | 18 | 15 | 39 | 43 | 41 | 15 | 3 | 4 | 0.0320729 | 0.8116391 | 0.9820678 | 0.0326585 |
| NC\_012920 | 9682 | 0.0000000 | 0.1049383 | 0.0000000 | 0.0000000 | 0.0000000 | 0.0000000 | 0.0000000 | 0.0000000 | 0.000 | 0.0075187 | 86 | 18 | 15 | 39 | 43 | 42 | 15 | 3 | 4 | 0.0519781 | 0.8106800 | 0.9912075 | 0.0524392 |
| NC\_012920 | 9689 | 0.0000000 | 0.0000000 | 0.0000000 | 0.0499671 | 0.0454300 | 0.0000000 | 0.0000000 | 0.0000000 | 0.000 | 0.0149804 | 86 | 18 | 15 | 39 | 43 | 42 | 15 | 3 | 4 | 0.0170298 | 0.8124742 | 0.9818760 | 0.0173441 |
| NC\_012920 | 9698 | 0.0886966 | 0.0000000 | 0.0000000 | 0.4260355 | 0.3309897 | 0.0000000 | 0.1244444 | 0.0000000 | 0.000 | 0.1769740 | 86 | 18 | 15 | 39 | 43 | 42 | 15 | 3 | 4 | 0.1397838 | 0.8378782 | 0.8183078 | 0.1708206 |
| NC\_012920 | 9716 | 0.0229854 | 0.0000000 | 0.0000000 | 0.4049967 | 0.3309897 | 0.0000000 | 0.1244444 | 0.0000000 | 0.000 | 0.1522535 | 86 | 18 | 15 | 39 | 43 | 42 | 15 | 3 | 4 | 0.1605151 | 0.8305020 | 0.8460997 | 0.1897118 |
| NC\_012920 | 9719 | 0.0000000 | 0.0000000 | 0.0000000 | 0.0000000 | 0.0000000 | 0.0464853 | 0.0000000 | 0.0000000 | 0.000 | 0.0075187 | 86 | 18 | 15 | 39 | 43 | 42 | 15 | 3 | 4 | 0.0201118 | 0.8113635 | 0.9909196 | 0.0202961 |
| NC\_012920 | 9746 | 0.0229854 | 0.0000000 | 0.0000000 | 0.0000000 | 0.0000000 | 0.0464853 | 0.0000000 | 0.0000000 | 0.000 | 0.0149804 | 86 | 18 | 15 | 39 | 43 | 42 | 15 | 3 | 4 | 0.0102489 | 0.8137843 | 0.9817803 | 0.0104391 |
| NC\_012920 | 9755 | 0.0454300 | 0.0000000 | 0.0000000 | 0.0000000 | 0.0454300 | 0.0000000 | 0.0000000 | 0.0000000 | 0.000 | 0.0223852 | 86 | 18 | 15 | 39 | 43 | 42 | 15 | 3 | 4 | 0.0120717 | 0.8161766 | 0.9729042 | 0.0124079 |
| NC\_012920 | 9758 | 0.0229854 | 0.0000000 | 0.0000000 | 0.0499671 | 0.0000000 | 0.0000000 | 0.0000000 | 0.0000000 | 0.000 | 0.0149804 | 86 | 18 | 15 | 39 | 43 | 42 | 15 | 3 | 4 | 0.0111716 | 0.8136988 | 0.9817954 | 0.0113788 |
| NC\_012920 | 9767 | 0.0229854 | 0.0000000 | 0.0000000 | 0.0000000 | 0.0454300 | 0.0000000 | 0.0000000 | 0.0000000 | 0.000 | 0.0149804 | 86 | 18 | 15 | 39 | 43 | 42 | 15 | 3 | 4 | 0.0099699 | 0.8138127 | 0.9817758 | 0.0101550 |
| NC\_012920 | 9804 | 0.0000000 | 0.0000000 | 0.0000000 | 0.0499671 | 0.0000000 | 0.0000000 | 0.0000000 | 0.0000000 | 0.000 | 0.0075471 | 86 | 18 | 15 | 39 | 43 | 41 | 15 | 3 | 4 | 0.0219362 | 0.8110365 | 0.9908987 | 0.0221377 |
| NC\_012920 | 9818 | 0.0454300 | 0.0000000 | 0.0000000 | 0.0000000 | 0.0454300 | 0.0000000 | 0.0000000 | 0.0000000 | 0.000 | 0.0223852 | 86 | 18 | 15 | 39 | 43 | 42 | 15 | 3 | 4 | 0.0120717 | 0.8161766 | 0.9729042 | 0.0124079 |
| NC\_012920 | 9855 | 0.0229854 | 0.0000000 | 0.0000000 | 0.0000000 | 0.0000000 | 0.0000000 | 0.3200000 | 0.0000000 | 0.000 | 0.0297330 | 86 | 18 | 15 | 39 | 43 | 42 | 15 | 3 | 4 | 0.1399247 | 0.8136419 | 0.9685702 | 0.1444652 |
| NC\_012920 | 9893 | 0.0000000 | 0.0000000 | 0.0000000 | 0.0000000 | 0.0886966 | 0.0000000 | 0.0000000 | 0.0000000 | 0.000 | 0.0149804 | 86 | 18 | 15 | 39 | 43 | 42 | 15 | 3 | 4 | 0.0392608 | 0.8125311 | 0.9822871 | 0.0399687 |
| NC\_012920 | 9899 | 0.0000000 | 0.0000000 | 0.0000000 | 0.0000000 | 0.0454300 | 0.0000000 | 0.0000000 | 0.0000000 | 0.000 | 0.0075187 | 86 | 18 | 15 | 39 | 43 | 42 | 15 | 3 | 4 | 0.0195560 | 0.8113920 | 0.9909148 | 0.0197353 |
| NC\_012920 | 9932 | 0.0229854 | 0.0000000 | 0.2311111 | 0.0000000 | 0.0000000 | 0.0464853 | 0.0000000 | 0.4444444 | 0.000 | 0.0370239 | 86 | 18 | 15 | 39 | 43 | 42 | 15 | 3 | 4 | 0.1103007 | 0.8145817 | 0.9595620 | 0.1149490 |
| NC\_012920 | 9941 | 0.0000000 | 0.0000000 | 0.0000000 | 0.0000000 | 0.0000000 | 0.0000000 | 0.1244444 | 0.0000000 | 0.000 | 0.0075187 | 86 | 18 | 15 | 39 | 43 | 42 | 15 | 3 | 4 | 0.0631313 | 0.8105945 | 0.9913101 | 0.0636847 |
| NC\_012920 | 9947 | 0.0000000 | 0.0000000 | 0.0000000 | 0.0000000 | 0.0000000 | 0.0907029 | 0.0000000 | 0.0000000 | 0.000 | 0.0149804 | 86 | 18 | 15 | 39 | 43 | 42 | 15 | 3 | 4 | 0.0403766 | 0.8124742 | 0.9823064 | 0.0411039 |
| NC\_012920 | 9950 | 0.0000000 | 0.0000000 | 0.0000000 | 0.0000000 | 0.0000000 | 0.0928019 | 0.0000000 | 0.0000000 | 0.000 | 0.0150367 | 86 | 18 | 15 | 39 | 43 | 41 | 15 | 3 | 4 | 0.0415193 | 0.8121843 | 0.9822547 | 0.0422694 |
| NC\_012920 | 9966 | 0.0000000 | 0.0000000 | 0.1244444 | 0.0499671 | 0.0000000 | 0.0928019 | 0.0000000 | 0.0000000 | 0.000 | 0.0298439 | 86 | 18 | 15 | 39 | 43 | 41 | 15 | 3 | 4 | 0.0328128 | 0.8136765 | 0.9645257 | 0.0340196 |
| NC\_012920 | 10032 | 0.0229854 | 0.0000000 | 0.0000000 | 0.0000000 | 0.0000000 | 0.0000000 | 0.0000000 | 0.0000000 | 0.000 | 0.0075471 | 86 | 18 | 15 | 39 | 43 | 41 | 15 | 3 | 4 | 0.0078698 | 0.8123852 | 0.9907831 | 0.0079430 |
| NC\_012920 | 10034 | 0.0454300 | 0.0000000 | 0.1244444 | 0.0000000 | 0.0000000 | 0.0475907 | 0.0000000 | 0.0000000 | 0.000 | 0.0298439 | 86 | 18 | 15 | 39 | 43 | 41 | 15 | 3 | 4 | 0.0195366 | 0.8163166 | 0.9641550 | 0.0202630 |
| NC\_012920 | 10115 | 0.0229854 | 0.1975309 | 0.0000000 | 0.0973044 | 0.0454300 | 0.0000000 | 0.1244444 | 0.0000000 | 0.000 | 0.0516242 | 86 | 18 | 15 | 39 | 43 | 41 | 15 | 3 | 4 | 0.0353268 | 0.8170340 | 0.9390472 | 0.0376198 |
| NC\_012920 | 10142 | 0.0000000 | 0.0000000 | 0.0000000 | 0.0000000 | 0.0886966 | 0.0475907 | 0.0000000 | 0.0000000 | 0.375 | 0.0298439 | 86 | 18 | 15 | 39 | 43 | 41 | 15 | 3 | 4 | 0.0778819 | 0.8135331 | 0.9661727 | 0.0806087 |
| NC\_012920 | 10143 | 0.0000000 | 0.0000000 | 0.0000000 | 0.0973044 | 0.0454300 | 0.0475907 | 0.0000000 | 0.0000000 | 0.000 | 0.0298439 | 86 | 18 | 15 | 39 | 43 | 41 | 15 | 3 | 4 | 0.0227457 | 0.8144226 | 0.9641893 | 0.0235904 |
| NC\_012920 | 10166 | 0.0454300 | 0.0000000 | 0.0000000 | 0.0000000 | 0.0454300 | 0.0000000 | 0.0000000 | 0.0000000 | 0.000 | 0.0225535 | 86 | 18 | 15 | 39 | 43 | 40 | 15 | 3 | 4 | 0.0119857 | 0.8157412 | 0.9726836 | 0.0123223 |
| NC\_012920 | 10172 | 0.0229854 | 0.0000000 | 0.0000000 | 0.0499671 | 0.0000000 | 0.0000000 | 0.0000000 | 0.0000000 | 0.000 | 0.0150935 | 86 | 18 | 15 | 39 | 43 | 40 | 15 | 3 | 4 | 0.0111144 | 0.8132256 | 0.9816463 | 0.0113222 |
| NC\_012920 | 10188 | 0.0000000 | 0.0000000 | 0.0000000 | 0.0000000 | 0.0000000 | 0.0000000 | 0.0000000 | 0.0000000 | 0.375 | 0.0076045 | 86 | 18 | 15 | 39 | 43 | 39 | 15 | 3 | 4 | 0.2471264 | 0.8094517 | 0.9929271 | 0.2488868 |
| NC\_012920 | 10217 | 0.0000000 | 0.0000000 | 0.0000000 | 0.0973044 | 0.0454300 | 0.0000000 | 0.0000000 | 0.0000000 | 0.000 | 0.0225535 | 86 | 18 | 15 | 39 | 43 | 40 | 15 | 3 | 4 | 0.0308854 | 0.8130232 | 0.9731165 | 0.0317387 |
| NC\_012920 | 10237 | 0.0229854 | 0.0000000 | 0.0000000 | 0.0499671 | 0.0454300 | 0.0000000 | 0.1244444 | 0.0000000 | 0.000 | 0.0299556 | 86 | 18 | 15 | 39 | 43 | 40 | 15 | 3 | 4 | 0.0168449 | 0.8148448 | 0.9638569 | 0.0174766 |
| NC\_012920 | 10238 | 0.1297999 | 0.0000000 | 0.2311111 | 0.0000000 | 0.0454300 | 0.0487500 | 0.0000000 | 0.0000000 | 0.375 | 0.0801515 | 86 | 18 | 15 | 39 | 43 | 40 | 15 | 3 | 4 | 0.0496636 | 0.8267287 | 0.9078647 | 0.0547038 |
| NC\_012920 | 10310 | 0.0229854 | 0.0000000 | 0.0000000 | 0.0000000 | 0.0000000 | 0.0000000 | 0.0000000 | 0.0000000 | 0.000 | 0.0075471 | 86 | 18 | 15 | 39 | 43 | 41 | 15 | 3 | 4 | 0.0078698 | 0.8123852 | 0.9907831 | 0.0079430 |
| NC\_012920 | 10321 | 0.0000000 | 0.0000000 | 0.0000000 | 0.0000000 | 0.0000000 | 0.0475907 | 0.0000000 | 0.0000000 | 0.000 | 0.0075471 | 86 | 18 | 15 | 39 | 43 | 41 | 15 | 3 | 4 | 0.0206807 | 0.8110939 | 0.9908876 | 0.0208709 |
| NC\_012920 | 10373 | 0.0229854 | 0.0000000 | 0.0000000 | 0.0000000 | 0.0000000 | 0.0000000 | 0.0000000 | 0.0000000 | 0.000 | 0.0075471 | 86 | 18 | 15 | 39 | 43 | 41 | 15 | 3 | 4 | 0.0078698 | 0.8123852 | 0.9907831 | 0.0079430 |
| NC\_012920 | 10376 | 0.0229854 | 0.0000000 | 0.0000000 | 0.0000000 | 0.0000000 | 0.0000000 | 0.0000000 | 0.0000000 | 0.000 | 0.0075471 | 86 | 18 | 15 | 39 | 43 | 41 | 15 | 3 | 4 | 0.0078698 | 0.8123852 | 0.9907831 | 0.0079430 |
| NC\_012920 | 10398 | 0.4672796 | 0.4444444 | 0.4800000 | 0.4733728 | 0.4672796 | 0.4497323 | 0.4800000 | 0.0000000 | 0.375 | 0.4628099 | 86 | 18 | 15 | 39 | 43 | 41 | 15 | 3 | 4 | 0.0090209 | 0.8983586 | 0.4894745 | 0.0184297 |
| NC\_012920 | 10400 | 0.1297999 | 0.1049383 | 0.3200000 | 0.2235371 | 0.4218496 | 0.2141582 | 0.0000000 | 0.0000000 | 0.000 | 0.2187500 | 86 | 18 | 15 | 39 | 43 | 41 | 15 | 3 | 4 | 0.0737723 | 0.8464761 | 0.7606402 | 0.0969871 |
| NC\_012920 | 10410 | 0.0454300 | 0.0000000 | 0.0000000 | 0.0499671 | 0.0000000 | 0.0000000 | 0.0000000 | 0.0000000 | 0.000 | 0.0225264 | 86 | 18 | 15 | 39 | 43 | 41 | 15 | 3 | 4 | 0.0153482 | 0.8158574 | 0.9728131 | 0.0157771 |
| NC\_012920 | 10463 | 0.1297999 | 0.1975309 | 0.2311111 | 0.0000000 | 0.0886966 | 0.0475907 | 0.1244444 | 0.0000000 | 0.000 | 0.1004362 | 86 | 18 | 15 | 39 | 43 | 41 | 15 | 3 | 4 | 0.0263358 | 0.8292872 | 0.8820781 | 0.0298566 |
| NC\_012920 | 10497 | 0.0000000 | 0.1049383 | 0.0000000 | 0.0000000 | 0.0000000 | 0.0000000 | 0.0000000 | 0.0000000 | 0.000 | 0.0075471 | 86 | 18 | 15 | 39 | 43 | 41 | 15 | 3 | 4 | 0.0519645 | 0.8104339 | 0.9911715 | 0.0524274 |
| NC\_012920 | 10499 | 0.1095187 | 0.0000000 | 0.1244444 | 0.0000000 | 0.0000000 | 0.0000000 | 0.0000000 | 0.0000000 | 0.000 | 0.0444215 | 86 | 18 | 15 | 39 | 43 | 41 | 15 | 3 | 4 | 0.0376900 | 0.8219697 | 0.9479941 | 0.0397577 |
| NC\_012920 | 10506 | 0.0454300 | 0.0000000 | 0.0000000 | 0.0000000 | 0.0000000 | 0.0000000 | 0.0000000 | 0.0000000 | 0.000 | 0.0150367 | 86 | 18 | 15 | 39 | 43 | 41 | 15 | 3 | 4 | 0.0157998 | 0.8147670 | 0.9818363 | 0.0160920 |
| NC\_012920 | 10511 | 0.0229854 | 0.0000000 | 0.0000000 | 0.0000000 | 0.0000000 | 0.0000000 | 0.0000000 | 0.0000000 | 0.000 | 0.0075471 | 86 | 18 | 15 | 39 | 43 | 41 | 15 | 3 | 4 | 0.0078698 | 0.8123852 | 0.9907831 | 0.0079430 |
| NC\_012920 | 10514 | 0.0000000 | 0.0000000 | 0.1244444 | 0.0000000 | 0.0000000 | 0.0000000 | 0.0000000 | 0.0000000 | 0.000 | 0.0075471 | 86 | 18 | 15 | 39 | 43 | 41 | 15 | 3 | 4 | 0.0631179 | 0.8103478 | 0.9912745 | 0.0636735 |
| NC\_012920 | 10548 | 0.0000000 | 0.0000000 | 0.0000000 | 0.0000000 | 0.0000000 | 0.0000000 | 0.0000000 | 0.0000000 | 0.375 | 0.0075471 | 86 | 18 | 15 | 39 | 43 | 41 | 15 | 3 | 4 | 0.2471483 | 0.8100321 | 0.9929857 | 0.2488941 |
| NC\_012920 | 10550 | 0.0886966 | 0.0000000 | 0.0000000 | 0.4260355 | 0.3309897 | 0.0000000 | 0.1244444 | 0.0000000 | 0.000 | 0.1775712 | 86 | 18 | 15 | 39 | 43 | 41 | 15 | 3 | 4 | 0.1394292 | 0.8378386 | 0.8176110 | 0.1705324 |
| NC\_012920 | 10556 | 0.0000000 | 0.0000000 | 0.0000000 | 0.0000000 | 0.0886966 | 0.0000000 | 0.0000000 | 0.0000000 | 0.000 | 0.0150367 | 86 | 18 | 15 | 39 | 43 | 41 | 15 | 3 | 4 | 0.0392331 | 0.8122991 | 0.9822149 | 0.0399435 |
| NC\_012920 | 10586 | 0.0000000 | 0.0000000 | 0.0000000 | 0.0499671 | 0.0000000 | 0.0475907 | 0.0000000 | 0.0000000 | 0.000 | 0.0150367 | 86 | 18 | 15 | 39 | 43 | 41 | 15 | 3 | 4 | 0.0175730 | 0.8121843 | 0.9818114 | 0.0178986 |
| NC\_012920 | 10589 | 0.0454300 | 0.0000000 | 0.0000000 | 0.0000000 | 0.0454300 | 0.0000000 | 0.0000000 | 0.0000000 | 0.000 | 0.0224690 | 86 | 18 | 15 | 39 | 43 | 41 | 15 | 3 | 4 | 0.0120289 | 0.8159722 | 0.9727947 | 0.0123653 |
| NC\_012920 | 10592 | 0.0229854 | 0.0000000 | 0.0000000 | 0.0000000 | 0.0000000 | 0.0000000 | 0.0000000 | 0.0000000 | 0.000 | 0.0075471 | 86 | 18 | 15 | 39 | 43 | 41 | 15 | 3 | 4 | 0.0078698 | 0.8123852 | 0.9907831 | 0.0079430 |
| NC\_012920 | 10601 | 0.0229854 | 0.0000000 | 0.0000000 | 0.0000000 | 0.0000000 | 0.0000000 | 0.0000000 | 0.0000000 | 0.000 | 0.0075471 | 86 | 18 | 15 | 39 | 43 | 41 | 15 | 3 | 4 | 0.0078698 | 0.8123852 | 0.9907831 | 0.0079430 |
| NC\_012920 | 10609 | 0.0229854 | 0.0000000 | 0.0000000 | 0.0000000 | 0.0000000 | 0.0000000 | 0.0000000 | 0.0000000 | 0.000 | 0.0075471 | 86 | 18 | 15 | 39 | 43 | 41 | 15 | 3 | 4 | 0.0078698 | 0.8123852 | 0.9907831 | 0.0079430 |
| NC\_012920 | 10610 | 0.0000000 | 0.0000000 | 0.0000000 | 0.0000000 | 0.0886966 | 0.0000000 | 0.0000000 | 0.0000000 | 0.000 | 0.0150367 | 86 | 18 | 15 | 39 | 43 | 41 | 15 | 3 | 4 | 0.0392331 | 0.8122991 | 0.9822149 | 0.0399435 |
| NC\_012920 | 10631 | 0.0000000 | 0.0000000 | 0.0000000 | 0.0000000 | 0.0454300 | 0.0000000 | 0.0000000 | 0.0000000 | 0.000 | 0.0075471 | 86 | 18 | 15 | 39 | 43 | 41 | 15 | 3 | 4 | 0.0195420 | 0.8111513 | 0.9908777 | 0.0197219 |
| NC\_012920 | 10634 | 0.0000000 | 0.1049383 | 0.0000000 | 0.0000000 | 0.0000000 | 0.0000000 | 0.0000000 | 0.0000000 | 0.000 | 0.0075471 | 86 | 18 | 15 | 39 | 43 | 41 | 15 | 3 | 4 | 0.0519645 | 0.8104339 | 0.9911715 | 0.0524274 |
| NC\_012920 | 10640 | 0.0000000 | 0.0000000 | 0.0000000 | 0.0000000 | 0.0454300 | 0.0475907 | 0.0000000 | 0.0000000 | 0.000 | 0.0150367 | 86 | 18 | 15 | 39 | 43 | 41 | 15 | 3 | 4 | 0.0163713 | 0.8122991 | 0.9817917 | 0.0166749 |
| NC\_012920 | 10646 | 0.0229854 | 0.0000000 | 0.0000000 | 0.0000000 | 0.0000000 | 0.0000000 | 0.0000000 | 0.0000000 | 0.000 | 0.0075471 | 86 | 18 | 15 | 39 | 43 | 41 | 15 | 3 | 4 | 0.0078698 | 0.8123852 | 0.9907831 | 0.0079430 |
| NC\_012920 | 10654 | 0.0000000 | 0.1049383 | 0.0000000 | 0.0499671 | 0.0000000 | 0.0000000 | 0.0000000 | 0.0000000 | 0.000 | 0.0150367 | 86 | 18 | 15 | 39 | 43 | 41 | 15 | 3 | 4 | 0.0332746 | 0.8115243 | 0.9820875 | 0.0338815 |
| NC\_012920 | 10664 | 0.0454300 | 0.0000000 | 0.0000000 | 0.0000000 | 0.0454300 | 0.0000000 | 0.0000000 | 0.0000000 | 0.000 | 0.0224690 | 86 | 18 | 15 | 39 | 43 | 41 | 15 | 3 | 4 | 0.0120289 | 0.8159722 | 0.9727947 | 0.0123653 |
| NC\_012920 | 10670 | 0.0229854 | 0.0000000 | 0.0000000 | 0.0499671 | 0.0000000 | 0.0475907 | 0.0000000 | 0.0000000 | 0.000 | 0.0224690 | 86 | 18 | 15 | 39 | 43 | 41 | 15 | 3 | 4 | 0.0092950 | 0.8146235 | 0.9726743 | 0.0095562 |
| NC\_012920 | 10685 | 0.0229854 | 0.0000000 | 0.2311111 | 0.0000000 | 0.0000000 | 0.0928019 | 0.0000000 | 0.4444444 | 0.000 | 0.0444215 | 86 | 18 | 15 | 39 | 43 | 41 | 15 | 3 | 4 | 0.0976911 | 0.8154270 | 0.9508455 | 0.1027413 |
| NC\_012920 | 10688 | 0.0886966 | 0.0000000 | 0.0000000 | 0.0499671 | 0.0454300 | 0.0928019 | 0.0000000 | 0.0000000 | 0.000 | 0.0587695 | 86 | 18 | 15 | 39 | 43 | 41 | 15 | 3 | 4 | 0.0116119 | 0.8238923 | 0.9294967 | 0.0124927 |
| NC\_012920 | 10700 | 0.0000000 | 0.0000000 | 0.0000000 | 0.0000000 | 0.0000000 | 0.0000000 | 0.1244444 | 0.0000000 | 0.000 | 0.0075471 | 86 | 18 | 15 | 39 | 43 | 41 | 15 | 3 | 4 | 0.0631179 | 0.8103478 | 0.9912745 | 0.0636735 |
| NC\_012920 | 10727 | 0.0000000 | 0.0000000 | 0.0000000 | 0.0000000 | 0.0454300 | 0.0000000 | 0.0000000 | 0.0000000 | 0.000 | 0.0075471 | 86 | 18 | 15 | 39 | 43 | 41 | 15 | 3 | 4 | 0.0195420 | 0.8111513 | 0.9908777 | 0.0197219 |
| NC\_012920 | 10783 | 0.0000000 | 0.0000000 | 0.0000000 | 0.0000000 | 0.0000000 | 0.0000000 | 0.3200000 | 0.0000000 | 0.000 | 0.0224690 | 86 | 18 | 15 | 39 | 43 | 41 | 15 | 3 | 4 | 0.1908046 | 0.8109791 | 0.9775804 | 0.1951805 |
| NC\_012920 | 10793 | 0.0000000 | 0.0000000 | 0.0000000 | 0.0000000 | 0.0454300 | 0.0000000 | 0.0000000 | 0.0000000 | 0.000 | 0.0075471 | 86 | 18 | 15 | 39 | 43 | 41 | 15 | 3 | 4 | 0.0195420 | 0.8111513 | 0.9908777 | 0.0197219 |
| NC\_012920 | 10810 | 0.0454300 | 0.0000000 | 0.0000000 | 0.0499671 | 0.0454300 | 0.0928019 | 0.0000000 | 0.0000000 | 0.000 | 0.0444215 | 86 | 18 | 15 | 39 | 43 | 41 | 15 | 3 | 4 | 0.0096537 | 0.8193010 | 0.9463046 | 0.0102015 |
| NC\_012920 | 10822 | 0.1095187 | 0.0000000 | 0.0000000 | 0.0000000 | 0.0000000 | 0.0000000 | 0.0000000 | 0.0000000 | 0.000 | 0.0371614 | 86 | 18 | 15 | 39 | 43 | 41 | 15 | 3 | 4 | 0.0399569 | 0.8215680 | 0.9565751 | 0.0417708 |
| NC\_012920 | 10873 | 0.2879935 | 0.3456790 | 0.3200000 | 0.3813281 | 0.4542996 | 0.3426532 | 0.3911111 | 0.0000000 | 0.000 | 0.3512397 | 86 | 18 | 15 | 39 | 43 | 41 | 15 | 3 | 4 | 0.0282062 | 0.8730487 | 0.6090338 | 0.0463130 |
| NC\_012920 | 10876 | 0.0673337 | 0.2777778 | 0.1244444 | 0.0000000 | 0.0454300 | 0.0928019 | 0.0000000 | 0.0000000 | 0.000 | 0.0728880 | 86 | 18 | 15 | 39 | 43 | 41 | 15 | 3 | 4 | 0.0429620 | 0.8222280 | 0.9151615 | 0.0469447 |
| NC\_012920 | 10915 | 0.0454300 | 0.0000000 | 0.0000000 | 0.0000000 | 0.0886966 | 0.0000000 | 0.0000000 | 0.0000000 | 0.000 | 0.0299556 | 86 | 18 | 15 | 39 | 43 | 40 | 15 | 3 | 4 | 0.0199785 | 0.8168977 | 0.9640626 | 0.0207232 |
| NC\_012920 | 11002 | 0.1095187 | 0.0000000 | 0.1244444 | 0.0000000 | 0.0000000 | 0.0000000 | 0.0000000 | 0.0000000 | 0.000 | 0.0444215 | 86 | 18 | 15 | 39 | 43 | 41 | 15 | 3 | 4 | 0.0376900 | 0.8219697 | 0.9479941 | 0.0397577 |
| NC\_012920 | 11009 | 0.0229854 | 0.0000000 | 0.0000000 | 0.0000000 | 0.0454300 | 0.0000000 | 0.1244444 | 0.0000000 | 0.000 | 0.0224690 | 86 | 18 | 15 | 39 | 43 | 41 | 15 | 3 | 4 | 0.0227450 | 0.8139922 | 0.9730244 | 0.0233755 |
| NC\_012920 | 11025 | 0.0229854 | 0.0000000 | 0.0000000 | 0.0000000 | 0.0000000 | 0.0000000 | 0.0000000 | 0.0000000 | 0.375 | 0.0150367 | 86 | 18 | 15 | 39 | 43 | 41 | 15 | 3 | 4 | 0.1241789 | 0.8124713 | 0.9837908 | 0.1262249 |
| NC\_012920 | 11047 | 0.0229854 | 0.0000000 | 0.0000000 | 0.0000000 | 0.0454300 | 0.0000000 | 0.0000000 | 0.0000000 | 0.000 | 0.0150367 | 86 | 18 | 15 | 39 | 43 | 41 | 15 | 3 | 4 | 0.0099414 | 0.8135904 | 0.9817018 | 0.0101267 |
| NC\_012920 | 11050 | 0.0454300 | 0.0000000 | 0.0000000 | 0.0000000 | 0.0886966 | 0.0000000 | 0.0000000 | 0.0000000 | 0.000 | 0.0298439 | 86 | 18 | 15 | 39 | 43 | 41 | 15 | 3 | 4 | 0.0200358 | 0.8171201 | 0.9642085 | 0.0207795 |
| NC\_012920 | 11083 | 0.0000000 | 0.0000000 | 0.0000000 | 0.0499671 | 0.0000000 | 0.0000000 | 0.0000000 | 0.0000000 | 0.000 | 0.0075471 | 86 | 18 | 15 | 39 | 43 | 41 | 15 | 3 | 4 | 0.0219362 | 0.8110365 | 0.9908987 | 0.0221377 |
| NC\_012920 | 11084 | 0.0000000 | 0.1049383 | 0.0000000 | 0.0000000 | 0.0000000 | 0.0000000 | 0.0000000 | 0.0000000 | 0.000 | 0.0075471 | 86 | 18 | 15 | 39 | 43 | 41 | 15 | 3 | 4 | 0.0519645 | 0.8104339 | 0.9911715 | 0.0524274 |
| NC\_012920 | 11092 | 0.0229854 | 0.0000000 | 0.0000000 | 0.0000000 | 0.0000000 | 0.0000000 | 0.0000000 | 0.0000000 | 0.000 | 0.0075471 | 86 | 18 | 15 | 39 | 43 | 41 | 15 | 3 | 4 | 0.0078698 | 0.8123852 | 0.9907831 | 0.0079430 |
| NC\_012920 | 11204 | 0.0229854 | 0.1975309 | 0.0000000 | 0.0499671 | 0.0454300 | 0.0000000 | 0.1244444 | 0.0000000 | 0.000 | 0.0444215 | 86 | 18 | 15 | 39 | 43 | 41 | 15 | 3 | 4 | 0.0363341 | 0.8160009 | 0.9475399 | 0.0383457 |
| NC\_012920 | 11251 | 0.3442401 | 0.4012346 | 0.3200000 | 0.2235371 | 0.0886966 | 0.0487500 | 0.3200000 | 0.0000000 | 0.375 | 0.2525987 | 86 | 18 | 15 | 39 | 43 | 40 | 15 | 3 | 4 | 0.0605822 | 0.8589397 | 0.7237341 | 0.0837078 |
| NC\_012920 | 11253 | 0.0229854 | 0.0000000 | 0.0000000 | 0.0000000 | 0.0000000 | 0.0000000 | 0.0000000 | 0.0000000 | 0.000 | 0.0075756 | 86 | 18 | 15 | 39 | 43 | 40 | 15 | 3 | 4 | 0.0078555 | 0.8121268 | 0.9907451 | 0.0079289 |
| NC\_012920 | 11260 | 0.0000000 | 0.0000000 | 0.1244444 | 0.0000000 | 0.0000000 | 0.0000000 | 0.0000000 | 0.0000000 | 0.000 | 0.0075756 | 86 | 18 | 15 | 39 | 43 | 40 | 15 | 3 | 4 | 0.0631043 | 0.8100739 | 0.9912383 | 0.0636621 |
| NC\_012920 | 11266 | 0.0000000 | 0.0000000 | 0.0000000 | 0.0000000 | 0.0000000 | 0.0487500 | 0.0000000 | 0.0000000 | 0.000 | 0.0075756 | 86 | 18 | 15 | 39 | 43 | 40 | 15 | 3 | 4 | 0.0212786 | 0.8107967 | 0.9908554 | 0.0214750 |
| NC\_012920 | 11293 | 0.0000000 | 0.0000000 | 0.0000000 | 0.0000000 | 0.0454300 | 0.0000000 | 0.0000000 | 0.0000000 | 0.375 | 0.0150935 | 86 | 18 | 15 | 39 | 43 | 40 | 15 | 3 | 4 | 0.1300120 | 0.8109702 | 0.9838081 | 0.1321518 |
| NC\_012920 | 11299 | 0.0886966 | 0.0000000 | 0.0000000 | 0.4260355 | 0.3309897 | 0.0487500 | 0.1244444 | 0.0000000 | 0.000 | 0.1842444 | 86 | 18 | 15 | 39 | 43 | 40 | 15 | 3 | 4 | 0.1272025 | 0.8389018 | 0.8083112 | 0.1573682 |
| NC\_012920 | 11332 | 0.0000000 | 0.0000000 | 0.0000000 | 0.0000000 | 0.0000000 | 0.0000000 | 0.1244444 | 0.0000000 | 0.000 | 0.0075756 | 86 | 18 | 15 | 39 | 43 | 40 | 15 | 3 | 4 | 0.0631043 | 0.8100739 | 0.9912383 | 0.0636621 |
| NC\_012920 | 11337 | 0.0454300 | 0.0000000 | 0.0000000 | 0.0000000 | 0.0000000 | 0.0000000 | 0.0000000 | 0.0000000 | 0.000 | 0.0150935 | 86 | 18 | 15 | 39 | 43 | 40 | 15 | 3 | 4 | 0.0157712 | 0.8145267 | 0.9817619 | 0.0160642 |
| NC\_012920 | 11344 | 0.0229854 | 0.0000000 | 0.0000000 | 0.0000000 | 0.0000000 | 0.0000000 | 0.0000000 | 0.0000000 | 0.000 | 0.0075756 | 86 | 18 | 15 | 39 | 43 | 40 | 15 | 3 | 4 | 0.0078555 | 0.8121268 | 0.9907451 | 0.0079289 |
| NC\_012920 | 11348 | 0.0000000 | 0.0000000 | 0.0000000 | 0.4049967 | 0.3309897 | 0.0000000 | 0.1244444 | 0.0000000 | 0.000 | 0.1469444 | 86 | 18 | 15 | 39 | 43 | 40 | 15 | 3 | 4 | 0.1747199 | 0.8278275 | 0.8535077 | 0.2047080 |
| NC\_012920 | 11353 | 0.0000000 | 0.0000000 | 0.0000000 | 0.0499671 | 0.0454300 | 0.0000000 | 0.1244444 | 0.0000000 | 0.000 | 0.0225535 | 86 | 18 | 15 | 39 | 43 | 40 | 15 | 3 | 4 | 0.0274272 | 0.8123871 | 0.9729995 | 0.0281883 |
| NC\_012920 | 11362 | 0.0229854 | 0.0000000 | 0.0000000 | 0.0000000 | 0.0454300 | 0.0000000 | 0.0000000 | 0.0000000 | 0.000 | 0.0150935 | 86 | 18 | 15 | 39 | 43 | 40 | 15 | 3 | 4 | 0.0099127 | 0.8133412 | 0.9816266 | 0.0100982 |
| NC\_012920 | 11377 | 0.1095187 | 0.0000000 | 0.1244444 | 0.0000000 | 0.0000000 | 0.0000000 | 0.0000000 | 0.0000000 | 0.000 | 0.0445864 | 86 | 18 | 15 | 39 | 43 | 40 | 15 | 3 | 4 | 0.0376049 | 0.8217843 | 0.9477846 | 0.0396767 |
| NC\_012920 | 11440 | 0.0229854 | 0.0000000 | 0.0000000 | 0.0000000 | 0.0000000 | 0.0928019 | 0.0000000 | 0.0000000 | 0.000 | 0.0224690 | 86 | 18 | 15 | 39 | 43 | 41 | 15 | 3 | 4 | 0.0253204 | 0.8146235 | 0.9731163 | 0.0260199 |
| NC\_012920 | 11467 | 0.3442401 | 0.4444444 | 0.2311111 | 0.4733728 | 0.4542996 | 0.3426532 | 0.3200000 | 0.4444444 | 0.375 | 0.3932220 | 86 | 18 | 15 | 39 | 43 | 41 | 15 | 3 | 4 | 0.0294840 | 0.8811123 | 0.5668790 | 0.0520111 |
| NC\_012920 | 11485 | 0.0886966 | 0.0000000 | 0.0000000 | 0.0000000 | 0.0000000 | 0.0000000 | 0.0000000 | 0.0000000 | 0.000 | 0.0298439 | 86 | 18 | 15 | 39 | 43 | 41 | 15 | 3 | 4 | 0.0318426 | 0.8193584 | 0.9647363 | 0.0330065 |
| NC\_012920 | 11582 | 0.0229854 | 0.0000000 | 0.0000000 | 0.0000000 | 0.0000000 | 0.0000000 | 0.0000000 | 0.0000000 | 0.000 | 0.0075471 | 86 | 18 | 15 | 39 | 43 | 41 | 15 | 3 | 4 | 0.0078698 | 0.8123852 | 0.9907831 | 0.0079430 |
| NC\_012920 | 11674 | 0.0000000 | 0.0000000 | 0.0000000 | 0.0000000 | 0.0000000 | 0.0928019 | 0.0000000 | 0.0000000 | 0.000 | 0.0150367 | 86 | 18 | 15 | 39 | 43 | 41 | 15 | 3 | 4 | 0.0415193 | 0.8121843 | 0.9822547 | 0.0422694 |
| NC\_012920 | 11719 | 0.4123851 | 0.2777778 | 0.3200000 | 0.3813281 | 0.2725798 | 0.2141582 | 0.4444444 | 0.4444444 | 0.000 | 0.3470787 | 86 | 18 | 15 | 39 | 43 | 41 | 15 | 3 | 4 | 0.0326367 | 0.8782427 | 0.6177010 | 0.0528357 |
| NC\_012920 | 11761 | 0.1095187 | 0.0000000 | 0.1244444 | 0.1840894 | 0.0000000 | 0.0475907 | 0.2311111 | 0.4444444 | 0.000 | 0.1004362 | 86 | 18 | 15 | 39 | 43 | 41 | 15 | 3 | 4 | 0.0489974 | 0.8279385 | 0.8846351 | 0.0553871 |
| NC\_012920 | 11809 | 0.0000000 | 0.0000000 | 0.0000000 | 0.0000000 | 0.0000000 | 0.0464853 | 0.0000000 | 0.0000000 | 0.000 | 0.0075187 | 86 | 18 | 15 | 39 | 43 | 42 | 15 | 3 | 4 | 0.0201118 | 0.8113635 | 0.9909196 | 0.0202961 |
| NC\_012920 | 11812 | 0.1297999 | 0.1049383 | 0.2311111 | 0.0000000 | 0.0454300 | 0.0907029 | 0.1244444 | 0.0000000 | 0.000 | 0.0933001 | 86 | 18 | 15 | 39 | 43 | 42 | 15 | 3 | 4 | 0.0233171 | 0.8289640 | 0.8900741 | 0.0261968 |
| NC\_012920 | 11827 | 0.0229854 | 0.0000000 | 0.0000000 | 0.0499671 | 0.0000000 | 0.0000000 | 0.0000000 | 0.0000000 | 0.000 | 0.0149804 | 86 | 18 | 15 | 39 | 43 | 42 | 15 | 3 | 4 | 0.0111716 | 0.8136988 | 0.9817954 | 0.0113788 |
| NC\_012920 | 11864 | 0.0000000 | 0.0000000 | 0.0000000 | 0.0000000 | 0.0000000 | 0.0464853 | 0.0000000 | 0.0000000 | 0.000 | 0.0075187 | 86 | 18 | 15 | 39 | 43 | 42 | 15 | 3 | 4 | 0.0201118 | 0.8113635 | 0.9909196 | 0.0202961 |
| NC\_012920 | 11869 | 0.0454300 | 0.0000000 | 0.0000000 | 0.0000000 | 0.0000000 | 0.0000000 | 0.0000000 | 0.0000000 | 0.000 | 0.0149804 | 86 | 18 | 15 | 39 | 43 | 42 | 15 | 3 | 4 | 0.0158281 | 0.8149804 | 0.9819096 | 0.0161197 |
| NC\_012920 | 11875 | 0.0000000 | 0.0000000 | 0.1244444 | 0.0000000 | 0.0000000 | 0.0000000 | 0.0000000 | 0.0000000 | 0.000 | 0.0075187 | 86 | 18 | 15 | 39 | 43 | 42 | 15 | 3 | 4 | 0.0631313 | 0.8105945 | 0.9913101 | 0.0636847 |
| NC\_012920 | 11887 | 0.0000000 | 0.0000000 | 0.0000000 | 0.0000000 | 0.0454300 | 0.0000000 | 0.0000000 | 0.0000000 | 0.000 | 0.0075187 | 86 | 18 | 15 | 39 | 43 | 42 | 15 | 3 | 4 | 0.0195560 | 0.8113920 | 0.9909148 | 0.0197353 |
| NC\_012920 | 11890 | 0.0229854 | 0.0000000 | 0.0000000 | 0.0000000 | 0.0000000 | 0.0000000 | 0.0000000 | 0.0000000 | 0.000 | 0.0075187 | 86 | 18 | 15 | 39 | 43 | 42 | 15 | 3 | 4 | 0.0078841 | 0.8126166 | 0.9908205 | 0.0079571 |
| NC\_012920 | 11893 | 0.0229854 | 0.0000000 | 0.0000000 | 0.0000000 | 0.0000000 | 0.0000000 | 0.0000000 | 0.0000000 | 0.000 | 0.0075187 | 86 | 18 | 15 | 39 | 43 | 42 | 15 | 3 | 4 | 0.0078841 | 0.8126166 | 0.9908205 | 0.0079571 |
| NC\_012920 | 11914 | 0.1495403 | 0.1975309 | 0.0000000 | 0.0973044 | 0.0886966 | 0.0464853 | 0.0000000 | 0.0000000 | 0.375 | 0.1067996 | 86 | 18 | 15 | 39 | 43 | 42 | 15 | 3 | 4 | 0.0291380 | 0.8325525 | 0.8754581 | 0.0332831 |
| NC\_012920 | 11923 | 0.0000000 | 0.0000000 | 0.0000000 | 0.0499671 | 0.0000000 | 0.0000000 | 0.0000000 | 0.0000000 | 0.000 | 0.0075187 | 86 | 18 | 15 | 39 | 43 | 42 | 15 | 3 | 4 | 0.0219503 | 0.8112780 | 0.9909357 | 0.0221511 |
| NC\_012920 | 11935 | 0.0673337 | 0.0000000 | 0.0000000 | 0.0000000 | 0.0000000 | 0.0000000 | 0.0000000 | 0.0000000 | 0.000 | 0.0223852 | 86 | 18 | 15 | 39 | 43 | 42 | 15 | 3 | 4 | 0.0238328 | 0.8172873 | 0.9732631 | 0.0244875 |
| NC\_012920 | 11944 | 0.0229854 | 0.2777778 | 0.0000000 | 0.0973044 | 0.0454300 | 0.0000000 | 0.1244444 | 0.0000000 | 0.000 | 0.0585546 | 86 | 18 | 15 | 39 | 43 | 42 | 15 | 3 | 4 | 0.0596257 | 0.8176006 | 0.9326526 | 0.0639313 |
| NC\_012920 | 11947 | 0.0000000 | 0.0000000 | 0.0000000 | 0.0000000 | 0.0000000 | 0.0907029 | 0.0000000 | 0.0000000 | 0.000 | 0.0149804 | 86 | 18 | 15 | 39 | 43 | 42 | 15 | 3 | 4 | 0.0403766 | 0.8124742 | 0.9823064 | 0.0411039 |
| NC\_012920 | 11959 | 0.0229854 | 0.0000000 | 0.0000000 | 0.0000000 | 0.0000000 | 0.0000000 | 0.0000000 | 0.0000000 | 0.000 | 0.0075756 | 86 | 18 | 15 | 39 | 43 | 40 | 15 | 3 | 4 | 0.0078555 | 0.8121268 | 0.9907451 | 0.0079289 |
| NC\_012920 | 11984 | 0.0000000 | 0.0000000 | 0.0000000 | 0.0499671 | 0.0000000 | 0.0000000 | 0.0000000 | 0.0000000 | 0.000 | 0.0075756 | 86 | 18 | 15 | 39 | 43 | 40 | 15 | 3 | 4 | 0.0219221 | 0.8107678 | 0.9908610 | 0.0221243 |
| NC\_012920 | 12001 | 0.0000000 | 0.0000000 | 0.0000000 | 0.0499671 | 0.1297999 | 0.0000000 | 0.0000000 | 0.0000000 | 0.000 | 0.0298439 | 86 | 18 | 15 | 39 | 43 | 41 | 15 | 3 | 4 | 0.0442549 | 0.8144800 | 0.9649799 | 0.0458609 |
| NC\_012920 | 12007 | 0.0886966 | 0.0000000 | 0.1244444 | 0.0499671 | 0.3309897 | 0.1760857 | 0.0000000 | 0.0000000 | 0.000 | 0.1335801 | 86 | 18 | 15 | 39 | 43 | 41 | 15 | 3 | 4 | 0.0671992 | 0.8338786 | 0.8505734 | 0.0790046 |
| NC\_012920 | 12019 | 0.0000000 | 0.0000000 | 0.0000000 | 0.0499671 | 0.0000000 | 0.0000000 | 0.0000000 | 0.0000000 | 0.000 | 0.0075471 | 86 | 18 | 15 | 39 | 43 | 41 | 15 | 3 | 4 | 0.0219362 | 0.8110365 | 0.9908987 | 0.0221377 |
| NC\_012920 | 12028 | 0.0229854 | 0.0000000 | 0.0000000 | 0.0000000 | 0.0000000 | 0.0475907 | 0.0000000 | 0.0000000 | 0.000 | 0.0150367 | 86 | 18 | 15 | 39 | 43 | 41 | 15 | 3 | 4 | 0.0105130 | 0.8135331 | 0.9817111 | 0.0107088 |
| NC\_012920 | 12061 | 0.0229854 | 0.0000000 | 0.0000000 | 0.0000000 | 0.0000000 | 0.0000000 | 0.0000000 | 0.0000000 | 0.000 | 0.0075471 | 86 | 18 | 15 | 39 | 43 | 41 | 15 | 3 | 4 | 0.0078698 | 0.8123852 | 0.9907831 | 0.0079430 |
| NC\_012920 | 12070 | 0.0000000 | 0.0000000 | 0.0000000 | 0.0499671 | 0.0000000 | 0.0000000 | 0.0000000 | 0.0000000 | 0.000 | 0.0075471 | 86 | 18 | 15 | 39 | 43 | 41 | 15 | 3 | 4 | 0.0219362 | 0.8110365 | 0.9908987 | 0.0221377 |
| NC\_012920 | 12076 | 0.0229854 | 0.0000000 | 0.0000000 | 0.0000000 | 0.0000000 | 0.0000000 | 0.1244444 | 0.0000000 | 0.000 | 0.0150367 | 86 | 18 | 15 | 39 | 43 | 41 | 15 | 3 | 4 | 0.0318125 | 0.8127870 | 0.9820883 | 0.0323927 |
| NC\_012920 | 12094 | 0.0000000 | 0.0000000 | 0.0000000 | 0.0000000 | 0.0000000 | 0.0000000 | 0.0000000 | 0.0000000 | 0.375 | 0.0075471 | 86 | 18 | 15 | 39 | 43 | 41 | 15 | 3 | 4 | 0.2471483 | 0.8100321 | 0.9929857 | 0.2488941 |
| NC\_012920 | 12106 | 0.0229854 | 0.1049383 | 0.0000000 | 0.0499671 | 0.0000000 | 0.0475907 | 0.0000000 | 0.0000000 | 0.000 | 0.0298439 | 86 | 18 | 15 | 39 | 43 | 41 | 15 | 3 | 4 | 0.0143699 | 0.8151113 | 0.9639129 | 0.0149079 |
| NC\_012920 | 12127 | 0.0229854 | 0.0000000 | 0.0000000 | 0.0000000 | 0.0000000 | 0.0464853 | 0.0000000 | 0.0000000 | 0.000 | 0.0149804 | 86 | 18 | 15 | 39 | 43 | 42 | 15 | 3 | 4 | 0.0102489 | 0.8137843 | 0.9817803 | 0.0104391 |
| NC\_012920 | 12171 | 0.0229854 | 0.0000000 | 0.0000000 | 0.0000000 | 0.0000000 | 0.0000000 | 0.0000000 | 0.0000000 | 0.000 | 0.0075187 | 86 | 18 | 15 | 39 | 43 | 42 | 15 | 3 | 4 | 0.0078841 | 0.8126166 | 0.9908205 | 0.0079571 |
| NC\_012920 | 12172 | 0.0229854 | 0.0000000 | 0.0000000 | 0.0499671 | 0.0454300 | 0.0000000 | 0.1244444 | 0.0000000 | 0.000 | 0.0297330 | 86 | 18 | 15 | 39 | 43 | 42 | 15 | 3 | 4 | 0.0169595 | 0.8152937 | 0.9641494 | 0.0175901 |
| NC\_012920 | 12173 | 0.0229854 | 0.0000000 | 0.0000000 | 0.0000000 | 0.0000000 | 0.0000000 | 0.0000000 | 0.0000000 | 0.000 | 0.0075187 | 86 | 18 | 15 | 39 | 43 | 42 | 15 | 3 | 4 | 0.0078841 | 0.8126166 | 0.9908205 | 0.0079571 |
| NC\_012920 | 12234 | 0.0000000 | 0.0000000 | 0.0000000 | 0.0499671 | 0.0000000 | 0.0464853 | 0.0000000 | 0.0000000 | 0.000 | 0.0149804 | 86 | 18 | 15 | 39 | 43 | 42 | 15 | 3 | 4 | 0.0173087 | 0.8124457 | 0.9818805 | 0.0176282 |
| NC\_012920 | 12308 | 0.3442401 | 0.4444444 | 0.2311111 | 0.4733728 | 0.4542996 | 0.1760857 | 0.3200000 | 0.4444444 | 0.375 | 0.3750000 | 86 | 18 | 15 | 39 | 43 | 41 | 15 | 3 | 4 | 0.0513072 | 0.8770948 | 0.5943884 | 0.0863193 |
| NC\_012920 | 12346 | 0.0454300 | 0.1049383 | 0.0000000 | 0.0000000 | 0.0454300 | 0.0000000 | 0.0000000 | 0.0000000 | 0.000 | 0.0298439 | 86 | 18 | 15 | 39 | 43 | 41 | 15 | 3 | 4 | 0.0164281 | 0.8164601 | 0.9640477 | 0.0170408 |
| NC\_012920 | 12358 | 0.0673337 | 0.1975309 | 0.1244444 | 0.0499671 | 0.0454300 | 0.0499671 | 0.0000000 | 0.0000000 | 0.000 | 0.0663423 | 86 | 18 | 15 | 39 | 43 | 39 | 15 | 3 | 4 | 0.0182856 | 0.8213974 | 0.9207093 | 0.0198604 |
| NC\_012920 | 12361 | 0.0454300 | 0.0000000 | 0.0000000 | 0.0000000 | 0.0000000 | 0.0000000 | 0.0000000 | 0.0000000 | 0.000 | 0.0152082 | 86 | 18 | 15 | 39 | 42 | 39 | 15 | 3 | 4 | 0.0157134 | 0.8140808 | 0.9816121 | 0.0160077 |
| NC\_012920 | 12372 | 0.3442401 | 0.4444444 | 0.2311111 | 0.4733728 | 0.4591837 | 0.1840894 | 0.3200000 | 0.4444444 | 0.375 | 0.3778570 | 86 | 18 | 15 | 39 | 42 | 39 | 15 | 3 | 4 | 0.0505908 | 0.8771744 | 0.5910267 | 0.0855982 |
| NC\_012920 | 12403 | 0.0000000 | 0.0000000 | 0.0000000 | 0.0000000 | 0.0454300 | 0.0000000 | 0.0000000 | 0.0000000 | 0.000 | 0.0076045 | 86 | 18 | 15 | 39 | 43 | 39 | 15 | 3 | 4 | 0.0195135 | 0.8105880 | 0.9908017 | 0.0196947 |
| NC\_012920 | 12406 | 0.0454300 | 0.1975309 | 0.1244444 | 0.0000000 | 0.0886966 | 0.0000000 | 0.1244444 | 0.0000000 | 0.000 | 0.0592040 | 86 | 18 | 15 | 39 | 43 | 39 | 15 | 3 | 4 | 0.0323394 | 0.8183964 | 0.9299980 | 0.0347736 |
| NC\_012920 | 12408 | 0.0000000 | 0.0000000 | 0.0000000 | 0.0499671 | 0.0000000 | 0.0000000 | 0.0000000 | 0.0000000 | 0.000 | 0.0076045 | 86 | 18 | 15 | 39 | 43 | 39 | 15 | 3 | 4 | 0.0219078 | 0.8104714 | 0.9908228 | 0.0221108 |
| NC\_012920 | 12414 | 0.0000000 | 0.0000000 | 0.0000000 | 0.0000000 | 0.0454300 | 0.0973044 | 0.0000000 | 0.0000000 | 0.000 | 0.0226385 | 86 | 18 | 15 | 39 | 43 | 39 | 15 | 3 | 4 | 0.0308428 | 0.8127440 | 0.9730047 | 0.0316985 |
| NC\_012920 | 12432 | 0.0000000 | 0.0000000 | 0.0000000 | 0.0000000 | 0.0000000 | 0.0487500 | 0.0000000 | 0.0000000 | 0.000 | 0.0075756 | 86 | 18 | 15 | 39 | 43 | 40 | 15 | 3 | 4 | 0.0212786 | 0.8107967 | 0.9908554 | 0.0214750 |
| NC\_012920 | 12477 | 0.0229854 | 0.0000000 | 0.0000000 | 0.0499671 | 0.0454300 | 0.0487500 | 0.0000000 | 0.0000000 | 0.000 | 0.0299556 | 86 | 18 | 15 | 39 | 43 | 40 | 15 | 3 | 4 | 0.0062674 | 0.8155677 | 0.9635004 | 0.0065048 |
| NC\_012920 | 12498 | 0.0000000 | 0.0000000 | 0.0000000 | 0.0000000 | 0.1297999 | 0.0000000 | 0.0000000 | 0.0000000 | 0.000 | 0.0225535 | 86 | 18 | 15 | 39 | 43 | 40 | 15 | 3 | 4 | 0.0590340 | 0.8131388 | 0.9739011 | 0.0606160 |
| NC\_012920 | 12501 | 0.1297999 | 0.0000000 | 0.2311111 | 0.0499671 | 0.0454300 | 0.0487500 | 0.0000000 | 0.0000000 | 0.375 | 0.0870910 | 86 | 18 | 15 | 39 | 43 | 40 | 15 | 3 | 4 | 0.0403092 | 0.8278275 | 0.8990364 | 0.0448360 |
| NC\_012920 | 12542 | 0.0229854 | 0.0000000 | 0.0000000 | 0.0000000 | 0.0000000 | 0.0000000 | 0.0000000 | 0.0000000 | 0.000 | 0.0075756 | 86 | 18 | 15 | 39 | 43 | 40 | 15 | 3 | 4 | 0.0078555 | 0.8121268 | 0.9907451 | 0.0079289 |
| NC\_012920 | 12561 | 0.0229854 | 0.0000000 | 0.0000000 | 0.0499671 | 0.0000000 | 0.0950000 | 0.0000000 | 0.0000000 | 0.000 | 0.0299556 | 86 | 18 | 15 | 39 | 43 | 40 | 15 | 3 | 4 | 0.0194032 | 0.8154231 | 0.9639765 | 0.0201283 |
| NC\_012920 | 12562 | 0.0000000 | 0.0000000 | 0.0000000 | 0.0499671 | 0.0000000 | 0.0000000 | 0.0000000 | 0.0000000 | 0.000 | 0.0075756 | 86 | 18 | 15 | 39 | 43 | 40 | 15 | 3 | 4 | 0.0219221 | 0.8107678 | 0.9908610 | 0.0221243 |
| NC\_012920 | 12570 | 0.1095187 | 0.0000000 | 0.1244444 | 0.0000000 | 0.0000000 | 0.0000000 | 0.0000000 | 0.0000000 | 0.000 | 0.0445864 | 86 | 18 | 15 | 39 | 43 | 40 | 15 | 3 | 4 | 0.0376049 | 0.8217843 | 0.9477846 | 0.0396767 |
| NC\_012920 | 12585 | 0.0000000 | 0.0000000 | 0.0000000 | 0.0000000 | 0.0454300 | 0.0000000 | 0.0000000 | 0.0000000 | 0.000 | 0.0075471 | 86 | 18 | 15 | 39 | 43 | 41 | 15 | 3 | 4 | 0.0195420 | 0.8111513 | 0.9908777 | 0.0197219 |
| NC\_012920 | 12609 | 0.0000000 | 0.0000000 | 0.0000000 | 0.0000000 | 0.0000000 | 0.0000000 | 0.3200000 | 0.0000000 | 0.000 | 0.0224690 | 86 | 18 | 15 | 39 | 43 | 41 | 15 | 3 | 4 | 0.1908046 | 0.8109791 | 0.9775804 | 0.1951805 |
| NC\_012920 | 12612 | 0.2879935 | 0.4259259 | 0.1244444 | 0.2235371 | 0.0000000 | 0.0000000 | 0.2311111 | 0.0000000 | 0.375 | 0.1963671 | 86 | 18 | 15 | 39 | 43 | 41 | 15 | 3 | 4 | 0.0743721 | 0.8485996 | 0.7858085 | 0.0946441 |
| NC\_012920 | 12615 | 0.0454300 | 0.0000000 | 0.0000000 | 0.0000000 | 0.0000000 | 0.0000000 | 0.0000000 | 0.0000000 | 0.000 | 0.0150367 | 86 | 18 | 15 | 39 | 43 | 41 | 15 | 3 | 4 | 0.0157998 | 0.8147670 | 0.9818363 | 0.0160920 |
| NC\_012920 | 12618 | 0.0000000 | 0.0000000 | 0.0000000 | 0.0000000 | 0.0886966 | 0.0475907 | 0.0000000 | 0.0000000 | 0.000 | 0.0224690 | 86 | 18 | 15 | 39 | 43 | 41 | 15 | 3 | 4 | 0.0280934 | 0.8134470 | 0.9731540 | 0.0288684 |
| NC\_012920 | 12630 | 0.0229854 | 0.0000000 | 0.0000000 | 0.0000000 | 0.0000000 | 0.0000000 | 0.0000000 | 0.0000000 | 0.000 | 0.0075471 | 86 | 18 | 15 | 39 | 43 | 41 | 15 | 3 | 4 | 0.0078698 | 0.8123852 | 0.9907831 | 0.0079430 |
| NC\_012920 | 12633 | 0.0000000 | 0.1049383 | 0.0000000 | 0.0000000 | 0.0454300 | 0.0000000 | 0.0000000 | 0.0000000 | 0.000 | 0.0150367 | 86 | 18 | 15 | 39 | 43 | 41 | 15 | 3 | 4 | 0.0320729 | 0.8116391 | 0.9820678 | 0.0326585 |
| NC\_012920 | 12654 | 0.0673337 | 0.0000000 | 0.2311111 | 0.0000000 | 0.0000000 | 0.0475907 | 0.0000000 | 0.4444444 | 0.000 | 0.0516242 | 86 | 18 | 15 | 39 | 43 | 41 | 15 | 3 | 4 | 0.0797482 | 0.8190427 | 0.9419966 | 0.0846586 |
| NC\_012920 | 12672 | 0.0000000 | 0.0000000 | 0.1244444 | 0.0000000 | 0.0000000 | 0.0000000 | 0.0000000 | 0.0000000 | 0.000 | 0.0075471 | 86 | 18 | 15 | 39 | 43 | 41 | 15 | 3 | 4 | 0.0631179 | 0.8103478 | 0.9912745 | 0.0636735 |
| NC\_012920 | 12681 | 0.0229854 | 0.0000000 | 0.0000000 | 0.0000000 | 0.0000000 | 0.0000000 | 0.0000000 | 0.0000000 | 0.000 | 0.0075471 | 86 | 18 | 15 | 39 | 43 | 41 | 15 | 3 | 4 | 0.0078698 | 0.8123852 | 0.9907831 | 0.0079430 |
| NC\_012920 | 12693 | 0.0229854 | 0.1975309 | 0.0000000 | 0.0973044 | 0.0454300 | 0.0000000 | 0.0000000 | 0.0000000 | 0.000 | 0.0444215 | 86 | 18 | 15 | 39 | 43 | 41 | 15 | 3 | 4 | 0.0380833 | 0.8166322 | 0.9476756 | 0.0401860 |
| NC\_012920 | 12705 | 0.3442401 | 0.2777778 | 0.4444444 | 0.3550296 | 0.4780963 | 0.4497323 | 0.3911111 | 0.0000000 | 0.375 | 0.3966942 | 86 | 18 | 15 | 39 | 43 | 41 | 15 | 3 | 4 | 0.0309945 | 0.8818297 | 0.5640894 | 0.0549461 |
| NC\_012920 | 12714 | 0.0229854 | 0.0000000 | 0.0000000 | 0.0000000 | 0.0000000 | 0.0000000 | 0.0000000 | 0.0000000 | 0.000 | 0.0075471 | 86 | 18 | 15 | 39 | 43 | 41 | 15 | 3 | 4 | 0.0078698 | 0.8123852 | 0.9907831 | 0.0079430 |
| NC\_012920 | 12720 | 0.0454300 | 0.0000000 | 0.0000000 | 0.0000000 | 0.0454300 | 0.0000000 | 0.0000000 | 0.0000000 | 0.000 | 0.0224690 | 86 | 18 | 15 | 39 | 43 | 41 | 15 | 3 | 4 | 0.0120289 | 0.8159722 | 0.9727947 | 0.0123653 |
| NC\_012920 | 12738 | 0.0000000 | 0.1049383 | 0.0000000 | 0.0000000 | 0.0000000 | 0.0000000 | 0.0000000 | 0.0000000 | 0.000 | 0.0075471 | 86 | 18 | 15 | 39 | 43 | 41 | 15 | 3 | 4 | 0.0519645 | 0.8104339 | 0.9911715 | 0.0524274 |
| NC\_012920 | 12757 | 0.0229854 | 0.0000000 | 0.0000000 | 0.0000000 | 0.0000000 | 0.0000000 | 0.0000000 | 0.0000000 | 0.000 | 0.0075471 | 86 | 18 | 15 | 39 | 43 | 41 | 15 | 3 | 4 | 0.0078698 | 0.8123852 | 0.9907831 | 0.0079430 |
| NC\_012920 | 12793 | 0.0000000 | 0.1049383 | 0.0000000 | 0.0499671 | 0.0000000 | 0.0475907 | 0.0000000 | 0.0000000 | 0.000 | 0.0224690 | 86 | 18 | 15 | 39 | 43 | 41 | 15 | 3 | 4 | 0.0241059 | 0.8126722 | 0.9730182 | 0.0247744 |
| NC\_012920 | 12810 | 0.0229854 | 0.0000000 | 0.1244444 | 0.1840894 | 0.0000000 | 0.0475907 | 0.0000000 | 0.0000000 | 0.000 | 0.0516242 | 86 | 18 | 15 | 39 | 43 | 41 | 15 | 3 | 4 | 0.0480358 | 0.8179522 | 0.9399178 | 0.0511064 |
| NC\_012920 | 12822 | 0.0229854 | 0.0000000 | 0.0000000 | 0.0000000 | 0.0454300 | 0.0000000 | 0.0000000 | 0.0000000 | 0.000 | 0.0150367 | 86 | 18 | 15 | 39 | 43 | 41 | 15 | 3 | 4 | 0.0099414 | 0.8135904 | 0.9817018 | 0.0101267 |
| NC\_012920 | 12879 | 0.0673337 | 0.1049383 | 0.1244444 | 0.0499671 | 0.0454300 | 0.0475907 | 0.1244444 | 0.0000000 | 0.000 | 0.0658574 | 86 | 18 | 15 | 39 | 43 | 41 | 15 | 3 | 4 | 0.0069030 | 0.8218262 | 0.9204177 | 0.0074998 |
| NC\_012920 | 12882 | 0.0229854 | 0.0000000 | 0.0000000 | 0.0000000 | 0.0000000 | 0.0000000 | 0.0000000 | 0.0000000 | 0.000 | 0.0075471 | 86 | 18 | 15 | 39 | 43 | 41 | 15 | 3 | 4 | 0.0078698 | 0.8123852 | 0.9907831 | 0.0079430 |
| NC\_012920 | 12945 | 0.0000000 | 0.0000000 | 0.0000000 | 0.0000000 | 0.0454300 | 0.0000000 | 0.0000000 | 0.0000000 | 0.000 | 0.0075471 | 86 | 18 | 15 | 39 | 43 | 41 | 15 | 3 | 4 | 0.0195420 | 0.8111513 | 0.9908777 | 0.0197219 |
| NC\_012920 | 12950 | 0.0229854 | 0.1049383 | 0.0000000 | 0.0000000 | 0.0886966 | 0.0000000 | 0.0000000 | 0.0000000 | 0.000 | 0.0298439 | 86 | 18 | 15 | 39 | 43 | 41 | 15 | 3 | 4 | 0.0252832 | 0.8152261 | 0.9643175 | 0.0262188 |
| NC\_012920 | 12969 | 0.0229854 | 0.0000000 | 0.0000000 | 0.0000000 | 0.0000000 | 0.0000000 | 0.0000000 | 0.0000000 | 0.000 | 0.0075471 | 86 | 18 | 15 | 39 | 43 | 41 | 15 | 3 | 4 | 0.0078698 | 0.8123852 | 0.9907831 | 0.0079430 |
| NC\_012920 | 12972 | 0.0000000 | 0.0000000 | 0.0000000 | 0.0000000 | 0.0000000 | 0.0000000 | 0.1244444 | 0.0000000 | 0.000 | 0.0075756 | 86 | 18 | 15 | 39 | 43 | 40 | 15 | 3 | 4 | 0.0631043 | 0.8100739 | 0.9912383 | 0.0636621 |
| NC\_012920 | 13020 | 0.0673337 | 0.2777778 | 0.2311111 | 0.0000000 | 0.0454300 | 0.0487500 | 0.0000000 | 0.0000000 | 0.000 | 0.0731542 | 86 | 18 | 15 | 39 | 43 | 40 | 15 | 3 | 4 | 0.0560664 | 0.8212639 | 0.9159190 | 0.0612132 |
| NC\_012920 | 13050 | 0.0000000 | 0.0000000 | 0.0000000 | 0.0000000 | 0.0000000 | 0.0475907 | 0.0000000 | 0.0000000 | 0.000 | 0.0075471 | 86 | 18 | 15 | 39 | 43 | 41 | 15 | 3 | 4 | 0.0206807 | 0.8110939 | 0.9908876 | 0.0208709 |
| NC\_012920 | 13104 | 0.0454300 | 0.1049383 | 0.1244444 | 0.0499671 | 0.0454300 | 0.0000000 | 0.1244444 | 0.0000000 | 0.000 | 0.0516242 | 86 | 18 | 15 | 39 | 43 | 41 | 15 | 3 | 4 | 0.0144828 | 0.8183540 | 0.9378307 | 0.0154428 |
| NC\_012920 | 13105 | 0.0454300 | 0.1049383 | 0.0000000 | 0.0973044 | 0.1297999 | 0.0928019 | 0.3200000 | 0.0000000 | 0.000 | 0.0936352 | 86 | 18 | 15 | 39 | 43 | 41 | 15 | 3 | 4 | 0.0381350 | 0.8240932 | 0.8907109 | 0.0428141 |
| NC\_012920 | 13116 | 0.0000000 | 0.0000000 | 0.0000000 | 0.0000000 | 0.0454300 | 0.0000000 | 0.0000000 | 0.0000000 | 0.000 | 0.0075471 | 86 | 18 | 15 | 39 | 43 | 41 | 15 | 3 | 4 | 0.0195420 | 0.8111513 | 0.9908777 | 0.0197219 |
| NC\_012920 | 13132 | 0.0000000 | 0.0000000 | 0.0000000 | 0.0000000 | 0.0454300 | 0.0000000 | 0.0000000 | 0.0000000 | 0.000 | 0.0075471 | 86 | 18 | 15 | 39 | 43 | 41 | 15 | 3 | 4 | 0.0195420 | 0.8111513 | 0.9908777 | 0.0197219 |
| NC\_012920 | 13135 | 0.0000000 | 0.0000000 | 0.2311111 | 0.0000000 | 0.1297999 | 0.0000000 | 0.0000000 | 0.0000000 | 0.000 | 0.0371614 | 86 | 18 | 15 | 39 | 43 | 41 | 15 | 3 | 4 | 0.0777265 | 0.8141357 | 0.9579026 | 0.0811424 |
| NC\_012920 | 13145 | 0.0673337 | 0.0000000 | 0.0000000 | 0.0000000 | 0.0000000 | 0.0000000 | 0.0000000 | 0.0000000 | 0.000 | 0.0224690 | 86 | 18 | 15 | 39 | 43 | 41 | 15 | 3 | 4 | 0.0237904 | 0.8170914 | 0.9731554 | 0.0244467 |
| NC\_012920 | 13149 | 0.0454300 | 0.0000000 | 0.0000000 | 0.0000000 | 0.0000000 | 0.0000000 | 0.0000000 | 0.0000000 | 0.000 | 0.0150367 | 86 | 18 | 15 | 39 | 43 | 41 | 15 | 3 | 4 | 0.0157998 | 0.8147670 | 0.9818363 | 0.0160920 |
| NC\_012920 | 13152 | 0.0229854 | 0.0000000 | 0.0000000 | 0.0000000 | 0.0000000 | 0.0000000 | 0.0000000 | 0.0000000 | 0.000 | 0.0075471 | 86 | 18 | 15 | 39 | 43 | 41 | 15 | 3 | 4 | 0.0078698 | 0.8123852 | 0.9907831 | 0.0079430 |
| NC\_012920 | 13174 | 0.0229854 | 0.0000000 | 0.0000000 | 0.0000000 | 0.0000000 | 0.0000000 | 0.0000000 | 0.0000000 | 0.000 | 0.0075187 | 86 | 18 | 15 | 39 | 43 | 42 | 15 | 3 | 4 | 0.0078841 | 0.8126166 | 0.9908205 | 0.0079571 |
| NC\_012920 | 13188 | 0.2401298 | 0.0000000 | 0.1244444 | 0.2603550 | 0.1297999 | 0.0907029 | 0.2311111 | 0.4444444 | 0.000 | 0.1830117 | 86 | 18 | 15 | 39 | 43 | 42 | 15 | 3 | 4 | 0.0337233 | 0.8480171 | 0.7914665 | 0.0426087 |
| NC\_012920 | 13194 | 0.0229854 | 0.1049383 | 0.0000000 | 0.0499671 | 0.0000000 | 0.0464853 | 0.0000000 | 0.0000000 | 0.000 | 0.0297330 | 86 | 18 | 15 | 39 | 43 | 42 | 15 | 3 | 4 | 0.0142797 | 0.8153507 | 0.9640542 | 0.0148121 |
| NC\_012920 | 13206 | 0.0000000 | 0.0000000 | 0.0000000 | 0.0499671 | 0.0454300 | 0.0000000 | 0.0000000 | 0.0000000 | 0.000 | 0.0149804 | 86 | 18 | 15 | 39 | 43 | 42 | 15 | 3 | 4 | 0.0170298 | 0.8124742 | 0.9818760 | 0.0173441 |
| NC\_012920 | 13215 | 0.0000000 | 0.0000000 | 0.0000000 | 0.0000000 | 0.0000000 | 0.0464853 | 0.0000000 | 0.0000000 | 0.000 | 0.0075187 | 86 | 18 | 15 | 39 | 43 | 42 | 15 | 3 | 4 | 0.0201118 | 0.8113635 | 0.9909196 | 0.0202961 |
| NC\_012920 | 13236 | 0.0000000 | 0.0000000 | 0.0000000 | 0.0000000 | 0.0454300 | 0.0000000 | 0.0000000 | 0.0000000 | 0.000 | 0.0075187 | 86 | 18 | 15 | 39 | 43 | 42 | 15 | 3 | 4 | 0.0195560 | 0.8113920 | 0.9909148 | 0.0197353 |
| NC\_012920 | 13254 | 0.0000000 | 0.0000000 | 0.1244444 | 0.0000000 | 0.0454300 | 0.0000000 | 0.0000000 | 0.0000000 | 0.000 | 0.0150367 | 86 | 18 | 15 | 39 | 43 | 41 | 15 | 3 | 4 | 0.0376709 | 0.8115530 | 0.9821696 | 0.0383547 |
| NC\_012920 | 13260 | 0.0000000 | 0.1049383 | 0.0000000 | 0.0000000 | 0.0000000 | 0.0000000 | 0.0000000 | 0.0000000 | 0.000 | 0.0075471 | 86 | 18 | 15 | 39 | 43 | 41 | 15 | 3 | 4 | 0.0519645 | 0.8104339 | 0.9911715 | 0.0524274 |
| NC\_012920 | 13276 | 0.0454300 | 0.0000000 | 0.0000000 | 0.0000000 | 0.0454300 | 0.0475907 | 0.0000000 | 0.0000000 | 0.000 | 0.0298439 | 86 | 18 | 15 | 39 | 43 | 41 | 15 | 3 | 4 | 0.0085170 | 0.8171201 | 0.9637878 | 0.0088370 |
| NC\_012920 | 13293 | 0.0000000 | 0.0000000 | 0.0000000 | 0.0000000 | 0.0000000 | 0.0499671 | 0.0000000 | 0.0000000 | 0.000 | 0.0076045 | 86 | 18 | 15 | 39 | 43 | 39 | 15 | 3 | 4 | 0.0219078 | 0.8104714 | 0.9908228 | 0.0221108 |
| NC\_012920 | 13327 | 0.0000000 | 0.0000000 | 0.0000000 | 0.0000000 | 0.0000000 | 0.0499671 | 0.0000000 | 0.0000000 | 0.000 | 0.0076045 | 86 | 18 | 15 | 39 | 43 | 39 | 15 | 3 | 4 | 0.0219078 | 0.8104714 | 0.9908228 | 0.0221108 |
| NC\_012920 | 13359 | 0.0229854 | 0.0000000 | 0.0000000 | 0.0000000 | 0.0000000 | 0.0000000 | 0.0000000 | 0.0000000 | 0.000 | 0.0075471 | 86 | 18 | 15 | 39 | 43 | 41 | 15 | 3 | 4 | 0.0078698 | 0.8123852 | 0.9907831 | 0.0079430 |
| NC\_012920 | 13368 | 0.1297999 | 0.1975309 | 0.2311111 | 0.0499671 | 0.0886966 | 0.0475907 | 0.1244444 | 0.0000000 | 0.000 | 0.1071798 | 86 | 18 | 15 | 39 | 43 | 41 | 15 | 3 | 4 | 0.0187268 | 0.8303776 | 0.8733436 | 0.0214427 |
| NC\_012920 | 13383 | 0.0000000 | 0.0000000 | 0.0000000 | 0.0000000 | 0.0000000 | 0.0475907 | 0.0000000 | 0.0000000 | 0.000 | 0.0075471 | 86 | 18 | 15 | 39 | 43 | 41 | 15 | 3 | 4 | 0.0206807 | 0.8110939 | 0.9908876 | 0.0208709 |
| NC\_012920 | 13392 | 0.0229854 | 0.0000000 | 0.0000000 | 0.0000000 | 0.0000000 | 0.0000000 | 0.0000000 | 0.0000000 | 0.000 | 0.0075471 | 86 | 18 | 15 | 39 | 43 | 41 | 15 | 3 | 4 | 0.0078698 | 0.8123852 | 0.9907831 | 0.0079430 |
| NC\_012920 | 13434 | 0.0229854 | 0.0000000 | 0.2311111 | 0.0000000 | 0.0454300 | 0.0475907 | 0.0000000 | 0.0000000 | 0.000 | 0.0371614 | 86 | 18 | 15 | 39 | 43 | 41 | 15 | 3 | 4 | 0.0471416 | 0.8154844 | 0.9565785 | 0.0492814 |
| NC\_012920 | 13437 | 0.0000000 | 0.0000000 | 0.0000000 | 0.0000000 | 0.0886966 | 0.0000000 | 0.0000000 | 0.0000000 | 0.000 | 0.0150367 | 86 | 18 | 15 | 39 | 43 | 41 | 15 | 3 | 4 | 0.0392331 | 0.8122991 | 0.9822149 | 0.0399435 |
| NC\_012920 | 13470 | 0.0229854 | 0.0000000 | 0.0000000 | 0.0000000 | 0.0000000 | 0.0000000 | 0.3200000 | 0.4444444 | 0.000 | 0.0371614 | 86 | 18 | 15 | 39 | 43 | 41 | 15 | 3 | 4 | 0.1733357 | 0.8134757 | 0.9622361 | 0.1801385 |
| NC\_012920 | 13474 | 0.0229854 | 0.0000000 | 0.0000000 | 0.0000000 | 0.0000000 | 0.0000000 | 0.0000000 | 0.0000000 | 0.000 | 0.0075471 | 86 | 18 | 15 | 39 | 43 | 41 | 15 | 3 | 4 | 0.0078698 | 0.8123852 | 0.9907831 | 0.0079430 |
| NC\_012920 | 13485 | 0.0000000 | 0.0000000 | 0.0000000 | 0.0499671 | 0.0000000 | 0.0475907 | 0.0000000 | 0.0000000 | 0.000 | 0.0150367 | 86 | 18 | 15 | 39 | 43 | 41 | 15 | 3 | 4 | 0.0175730 | 0.8121843 | 0.9818114 | 0.0178986 |
| NC\_012920 | 13488 | 0.0454300 | 0.1049383 | 0.0000000 | 0.0000000 | 0.0000000 | 0.0000000 | 0.0000000 | 0.0000000 | 0.000 | 0.0224690 | 86 | 18 | 15 | 39 | 43 | 41 | 15 | 3 | 4 | 0.0229192 | 0.8152548 | 0.9730710 | 0.0235535 |
| NC\_012920 | 13497 | 0.0000000 | 0.0000000 | 0.0000000 | 0.0000000 | 0.0000000 | 0.0475907 | 0.0000000 | 0.0000000 | 0.000 | 0.0075471 | 86 | 18 | 15 | 39 | 43 | 41 | 15 | 3 | 4 | 0.0206807 | 0.8110939 | 0.9908876 | 0.0208709 |
| NC\_012920 | 13500 | 0.0229854 | 0.0000000 | 0.2311111 | 0.0000000 | 0.0886966 | 0.1356336 | 0.0000000 | 0.4444444 | 0.375 | 0.0728880 | 86 | 18 | 15 | 39 | 43 | 41 | 15 | 3 | 4 | 0.0826689 | 0.8188992 | 0.9183509 | 0.0900189 |
| NC\_012920 | 13506 | 0.0454300 | 0.0000000 | 0.0000000 | 0.0499671 | 0.0454300 | 0.0928019 | 0.0000000 | 0.0000000 | 0.000 | 0.0444215 | 86 | 18 | 15 | 39 | 43 | 41 | 15 | 3 | 4 | 0.0096537 | 0.8193010 | 0.9463046 | 0.0102015 |
| NC\_012920 | 13515 | 0.0229854 | 0.0000000 | 0.0000000 | 0.0000000 | 0.0000000 | 0.0000000 | 0.0000000 | 0.0000000 | 0.000 | 0.0075471 | 86 | 18 | 15 | 39 | 43 | 41 | 15 | 3 | 4 | 0.0078698 | 0.8123852 | 0.9907831 | 0.0079430 |
| NC\_012920 | 13581 | 0.0000000 | 0.0000000 | 0.0000000 | 0.0000000 | 0.0000000 | 0.0475907 | 0.1244444 | 0.0000000 | 0.000 | 0.0150654 | 86 | 18 | 15 | 39 | 43 | 41 | 15 | 3 | 4 | 0.0400743 | 0.8114956 | 0.9821790 | 0.0408015 |
| NC\_012920 | 13590 | 0.0229854 | 0.1975309 | 0.0000000 | 0.0973044 | 0.0454300 | 0.0000000 | 0.1244444 | 0.0000000 | 0.000 | 0.0516242 | 86 | 18 | 15 | 39 | 43 | 41 | 15 | 3 | 4 | 0.0353268 | 0.8170340 | 0.9390472 | 0.0376198 |
| NC\_012920 | 13602 | 0.0000000 | 0.0000000 | 0.0000000 | 0.0000000 | 0.0000000 | 0.0475907 | 0.0000000 | 0.0000000 | 0.000 | 0.0075471 | 86 | 18 | 15 | 39 | 43 | 41 | 15 | 3 | 4 | 0.0206807 | 0.8110939 | 0.9908876 | 0.0208709 |
| NC\_012920 | 13626 | 0.0000000 | 0.0000000 | 0.1244444 | 0.0000000 | 0.0454300 | 0.0000000 | 0.0000000 | 0.0000000 | 0.000 | 0.0150367 | 86 | 18 | 15 | 39 | 43 | 41 | 15 | 3 | 4 | 0.0376709 | 0.8115530 | 0.9821696 | 0.0383547 |
| NC\_012920 | 13635 | 0.0000000 | 0.0000000 | 0.0000000 | 0.0000000 | 0.0454300 | 0.0000000 | 0.0000000 | 0.0000000 | 0.375 | 0.0150935 | 86 | 18 | 15 | 39 | 43 | 40 | 15 | 3 | 4 | 0.1300120 | 0.8109702 | 0.9838081 | 0.1321518 |
| NC\_012920 | 13637 | 0.0229854 | 0.0000000 | 0.0000000 | 0.0499671 | 0.0000000 | 0.0000000 | 0.0000000 | 0.0000000 | 0.375 | 0.0225535 | 86 | 18 | 15 | 39 | 43 | 40 | 15 | 3 | 4 | 0.0853227 | 0.8133123 | 0.9746357 | 0.0875432 |
| NC\_012920 | 13650 | 0.0673337 | 0.1975309 | 0.0000000 | 0.1420118 | 0.0886966 | 0.0950000 | 0.1244444 | 0.0000000 | 0.000 | 0.0939727 | 86 | 18 | 15 | 39 | 43 | 40 | 15 | 3 | 4 | 0.0141411 | 0.8258902 | 0.8878255 | 0.0159278 |
| NC\_012920 | 13653 | 0.0000000 | 0.0000000 | 0.0000000 | 0.0000000 | 0.0886966 | 0.0000000 | 0.0000000 | 0.0000000 | 0.000 | 0.0150935 | 86 | 18 | 15 | 39 | 43 | 40 | 15 | 3 | 4 | 0.0392052 | 0.8120401 | 0.9821416 | 0.0399181 |
| NC\_012920 | 13656 | 0.0000000 | 0.1049383 | 0.0000000 | 0.0499671 | 0.0000000 | 0.0487500 | 0.1244444 | 0.0000000 | 0.000 | 0.0299556 | 86 | 18 | 15 | 39 | 43 | 40 | 15 | 3 | 4 | 0.0284392 | 0.8127919 | 0.9641929 | 0.0294954 |
| NC\_012920 | 13674 | 0.0229854 | 0.0000000 | 0.0000000 | 0.0499671 | 0.0000000 | 0.0000000 | 0.0000000 | 0.0000000 | 0.000 | 0.0150935 | 86 | 18 | 15 | 39 | 43 | 40 | 15 | 3 | 4 | 0.0111144 | 0.8132256 | 0.9816463 | 0.0113222 |
| NC\_012920 | 13695 | 0.0000000 | 0.0000000 | 0.0000000 | 0.0000000 | 0.0000000 | 0.0000000 | 0.1244444 | 0.0000000 | 0.000 | 0.0075756 | 86 | 18 | 15 | 39 | 43 | 40 | 15 | 3 | 4 | 0.0631043 | 0.8100739 | 0.9912383 | 0.0636621 |
| NC\_012920 | 13708 | 0.2879935 | 0.2777778 | 0.1244444 | 0.2235371 | 0.0454300 | 0.0000000 | 0.2311111 | 0.0000000 | 0.375 | 0.1902586 | 86 | 18 | 15 | 39 | 43 | 40 | 15 | 3 | 4 | 0.0552742 | 0.8491376 | 0.7883238 | 0.0701161 |
| NC\_012920 | 13734 | 0.0673337 | 0.2777778 | 0.1244444 | 0.0000000 | 0.0454300 | 0.0928019 | 0.0000000 | 0.0000000 | 0.000 | 0.0728880 | 86 | 18 | 15 | 39 | 43 | 41 | 15 | 3 | 4 | 0.0429620 | 0.8222280 | 0.9151615 | 0.0469447 |
| NC\_012920 | 13743 | 0.0000000 | 0.1049383 | 0.0000000 | 0.0000000 | 0.0454300 | 0.0000000 | 0.0000000 | 0.0000000 | 0.000 | 0.0150367 | 86 | 18 | 15 | 39 | 43 | 41 | 15 | 3 | 4 | 0.0320729 | 0.8116391 | 0.9820678 | 0.0326585 |
| NC\_012920 | 13748 | 0.0229854 | 0.0000000 | 0.0000000 | 0.0000000 | 0.0000000 | 0.0000000 | 0.0000000 | 0.0000000 | 0.000 | 0.0075471 | 86 | 18 | 15 | 39 | 43 | 41 | 15 | 3 | 4 | 0.0078698 | 0.8123852 | 0.9907831 | 0.0079430 |
| NC\_012920 | 13780 | 0.0886966 | 0.0000000 | 0.2311111 | 0.0000000 | 0.0000000 | 0.0475907 | 0.0000000 | 0.0000000 | 0.375 | 0.0587695 | 86 | 18 | 15 | 39 | 43 | 41 | 15 | 3 | 4 | 0.0624782 | 0.8213384 | 0.9329172 | 0.0669708 |
| NC\_012920 | 13785 | 0.0229854 | 0.0000000 | 0.0000000 | 0.0000000 | 0.0000000 | 0.0475907 | 0.0000000 | 0.0000000 | 0.000 | 0.0150367 | 86 | 18 | 15 | 39 | 43 | 41 | 15 | 3 | 4 | 0.0105130 | 0.8135331 | 0.9817111 | 0.0107088 |
| NC\_012920 | 13789 | 0.0000000 | 0.0000000 | 0.0000000 | 0.0499671 | 0.0000000 | 0.0475907 | 0.0000000 | 0.0000000 | 0.000 | 0.0150367 | 86 | 18 | 15 | 39 | 43 | 41 | 15 | 3 | 4 | 0.0175730 | 0.8121843 | 0.9818114 | 0.0178986 |
| NC\_012920 | 13803 | 0.0229854 | 0.1975309 | 0.0000000 | 0.0973044 | 0.0454300 | 0.0000000 | 0.0000000 | 0.0000000 | 0.000 | 0.0444215 | 86 | 18 | 15 | 39 | 43 | 41 | 15 | 3 | 4 | 0.0380833 | 0.8166322 | 0.9476756 | 0.0401860 |
| NC\_012920 | 13812 | 0.0000000 | 0.0000000 | 0.0000000 | 0.0000000 | 0.0000000 | 0.0000000 | 0.1244444 | 0.0000000 | 0.000 | 0.0075471 | 86 | 18 | 15 | 39 | 43 | 41 | 15 | 3 | 4 | 0.0631179 | 0.8103478 | 0.9912745 | 0.0636735 |
| NC\_012920 | 13813 | 0.0229854 | 0.0000000 | 0.0000000 | 0.0000000 | 0.0000000 | 0.0000000 | 0.0000000 | 0.0000000 | 0.000 | 0.0075471 | 86 | 18 | 15 | 39 | 43 | 41 | 15 | 3 | 4 | 0.0078698 | 0.8123852 | 0.9907831 | 0.0079430 |
| NC\_012920 | 13820 | 0.0229854 | 0.0000000 | 0.0000000 | 0.0000000 | 0.0000000 | 0.0000000 | 0.0000000 | 0.0000000 | 0.000 | 0.0075471 | 86 | 18 | 15 | 39 | 43 | 41 | 15 | 3 | 4 | 0.0078698 | 0.8123852 | 0.9907831 | 0.0079430 |
| NC\_012920 | 13827 | 0.0673337 | 0.0000000 | 0.0000000 | 0.0000000 | 0.0000000 | 0.0000000 | 0.0000000 | 0.0000000 | 0.000 | 0.0224690 | 86 | 18 | 15 | 39 | 43 | 41 | 15 | 3 | 4 | 0.0237904 | 0.8170914 | 0.9731554 | 0.0244467 |
| NC\_012920 | 13866 | 0.0229854 | 0.0000000 | 0.0000000 | 0.0000000 | 0.0000000 | 0.0000000 | 0.0000000 | 0.0000000 | 0.000 | 0.0075471 | 86 | 18 | 15 | 39 | 43 | 41 | 15 | 3 | 4 | 0.0078698 | 0.8123852 | 0.9907831 | 0.0079430 |
| NC\_012920 | 13879 | 0.0229854 | 0.0000000 | 0.0000000 | 0.0499671 | 0.0000000 | 0.0487500 | 0.1244444 | 0.0000000 | 0.000 | 0.0299556 | 86 | 18 | 15 | 39 | 43 | 40 | 15 | 3 | 4 | 0.0172877 | 0.8147581 | 0.9638693 | 0.0179357 |
| NC\_012920 | 13885 | 0.0229854 | 0.0000000 | 0.0000000 | 0.0000000 | 0.0000000 | 0.0000000 | 0.0000000 | 0.0000000 | 0.000 | 0.0075756 | 86 | 18 | 15 | 39 | 43 | 40 | 15 | 3 | 4 | 0.0078555 | 0.8121268 | 0.9907451 | 0.0079289 |
| NC\_012920 | 13886 | 0.0000000 | 0.0000000 | 0.0000000 | 0.0499671 | 0.0454300 | 0.0000000 | 0.0000000 | 0.0000000 | 0.000 | 0.0150935 | 86 | 18 | 15 | 39 | 43 | 40 | 15 | 3 | 4 | 0.0169729 | 0.8119822 | 0.9817271 | 0.0172888 |
| NC\_012920 | 13914 | 0.0229854 | 0.0000000 | 0.2311111 | 0.0000000 | 0.0000000 | 0.0487500 | 0.0000000 | 0.4444444 | 0.000 | 0.0372999 | 86 | 18 | 15 | 39 | 43 | 40 | 15 | 3 | 4 | 0.1104133 | 0.8140641 | 0.9592397 | 0.1151050 |
| NC\_012920 | 13919 | 0.0454300 | 0.0000000 | 0.0000000 | 0.0000000 | 0.0454300 | 0.0000000 | 0.0000000 | 0.0000000 | 0.000 | 0.0225535 | 86 | 18 | 15 | 39 | 43 | 40 | 15 | 3 | 4 | 0.0119857 | 0.8157412 | 0.9726836 | 0.0123223 |
| NC\_012920 | 13933 | 0.0886966 | 0.1049383 | 0.0000000 | 0.0499671 | 0.0000000 | 0.0000000 | 0.0000000 | 0.0000000 | 0.000 | 0.0445864 | 86 | 18 | 15 | 39 | 43 | 40 | 15 | 3 | 4 | 0.0222341 | 0.8207434 | 0.9468834 | 0.0234813 |
| NC\_012920 | 13934 | 0.0454300 | 0.1975309 | 0.0000000 | 0.0000000 | 0.0000000 | 0.0000000 | 0.0000000 | 0.0000000 | 0.000 | 0.0299556 | 86 | 18 | 15 | 39 | 43 | 40 | 15 | 3 | 4 | 0.0527770 | 0.8154520 | 0.9652038 | 0.0546797 |
| NC\_012920 | 13966 | 0.0454300 | 0.0000000 | 0.0000000 | 0.0000000 | 0.0000000 | 0.0950000 | 0.0000000 | 0.0000000 | 0.000 | 0.0299556 | 86 | 18 | 15 | 39 | 43 | 40 | 15 | 3 | 4 | 0.0217496 | 0.8167243 | 0.9641200 | 0.0225590 |
| NC\_012920 | 13971 | 0.0000000 | 0.0000000 | 0.0000000 | 0.0000000 | 0.0000000 | 0.0000000 | 0.0000000 | 0.0000000 | 0.375 | 0.0075756 | 86 | 18 | 15 | 39 | 43 | 40 | 15 | 3 | 4 | 0.2471374 | 0.8097558 | 0.9929566 | 0.2488904 |
| NC\_012920 | 13983 | 0.0229854 | 0.0000000 | 0.0000000 | 0.0000000 | 0.0000000 | 0.0000000 | 0.0000000 | 0.0000000 | 0.000 | 0.0075756 | 86 | 18 | 15 | 39 | 43 | 40 | 15 | 3 | 4 | 0.0078555 | 0.8121268 | 0.9907451 | 0.0079289 |
| NC\_012920 | 14000 | 0.0000000 | 0.0000000 | 0.0000000 | 0.0499671 | 0.0000000 | 0.0487500 | 0.0000000 | 0.0000000 | 0.000 | 0.0150935 | 86 | 18 | 15 | 39 | 43 | 40 | 15 | 3 | 4 | 0.0178517 | 0.8118955 | 0.9817415 | 0.0181837 |
| NC\_012920 | 14007 | 0.0229854 | 0.0000000 | 0.0000000 | 0.0000000 | 0.0000000 | 0.0000000 | 0.0000000 | 0.0000000 | 0.000 | 0.0076045 | 86 | 18 | 15 | 39 | 43 | 39 | 15 | 3 | 4 | 0.0078410 | 0.8118408 | 0.9907065 | 0.0079146 |
| NC\_012920 | 14020 | 0.0673337 | 0.1049383 | 0.0000000 | 0.0499671 | 0.0000000 | 0.0000000 | 0.0000000 | 0.0000000 | 0.000 | 0.0374395 | 86 | 18 | 15 | 39 | 43 | 39 | 15 | 3 | 4 | 0.0184374 | 0.8182215 | 0.9550864 | 0.0193045 |
| NC\_012920 | 14028 | 0.0000000 | 0.0000000 | 0.0000000 | 0.0499671 | 0.0000000 | 0.0000000 | 0.0000000 | 0.0000000 | 0.000 | 0.0076045 | 86 | 18 | 15 | 39 | 43 | 39 | 15 | 3 | 4 | 0.0219078 | 0.8104714 | 0.9908228 | 0.0221108 |
| NC\_012920 | 14040 | 0.0000000 | 0.0000000 | 0.0000000 | 0.0000000 | 0.0454300 | 0.0000000 | 0.0000000 | 0.0000000 | 0.375 | 0.0151506 | 86 | 18 | 15 | 39 | 43 | 39 | 15 | 3 | 4 | 0.1299866 | 0.8106754 | 0.9837404 | 0.1321350 |
| NC\_012920 | 14053 | 0.0000000 | 0.0000000 | 0.0000000 | 0.0000000 | 0.0454300 | 0.0499671 | 0.1244444 | 0.0000000 | 0.000 | 0.0226385 | 86 | 18 | 15 | 39 | 43 | 39 | 15 | 3 | 4 | 0.0273843 | 0.8121030 | 0.9728869 | 0.0281475 |
| NC\_012920 | 14058 | 0.0454300 | 0.0000000 | 0.0000000 | 0.0000000 | 0.0000000 | 0.0000000 | 0.0000000 | 0.0000000 | 0.000 | 0.0151506 | 86 | 18 | 15 | 39 | 43 | 39 | 15 | 3 | 4 | 0.0157424 | 0.8142591 | 0.9816863 | 0.0160361 |
| NC\_012920 | 14070 | 0.0673337 | 0.1049383 | 0.1244444 | 0.0499671 | 0.0454300 | 0.0000000 | 0.1244444 | 0.0000000 | 0.000 | 0.0592040 | 86 | 18 | 15 | 39 | 43 | 39 | 15 | 3 | 4 | 0.0126567 | 0.8202611 | 0.9287365 | 0.0136279 |
| NC\_012920 | 14094 | 0.1095187 | 0.0000000 | 0.0000000 | 0.0000000 | 0.0000000 | 0.0000000 | 0.0000000 | 0.4444444 | 0.000 | 0.0447526 | 86 | 18 | 15 | 39 | 43 | 39 | 15 | 3 | 4 | 0.0830053 | 0.8212225 | 0.9500282 | 0.0873714 |
| NC\_012920 | 14110 | 0.0229854 | 0.1049383 | 0.0000000 | 0.0000000 | 0.0886966 | 0.0000000 | 0.0000000 | 0.0000000 | 0.000 | 0.0300682 | 86 | 18 | 15 | 39 | 43 | 39 | 15 | 3 | 4 | 0.0251688 | 0.8147252 | 0.9640230 | 0.0261081 |
| NC\_012920 | 14118 | 0.0000000 | 0.0000000 | 0.0000000 | 0.0000000 | 0.0000000 | 0.0499671 | 0.0000000 | 0.0000000 | 0.000 | 0.0076045 | 86 | 18 | 15 | 39 | 43 | 39 | 15 | 3 | 4 | 0.0219078 | 0.8104714 | 0.9908228 | 0.0221108 |
| NC\_012920 | 14128 | 0.0000000 | 0.0000000 | 0.0000000 | 0.0000000 | 0.0000000 | 0.0499671 | 0.0000000 | 0.0000000 | 0.000 | 0.0076045 | 86 | 18 | 15 | 39 | 43 | 39 | 15 | 3 | 4 | 0.0219078 | 0.8104714 | 0.9908228 | 0.0221108 |
| NC\_012920 | 14139 | 0.0454300 | 0.1049383 | 0.0000000 | 0.0000000 | 0.0454300 | 0.0000000 | 0.0000000 | 0.0000000 | 0.000 | 0.0300682 | 86 | 18 | 15 | 39 | 43 | 39 | 15 | 3 | 4 | 0.0163126 | 0.8159781 | 0.9637519 | 0.0169262 |
| NC\_012920 | 14148 | 0.0000000 | 0.0000000 | 0.0000000 | 0.0000000 | 0.0000000 | 0.0499671 | 0.0000000 | 0.0000000 | 0.000 | 0.0076045 | 86 | 18 | 15 | 39 | 43 | 39 | 15 | 3 | 4 | 0.0219078 | 0.8104714 | 0.9908228 | 0.0221108 |
| NC\_012920 | 14161 | 0.0000000 | 0.0000000 | 0.0000000 | 0.0000000 | 0.0000000 | 0.0499671 | 0.0000000 | 0.0000000 | 0.000 | 0.0076045 | 86 | 18 | 15 | 39 | 43 | 39 | 15 | 3 | 4 | 0.0219078 | 0.8104714 | 0.9908228 | 0.0221108 |
| NC\_012920 | 14167 | 0.0886966 | 0.0000000 | 0.0000000 | 0.4260355 | 0.3309897 | 0.0000000 | 0.1244444 | 0.0000000 | 0.000 | 0.1787775 | 86 | 18 | 15 | 39 | 43 | 39 | 15 | 3 | 4 | 0.1387109 | 0.8376843 | 0.8161848 | 0.1699504 |
| NC\_012920 | 14178 | 0.0229854 | 0.0000000 | 0.0000000 | 0.0499671 | 0.0000000 | 0.0499671 | 0.0000000 | 0.0000000 | 0.000 | 0.0226385 | 86 | 18 | 15 | 39 | 43 | 39 | 15 | 3 | 4 | 0.0096299 | 0.8140551 | 0.9724582 | 0.0099026 |
| NC\_012920 | 14179 | 0.0454300 | 0.0000000 | 0.0000000 | 0.0000000 | 0.0000000 | 0.0000000 | 0.1244444 | 0.0000000 | 0.000 | 0.0226385 | 86 | 18 | 15 | 39 | 43 | 39 | 15 | 3 | 4 | 0.0265801 | 0.8146670 | 0.9729499 | 0.0273191 |
| NC\_012920 | 14198 | 0.0000000 | 0.0000000 | 0.0000000 | 0.0000000 | 0.0454300 | 0.0000000 | 0.0000000 | 0.0000000 | 0.000 | 0.0076045 | 86 | 18 | 15 | 39 | 43 | 39 | 15 | 3 | 4 | 0.0195135 | 0.8105880 | 0.9908017 | 0.0196947 |
| NC\_012920 | 14207 | 0.0229854 | 0.0000000 | 0.0000000 | 0.0000000 | 0.0000000 | 0.0499671 | 0.0000000 | 0.0000000 | 0.000 | 0.0151506 | 86 | 18 | 15 | 39 | 43 | 39 | 15 | 3 | 4 | 0.0110855 | 0.8129480 | 0.9815699 | 0.0112936 |
| NC\_012920 | 14208 | 0.0000000 | 0.0000000 | 0.1244444 | 0.0000000 | 0.0000000 | 0.0000000 | 0.0000000 | 0.0000000 | 0.000 | 0.0076045 | 86 | 18 | 15 | 39 | 43 | 39 | 15 | 3 | 4 | 0.0630907 | 0.8097722 | 0.9912016 | 0.0636507 |
| NC\_012920 | 14233 | 0.1297999 | 0.1049383 | 0.2311111 | 0.0000000 | 0.0454300 | 0.0487500 | 0.1244444 | 0.0000000 | 0.000 | 0.0870910 | 86 | 18 | 15 | 39 | 43 | 40 | 15 | 3 | 4 | 0.0269130 | 0.8275383 | 0.8975913 | 0.0299836 |
| NC\_012920 | 14257 | 0.0229854 | 0.0000000 | 0.0000000 | 0.0000000 | 0.0000000 | 0.0000000 | 0.0000000 | 0.0000000 | 0.000 | 0.0075756 | 86 | 18 | 15 | 39 | 43 | 40 | 15 | 3 | 4 | 0.0078555 | 0.8121268 | 0.9907451 | 0.0079289 |
| NC\_012920 | 14284 | 0.0000000 | 0.0000000 | 0.0000000 | 0.0499671 | 0.0454300 | 0.0000000 | 0.0000000 | 0.0000000 | 0.000 | 0.0150935 | 86 | 18 | 15 | 39 | 43 | 40 | 15 | 3 | 4 | 0.0169729 | 0.8119822 | 0.9817271 | 0.0172888 |
| NC\_012920 | 14287 | 0.0000000 | 0.0000000 | 0.0000000 | 0.0000000 | 0.0000000 | 0.0487500 | 0.0000000 | 0.0000000 | 0.000 | 0.0075756 | 86 | 18 | 15 | 39 | 43 | 40 | 15 | 3 | 4 | 0.0212786 | 0.8107967 | 0.9908554 | 0.0214750 |
| NC\_012920 | 14302 | 0.0229854 | 0.0000000 | 0.0000000 | 0.0000000 | 0.0886966 | 0.0000000 | 0.0000000 | 0.0000000 | 0.000 | 0.0225535 | 86 | 18 | 15 | 39 | 43 | 40 | 15 | 3 | 4 | 0.0237478 | 0.8144978 | 0.9729676 | 0.0244076 |
| NC\_012920 | 14305 | 0.0229854 | 0.0000000 | 0.2311111 | 0.0000000 | 0.0454300 | 0.0950000 | 0.0000000 | 0.4444444 | 0.000 | 0.0518151 | 86 | 18 | 15 | 39 | 43 | 40 | 15 | 3 | 4 | 0.0805103 | 0.8163484 | 0.9416383 | 0.0855002 |
| NC\_012920 | 14308 | 0.0454300 | 0.0000000 | 0.0000000 | 0.0000000 | 0.0454300 | 0.0000000 | 0.0000000 | 0.0000000 | 0.000 | 0.0225535 | 86 | 18 | 15 | 39 | 43 | 40 | 15 | 3 | 4 | 0.0119857 | 0.8157412 | 0.9726836 | 0.0123223 |
| NC\_012920 | 14311 | 0.0000000 | 0.0000000 | 0.0000000 | 0.0000000 | 0.0000000 | 0.0487500 | 0.0000000 | 0.0000000 | 0.000 | 0.0075756 | 86 | 18 | 15 | 39 | 43 | 40 | 15 | 3 | 4 | 0.0212786 | 0.8107967 | 0.9908554 | 0.0214750 |
| NC\_012920 | 14319 | 0.0886966 | 0.0000000 | 0.0000000 | 0.0000000 | 0.0000000 | 0.0000000 | 0.0000000 | 0.0000000 | 0.000 | 0.0299556 | 86 | 18 | 15 | 39 | 43 | 40 | 15 | 3 | 4 | 0.0317859 | 0.8191531 | 0.9645934 | 0.0329527 |
| NC\_012920 | 14323 | 0.0454300 | 0.0000000 | 0.0000000 | 0.0973044 | 0.0886966 | 0.0000000 | 0.1244444 | 0.0000000 | 0.000 | 0.0518151 | 86 | 18 | 15 | 39 | 43 | 40 | 15 | 3 | 4 | 0.0179717 | 0.8194422 | 0.9379042 | 0.0191616 |
| NC\_012920 | 14353 | 0.0000000 | 0.0000000 | 0.0000000 | 0.0499671 | 0.0000000 | 0.0000000 | 0.0000000 | 0.0000000 | 0.000 | 0.0076045 | 86 | 18 | 15 | 39 | 43 | 39 | 15 | 3 | 4 | 0.0219078 | 0.8104714 | 0.9908228 | 0.0221108 |
| NC\_012920 | 14364 | 0.0229854 | 0.1049383 | 0.1244444 | 0.0499671 | 0.0454300 | 0.0000000 | 0.1244444 | 0.0000000 | 0.000 | 0.0447526 | 86 | 18 | 15 | 39 | 43 | 39 | 15 | 3 | 4 | 0.0191057 | 0.8154828 | 0.9461698 | 0.0201927 |
| NC\_012920 | 14368 | 0.0000000 | 0.0000000 | 0.0000000 | 0.0000000 | 0.0454300 | 0.0000000 | 0.0000000 | 0.0000000 | 0.000 | 0.0076045 | 86 | 18 | 15 | 39 | 43 | 39 | 15 | 3 | 4 | 0.0195135 | 0.8105880 | 0.9908017 | 0.0196947 |
| NC\_012920 | 14384 | 0.0229854 | 0.0000000 | 0.0000000 | 0.0000000 | 0.0000000 | 0.0000000 | 0.0000000 | 0.0000000 | 0.000 | 0.0076045 | 86 | 18 | 15 | 39 | 43 | 39 | 15 | 3 | 4 | 0.0078410 | 0.8118408 | 0.9907065 | 0.0079146 |
| NC\_012920 | 14470 | 0.0673337 | 0.0000000 | 0.0000000 | 0.0000000 | 0.0000000 | 0.0475907 | 0.0000000 | 0.0000000 | 0.000 | 0.0298439 | 86 | 18 | 15 | 39 | 43 | 41 | 15 | 3 | 4 | 0.0173720 | 0.8182392 | 0.9641603 | 0.0180178 |
| NC\_012920 | 14476 | 0.0000000 | 0.0000000 | 0.0000000 | 0.0000000 | 0.0000000 | 0.0475907 | 0.0000000 | 0.0000000 | 0.000 | 0.0075471 | 86 | 18 | 15 | 39 | 43 | 41 | 15 | 3 | 4 | 0.0206807 | 0.8110939 | 0.9908876 | 0.0208709 |
| NC\_012920 | 14500 | 0.0000000 | 0.1049383 | 0.0000000 | 0.0000000 | 0.0000000 | 0.0000000 | 0.0000000 | 0.0000000 | 0.000 | 0.0075756 | 86 | 18 | 15 | 39 | 43 | 40 | 15 | 3 | 4 | 0.0519508 | 0.8101606 | 0.9911350 | 0.0524155 |
| NC\_012920 | 14518 | 0.0000000 | 0.0000000 | 0.1244444 | 0.0000000 | 0.0000000 | 0.0000000 | 0.0000000 | 0.0000000 | 0.000 | 0.0075756 | 86 | 18 | 15 | 39 | 43 | 40 | 15 | 3 | 4 | 0.0631043 | 0.8100739 | 0.9912383 | 0.0636621 |
| NC\_012920 | 14544 | 0.0000000 | 0.0000000 | 0.0000000 | 0.0000000 | 0.0454300 | 0.0000000 | 0.0000000 | 0.0000000 | 0.375 | 0.0150935 | 86 | 18 | 15 | 39 | 43 | 40 | 15 | 3 | 4 | 0.1300120 | 0.8109702 | 0.9838081 | 0.1321518 |
| NC\_012920 | 14553 | 0.0000000 | 0.0000000 | 0.0000000 | 0.0000000 | 0.0454300 | 0.0000000 | 0.0000000 | 0.0000000 | 0.000 | 0.0075756 | 86 | 18 | 15 | 39 | 43 | 40 | 15 | 3 | 4 | 0.0195278 | 0.8108835 | 0.9908400 | 0.0197083 |
| NC\_012920 | 14560 | 0.0229854 | 0.0000000 | 0.0000000 | 0.0499671 | 0.0000000 | 0.0950000 | 0.0000000 | 0.0000000 | 0.000 | 0.0299556 | 86 | 18 | 15 | 39 | 43 | 40 | 15 | 3 | 4 | 0.0194032 | 0.8154231 | 0.9639765 | 0.0201283 |
| NC\_012920 | 14566 | 0.0229854 | 0.1975309 | 0.0000000 | 0.0973044 | 0.0454300 | 0.0000000 | 0.0000000 | 0.0000000 | 0.000 | 0.0445864 | 86 | 18 | 15 | 39 | 43 | 40 | 15 | 3 | 4 | 0.0379982 | 0.8164062 | 0.9474621 | 0.0401052 |
| NC\_012920 | 14569 | 0.0229854 | 0.0000000 | 0.0000000 | 0.0000000 | 0.0886966 | 0.0487500 | 0.0000000 | 0.0000000 | 0.375 | 0.0372999 | 86 | 18 | 15 | 39 | 43 | 40 | 15 | 3 | 4 | 0.0580219 | 0.8157122 | 0.9569263 | 0.0606336 |
| NC\_012920 | 14577 | 0.0000000 | 0.0000000 | 0.1244444 | 0.0000000 | 0.0454300 | 0.0000000 | 0.0000000 | 0.0000000 | 0.000 | 0.0150935 | 86 | 18 | 15 | 39 | 43 | 40 | 15 | 3 | 4 | 0.0376429 | 0.8112883 | 0.9820960 | 0.0383292 |
| NC\_012920 | 14582 | 0.0000000 | 0.0000000 | 0.0000000 | 0.0000000 | 0.0000000 | 0.0000000 | 0.1244444 | 0.0000000 | 0.000 | 0.0076045 | 86 | 18 | 15 | 39 | 43 | 39 | 15 | 3 | 4 | 0.0630907 | 0.8097722 | 0.9912016 | 0.0636507 |
| NC\_012920 | 14620 | 0.0000000 | 0.0000000 | 0.0000000 | 0.0000000 | 0.0000000 | 0.0000000 | 0.1244444 | 0.0000000 | 0.000 | 0.0076045 | 86 | 18 | 15 | 39 | 43 | 39 | 15 | 3 | 4 | 0.0630907 | 0.8097722 | 0.9912016 | 0.0636507 |
| NC\_012920 | 14668 | 0.0000000 | 0.0000000 | 0.0000000 | 0.0000000 | 0.0000000 | 0.0487500 | 0.0000000 | 0.0000000 | 0.000 | 0.0075756 | 86 | 18 | 15 | 39 | 43 | 40 | 15 | 3 | 4 | 0.0212786 | 0.8107967 | 0.9908554 | 0.0214750 |
| NC\_012920 | 14687 | 0.0000000 | 0.1049383 | 0.0000000 | 0.0000000 | 0.0000000 | 0.0000000 | 0.0000000 | 0.0000000 | 0.000 | 0.0075471 | 86 | 18 | 15 | 39 | 43 | 41 | 15 | 3 | 4 | 0.0519645 | 0.8104339 | 0.9911715 | 0.0524274 |
| NC\_012920 | 14751 | 0.0000000 | 0.0000000 | 0.0000000 | 0.0000000 | 0.0454300 | 0.0000000 | 0.0000000 | 0.0000000 | 0.000 | 0.0075756 | 86 | 18 | 15 | 39 | 43 | 40 | 15 | 3 | 4 | 0.0195278 | 0.8108835 | 0.9908400 | 0.0197083 |
| NC\_012920 | 14755 | 0.0229854 | 0.0000000 | 0.0000000 | 0.0000000 | 0.0454300 | 0.0000000 | 0.0000000 | 0.0000000 | 0.000 | 0.0150935 | 86 | 18 | 15 | 39 | 43 | 40 | 15 | 3 | 4 | 0.0099127 | 0.8133412 | 0.9816266 | 0.0100982 |
| NC\_012920 | 14766 | 0.2566252 | 0.2777778 | 0.3200000 | 0.1420118 | 0.2055165 | 0.1800000 | 0.3200000 | 0.0000000 | 0.000 | 0.2251298 | 86 | 18 | 15 | 39 | 43 | 40 | 15 | 3 | 4 | 0.0162760 | 0.8532724 | 0.7404515 | 0.0219812 |
| NC\_012920 | 14769 | 0.0229854 | 0.0000000 | 0.0000000 | 0.0000000 | 0.0000000 | 0.0950000 | 0.0000000 | 0.0000000 | 0.000 | 0.0225535 | 86 | 18 | 15 | 39 | 43 | 40 | 15 | 3 | 4 | 0.0261002 | 0.8143243 | 0.9730270 | 0.0268237 |
| NC\_012920 | 14783 | 0.1297999 | 0.1049383 | 0.3200000 | 0.2235371 | 0.4218496 | 0.2187500 | 0.0000000 | 0.0000000 | 0.000 | 0.2194625 | 86 | 18 | 15 | 39 | 43 | 40 | 15 | 3 | 4 | 0.0737971 | 0.8463329 | 0.7598264 | 0.0971236 |
| NC\_012920 | 14798 | 0.0886966 | 0.0000000 | 0.1244444 | 0.4260355 | 0.3569497 | 0.0000000 | 0.1244444 | 0.0000000 | 0.000 | 0.1896235 | 86 | 18 | 15 | 39 | 43 | 41 | 15 | 3 | 4 | 0.1345398 | 0.8389291 | 0.8043796 | 0.1672591 |
| NC\_012920 | 14845 | 0.0000000 | 0.0000000 | 0.0000000 | 0.0000000 | 0.0000000 | 0.0000000 | 0.1244444 | 0.0000000 | 0.000 | 0.0075471 | 86 | 18 | 15 | 39 | 43 | 41 | 15 | 3 | 4 | 0.0631179 | 0.8103478 | 0.9912745 | 0.0636735 |
| NC\_012920 | 14866 | 0.0000000 | 0.0000000 | 0.0000000 | 0.0000000 | 0.0000000 | 0.0000000 | 0.1244444 | 0.0000000 | 0.000 | 0.0075471 | 86 | 18 | 15 | 39 | 43 | 41 | 15 | 3 | 4 | 0.0631179 | 0.8103478 | 0.9912745 | 0.0636735 |
| NC\_012920 | 14872 | 0.0454300 | 0.0000000 | 0.0000000 | 0.0000000 | 0.0000000 | 0.0000000 | 0.1244444 | 0.0000000 | 0.000 | 0.0224690 | 86 | 18 | 15 | 39 | 43 | 41 | 15 | 3 | 4 | 0.0266655 | 0.8151687 | 0.9731714 | 0.0274006 |
| NC\_012920 | 14884 | 0.0000000 | 0.0000000 | 0.0000000 | 0.0000000 | 0.0000000 | 0.0475907 | 0.0000000 | 0.0000000 | 0.000 | 0.0075471 | 86 | 18 | 15 | 39 | 43 | 41 | 15 | 3 | 4 | 0.0206807 | 0.8110939 | 0.9908876 | 0.0208709 |
| NC\_012920 | 14905 | 0.1297999 | 0.1975309 | 0.2311111 | 0.0000000 | 0.0886966 | 0.0928019 | 0.1244444 | 0.0000000 | 0.000 | 0.1071798 | 86 | 18 | 15 | 39 | 43 | 41 | 15 | 3 | 4 | 0.0220864 | 0.8303776 | 0.8737773 | 0.0252769 |
| NC\_012920 | 14911 | 0.0000000 | 0.0000000 | 0.0000000 | 0.0499671 | 0.0000000 | 0.0475907 | 0.0000000 | 0.0000000 | 0.000 | 0.0150367 | 86 | 18 | 15 | 39 | 43 | 41 | 15 | 3 | 4 | 0.0175730 | 0.8121843 | 0.9818114 | 0.0178986 |
| NC\_012920 | 14927 | 0.0000000 | 0.0000000 | 0.0000000 | 0.0499671 | 0.0000000 | 0.0000000 | 0.0000000 | 0.0000000 | 0.000 | 0.0075471 | 86 | 18 | 15 | 39 | 43 | 41 | 15 | 3 | 4 | 0.0219362 | 0.8110365 | 0.9908987 | 0.0221377 |
| NC\_012920 | 14935 | 0.0229854 | 0.0000000 | 0.0000000 | 0.0000000 | 0.0454300 | 0.0000000 | 0.0000000 | 0.0000000 | 0.000 | 0.0150367 | 86 | 18 | 15 | 39 | 43 | 41 | 15 | 3 | 4 | 0.0099414 | 0.8135904 | 0.9817018 | 0.0101267 |
| NC\_012920 | 14953 | 0.0454300 | 0.0000000 | 0.0000000 | 0.0000000 | 0.0000000 | 0.0000000 | 0.0000000 | 0.0000000 | 0.000 | 0.0149804 | 86 | 18 | 15 | 39 | 43 | 42 | 15 | 3 | 4 | 0.0158281 | 0.8149804 | 0.9819096 | 0.0161197 |
| NC\_012920 | 14968 | 0.0000000 | 0.0000000 | 0.0000000 | 0.0000000 | 0.0454300 | 0.0000000 | 0.0000000 | 0.0000000 | 0.000 | 0.0075187 | 86 | 18 | 15 | 39 | 43 | 42 | 15 | 3 | 4 | 0.0195560 | 0.8113920 | 0.9909148 | 0.0197353 |
| NC\_012920 | 14990 | 0.0000000 | 0.0000000 | 0.0000000 | 0.0000000 | 0.0000000 | 0.0000000 | 0.0000000 | 0.0000000 | 0.375 | 0.0075187 | 86 | 18 | 15 | 39 | 43 | 42 | 15 | 3 | 4 | 0.2471591 | 0.8102812 | 0.9930143 | 0.2488978 |
| NC\_012920 | 15019 | 0.0000000 | 0.0000000 | 0.0000000 | 0.0000000 | 0.0000000 | 0.0000000 | 0.2311111 | 0.0000000 | 0.000 | 0.0150367 | 86 | 18 | 15 | 39 | 43 | 41 | 15 | 3 | 4 | 0.1267176 | 0.8106921 | 0.9838023 | 0.1288039 |
| NC\_012920 | 15043 | 0.2230936 | 0.1049383 | 0.4444444 | 0.2235371 | 0.4391563 | 0.2498513 | 0.0000000 | 0.0000000 | 0.000 | 0.2675620 | 86 | 18 | 15 | 39 | 43 | 41 | 15 | 3 | 4 | 0.0714814 | 0.8580980 | 0.7104803 | 0.1006100 |
| NC\_012920 | 15049 | 0.0229854 | 0.1049383 | 0.0000000 | 0.0499671 | 0.0000000 | 0.0475907 | 0.0000000 | 0.0000000 | 0.000 | 0.0298439 | 86 | 18 | 15 | 39 | 43 | 41 | 15 | 3 | 4 | 0.0143699 | 0.8151113 | 0.9639129 | 0.0149079 |
| NC\_012920 | 15055 | 0.0229854 | 0.0000000 | 0.0000000 | 0.0000000 | 0.0000000 | 0.0000000 | 0.0000000 | 0.0000000 | 0.000 | 0.0075471 | 86 | 18 | 15 | 39 | 43 | 41 | 15 | 3 | 4 | 0.0078698 | 0.8123852 | 0.9907831 | 0.0079430 |
| NC\_012920 | 15061 | 0.0229854 | 0.0000000 | 0.0000000 | 0.0000000 | 0.0454300 | 0.0000000 | 0.0000000 | 0.0000000 | 0.000 | 0.0150367 | 86 | 18 | 15 | 39 | 43 | 41 | 15 | 3 | 4 | 0.0099414 | 0.8135904 | 0.9817018 | 0.0101267 |
| NC\_012920 | 15064 | 0.0229854 | 0.0000000 | 0.0000000 | 0.0000000 | 0.0000000 | 0.0000000 | 0.0000000 | 0.0000000 | 0.000 | 0.0075471 | 86 | 18 | 15 | 39 | 43 | 41 | 15 | 3 | 4 | 0.0078698 | 0.8123852 | 0.9907831 | 0.0079430 |
| NC\_012920 | 15094 | 0.0000000 | 0.0000000 | 0.0000000 | 0.0000000 | 0.0000000 | 0.0464853 | 0.0000000 | 0.0000000 | 0.000 | 0.0075187 | 86 | 18 | 15 | 39 | 43 | 42 | 15 | 3 | 4 | 0.0201118 | 0.8113635 | 0.9909196 | 0.0202961 |
| NC\_012920 | 15110 | 0.0000000 | 0.0000000 | 0.0000000 | 0.0499671 | 0.0000000 | 0.0000000 | 0.0000000 | 0.0000000 | 0.000 | 0.0075187 | 86 | 18 | 15 | 39 | 43 | 42 | 15 | 3 | 4 | 0.0219503 | 0.8112780 | 0.9909357 | 0.0221511 |
| NC\_012920 | 15133 | 0.0000000 | 0.0000000 | 0.0000000 | 0.0000000 | 0.0000000 | 0.0464853 | 0.0000000 | 0.0000000 | 0.000 | 0.0075187 | 86 | 18 | 15 | 39 | 43 | 42 | 15 | 3 | 4 | 0.0201118 | 0.8113635 | 0.9909196 | 0.0202961 |
| NC\_012920 | 15136 | 0.0454300 | 0.0000000 | 0.0000000 | 0.0000000 | 0.0454300 | 0.0000000 | 0.0000000 | 0.0000000 | 0.000 | 0.0223852 | 86 | 18 | 15 | 39 | 43 | 42 | 15 | 3 | 4 | 0.0120717 | 0.8161766 | 0.9729042 | 0.0124079 |
| NC\_012920 | 15148 | 0.0454300 | 0.1049383 | 0.1244444 | 0.0499671 | 0.0454300 | 0.0464853 | 0.1244444 | 0.0000000 | 0.000 | 0.0585546 | 86 | 18 | 15 | 39 | 43 | 42 | 15 | 3 | 4 | 0.0085838 | 0.8197081 | 0.9291796 | 0.0092381 |
| NC\_012920 | 15172 | 0.0454300 | 0.0000000 | 0.0000000 | 0.0000000 | 0.0000000 | 0.0464853 | 0.0000000 | 0.0000000 | 0.000 | 0.0223852 | 86 | 18 | 15 | 39 | 43 | 42 | 15 | 3 | 4 | 0.0122584 | 0.8161481 | 0.9729084 | 0.0125998 |
| NC\_012920 | 15178 | 0.0000000 | 0.0000000 | 0.0000000 | 0.0499671 | 0.0000000 | 0.0000000 | 0.0000000 | 0.0000000 | 0.000 | 0.0075187 | 86 | 18 | 15 | 39 | 43 | 42 | 15 | 3 | 4 | 0.0219503 | 0.8112780 | 0.9909357 | 0.0221511 |
| NC\_012920 | 15190 | 0.0229854 | 0.0000000 | 0.0000000 | 0.0000000 | 0.0000000 | 0.0464853 | 0.0000000 | 0.0000000 | 0.000 | 0.0149804 | 86 | 18 | 15 | 39 | 43 | 42 | 15 | 3 | 4 | 0.0102489 | 0.8137843 | 0.9817803 | 0.0104391 |
| NC\_012920 | 15204 | 0.0000000 | 0.0000000 | 0.0000000 | 0.0000000 | 0.0000000 | 0.0000000 | 0.0000000 | 0.0000000 | 0.375 | 0.0075187 | 86 | 18 | 15 | 39 | 43 | 42 | 15 | 3 | 4 | 0.2471591 | 0.8102812 | 0.9930143 | 0.2488978 |
| NC\_012920 | 15214 | 0.0229854 | 0.0000000 | 0.0000000 | 0.0000000 | 0.0000000 | 0.0000000 | 0.0000000 | 0.0000000 | 0.000 | 0.0075187 | 86 | 18 | 15 | 39 | 43 | 42 | 15 | 3 | 4 | 0.0078841 | 0.8126166 | 0.9908205 | 0.0079571 |
| NC\_012920 | 15217 | 0.0673337 | 0.0000000 | 0.0000000 | 0.0000000 | 0.0000000 | 0.0000000 | 0.0000000 | 0.0000000 | 0.000 | 0.0223852 | 86 | 18 | 15 | 39 | 43 | 42 | 15 | 3 | 4 | 0.0238328 | 0.8172873 | 0.9732631 | 0.0244875 |
| NC\_012920 | 15226 | 0.0000000 | 0.0000000 | 0.0000000 | 0.0499671 | 0.0000000 | 0.0000000 | 0.0000000 | 0.0000000 | 0.000 | 0.0075187 | 86 | 18 | 15 | 39 | 43 | 42 | 15 | 3 | 4 | 0.0219503 | 0.8112780 | 0.9909357 | 0.0221511 |
| NC\_012920 | 15236 | 0.0000000 | 0.0000000 | 0.0000000 | 0.0000000 | 0.0454300 | 0.0464853 | 0.0000000 | 0.0000000 | 0.000 | 0.0149804 | 86 | 18 | 15 | 39 | 43 | 42 | 15 | 3 | 4 | 0.0161071 | 0.8125596 | 0.9818609 | 0.0164046 |
| NC\_012920 | 15244 | 0.0229854 | 0.0000000 | 0.0000000 | 0.0000000 | 0.0000000 | 0.0000000 | 0.0000000 | 0.0000000 | 0.000 | 0.0075187 | 86 | 18 | 15 | 39 | 43 | 42 | 15 | 3 | 4 | 0.0078841 | 0.8126166 | 0.9908205 | 0.0079571 |
| NC\_012920 | 15257 | 0.1297999 | 0.0000000 | 0.1244444 | 0.0499671 | 0.0000000 | 0.0000000 | 0.0000000 | 0.0000000 | 0.000 | 0.0587695 | 86 | 18 | 15 | 39 | 43 | 41 | 15 | 3 | 4 | 0.0346098 | 0.8252124 | 0.9312474 | 0.0371650 |
| NC\_012920 | 15259 | 0.0000000 | 0.0000000 | 0.1244444 | 0.0000000 | 0.0000000 | 0.0000000 | 0.0000000 | 0.0000000 | 0.000 | 0.0075471 | 86 | 18 | 15 | 39 | 43 | 41 | 15 | 3 | 4 | 0.0631179 | 0.8103478 | 0.9912745 | 0.0636735 |
| NC\_012920 | 15262 | 0.0229854 | 0.0000000 | 0.0000000 | 0.0499671 | 0.0000000 | 0.0000000 | 0.0000000 | 0.0000000 | 0.000 | 0.0150367 | 86 | 18 | 15 | 39 | 43 | 41 | 15 | 3 | 4 | 0.0111431 | 0.8134757 | 0.9817214 | 0.0113506 |
| NC\_012920 | 15263 | 0.0000000 | 0.0000000 | 0.0000000 | 0.0000000 | 0.0000000 | 0.0000000 | 0.1244444 | 0.0000000 | 0.000 | 0.0075471 | 86 | 18 | 15 | 39 | 43 | 41 | 15 | 3 | 4 | 0.0631179 | 0.8103478 | 0.9912745 | 0.0636735 |
| NC\_012920 | 15265 | 0.0454300 | 0.0000000 | 0.0000000 | 0.0000000 | 0.0000000 | 0.0000000 | 0.0000000 | 0.0000000 | 0.000 | 0.0150935 | 86 | 18 | 15 | 39 | 43 | 40 | 15 | 3 | 4 | 0.0157712 | 0.8145267 | 0.9817619 | 0.0160642 |
| NC\_012920 | 15283 | 0.0000000 | 0.0000000 | 0.0000000 | 0.0000000 | 0.0454300 | 0.0000000 | 0.0000000 | 0.0000000 | 0.000 | 0.0075756 | 86 | 18 | 15 | 39 | 43 | 40 | 15 | 3 | 4 | 0.0195278 | 0.8108835 | 0.9908400 | 0.0197083 |
| NC\_012920 | 15289 | 0.0000000 | 0.0000000 | 0.0000000 | 0.0000000 | 0.0454300 | 0.0000000 | 0.0000000 | 0.0000000 | 0.000 | 0.0075471 | 86 | 18 | 15 | 39 | 43 | 41 | 15 | 3 | 4 | 0.0195420 | 0.8111513 | 0.9908777 | 0.0197219 |
| NC\_012920 | 15301 | 0.2055165 | 0.2777778 | 0.3200000 | 0.3261012 | 0.4542996 | 0.2777778 | 0.3911111 | 0.0000000 | 0.000 | 0.3061588 | 86 | 18 | 15 | 39 | 43 | 42 | 15 | 3 | 4 | 0.0477184 | 0.8614026 | 0.6615411 | 0.0721321 |
| NC\_012920 | 15307 | 0.0000000 | 0.0000000 | 0.0000000 | 0.0000000 | 0.0454300 | 0.0000000 | 0.0000000 | 0.0000000 | 0.000 | 0.0075187 | 86 | 18 | 15 | 39 | 43 | 42 | 15 | 3 | 4 | 0.0195560 | 0.8113920 | 0.9909148 | 0.0197353 |
| NC\_012920 | 15314 | 0.0229854 | 0.0000000 | 0.0000000 | 0.0000000 | 0.0000000 | 0.0000000 | 0.0000000 | 0.0000000 | 0.000 | 0.0075187 | 86 | 18 | 15 | 39 | 43 | 42 | 15 | 3 | 4 | 0.0078841 | 0.8126166 | 0.9908205 | 0.0079571 |
| NC\_012920 | 15315 | 0.0229854 | 0.0000000 | 0.0000000 | 0.0000000 | 0.0000000 | 0.0000000 | 0.0000000 | 0.0000000 | 0.000 | 0.0075187 | 86 | 18 | 15 | 39 | 43 | 42 | 15 | 3 | 4 | 0.0078841 | 0.8126166 | 0.9908205 | 0.0079571 |
| NC\_012920 | 15326 | 0.0229854 | 0.0000000 | 0.0000000 | 0.0000000 | 0.0000000 | 0.0464853 | 0.0000000 | 0.0000000 | 0.000 | 0.0149804 | 86 | 18 | 15 | 39 | 43 | 42 | 15 | 3 | 4 | 0.0102489 | 0.8137843 | 0.9817803 | 0.0104391 |
| NC\_012920 | 15327 | 0.0000000 | 0.0000000 | 0.0000000 | 0.0000000 | 0.0000000 | 0.0000000 | 0.1244444 | 0.0000000 | 0.000 | 0.0075187 | 86 | 18 | 15 | 39 | 43 | 42 | 15 | 3 | 4 | 0.0631313 | 0.8105945 | 0.9913101 | 0.0636847 |
| NC\_012920 | 15355 | 0.0000000 | 0.0000000 | 0.0000000 | 0.0000000 | 0.0000000 | 0.0464853 | 0.0000000 | 0.0000000 | 0.000 | 0.0075187 | 86 | 18 | 15 | 39 | 43 | 42 | 15 | 3 | 4 | 0.0201118 | 0.8113635 | 0.9909196 | 0.0202961 |
| NC\_012920 | 15367 | 0.0229854 | 0.0000000 | 0.0000000 | 0.0000000 | 0.0000000 | 0.0000000 | 0.0000000 | 0.0000000 | 0.000 | 0.0075471 | 86 | 18 | 15 | 39 | 43 | 41 | 15 | 3 | 4 | 0.0078698 | 0.8123852 | 0.9907831 | 0.0079430 |
| NC\_012920 | 15379 | 0.0000000 | 0.0000000 | 0.0000000 | 0.0000000 | 0.0454300 | 0.0000000 | 0.0000000 | 0.0000000 | 0.000 | 0.0075471 | 86 | 18 | 15 | 39 | 43 | 41 | 15 | 3 | 4 | 0.0195420 | 0.8111513 | 0.9908777 | 0.0197219 |
| NC\_012920 | 15383 | 0.0229854 | 0.0000000 | 0.0000000 | 0.0000000 | 0.0000000 | 0.0000000 | 0.0000000 | 0.0000000 | 0.000 | 0.0075471 | 86 | 18 | 15 | 39 | 43 | 41 | 15 | 3 | 4 | 0.0078698 | 0.8123852 | 0.9907831 | 0.0079430 |
| NC\_012920 | 15385 | 0.0000000 | 0.0000000 | 0.0000000 | 0.0000000 | 0.0454300 | 0.0000000 | 0.0000000 | 0.0000000 | 0.375 | 0.0150367 | 86 | 18 | 15 | 39 | 43 | 41 | 15 | 3 | 4 | 0.1300373 | 0.8112374 | 0.9838748 | 0.1321685 |
| NC\_012920 | 15402 | 0.0229854 | 0.0000000 | 0.0000000 | 0.0000000 | 0.0000000 | 0.0000000 | 0.0000000 | 0.0000000 | 0.000 | 0.0075471 | 86 | 18 | 15 | 39 | 43 | 41 | 15 | 3 | 4 | 0.0078698 | 0.8123852 | 0.9907831 | 0.0079430 |
| NC\_012920 | 15418 | 0.0000000 | 0.0000000 | 0.0000000 | 0.0000000 | 0.0000000 | 0.0475907 | 0.0000000 | 0.0000000 | 0.000 | 0.0075471 | 86 | 18 | 15 | 39 | 43 | 41 | 15 | 3 | 4 | 0.0206807 | 0.8110939 | 0.9908876 | 0.0208709 |
| NC\_012920 | 15431 | 0.0454300 | 0.0000000 | 0.1244444 | 0.0499671 | 0.0454300 | 0.0475907 | 0.0000000 | 0.0000000 | 0.000 | 0.0444215 | 86 | 18 | 15 | 39 | 43 | 41 | 15 | 3 | 4 | 0.0085445 | 0.8186123 | 0.9461993 | 0.0090303 |
| NC\_012920 | 15434 | 0.0000000 | 0.0000000 | 0.0000000 | 0.0499671 | 0.0000000 | 0.0000000 | 0.0000000 | 0.0000000 | 0.000 | 0.0075471 | 86 | 18 | 15 | 39 | 43 | 41 | 15 | 3 | 4 | 0.0219362 | 0.8110365 | 0.9908987 | 0.0221377 |
| NC\_012920 | 15448 | 0.0000000 | 0.0000000 | 0.0000000 | 0.0000000 | 0.0000000 | 0.0000000 | 0.2311111 | 0.0000000 | 0.000 | 0.0150367 | 86 | 18 | 15 | 39 | 43 | 41 | 15 | 3 | 4 | 0.1267176 | 0.8106921 | 0.9838023 | 0.1288039 |
| NC\_012920 | 15452 | 0.3442401 | 0.4012346 | 0.3200000 | 0.2235371 | 0.0886966 | 0.0475907 | 0.3200000 | 0.0000000 | 0.375 | 0.2518079 | 86 | 18 | 15 | 39 | 43 | 41 | 15 | 3 | 4 | 0.0611830 | 0.8588728 | 0.7247538 | 0.0844190 |
| NC\_012920 | 15453 | 0.0229854 | 0.0000000 | 0.0000000 | 0.0000000 | 0.0000000 | 0.0000000 | 0.0000000 | 0.0000000 | 0.000 | 0.0075471 | 86 | 18 | 15 | 39 | 43 | 41 | 15 | 3 | 4 | 0.0078698 | 0.8123852 | 0.9907831 | 0.0079430 |
| NC\_012920 | 15454 | 0.0454300 | 0.1049383 | 0.0000000 | 0.0000000 | 0.0454300 | 0.0000000 | 0.0000000 | 0.0000000 | 0.000 | 0.0298439 | 86 | 18 | 15 | 39 | 43 | 41 | 15 | 3 | 4 | 0.0164281 | 0.8164601 | 0.9640477 | 0.0170408 |
| NC\_012920 | 15458 | 0.0000000 | 0.0000000 | 0.0000000 | 0.0000000 | 0.0000000 | 0.0000000 | 0.1244444 | 0.0000000 | 0.000 | 0.0075471 | 86 | 18 | 15 | 39 | 43 | 41 | 15 | 3 | 4 | 0.0631179 | 0.8103478 | 0.9912745 | 0.0636735 |
| NC\_012920 | 15459 | 0.0229854 | 0.0000000 | 0.0000000 | 0.0000000 | 0.0000000 | 0.0000000 | 0.0000000 | 0.0000000 | 0.000 | 0.0075471 | 86 | 18 | 15 | 39 | 43 | 41 | 15 | 3 | 4 | 0.0078698 | 0.8123852 | 0.9907831 | 0.0079430 |
| NC\_012920 | 15470 | 0.0229854 | 0.0000000 | 0.0000000 | 0.0000000 | 0.0000000 | 0.0000000 | 0.0000000 | 0.0000000 | 0.000 | 0.0075471 | 86 | 18 | 15 | 39 | 43 | 41 | 15 | 3 | 4 | 0.0078698 | 0.8123852 | 0.9907831 | 0.0079430 |
| NC\_012920 | 15479 | 0.0000000 | 0.0000000 | 0.0000000 | 0.0499671 | 0.0000000 | 0.0000000 | 0.0000000 | 0.0000000 | 0.000 | 0.0075471 | 86 | 18 | 15 | 39 | 43 | 41 | 15 | 3 | 4 | 0.0219362 | 0.8110365 | 0.9908987 | 0.0221377 |
| NC\_012920 | 15481 | 0.0229854 | 0.0000000 | 0.0000000 | 0.0000000 | 0.0000000 | 0.0000000 | 0.0000000 | 0.0000000 | 0.000 | 0.0075471 | 86 | 18 | 15 | 39 | 43 | 41 | 15 | 3 | 4 | 0.0078698 | 0.8123852 | 0.9907831 | 0.0079430 |
| NC\_012920 | 15511 | 0.0000000 | 0.0000000 | 0.0000000 | 0.0499671 | 0.0000000 | 0.0000000 | 0.0000000 | 0.0000000 | 0.000 | 0.0075471 | 86 | 18 | 15 | 39 | 43 | 41 | 15 | 3 | 4 | 0.0219362 | 0.8110365 | 0.9908987 | 0.0221377 |
| NC\_012920 | 15514 | 0.0000000 | 0.0000000 | 0.0000000 | 0.0000000 | 0.0000000 | 0.0907029 | 0.0000000 | 0.0000000 | 0.000 | 0.0149804 | 86 | 18 | 15 | 39 | 43 | 42 | 15 | 3 | 4 | 0.0403766 | 0.8124742 | 0.9823064 | 0.0411039 |
| NC\_012920 | 15562 | 0.0000000 | 0.1049383 | 0.0000000 | 0.0000000 | 0.0000000 | 0.0000000 | 0.0000000 | 0.0000000 | 0.000 | 0.0075471 | 86 | 18 | 15 | 39 | 43 | 41 | 15 | 3 | 4 | 0.0519645 | 0.8104339 | 0.9911715 | 0.0524274 |
| NC\_012920 | 15607 | 0.1297999 | 0.1975309 | 0.2311111 | 0.0000000 | 0.0886966 | 0.0475907 | 0.1244444 | 0.0000000 | 0.000 | 0.1004362 | 86 | 18 | 15 | 39 | 43 | 41 | 15 | 3 | 4 | 0.0263358 | 0.8292872 | 0.8820781 | 0.0298566 |
| NC\_012920 | 15626 | 0.0229854 | 0.0000000 | 0.0000000 | 0.0000000 | 0.0000000 | 0.0000000 | 0.0000000 | 0.0000000 | 0.000 | 0.0075471 | 86 | 18 | 15 | 39 | 43 | 41 | 15 | 3 | 4 | 0.0078698 | 0.8123852 | 0.9907831 | 0.0079430 |
| NC\_012920 | 15629 | 0.0229854 | 0.0000000 | 0.0000000 | 0.0000000 | 0.0000000 | 0.0000000 | 0.0000000 | 0.0000000 | 0.000 | 0.0075471 | 86 | 18 | 15 | 39 | 43 | 41 | 15 | 3 | 4 | 0.0078698 | 0.8123852 | 0.9907831 | 0.0079430 |
| NC\_012920 | 15631 | 0.0229854 | 0.0000000 | 0.0000000 | 0.0000000 | 0.0000000 | 0.0000000 | 0.0000000 | 0.0000000 | 0.000 | 0.0075471 | 86 | 18 | 15 | 39 | 43 | 41 | 15 | 3 | 4 | 0.0078698 | 0.8123852 | 0.9907831 | 0.0079430 |
| NC\_012920 | 15650 | 0.0000000 | 0.0000000 | 0.0000000 | 0.0000000 | 0.0000000 | 0.0475907 | 0.0000000 | 0.0000000 | 0.000 | 0.0075471 | 86 | 18 | 15 | 39 | 43 | 41 | 15 | 3 | 4 | 0.0206807 | 0.8110939 | 0.9908876 | 0.0208709 |
| NC\_012920 | 15652 | 0.0000000 | 0.0000000 | 0.0000000 | 0.0499671 | 0.0000000 | 0.0000000 | 0.0000000 | 0.0000000 | 0.000 | 0.0075471 | 86 | 18 | 15 | 39 | 43 | 41 | 15 | 3 | 4 | 0.0219362 | 0.8110365 | 0.9908987 | 0.0221377 |
| NC\_012920 | 15657 | 0.0000000 | 0.0000000 | 0.0000000 | 0.0000000 | 0.0000000 | 0.0475907 | 0.0000000 | 0.0000000 | 0.000 | 0.0075471 | 86 | 18 | 15 | 39 | 43 | 41 | 15 | 3 | 4 | 0.0206807 | 0.8110939 | 0.9908876 | 0.0208709 |
| NC\_012920 | 15663 | 0.0000000 | 0.0000000 | 0.0000000 | 0.0499671 | 0.0000000 | 0.0000000 | 0.0000000 | 0.0000000 | 0.000 | 0.0075471 | 86 | 18 | 15 | 39 | 43 | 41 | 15 | 3 | 4 | 0.0219362 | 0.8110365 | 0.9908987 | 0.0221377 |
| NC\_012920 | 15670 | 0.0000000 | 0.0000000 | 0.0000000 | 0.0499671 | 0.0000000 | 0.0000000 | 0.0000000 | 0.0000000 | 0.000 | 0.0075471 | 86 | 18 | 15 | 39 | 43 | 41 | 15 | 3 | 4 | 0.0219362 | 0.8110365 | 0.9908987 | 0.0221377 |
| NC\_012920 | 15672 | 0.0229854 | 0.0000000 | 0.0000000 | 0.0000000 | 0.0000000 | 0.0475907 | 0.0000000 | 0.0000000 | 0.000 | 0.0150367 | 86 | 18 | 15 | 39 | 43 | 41 | 15 | 3 | 4 | 0.0105130 | 0.8135331 | 0.9817111 | 0.0107088 |
| NC\_012920 | 15674 | 0.1297999 | 0.0000000 | 0.0000000 | 0.1420118 | 0.1297999 | 0.0475907 | 0.0000000 | 0.0000000 | 0.000 | 0.0936352 | 86 | 18 | 15 | 39 | 43 | 41 | 15 | 3 | 4 | 0.0196536 | 0.8314107 | 0.8895913 | 0.0220928 |
| NC\_012920 | 15679 | 0.1095187 | 0.0000000 | 0.1244444 | 0.0000000 | 0.0000000 | 0.0000000 | 0.0000000 | 0.0000000 | 0.000 | 0.0444215 | 86 | 18 | 15 | 39 | 43 | 41 | 15 | 3 | 4 | 0.0376900 | 0.8219697 | 0.9479941 | 0.0397577 |
| NC\_012920 | 15693 | 0.0000000 | 0.0000000 | 0.0000000 | 0.0000000 | 0.0000000 | 0.0000000 | 0.1244444 | 0.0000000 | 0.000 | 0.0075756 | 86 | 18 | 15 | 39 | 43 | 40 | 15 | 3 | 4 | 0.0631043 | 0.8100739 | 0.9912383 | 0.0636621 |
| NC\_012920 | 15703 | 0.0000000 | 0.0000000 | 0.0000000 | 0.0000000 | 0.0000000 | 0.0000000 | 0.1244444 | 0.0000000 | 0.000 | 0.0075756 | 86 | 18 | 15 | 39 | 43 | 40 | 15 | 3 | 4 | 0.0631043 | 0.8100739 | 0.9912383 | 0.0636621 |
| NC\_012920 | 15712 | 0.0454300 | 0.0000000 | 0.0000000 | 0.0000000 | 0.0000000 | 0.0000000 | 0.0000000 | 0.0000000 | 0.000 | 0.0150935 | 86 | 18 | 15 | 39 | 43 | 40 | 15 | 3 | 4 | 0.0157712 | 0.8145267 | 0.9817619 | 0.0160642 |
| NC\_012920 | 15718 | 0.0000000 | 0.0000000 | 0.0000000 | 0.0000000 | 0.0000000 | 0.0000000 | 0.0000000 | 0.4444444 | 0.000 | 0.0075756 | 86 | 18 | 15 | 39 | 43 | 40 | 15 | 3 | 4 | 0.3307888 | 0.8097269 | 0.9937390 | 0.3328729 |
| NC\_012920 | 15721 | 0.0229854 | 0.0000000 | 0.0000000 | 0.0000000 | 0.0000000 | 0.0000000 | 0.0000000 | 0.0000000 | 0.000 | 0.0075756 | 86 | 18 | 15 | 39 | 43 | 40 | 15 | 3 | 4 | 0.0078555 | 0.8121268 | 0.9907451 | 0.0079289 |
| NC\_012920 | 15734 | 0.0000000 | 0.0000000 | 0.0000000 | 0.0000000 | 0.0454300 | 0.0000000 | 0.0000000 | 0.0000000 | 0.000 | 0.0075756 | 86 | 18 | 15 | 39 | 43 | 40 | 15 | 3 | 4 | 0.0195278 | 0.8108835 | 0.9908400 | 0.0197083 |
| NC\_012920 | 15735 | 0.0000000 | 0.0000000 | 0.0000000 | 0.0973044 | 0.0454300 | 0.0000000 | 0.0000000 | 0.0000000 | 0.000 | 0.0225535 | 86 | 18 | 15 | 39 | 43 | 40 | 15 | 3 | 4 | 0.0308854 | 0.8130232 | 0.9731165 | 0.0317387 |
| NC\_012920 | 15746 | 0.0229854 | 0.0000000 | 0.0000000 | 0.0000000 | 0.0000000 | 0.0475907 | 0.0000000 | 0.0000000 | 0.000 | 0.0150367 | 86 | 18 | 15 | 39 | 43 | 41 | 15 | 3 | 4 | 0.0105130 | 0.8135331 | 0.9817111 | 0.0107088 |
| NC\_012920 | 15758 | 0.0229854 | 0.0000000 | 0.0000000 | 0.0499671 | 0.0000000 | 0.0000000 | 0.0000000 | 0.0000000 | 0.000 | 0.0150367 | 86 | 18 | 15 | 39 | 43 | 41 | 15 | 3 | 4 | 0.0111431 | 0.8134757 | 0.9817214 | 0.0113506 |
| NC\_012920 | 15759 | 0.0000000 | 0.1049383 | 0.0000000 | 0.0000000 | 0.0000000 | 0.0000000 | 0.0000000 | 0.0000000 | 0.000 | 0.0075471 | 86 | 18 | 15 | 39 | 43 | 41 | 15 | 3 | 4 | 0.0519645 | 0.8104339 | 0.9911715 | 0.0524274 |
| NC\_012920 | 15766 | 0.0000000 | 0.0000000 | 0.0000000 | 0.0000000 | 0.0000000 | 0.0000000 | 0.1244444 | 0.0000000 | 0.000 | 0.0075471 | 86 | 18 | 15 | 39 | 43 | 41 | 15 | 3 | 4 | 0.0631179 | 0.8103478 | 0.9912745 | 0.0636735 |
| NC\_012920 | 15777 | 0.0000000 | 0.0000000 | 0.0000000 | 0.0000000 | 0.0000000 | 0.0000000 | 0.1244444 | 0.0000000 | 0.000 | 0.0075756 | 86 | 18 | 15 | 39 | 43 | 40 | 15 | 3 | 4 | 0.0631043 | 0.8100739 | 0.9912383 | 0.0636621 |
| NC\_012920 | 15784 | 0.0229854 | 0.1975309 | 0.0000000 | 0.0000000 | 0.0000000 | 0.0000000 | 0.0000000 | 0.0000000 | 0.000 | 0.0225535 | 86 | 18 | 15 | 39 | 43 | 40 | 15 | 3 | 4 | 0.0673110 | 0.8130521 | 0.9741279 | 0.0690987 |
| NC\_012920 | 15796 | 0.0000000 | 0.0000000 | 0.0000000 | 0.0000000 | 0.0000000 | 0.0000000 | 0.1244444 | 0.0000000 | 0.000 | 0.0075756 | 86 | 18 | 15 | 39 | 43 | 40 | 15 | 3 | 4 | 0.0631043 | 0.8100739 | 0.9912383 | 0.0636621 |
| NC\_012920 | 15799 | 0.0000000 | 0.0000000 | 0.0000000 | 0.0499671 | 0.0000000 | 0.0000000 | 0.0000000 | 0.0000000 | 0.000 | 0.0075756 | 86 | 18 | 15 | 39 | 43 | 40 | 15 | 3 | 4 | 0.0219221 | 0.8107678 | 0.9908610 | 0.0221243 |
| NC\_012920 | 15812 | 0.0229854 | 0.0000000 | 0.0000000 | 0.0499671 | 0.0000000 | 0.0000000 | 0.0000000 | 0.0000000 | 0.000 | 0.0150935 | 86 | 18 | 15 | 39 | 43 | 40 | 15 | 3 | 4 | 0.0111144 | 0.8132256 | 0.9816463 | 0.0113222 |
| NC\_012920 | 15833 | 0.0000000 | 0.1049383 | 0.0000000 | 0.0000000 | 0.0000000 | 0.0000000 | 0.0000000 | 0.0000000 | 0.000 | 0.0075471 | 86 | 18 | 15 | 39 | 43 | 41 | 15 | 3 | 4 | 0.0519645 | 0.8104339 | 0.9911715 | 0.0524274 |
| NC\_012920 | 15884 | 0.0000000 | 0.0000000 | 0.1244444 | 0.0000000 | 0.0000000 | 0.0475907 | 0.0000000 | 0.0000000 | 0.000 | 0.0150654 | 86 | 18 | 15 | 39 | 43 | 41 | 15 | 3 | 4 | 0.0400743 | 0.8114956 | 0.9821790 | 0.0408015 |
| NC\_012920 | 15905 | 0.0000000 | 0.0000000 | 0.0000000 | 0.0499671 | 0.0000000 | 0.0000000 | 0.0000000 | 0.0000000 | 0.000 | 0.0075471 | 86 | 18 | 15 | 39 | 43 | 41 | 15 | 3 | 4 | 0.0219362 | 0.8110365 | 0.9908987 | 0.0221377 |
| NC\_012920 | 15907 | 0.0673337 | 0.2777778 | 0.1244444 | 0.0000000 | 0.0454300 | 0.0475907 | 0.0000000 | 0.0000000 | 0.000 | 0.0658574 | 86 | 18 | 15 | 39 | 43 | 41 | 15 | 3 | 4 | 0.0474102 | 0.8211375 | 0.9235997 | 0.0513320 |
| NC\_012920 | 15924 | 0.1873986 | 0.0000000 | 0.1244444 | 0.0499671 | 0.0454300 | 0.1356336 | 0.1244444 | 0.0000000 | 0.000 | 0.1138659 | 86 | 18 | 15 | 39 | 43 | 41 | 15 | 3 | 4 | 0.0248768 | 0.8362029 | 0.8672173 | 0.0286858 |
| NC\_012920 | 15928 | 0.1297999 | 0.1975309 | 0.2311111 | 0.0000000 | 0.0886966 | 0.0464853 | 0.1244444 | 0.0000000 | 0.000 | 0.1000783 | 86 | 18 | 15 | 39 | 43 | 42 | 15 | 3 | 4 | 0.0264978 | 0.8294197 | 0.8825366 | 0.0300245 |
| NC\_012920 | 15930 | 0.0000000 | 0.1049383 | 0.1244444 | 0.0499671 | 0.0000000 | 0.0907029 | 0.0000000 | 0.0000000 | 0.000 | 0.0370239 | 86 | 18 | 15 | 39 | 43 | 42 | 15 | 3 | 4 | 0.0303245 | 0.8144393 | 0.9559192 | 0.0317229 |
| NC\_012920 | 15941 | 0.0000000 | 0.0000000 | 0.0000000 | 0.0000000 | 0.0000000 | 0.0000000 | 0.1244444 | 0.0000000 | 0.000 | 0.0075187 | 86 | 18 | 15 | 39 | 43 | 42 | 15 | 3 | 4 | 0.0631313 | 0.8105945 | 0.9913101 | 0.0636847 |
| NC\_012920 | 15954 | 0.0454300 | 0.1049383 | 0.1244444 | 0.0499671 | 0.0454300 | 0.0000000 | 0.1244444 | 0.0000000 | 0.000 | 0.0514347 | 86 | 18 | 15 | 39 | 43 | 42 | 15 | 3 | 4 | 0.0145841 | 0.8185404 | 0.9380794 | 0.0155467 |
| NC\_012920 | 15968 | 0.0000000 | 0.0000000 | 0.0000000 | 0.0000000 | 0.0000000 | 0.0907029 | 0.0000000 | 0.0000000 | 0.000 | 0.0149804 | 86 | 18 | 15 | 39 | 43 | 42 | 15 | 3 | 4 | 0.0403766 | 0.8124742 | 0.9823064 | 0.0411039 |
| NC\_012920 | 15978 | 0.0000000 | 0.0000000 | 0.0000000 | 0.0499671 | 0.0000000 | 0.0000000 | 0.0000000 | 0.0000000 | 0.000 | 0.0075187 | 86 | 18 | 15 | 39 | 43 | 42 | 15 | 3 | 4 | 0.0219503 | 0.8112780 | 0.9909357 | 0.0221511 |
| NC\_012920 | 16048 | 0.0000000 | 0.0000000 | 0.1244444 | 0.0000000 | 0.0000000 | 0.0000000 | 0.0000000 | 0.0000000 | 0.000 | 0.0075187 | 86 | 18 | 15 | 39 | 43 | 42 | 15 | 3 | 4 | 0.0631313 | 0.8105945 | 0.9913101 | 0.0636847 |
| NC\_012920 | 16051 | 0.1687399 | 0.3456790 | 0.2311111 | 0.0499671 | 0.0886966 | 0.1326531 | 0.0000000 | 0.4444444 | 0.000 | 0.1459309 | 86 | 18 | 15 | 39 | 43 | 42 | 15 | 3 | 4 | 0.0466426 | 0.8371093 | 0.8338039 | 0.0559395 |
| NC\_012920 | 16069 | 0.2566252 | 0.2777778 | 0.1244444 | 0.2235371 | 0.0000000 | 0.0000000 | 0.2311111 | 0.0000000 | 0.375 | 0.1708793 | 86 | 18 | 15 | 39 | 43 | 42 | 15 | 3 | 4 | 0.0587852 | 0.8445710 | 0.8095671 | 0.0726131 |
| NC\_012920 | 16071 | 0.0229854 | 0.0000000 | 0.2311111 | 0.0000000 | 0.0000000 | 0.0475907 | 0.1244444 | 0.4444444 | 0.000 | 0.0444215 | 86 | 18 | 15 | 39 | 43 | 41 | 15 | 3 | 4 | 0.0965819 | 0.8147383 | 0.9507435 | 0.1015857 |
| NC\_012920 | 16093 | 0.1297999 | 0.1975309 | 0.0000000 | 0.2603550 | 0.0886966 | 0.1760857 | 0.2311111 | 0.0000000 | 0.375 | 0.1590622 | 86 | 18 | 15 | 39 | 43 | 41 | 15 | 3 | 4 | 0.0266738 | 0.8377525 | 0.8151967 | 0.0327207 |
| NC\_012920 | 16124 | 0.0454300 | 0.0000000 | 0.0000000 | 0.0973044 | 0.0886966 | 0.0000000 | 0.0000000 | 0.0000000 | 0.000 | 0.0444215 | 86 | 18 | 15 | 39 | 43 | 41 | 15 | 3 | 4 | 0.0180324 | 0.8192436 | 0.9467552 | 0.0190465 |
| NC\_012920 | 16126 | 0.4469984 | 0.3456790 | 0.3911111 | 0.4260355 | 0.2401298 | 0.1356336 | 0.4444444 | 0.4444444 | 0.375 | 0.3711834 | 86 | 18 | 15 | 39 | 43 | 41 | 15 | 3 | 4 | 0.0557159 | 0.8807679 | 0.6020488 | 0.0925438 |
| NC\_012920 | 16129 | 0.1911844 | 0.2037037 | 0.2400000 | 0.0973044 | 0.1719849 | 0.1796550 | 0.0000000 | 0.0000000 | 0.000 | 0.1619892 | 86 | 18 | 15 | 39 | 43 | 41 | 15 | 3 | 4 | 0.0117046 | 0.8429752 | 0.8100855 | 0.0144486 |
| NC\_012920 | 16145 | 0.1873986 | 0.1975309 | 0.0000000 | 0.0973044 | 0.1297999 | 0.0928019 | 0.2311111 | 0.0000000 | 0.375 | 0.1464360 | 86 | 18 | 15 | 39 | 43 | 41 | 15 | 3 | 4 | 0.0217140 | 0.8393882 | 0.8293326 | 0.0261825 |
| NC\_012920 | 16146 | 0.1095187 | 0.0000000 | 0.0000000 | 0.0000000 | 0.0000000 | 0.0000000 | 0.0000000 | 0.0000000 | 0.000 | 0.0371614 | 86 | 18 | 15 | 39 | 43 | 41 | 15 | 3 | 4 | 0.0399569 | 0.8215680 | 0.9565751 | 0.0417708 |
| NC\_012920 | 16168 | 0.0229854 | 0.0000000 | 0.0000000 | 0.0000000 | 0.0000000 | 0.0000000 | 0.0000000 | 0.0000000 | 0.000 | 0.0076045 | 86 | 18 | 15 | 39 | 42 | 40 | 15 | 3 | 4 | 0.0078410 | 0.8119282 | 0.9907075 | 0.0079146 |
| NC\_012920 | 16172 | 0.0454300 | 0.1049383 | 0.1244444 | 0.0000000 | 0.0907029 | 0.0000000 | 0.1244444 | 0.0000000 | 0.000 | 0.0518151 | 86 | 18 | 15 | 39 | 42 | 41 | 15 | 3 | 4 | 0.0211819 | 0.8181989 | 0.9380131 | 0.0225817 |
| NC\_012920 | 16176 | 0.0000000 | 0.0000000 | 0.0000000 | 0.0000000 | 0.0000000 | 0.0475907 | 0.0000000 | 0.0000000 | 0.000 | 0.0076922 | 84 | 17 | 15 | 38 | 42 | 41 | 15 | 3 | 4 | 0.0206088 | 0.8117351 | 0.9907191 | 0.0208019 |
| NC\_012920 | 16192 | 0.0000000 | 0.0000000 | 0.0000000 | 0.0000000 | 0.0000000 | 0.0475907 | 0.0000000 | 0.0000000 | 0.375 | 0.0150367 | 86 | 18 | 15 | 39 | 43 | 41 | 15 | 3 | 4 | 0.1306088 | 0.8111800 | 0.9838842 | 0.1327482 |
| NC\_012920 | 16201 | 0.0229854 | 0.0000000 | 0.0000000 | 0.0000000 | 0.0000000 | 0.0000000 | 0.0000000 | 0.0000000 | 0.375 | 0.0150367 | 86 | 18 | 15 | 39 | 43 | 41 | 15 | 3 | 4 | 0.1241789 | 0.8124713 | 0.9837908 | 0.1262249 |
| NC\_012920 | 16207 | 0.0454300 | 0.0000000 | 0.0000000 | 0.0000000 | 0.0886966 | 0.0475907 | 0.0000000 | 0.0000000 | 0.000 | 0.0371614 | 86 | 18 | 15 | 39 | 43 | 41 | 15 | 3 | 4 | 0.0141129 | 0.8182679 | 0.9552262 | 0.0147744 |
| NC\_012920 | 16209 | 0.0229854 | 0.0000000 | 0.0000000 | 0.0000000 | 0.0000000 | 0.0928019 | 0.0000000 | 0.0000000 | 0.000 | 0.0224690 | 86 | 18 | 15 | 39 | 43 | 41 | 15 | 3 | 4 | 0.0253204 | 0.8146235 | 0.9731163 | 0.0260199 |
| NC\_012920 | 16213 | 0.0000000 | 0.0000000 | 0.0000000 | 0.4049967 | 0.3309897 | 0.0000000 | 0.1244444 | 0.0000000 | 0.000 | 0.1464360 | 86 | 18 | 15 | 39 | 43 | 41 | 15 | 3 | 4 | 0.1749910 | 0.8279672 | 0.8540872 | 0.2048866 |
| NC\_012920 | 16218 | 0.0000000 | 0.0000000 | 0.0000000 | 0.0499671 | 0.0454300 | 0.0475907 | 0.0000000 | 0.0000000 | 0.000 | 0.0224690 | 86 | 18 | 15 | 39 | 43 | 41 | 15 | 3 | 4 | 0.0132156 | 0.8133896 | 0.9727411 | 0.0135859 |
| NC\_012920 | 16221 | 0.0000000 | 0.0000000 | 0.0000000 | 0.0000000 | 0.0000000 | 0.0475907 | 0.0000000 | 0.0000000 | 0.000 | 0.0075471 | 86 | 18 | 15 | 39 | 43 | 41 | 15 | 3 | 4 | 0.0206807 | 0.8110939 | 0.9908876 | 0.0208709 |
| NC\_012920 | 16222 | 0.0000000 | 0.0000000 | 0.0000000 | 0.0499671 | 0.0000000 | 0.0000000 | 0.1244444 | 0.0000000 | 0.000 | 0.0150367 | 86 | 18 | 15 | 39 | 43 | 41 | 15 | 3 | 4 | 0.0388726 | 0.8114382 | 0.9821894 | 0.0395775 |
| NC\_012920 | 16223 | 0.3807463 | 0.3456790 | 0.4800000 | 0.3550296 | 0.4780963 | 0.4330756 | 0.3200000 | 0.0000000 | 0.375 | 0.4067665 | 86 | 18 | 15 | 39 | 43 | 41 | 15 | 3 | 4 | 0.0256975 | 0.8855028 | 0.5524422 | 0.0465162 |
| NC\_012920 | 16224 | 0.0886966 | 0.0000000 | 0.0000000 | 0.4444444 | 0.3309897 | 0.0000000 | 0.1244444 | 0.0000000 | 0.000 | 0.1836260 | 86 | 18 | 15 | 39 | 43 | 41 | 15 | 3 | 4 | 0.1529956 | 0.8382404 | 0.8144541 | 0.1878504 |
| NC\_012920 | 16230 | 0.0454300 | 0.0000000 | 0.0000000 | 0.0000000 | 0.0454300 | 0.0000000 | 0.0000000 | 0.0000000 | 0.000 | 0.0224690 | 86 | 18 | 15 | 39 | 43 | 41 | 15 | 3 | 4 | 0.0120289 | 0.8159722 | 0.9727947 | 0.0123653 |
| NC\_012920 | 16239 | 0.0000000 | 0.1049383 | 0.0000000 | 0.0499671 | 0.0000000 | 0.0475907 | 0.0000000 | 0.0000000 | 0.000 | 0.0225535 | 86 | 18 | 15 | 39 | 42 | 41 | 15 | 3 | 4 | 0.0240633 | 0.8124738 | 0.9729090 | 0.0247333 |
| NC\_012920 | 16241 | 0.0886966 | 0.0000000 | 0.0000000 | 0.0000000 | 0.0000000 | 0.0000000 | 0.0000000 | 0.0000000 | 0.000 | 0.0299556 | 86 | 18 | 15 | 39 | 42 | 41 | 15 | 3 | 4 | 0.0317859 | 0.8192109 | 0.9645959 | 0.0329526 |
| NC\_012920 | 16243 | 0.0229854 | 0.0000000 | 0.0000000 | 0.0000000 | 0.0000000 | 0.0000000 | 0.1244444 | 0.0000000 | 0.000 | 0.0150935 | 86 | 18 | 15 | 39 | 42 | 41 | 15 | 3 | 4 | 0.0317844 | 0.8125895 | 0.9820159 | 0.0323665 |
| NC\_012920 | 16244 | 0.0000000 | 0.1049383 | 0.0000000 | 0.0499671 | 0.0000000 | 0.0475907 | 0.0000000 | 0.0000000 | 0.000 | 0.0225535 | 86 | 18 | 15 | 39 | 42 | 41 | 15 | 3 | 4 | 0.0240633 | 0.8124738 | 0.9729090 | 0.0247333 |
| NC\_012920 | 16249 | 0.0229854 | 0.0000000 | 0.2311111 | 0.0000000 | 0.0000000 | 0.0000000 | 0.0000000 | 0.0000000 | 0.000 | 0.0224690 | 86 | 18 | 15 | 39 | 43 | 41 | 15 | 3 | 4 | 0.0823369 | 0.8131313 | 0.9746425 | 0.0844790 |
| NC\_012920 | 16256 | 0.0000000 | 0.1049383 | 0.1244444 | 0.0000000 | 0.0454300 | 0.0000000 | 0.0000000 | 0.0000000 | 0.000 | 0.0225535 | 86 | 18 | 15 | 39 | 43 | 40 | 15 | 3 | 4 | 0.0375137 | 0.8117798 | 0.9732595 | 0.0385444 |
| NC\_012920 | 16261 | 0.1687399 | 0.1975309 | 0.0000000 | 0.1840894 | 0.0886966 | 0.0475907 | 0.0000000 | 0.0000000 | 0.375 | 0.1270661 | 86 | 18 | 15 | 39 | 43 | 41 | 15 | 3 | 4 | 0.0308122 | 0.8363751 | 0.8527564 | 0.0361325 |
| NC\_012920 | 16278 | 0.2230936 | 0.1975309 | 0.0000000 | 0.1420118 | 0.1297999 | 0.1356336 | 0.1244444 | 0.4444444 | 0.000 | 0.1658546 | 86 | 18 | 14 | 39 | 43 | 41 | 15 | 3 | 4 | 0.0228643 | 0.8440486 | 0.8079939 | 0.0282977 |
| NC\_012920 | 16290 | 0.0229854 | 0.1049383 | 0.0000000 | 0.0973044 | 0.0454300 | 0.0000000 | 0.0000000 | 0.0000000 | 0.000 | 0.0370239 | 86 | 18 | 15 | 39 | 43 | 42 | 15 | 3 | 4 | 0.0201122 | 0.8164044 | 0.9555622 | 0.0210475 |
| NC\_012920 | 16291 | 0.0454300 | 0.0000000 | 0.1244444 | 0.0000000 | 0.0454300 | 0.0000000 | 0.0000000 | 0.0000000 | 0.000 | 0.0297330 | 86 | 18 | 15 | 39 | 43 | 42 | 15 | 3 | 4 | 0.0193056 | 0.8165753 | 0.9642911 | 0.0200205 |
| NC\_012920 | 16292 | 0.0673337 | 0.0000000 | 0.0000000 | 0.0000000 | 0.0000000 | 0.0907029 | 0.0000000 | 0.0000000 | 0.000 | 0.0370239 | 86 | 18 | 15 | 39 | 43 | 42 | 15 | 3 | 4 | 0.0215159 | 0.8195657 | 0.9557970 | 0.0225109 |
| NC\_012920 | 16293 | 0.0229854 | 0.0000000 | 0.0000000 | 0.0000000 | 0.0000000 | 0.0464853 | 0.3200000 | 0.0000000 | 0.000 | 0.0370239 | 86 | 18 | 15 | 39 | 43 | 42 | 15 | 3 | 4 | 0.1103007 | 0.8148095 | 0.9595733 | 0.1149477 |
| NC\_012920 | 16294 | 0.1297999 | 0.1975309 | 0.2311111 | 0.0973044 | 0.0454300 | 0.0464853 | 0.1244444 | 0.0000000 | 0.000 | 0.1067996 | 86 | 18 | 15 | 39 | 43 | 42 | 15 | 3 | 4 | 0.0194145 | 0.8303880 | 0.8738829 | 0.0222163 |
| NC\_012920 | 16295 | 0.0454300 | 0.0000000 | 0.0000000 | 0.0000000 | 0.0886966 | 0.0464853 | 0.1244444 | 0.0000000 | 0.000 | 0.0442577 | 86 | 18 | 15 | 39 | 43 | 42 | 15 | 3 | 4 | 0.0160572 | 0.8188822 | 0.9468213 | 0.0169591 |
| NC\_012920 | 16296 | 0.1095187 | 0.1049383 | 0.2311111 | 0.0000000 | 0.0000000 | 0.0464853 | 0.1244444 | 0.0000000 | 0.000 | 0.0726237 | 86 | 18 | 15 | 39 | 43 | 42 | 15 | 3 | 4 | 0.0338823 | 0.8245212 | 0.9149045 | 0.0370337 |
| NC\_012920 | 16298 | 0.0000000 | 0.2777778 | 0.0000000 | 0.0499671 | 0.0454300 | 0.0000000 | 0.0000000 | 0.0000000 | 0.000 | 0.0370239 | 86 | 18 | 15 | 39 | 43 | 42 | 15 | 3 | 4 | 0.0926597 | 0.8137558 | 0.9587183 | 0.0966496 |
| NC\_012920 | 16300 | 0.0229854 | 0.1975309 | 0.0000000 | 0.0499671 | 0.0000000 | 0.0464853 | 0.1244444 | 0.0000000 | 0.000 | 0.0442577 | 86 | 18 | 15 | 39 | 43 | 42 | 15 | 3 | 4 | 0.0365131 | 0.8161766 | 0.9477543 | 0.0385259 |
| NC\_012920 | 16304 | 0.0454300 | 0.1049383 | 0.1244444 | 0.0000000 | 0.0454300 | 0.0464853 | 0.1244444 | 0.0000000 | 0.375 | 0.0585546 | 86 | 18 | 15 | 39 | 43 | 42 | 15 | 3 | 4 | 0.0375017 | 0.8187113 | 0.9311616 | 0.0402741 |
| NC\_012920 | 16309 | 0.0454300 | 0.1049383 | 0.0000000 | 0.0973044 | 0.1297999 | 0.0464853 | 0.0000000 | 0.0000000 | 0.375 | 0.0726237 | 86 | 18 | 15 | 39 | 43 | 42 | 15 | 3 | 4 | 0.0322557 | 0.8222428 | 0.9145250 | 0.0352704 |
| NC\_012920 | 16311 | 0.3442401 | 0.1975309 | 0.2311111 | 0.4838922 | 0.4023797 | 0.2448980 | 0.4444444 | 0.0000000 | 0.000 | 0.3584478 | 86 | 18 | 15 | 39 | 43 | 42 | 15 | 3 | 4 | 0.0551150 | 0.8767533 | 0.6136975 | 0.0898081 |
| NC\_012920 | 16318 | 0.0229854 | 0.0000000 | 0.0000000 | 0.0000000 | 0.0886966 | 0.0464853 | 0.0000000 | 0.0000000 | 0.375 | 0.0370239 | 86 | 18 | 15 | 39 | 43 | 42 | 15 | 3 | 4 | 0.0579170 | 0.8162051 | 0.9572662 | 0.0605025 |
| NC\_012920 | 16319 | 0.0000000 | 0.0000000 | 0.1244444 | 0.0973044 | 0.0886966 | 0.0907029 | 0.0000000 | 0.0000000 | 0.000 | 0.0514347 | 86 | 18 | 15 | 39 | 43 | 42 | 15 | 3 | 4 | 0.0253241 | 0.8173158 | 0.9386625 | 0.0269790 |
| NC\_012920 | 16320 | 0.0673337 | 0.0000000 | 0.0000000 | 0.0000000 | 0.0454300 | 0.0464853 | 0.0000000 | 0.0000000 | 0.000 | 0.0370239 | 86 | 18 | 15 | 39 | 43 | 42 | 15 | 3 | 4 | 0.0116961 | 0.8196511 | 0.9553581 | 0.0122426 |
| NC\_012920 | 16325 | 0.0673337 | 0.0000000 | 0.0000000 | 0.0000000 | 0.0000000 | 0.1326531 | 0.0000000 | 0.0000000 | 0.375 | 0.0514347 | 86 | 18 | 15 | 39 | 43 | 42 | 15 | 3 | 4 | 0.0563501 | 0.8207049 | 0.9408602 | 0.0598921 |
| NC\_012920 | 16335 | 0.0229854 | 0.0000000 | 0.0000000 | 0.0000000 | 0.0000000 | 0.0464853 | 0.0000000 | 0.0000000 | 0.000 | 0.0149804 | 86 | 18 | 15 | 39 | 43 | 42 | 15 | 3 | 4 | 0.0102489 | 0.8137843 | 0.9817803 | 0.0104391 |
| NC\_012920 | 16343 | 0.0454300 | 0.1049383 | 0.0000000 | 0.0000000 | 0.0454300 | 0.0000000 | 0.0000000 | 0.0000000 | 0.000 | 0.0297330 | 86 | 18 | 15 | 39 | 43 | 42 | 15 | 3 | 4 | 0.0164852 | 0.8166607 | 0.9641922 | 0.0170975 |
| NC\_012920 | 16344 | 0.0229854 | 0.0000000 | 0.0000000 | 0.0499671 | 0.0000000 | 0.0000000 | 0.0000000 | 0.0000000 | 0.000 | 0.0149804 | 86 | 18 | 15 | 39 | 43 | 42 | 15 | 3 | 4 | 0.0111716 | 0.8136988 | 0.9817954 | 0.0113788 |
| NC\_012920 | 16352 | 0.0000000 | 0.1049383 | 0.0000000 | 0.0499671 | 0.0000000 | 0.0464853 | 0.0000000 | 0.0000000 | 0.000 | 0.0223852 | 86 | 18 | 15 | 39 | 43 | 42 | 15 | 3 | 4 | 0.0239524 | 0.8129299 | 0.9731231 | 0.0246140 |
| NC\_012920 | 16353 | 0.0000000 | 0.1049383 | 0.0000000 | 0.0499671 | 0.0000000 | 0.0464853 | 0.0000000 | 0.0000000 | 0.000 | 0.0223852 | 86 | 18 | 15 | 39 | 43 | 42 | 15 | 3 | 4 | 0.0239524 | 0.8129299 | 0.9731231 | 0.0246140 |
| NC\_012920 | 16354 | 0.0000000 | 0.0000000 | 0.1244444 | 0.0000000 | 0.0000000 | 0.0000000 | 0.0000000 | 0.0000000 | 0.000 | 0.0075187 | 86 | 18 | 15 | 39 | 43 | 42 | 15 | 3 | 4 | 0.0631313 | 0.8105945 | 0.9913101 | 0.0636847 |
| NC\_012920 | 16355 | 0.1095187 | 0.0000000 | 0.2311111 | 0.1840894 | 0.0000000 | 0.0907029 | 0.4444444 | 0.4444444 | 0.000 | 0.1331150 | 86 | 18 | 15 | 39 | 43 | 42 | 15 | 3 | 4 | 0.0964180 | 0.8302172 | 0.8551219 | 0.1127535 |
| NC\_012920 | 16356 | 0.0454300 | 0.0000000 | 0.0000000 | 0.0000000 | 0.0454300 | 0.0464853 | 0.1244444 | 0.0000000 | 0.375 | 0.0442577 | 86 | 18 | 15 | 39 | 43 | 42 | 15 | 3 | 4 | 0.0467919 | 0.8178284 | 0.9484160 | 0.0493369 |
| NC\_012920 | 16357 | 0.0000000 | 0.0000000 | 0.0000000 | 0.0000000 | 0.0000000 | 0.0464853 | 0.0000000 | 0.0000000 | 0.000 | 0.0075187 | 86 | 18 | 15 | 39 | 43 | 42 | 15 | 3 | 4 | 0.0201118 | 0.8113635 | 0.9909196 | 0.0202961 |
| NC\_012920 | 16359 | 0.0229854 | 0.1049383 | 0.0000000 | 0.0000000 | 0.0454300 | 0.0000000 | 0.0000000 | 0.0000000 | 0.000 | 0.0223852 | 86 | 18 | 15 | 39 | 43 | 42 | 15 | 3 | 4 | 0.0190412 | 0.8142969 | 0.9730332 | 0.0195689 |
| NC\_012920 | 16362 | 0.3309897 | 0.2777778 | 0.3911111 | 0.3261012 | 0.2055165 | 0.1723356 | 0.4444444 | 0.4444444 | 0.375 | 0.3014311 | 86 | 18 | 15 | 39 | 43 | 42 | 15 | 3 | 4 | 0.0282202 | 0.8659594 | 0.6617340 | 0.0426459 |
| NC\_012920 | 16390 | 0.0673337 | 0.3456790 | 0.1244444 | 0.0973044 | 0.1297999 | 0.0000000 | 0.1244444 | 0.0000000 | 0.000 | 0.1000783 | 86 | 18 | 15 | 39 | 43 | 42 | 15 | 3 | 4 | 0.0527223 | 0.8252047 | 0.8851170 | 0.0595653 |
| NC\_012920 | 16391 | 0.0229854 | 0.0000000 | 0.0000000 | 0.0000000 | 0.0000000 | 0.0000000 | 0.0000000 | 0.0000000 | 0.000 | 0.0075187 | 86 | 18 | 15 | 39 | 43 | 42 | 15 | 3 | 4 | 0.0078841 | 0.8126166 | 0.9908205 | 0.0079571 |
| NC\_012920 | 16399 | 0.0000000 | 0.0000000 | 0.0000000 | 0.0000000 | 0.0000000 | 0.0000000 | 0.1244444 | 0.0000000 | 0.000 | 0.0075187 | 86 | 18 | 15 | 39 | 43 | 42 | 15 | 3 | 4 | 0.0631313 | 0.8105945 | 0.9913101 | 0.0636847 |
| NC\_012920 | 16451 | 0.0229854 | 0.1049383 | 0.0000000 | 0.0499671 | 0.0000000 | 0.0000000 | 0.0000000 | 0.0000000 | 0.000 | 0.0225535 | 86 | 18 | 15 | 39 | 42 | 41 | 15 | 3 | 4 | 0.0197601 | 0.8137750 | 0.9728330 | 0.0203119 |
