# Supplementary material for: Genomic Landscape of the Mitochondrial Genome in the United Arab Emirates Native Population
Source: Genes (Basel). 2020 Aug 1;11(8):876. doi: 10.3390/genes11080876 (PMC7464197; doi:10.3390/genes11080876)
Supplement: Supplementary file 1 [file genes-11-00876-s001.zip › sup_files/Table_S4.html]

| Gst | Htmax | Gstmax | Gprimest |
| --- | --- | --- | --- |
| 0.0200404 | 0.8113905 | 0.9907453 | 0.0202276 |
| 0.0661784 | 0.8205524 | 0.9084515 | 0.0728475 |
| 0.0350905 | 0.8400152 | 0.8160276 | 0.0430016 |
| 0.0237048 | 0.8165899 | 0.9729339 | 0.0243642 |
| 0.0279421 | 0.8797564 | 0.6098248 | 0.0458199 |
| 0.0185100 | 0.8160881 | 0.9551402 | 0.0193794 |
