# Supplementary material for: Genomic Landscape of the Mitochondrial Genome in the United Arab Emirates Native Population
Source: Genes (Basel). 2020 Aug 1;11(8):876. doi: 10.3390/genes11080876 (PMC7464197; doi:10.3390/genes11080876)
Supplement: Supplementary file 1 [file genes-11-00876-s001.zip › sup_files/Figure_S2.pdf]

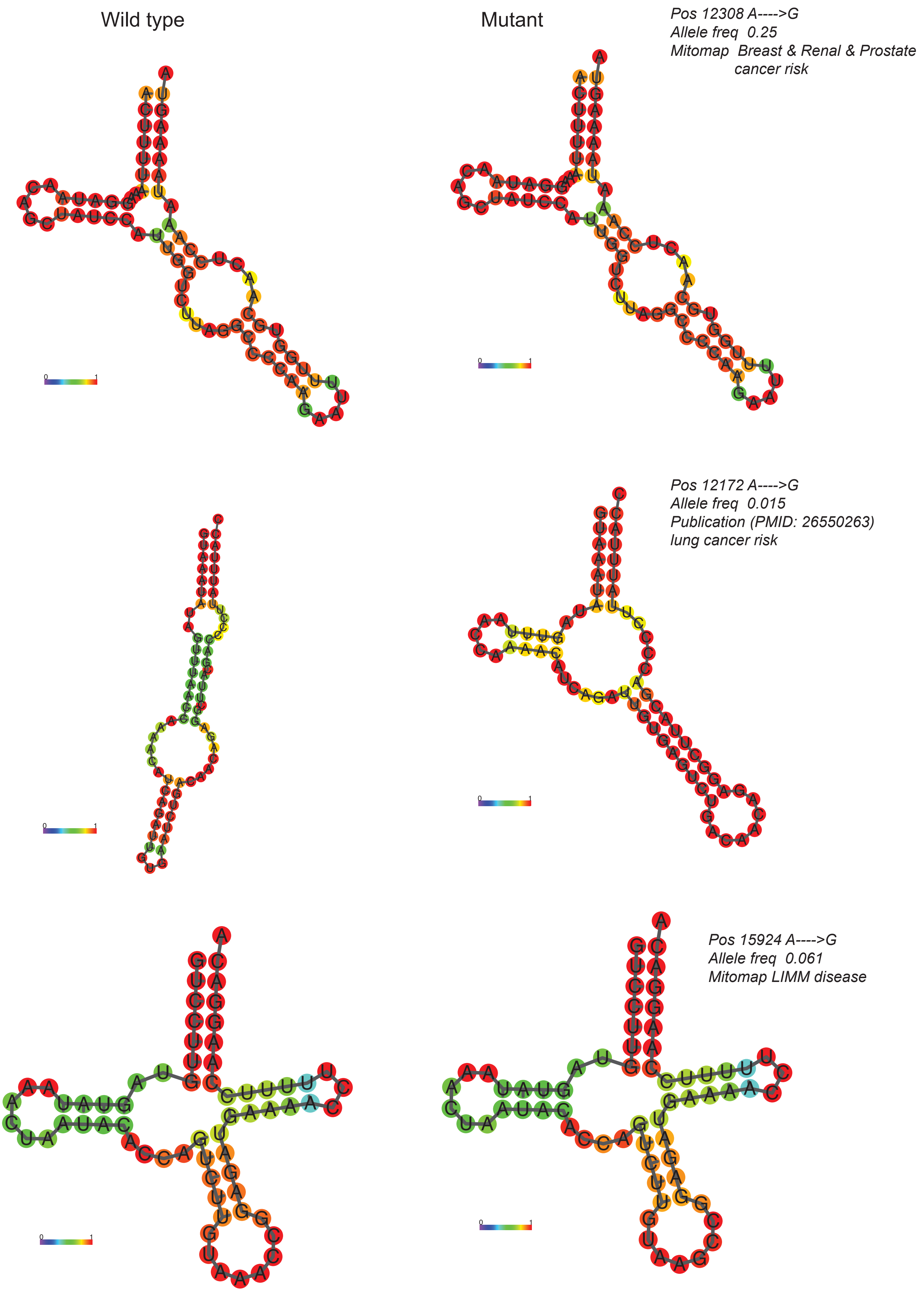

Wild type
Mutant
Pos 12308 A---->G
Allele freq 0.25
Mitomap Breast & Renal & Prostate cancer risk
Pos 12172 A---->G
Allele freq 0.015
Publication (PMID: 26550263)
lung cancer risk
Pos 15924 A---->G
Allele freq 0.061
Mitomap LIMM disease
