# Supplementary figures and images for: Genomic Landscape of the Mitochondrial Genome in the United Arab Emirates Native Population
Source: Genes (Basel). 2020 Aug 1;11(8):876. doi: 10.3390/genes11080876 (PMC7464197; doi:10.3390/genes11080876)

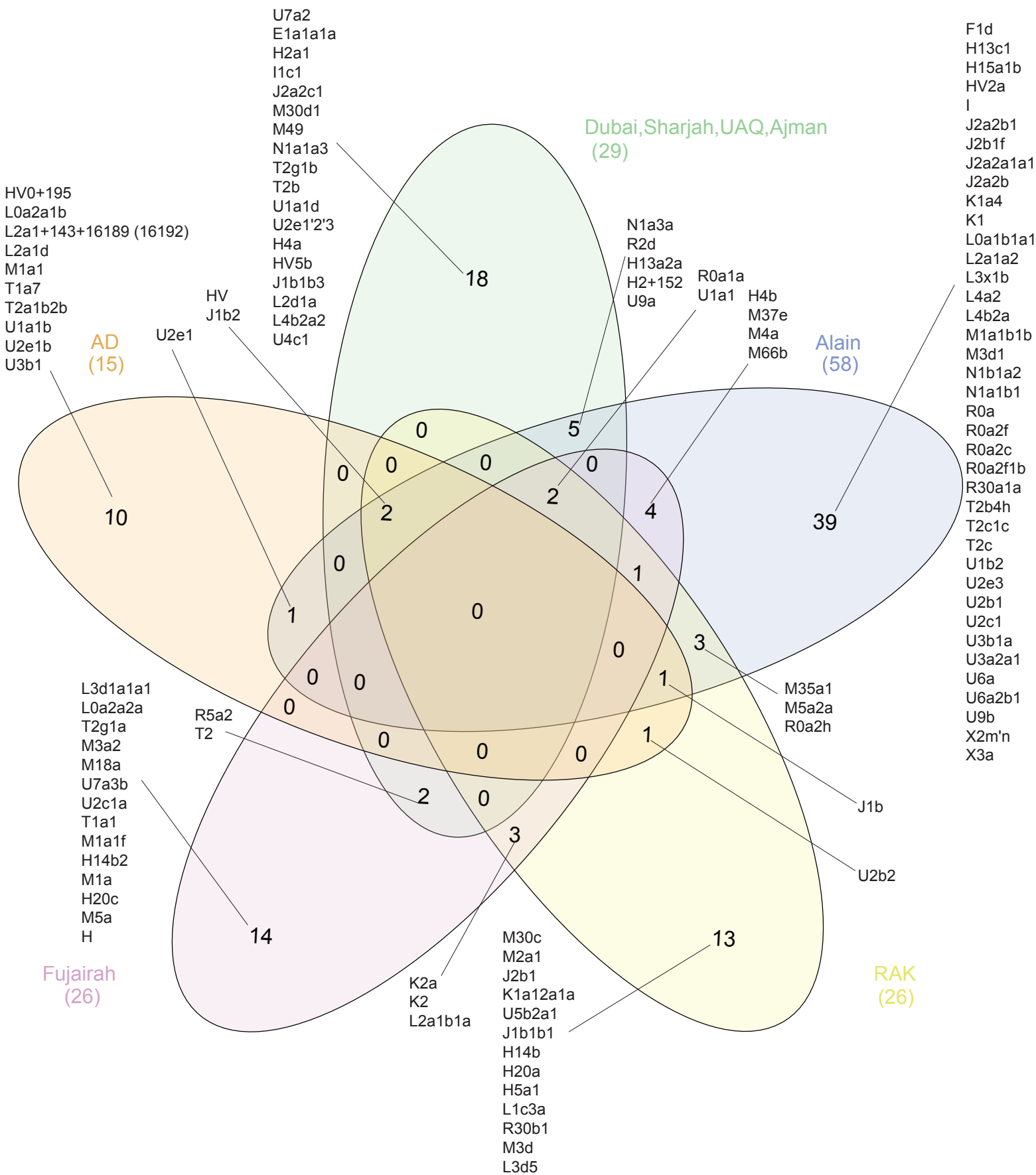

Supplement: Supplementary file 1 [file genes-11-00876-s001.zip › sup_files/Figure_S1.pdf]
